# Burden of ageing spectrum of diseases in China, 1990–2021: a systematic analysis of global burden of disease study 2021

**DOI:** 10.3389/fpubh.2025.1611901

**Published:** 2025-07-29

**Authors:** Li Yang, Yuhan Xing, Xinjing Gao, Cui Guo, Dongze Wu

**Affiliations:** ^1^Department of Dermatology, Guangzhou Women and Children’s Medical Center, Guangzhou Medical University, Guangzhou, China; ^2^School of Public Health (Shenzhen), Sun Yat-sen University, Shenzhen, China; ^3^Department of Urban Planning and Design, The University of Hong Kong, Hong Kong, Hong Kong SAR, China; ^4^Department of Rheumatology and Immunology, Sichuan Provincial People's Hospital, School of Medicine, University of Electronic Science and Technology of China, Chengdu, China

**Keywords:** ageing, disease burden, risk factor, China, DALYs

## Abstract

**Background:**

Population aging represents a pressing challenge for China, given its vast population and the growing proportion of adults aged 65 years and older. The study aimed to assess burden of ageing spectrum of diseases in China from 1990 to 2021.

**Methods:**

The main outcome measures were disability-adjusted life years (DALYs), summary exposure values (SEVs), and attributable risk. The average annual percent change (AAPC) in number and age specific rate (ASR) were calculated to quantify the temporal trends. The decomposition analysis was utilized to assess the impact of population aging on the burden of disease.

**Results:**

From 1990 to 2021, The ASR of all-cause DALYs in China decreased by 35.94% overall and by 33.99% among individuals ≥65 years. However, the number of DALYs attributed to population ageing among people aged 65 and older has been increasing by 10.45 million. Chronic obstructive pulmonary disease, stroke, and ischemic heart disease are most significantly affected by the aging trend. Notably, the older adults population exhibited the most pronounced increase in HIV/AIDS-related DALYs, especially in the 70–74 years group (AAPC: 7.96, 95% CI 6.69, 9.24). Additionally, notable changes in COVID-19-related DALYs became evident beginning in 2019. Regarding risk factors, particulate matter pollution, smoking, and high sodium diets emerged as the top three contributors to health risks among the older adults.

**Conclusion:**

To address burden of ageing spectrum of diseases, China should leverage complementary roles of public and private insurance to manage cardiovascular and pulmonary disease, promote healthy environments and diets and prioritize prevention of HIV/AIDS.

## Introduction

The acceleration of global population aging is expected to continue as global life expectancy rises from 2022 to 2050 (1–3). However, the pace may be somewhat slower compared to the three decades prior to the onset of the COVID-19 pandemic in 2020 (4). By 2021, the proportion of the Chinese population aged 65 and older had surpassed 14%, officially marking the country’s entry into an “aged society.” (5) Unlike younger populations, who primarily face acute illnesses and injuries, older adults are more vulnerable to non-communicable diseases (NCDs) such as cardiovascular diseases, chronic respiratory conditions, neurodegenerative disorders, and cancers (6). Understanding how the spectrum of aging-related diseases has evolved over time is essential for planning effective healthcare policies and interventions.

Despite increasing awareness of the challenges posed by an aging population, there remains a gap in comprehensive, long-term assessments of the burden of aging-related diseases in China (7). The Global Burden of Disease (GBD) Study provides a valuable opportunity to address this gap by offering standardized and longitudinal data on disease burden, risk factors, and trends (8–10).

This study systematically evaluates the disease burden of ageing-related conditions in China from 1990 to 2021 through three analytical objectives: (i) evaluate the long-term impact of population aging (populations aged ≥65 years), (ii) identify high-burden diseases, (iii) assess key risk factors. The study highlights the need for proactive strategies, including disease prevention, healthcare system strengthening, and risk factor mitigation. Addressing these challenges is not only a public health priority but also a key step in ensuring the sustainability of China’s healthcare system in the face of rapid demographic change.

## Methods

### Data source

The GBD study provides comprehensive estimates on a wide range of health metrics, including causes of death and injury, risk factors, etiologies, impairments, SEVs, healthy life expectancy (HALE), injuries by nature, all-cause mortality, fertility, and population. Data for this study were obtained from the Global Health Data Exchange (GHDx) query tool^1^ for the period 1990 to 2021. The methods and estimates utilized in this analysis are publicly accessible through the Institute for Health Metrics and Evaluation (IHME) website, including the GBD Compare tool^2^ and the GBD Results Tool (see text footnote 1).

### Definition of older adults population

The study focused on the older adults population, defined as individuals aged 65 years or older, stratified into seven distinct age groups: 65–69 years, 70–74 years, 75–79 years, 80–84 years, 85–89 years, 90–94 years, and 95 years and older.

### Definition of cause

The study categorized causes into hierarchical framework comprising four levels. Level 1 encompassed broad categories, including communicable, maternal, neonatal, and nutritional diseases, injuries, NCDs, and outcomes related to the COVID-19 pandemic. Level 2 subdivided these into 22 specific clusters, providing greater granularity. Level 3 further disaggregated level 2 clusters into 175 distinct single causes. Finally, level 4 refined this classification, identifying 170 specific causes for detailed analysis.

### Definition of risk factors

The GBD study employs a hierarchical framework to classify risk factors across four levels. Level 1 categorizes risks into four broad domains: behavioral, environmental and occupational, and metabolic factors. Level 2 expands this classification to include 20 specific risks or clusters of risks. Level 3 further disaggregates these into 42 distinct risks or clusters. Finally, level 4 provides the most detailed classification, encompassing 69 specific risk factors.

### Decomposition analysis

The study conducted a decomposition analysis of DALYs and SEVs to assess the relative contributions of aging, population growth, and epidemiological shifts in shaping disease patterns over the past three decades.

### Projection analysis

The study utilized the Bayesian age-period-cohort (BAPC) model to project the number and rate of DALYs and SEVs from 2020 to 2045. These projections were performed using the BAPC and INLA packages in the R statistical environment.

### Joinpoint analysis

The Joinpoint regression model was employed to evaluate trends in disease burden from 1990 to 2021, using annual percent change (APC) and average annual percent change (AAPC) metrics. Analyses were conducted using the Joinpoint Regression Program (Version 4.8.0.1, Statistical Methodology and Applications Branch, Surveillance Research Program, National Cancer Institute). Trends were assessed for the entire period (1990–2021) as well as for three segmented intervals (1990–2000, 2001–2011, and 2012–2021) to capture both overall and interval-specific trends.

## Result

### The transition of population composition in China

In the past three decades, the population in China is increasing, but the population composition is changing. The proportion of the population aged ≥65 years has expanded substantially, despite those aged 0–14 years having declined (Supplementary Figure 1).

### Temporal trends of disease burden in the older adults

**Figure 1:**
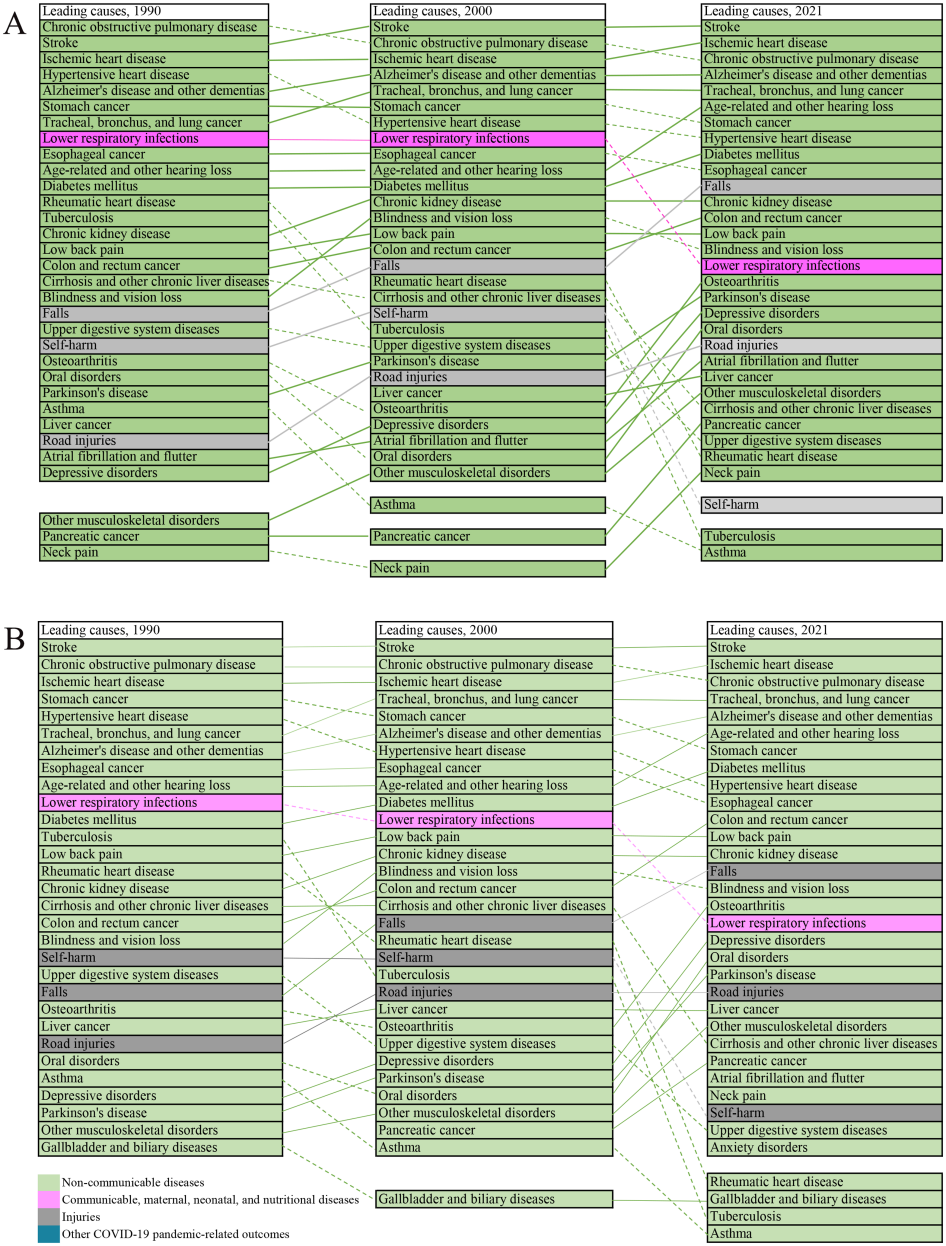
Leading level 3 causes among older adults in China, 1990–2021. Top 30 causes according to age specific rate of DALYs **(A)**. Top 30 causes according to the number of DALYs **(B)**. Lines connect ranking of causes across the time points (1990, 2021). Solid lines represent stable or increased rankings; dashed lines represent decreased ranking. Data in parentheses are 95% uncertainty intervals.

The number of DALYs in the older adults (≥65 years) significantly increased for all causes and level 1 causes between 1990 and 2021 (Supplementary Figures 2–5). Conversely, the rate of DALYs in the older adults significantly decreased for all causes between 1990 and 2021 (Supplementary Figure 2). Among level 2 causes, NCDs and injuries became the primary health challenge for older adults, characterized by persistently rising in number of DALYs and a slower decline in age specific rate (ASR) of DALYs (Figure 1, Supplementary Figures 3–5). In addition, the analysis further indicates a consistently higher disease burden in male than female in older adults population, underscoring the need for gender-specific interventions.

We analyzed 30 leading level 3 causes of DALYs among older adults in 1990, and 2021(Figures 1A,B). Stroke, COPD, and IHD constituted the top three causes of DALYs throughout the study period. A longitudinal analysis from 1990 to 2021 shows that 18 of the top 30 diseases increased in ranking based on the ASR of DALYs (Figure 1A), and 17 of the top 30 diseases increased in ranking based on the number of DALYs (Figure 1B).

NCDs persisted the leading contributors to the overall disease burden, accompanied by substantial reordering in the rankings of the top 30 level 3 causes: lower respiratory infections (from 8th in 1990 to 16th in 2021) and self-harm (from 10th in 1990 to 30th in 2021) declined substantially in ranking, while falls (from 19th in 1990 to 11st in 2021) and road injuries (from 27th to 21st) increased in ranking. By 2021, pancreatic cancer, other musculoskeletal disorders, and neck pain had entered the top 30 causes, whereas gallbladder and biliary diseases, tuberculosis, and asthma dropped out of the top 30 causes (Figure 1A).

### The age effect of disease burden in the older adults

An increasing trend in the number of DALYs was observed across nearly all causes and level 1 causes (AAPC > 0), except for the 65–79 years age group—specifically the 65–69 years, 70–74 years, and 75–79 years subgroups—within the category of communicable, maternal, neonatal, and nutritional diseases (AAPC < 0) (Table 1).

**Table 1:** The age specific rate and number of disability-adjusted life year attributable to all cause and level 1 cause and average annual percent change according to gender, from 65-69 to 95+, 1990 to 2021.

	**1990**	**2021**	**1990-2021**	**1990-2000**	**2001-2011**	**2012-2021**
Age group	N(95%UI)	N(95%UI)	AAPC (95%CI)	APC (95%CI)	APC (95%CI)	APC (95%CI)
Age specific rate						
All causes						
65-69 years	94,928.55(84,344.48-105,016.80)	58,709.87(50,106.15-67,666.75)	-1.55(-1.60, -1.51)	-1.39(-1.46, -1.32)	-1.88(-1.93, -1.79)	-1.47(-1.62, -1.33)
70-74 years	131,041.66(11,8113.31-143,569.26)	81,588.96(70,594.17-92,545.32)	-1.52(-1.56, -1.49)	-1.28(-1.35, -1.22)	-2.12(-2.17, -2.07)	-1.22(-1.34, -1.10)
75-79 years	163,651.72(148,937.42-178,431.74)	105,736.70(92,515.73-119,244.92)	-1.42(-1.46, -1.35)	-1.20(-1.29, -1.12)	-1.70(-1.76, -1.59)	-1.42(-1.62, -1.20)
80-84 years	207,749.88(191,921.93-223,484.29)	145,157.63(129,289.04-160,355.82)	-1.16(-1.22, -1.09)	-0.96(-1.08, -0.85)	-1.17(-1.26, -1.02)	-1.30(-1.50, -1.09)
85-89 years	285,100.41(267,260.23-303,090.66)	206,620.08(187259.17-226,194.13)	-1.15(-1.25, -1.05)	-0.26(-0.50, -0.03)	-1.76(-1.92, -1.61)	-1.71(-1.92, -1.44)
90-94 years	385,755.90(364,554.64-407,790.69)	271,677.57(246,874.84-298,541.21)	-1.19(-1.33, -1.08)	-0.13(-0.39, 0.20)	-1.49(-1.70, -1.30)	-2.06(-2.38, -1.83)
95+ years	441,027.51(419,525.01-458,801.59)	322,760.60(290,563.73-358,158.24)	-0.99(-1.10, -0.88)	-1.12(-1.33, -0.95)	-0.78(-1.09, -0.60)	-0.66(-1.09, -0.28)
Communicable, maternal, neonatal, and nutritional diseases						
65-69 years	4,412.50(3,843.77-5,129.56)	1,154.43(966.79-1,359.74)	-4.27(-4.35, -4.19)	-4.73(-4.92, -4.56)	-5.6(-5.80, -5.40)	-2.26(-2.59, -2.01)
70-74 years	6,023.51(5,230.10-6,778.85)	1,588.84(1,341.59-1,852.52)	-4.22(-4.28, -4.16)	-4.27 (-4.37, -4.16)	-5.45 (-5.62, -5.33)	-2.73 (-3.03, -2.53)
75-79 years	7,881.55(6,827.57-8,830.17)	2,182.77(1,872.39-2,530.14)	-4.04 (-4.11, -3.97)	-4.22 (-4.40, -4.05)	-4.99 (-5.11, -4.87)	-2.83 (-3.04, -2.65)
80-84 years	11,464.95(9,500.20-12,847.34)	3,675.95(3,100.62-4,299.69)	-3.64 (-3.71, -3.57)	-3.57(-3.72, -3.39)	-4.68(-4.79, -4.58)	-2.29(-2.51, -2.08)
85-89 years	17,278.72(14,600.64-19,210.43)	6,406.95(5,416.25-7,663.64)	-3.19 (-3.28, -3.09)	-2.36 (-2.50, -2.21)	-4.73 (-4.88, -4.62)	-2.42 (-2.70, -2.09)
90-94 years	30,845.93(26,050.28-34,443.76)	11,168.37(8,917.92-13,599.31)	-3.29 (-3.45, -3.14)	-2.31 (-2.58, -2.02)	-4.43 (-4.73, -4.24)	-2.85 (-3.37, -2.36)
95+ years	43,594.14(33,394.54-50,112.64)	16,211.13(12,065.05-20,429.43)	-3.11 (-3.19, -3.03)	-2.88 (-3.12, -2.67)	-4.07 (-4.31, -3.89)	-1.82 (-2.10, -1.57)
**Injuries**						
65-69 years	4,256.75(3,654.61-4,830.18)	2,889.44(2,436.78-3,461.93)	-1.18(-1.35, -1.04)	-0.53(-0.85, -0.07)	-1.36 (-1.64, -1.02)	-1.72 (-2.20, -1.46)
70-74 years	4,700.41(4079.01-5327.65)	3,288.92(2,753.41-3,948.27)	-1.06(-1.19, -0.93)	-0.47(-0.64, -0.26)	-1.96(-2.25, -1.51)	-0.82(-1.35, -0.2)
75-79 years	5,110.56(4,433.24-5,757.59)	3,801.31(3,128.12-4,540.30)	-0.93(-1.10, -0.75)	-0.21(-0.43, 0.07)	-1.73(-2.08, -1.20)	-0.92(-1.61, -0.16)
80-84 years	5,951.08(5,252.43-6,725.61)	4,861.69(3,940.96-5,770.51)	-0.55(-0.69, -0.40)	0.21(0.01, 0.45)	-1.09(-1.54, -0.76)	-0.49(-1.18, 0.07)
85-89 years	7,935.87(6945.19-9,067.33)	7,171.70(5,677.46-8,495.89)	-0.3(-0.42, -0.18)	0.46(0.22, 0.67)	-1.12(-1.41, -0.89)	-0.35(-0.77, 0.11)
90-94 years	10,134.50(8,843.19-11,599.49)	9,709.42(7,258.17-11,661.58)	-0.14(-0.22, -0.04)	0.19(0.02, 0.34)	-0.76(-0.99, -0.55)	0.08(-0.33, 0.40)
95+ years	10,943.36(9,243.47-12,637.18)	12,183.52(8,584.73-15,323.59)	0.45(0.33, 0.57)	-0.48(-0.74, -0.09)	-0.14(-0.55, 0.30)	1.91(1.28, 2.36)
Non-communicable diseases						
65-69 years	86,259.29(76,651.96-95,639.01)	5,4610.43(46,660.94-62,862.38)	-1.50(-1.55, -1.46)	-1.28(-1.36, -1.19)	-1.68(-1.83, -1.61)	-1.58(-1.73, -1.44)
70-74 years	120,317.75(108,462.61-131,791.94)	76,641.60(66,347.80-86,738.43)	-1.46(-1.49, -1.42)	-1.18(-1.24, -1.11)	-2.04(-2.09, -2.00)	-1.21(-1.31, -1.09)
75-79 years	150,659.61(137,317.79-164,192.41)	99,671.95(87,120.97-112,391.28)	-1.34(-1.39, -1.28)	-1.09(-1.18, -1.00)	-1.61(-1.67, -1.48)	-1.42(-1.63, -1.19)
80-84 years	190,333.84(176,825.05-20,5345.05)	136,513.68(121,797.29-151,033.88)	-1.07(-1.14, -1.01)	-0.84(-0.96, -0.74)	-1.05(-1.13, -0.90)	-1.31(-1.51, -1.09)
85-89 years	259,885.82(243,453.94-276,107.08)	192,899.03(175,264.69-211,551.00)	-1.00(-1.09, -0.89)	-0.15(-0.32, 0.01)	-1.51(-1.63, -1.31)	-1.49(-1.81, -1.12)
90-94 years	344,775.46(325,458.46-365,881.81)	250,552.02(227,148.27-275,588.01)	-1.06(-1.18, -0.96)	-0.22(-0.42, -0.04)	-1.26(-1.39, -1.02)	-1.76(-2.15, -1.46)
95+ years	386,490.00(36,6153.06-40,6128.18)	293,983.05(264,955.28-329,772.36)	-0.86(-0.96, -0.76)	-0.95(-1.15, -0.78)	-0.56(-0.85, -0.38)	-0.71(-1.08, -0.36)
**Other COVID-19 pandemic-related outcomes**						
65-69 years	NA	55.57(6.45-256.89)	NA	NA	NA	NA
70-74 years	NA	69.61(9.90-310.28)	NA	NA	NA	NA
75-79 years	NA	80.67(13.16-354.65)	NA	NA	NA	NA
80-84 years	NA	106.31(22.47-437.23)	NA	NA	NA	NA
85-89 years	NA	142.40(33.99-536.50)	NA	NA	NA	NA
90-94 years	NA	247.76(63.63-776.03)	NA	NA	NA	NA
95+ years	NA	382.89(66.98-1,198.18)	NA	NA	NA	NA
**Number**						
**All causes**						
65-69 years	25,898,227(23,010,701-28,650,486)	45,032,531(38,433,174-51,902,773)	1.86(1.50,2.22)	1.29(1.12,1.45)	0.11(-0.89,1.12)	4.42(4.13,4.71)
70-74 years	24,658,962(22,226,149-27,016,361)	43,484,013(37,624,180-49,323,364)	1.85(1.71,2.00)	1.89(1.64,2.15)	0.40(0.23,0.56)	3.64(3.40,3.88)
75-79 years	18,624,706(16,950,116-20,306,775)	35,018,869(30,640,226-39,492,651)	2.05(1.69,2.40)	2.39(2.24,2.54)	2.39(2.24,2.54)	1.20(0.05,2.37)
80-84 years	11,004,771(10,166,345-11,838,243)	28,729,389(25,588,701-31,737,392)	3.21(2.81,3.62)	3.55(3.38,3.73)	3.32(2.11,4.56)	2.73(2.56,2.91)
85-89 years	4,809,232(4,508,294-5,112,701)	19,682,114(17,837,842-21,546,689)	4.50(3.90,5.10)	5.06(4.33,5.78)	3.55(1.98,5.14)	4.71(4.49,4.93)
90-94 years	1,183,584(1,118,534-1,251,191)	7,965,552(7,238,339-8,753,190)	6.37(5.91,6.83)	7.52(6.96,8.09)	5.78(4.55,7.02)	5.98(5.75,6.21)
95+ years	178,581(169,874-185,778)	2,062,757(1,856,988-2,288,983)	8.55(7.72,9.39)	7.38(6.81,7.95)	8.20(5.84,10.62)	10.08(9.48,10.69)
**Communicable, maternal, neonatal, and nutritional diseases**						
65-69 years	1,203,810(1,048,651-1,399,437)	885,485(741,560-1,042,971)	-0.91(-1.64, -0.17)	-2.23(-2.49, -1.98)	-3.96(-6.09, -1.79)	3.77(3.25,4.29)
70-74 years	1,133,482(984,181-1,275,619)	846,793(715,018-987,329)	-0.91(-1.23, -0.59)	-1.28(-1.46, -1.10)	-3.14(-3.68, -2.60)	2.13(1.46,2.81)
75-79 years	896,975(777,025-1,004,934)	722,910(620,115-837,955)	-0.81(-1.24, -0.37)	-1.04(-1.35, -0.72)	-1.25(-2.43, -0.05)	-0.19(-0.38,0.01)
80-84 years	607,312(503,237-680,539)	727,538(613,669-850,988)	0.63(0.21,1.05)	0.70(0.27,1.13)	-0.38(-1.53,0.79)	1.70(1.51,1.88)
85-89 years	291,466(246,291-324,052)	610,310(515,938-730,018)	2.32(1.96,2.68)	2.87(2.61,3.13)	0.36(-0.55,1.27)	3.88(3.44,4.31)
90-94 years	94,642(79,928-105,681)	327,455(261,472-398,729)	4.03(3.82,4.23)	5.33(4.90,5.76)	2.53(2.26,2.81)	4.60(4.45,4.75)
95+ years	17,652(13,522-20,291)	103,605(77,107-130,564)	6.03(5.33,6.74)	4.82(4.40,5.25)	4.82(2.85,6.82)	8.54(7.87,9.23)
**Injuries**						
65-69 years	1,161,318(997,043-1,317,759)	2,216,301(1,869,099-2,655,425)	2.19(1.82,2.57)	1.92(1.50,2.34)	0.63(-0.30,1.58)	4.09(3.68,4.49)
70-74 years	884,505(767,573-1,002,537)	1,752,875(1,467,467-2,104,288)	2.32(1.99,2.66)	2.92(2.58,3.27)	0.68(0.20,1.15)	3.80(3.09,4.51)
75-79 years	581,617(504,533-655,254)	1,258,953(1,036,000-1,503,698)	2.48(2.22,2.73)	3.62(3.19,4.06)	2.29(2.05,2.54)	1.42(1.10,1.74)
80-84 years	315,236(278,227-356,264)	962,219(779,988-1,142,090)	3.83(3.58,4.07)	4.68(4.21,5.15)	3.59(3.37,3.81)	3.13(2.87,3.38)
85-89 years	133,866(117,155-152,952)	683,158(540,820-809,297)	5.36(4.82,5.90)	5.82(4.71,6.94)	4.00(3.32,4.69)	6.25(5.71,6.79)
90-94 years	31,094(27,132-35,589)	284,679(212,808-341,916)	7.55(7.26,7.84)	8.12(7.72,8.52)	6.55(5.90,7.21)	7.93(7.60,8.26)
95+ years	4,431(3,742-5,117)	77,864(54,864-97,932)	9.77(8.82,10.72)	7.30(4.85,9.80)	9.42(8.94,9.89)	13.07(11.60,14.56)
**Non-communicable diseases**						
65-69 years	23,533,097(20,912,043-26,092,054)	41,888,118(35,790,581-48,217,651)	1.93(1.59,2.27)	1.40(1.25,1.55)	0.19(-0.75,1.14)	4.43(4.16,4.70)
70-74 years	22,640,973(20,410,115-24,800,146)	40,847,248(35,361,014-46,228,499)	1.93(1.78,2.08)	2.00(1.76,2.23)	0.49(0.30,0.68)	3.66(3.42,3.89)
75-79 years	17,146,113(15,627,721-18,686,240)	33,010,289(28,853,538-37,222,797)	2.12(1.76,2.48)	2.50(2.34,2.65)	2.50(2.34,2.65)	1.20(0.03,2.38)
80-84 years	10,082,222(9,366,644-10,877,385)	27,018,590(24,105,943-29,892,408)	3.30(2.89,3.72)	3.69(3.51,3.86)	3.46(2.22,4.72)	2.73(2.55,2.91)
85-89 years	4,383,898(4,106,716-4,657,527)	18,375,080(16,695,278-20,151,821)	4.58(4.01,5.17)	5.17(4.46,5.88)	3.66(2.14,5.20)	4.73(4.52,4.94)
90-94 years	1,057,847(998,578-1,122,606)	7,346,154(6,659,958-8,080,206)	6.48(6.02,6.94)	7.69(7.12,8.25)	5.96(4.74,7.19)	5.96(5.74,6.19)
95+ years	156,497(148,263-164,449)	1,878,841(1,693,325-2,107,570)	8.69(7.83,9.55)	7.58(6.99,8.18)	8.42(5.98,10.92)	10.06(9.44,10.68)
**Other COVID-19 pandemic related outcomes**						
65-69 years	NA	42,625(4,945-197,044)	NA	NA	NA	NA
70-74 years	NA	37,097(5,273-165,366)	NA	NA	NA	NA
75-79 years	NA	26,716(4,360-117,456)	NA	NA	NA	NA
80-84 years	NA	21,040(4,447-86,535)	NA	NA	NA	NA
85-89 years	NA	13,564(3,237-51,105)	NA	NA	NA	NA
90-94 years	NA	7,264(1,865-22,753)	NA	NA	NA	NA
95+ years	NA	2,447(428-7,657)	NA	NA	NA	NA

UI, Uncertainty Interval; CI, Confidence Interval.

**Figure 2:**
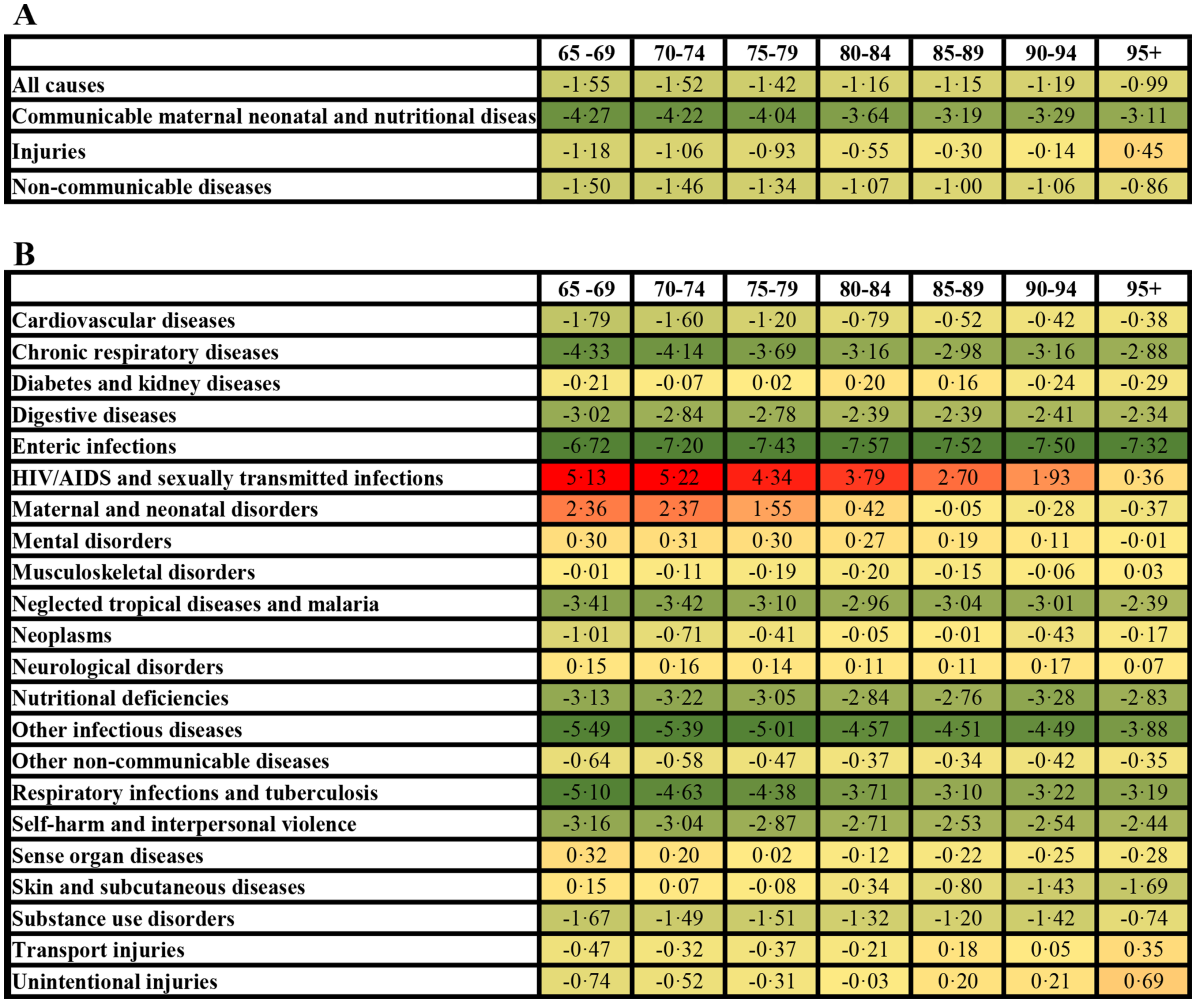
Average annual percentage change in age specific rate of DALYs among older adults in China (1990–2021). All-cause and level 1 causes **(A)**, level 2 causes **(B)**.

The ASR of DALYs declined consistently across age groups from 65–69 years to 95 + years, both for all causes and level 1 causes. However, the ASR of injury increase markedly among those aged 95 + years, in contrast to a decline observed in the population aged 65–94 years (Figure 2A, Table 1). Analysis of level 2 causes revealed divergent trajectories: HIV/AIDS and sexually transmitted infections exhibited the largest increase in the ASR of DALYs, peaking in the 70–74 years age group and maintaining persistently high levels through the 80–84 years age group, then it gradually declining Meanwhile, the ASR of DALYs rate for injuries increased with advancing age. This trend was particularly driven by transport injuries, which accelerated from the 85 + years, and unintentional injuries, which increase most steeply among those aged 95 + years (Figure 2B). Communicable disease exhibited divergent trends: enteric infections and other infectious diseases, alongside respiratory infections and tuberculosis, chronic respiratory disease demonstrated the steepest declines (ASR of AAPC = 3.19–7.57), particularly in those aged 65–84 years (Figure 2B; Supplementary Tables 1–3).

**Figure 3:**
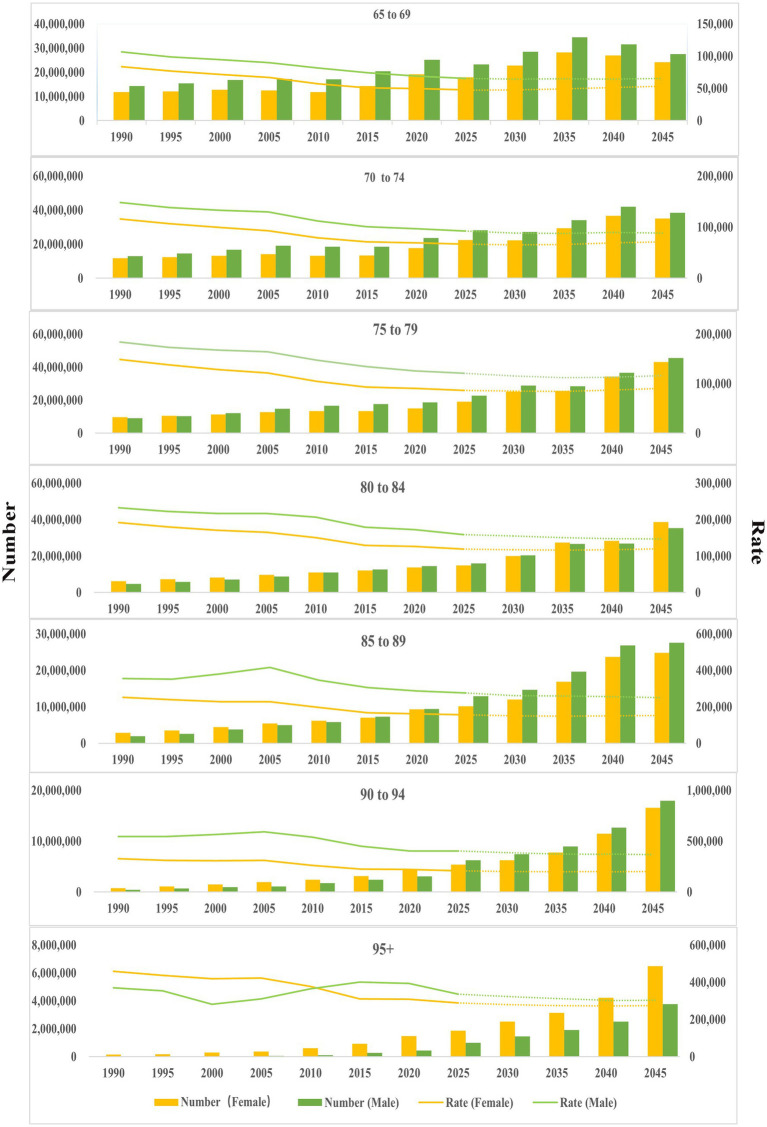
Age-sex distribution of DALYs (1990–2021), and projection from 2022 to 2045. The dotted line represents the projection from 2022 to 2045.

The number and ASR of DALYs across all diseases for seven age groups were projected from 2022 to 2045 (Figure 3). Specifically, there was an increasing trend in the number of DALYs within 75–79 years, 80–84 years, 85–89 years, 90–94 years, 95 + years. However, the ASR of DALYs exhibited contrasting trends by gender: a decreasing trend among males and an increasing trend among females. For the population aged 80–95 + years, the number of DALYs continued to increase, while the ASR showed a decreasing trend (Figure 3, Supplementary Tables 4–10, Supplementary Figures 2–5).

### Temporal trend of disease burden attributable to risk factors

The attribution pattern of level 1 risk factors revealed distinct age- and disease-specific profiles (Supplementary Table 11). For communicable, maternal, neonatal, and nutritional diseases, behavioral risks dominated across all age groups. Disease burden of injury in 65–79 years group primarily attributed to metabolic risks, shifting to environmental/occupational risks in the 85 + years group. The contribution of risk factors to NCDs is mainly attributable to behavioral, environmental/occupational, and metabolic risks from 65–69 years group to 95 + year group.

The ranking according to the percentage of DALYs for level 2 causes attributable to level 2 risk factors remained largely unchanged from 1990 to 2021. Notably, a shift occurred for musculoskeletal disorders, where high body mass index (BMI, from 4.29% in 1990 to 9.01% in 2021) superseded occupational risks (from 9.76% in 1990 to 5.33% in 2021) as the primary contributor (Supplementary Table 12).

**Figure 4:**
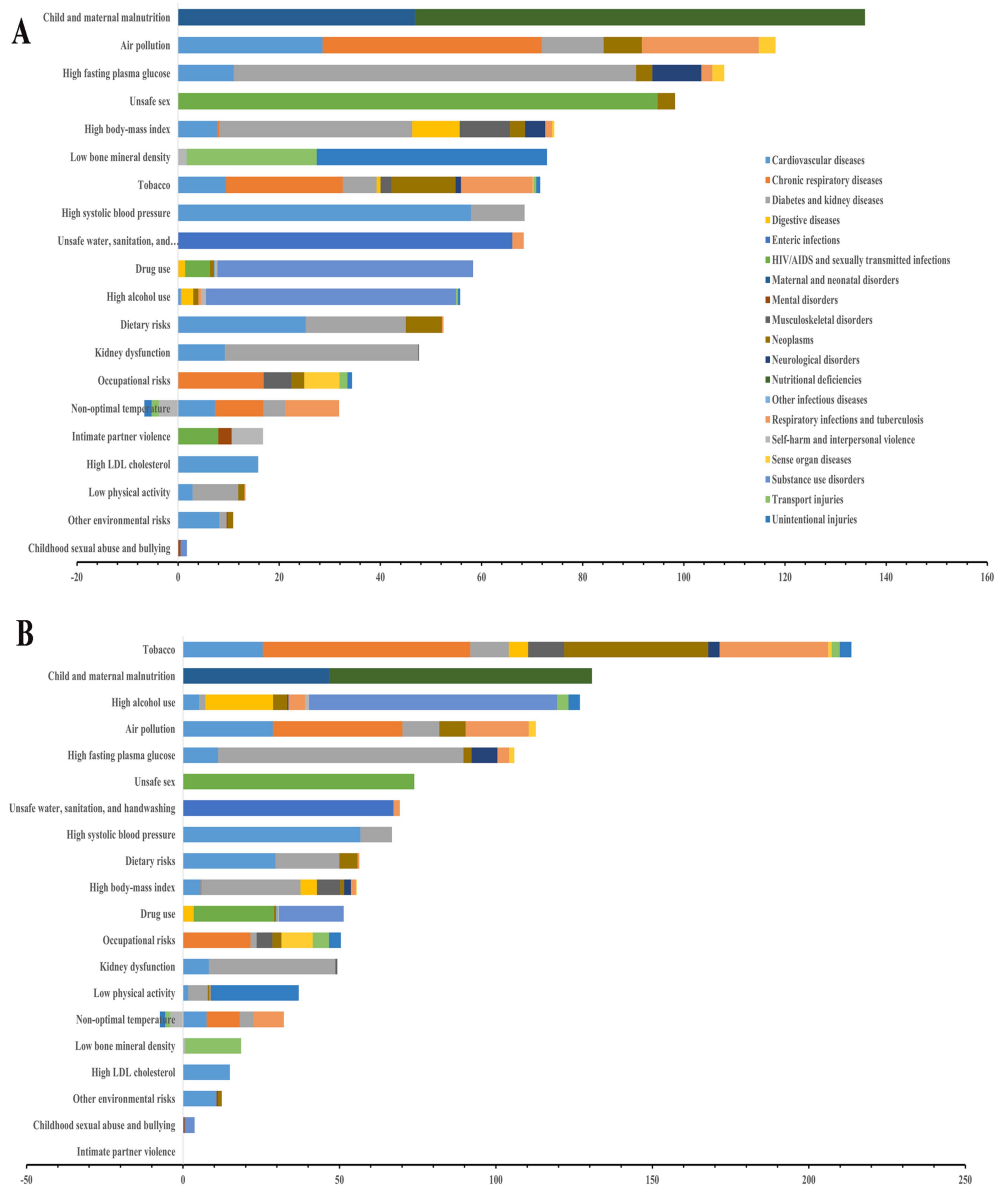
The contribution of level 2 risk factors to level 2 cause in China, 2021. **(A)** Female; **(B)** Male.

Stratified by gender, the analysis identified the top 20 risk factors contributing to the percentage of DALYs from level 2 causes attributable to level 2 risk factors (Figure 4, Supplementary Table 13). For females, the top five risk factors were child and maternal malnutrition, air pollution, high fasting plasma glucose, unsafe sex, and high BMI. Among males, the leading five risk factors comprised tobacco use, child and maternal malnutrition, high alcohol use, air pollution, and high fasting plasma glucose. When it comes to level 3 risk factors, particulate matter pollution and smoking disproportionately affect cardiovascular diseases, chronic respiratory diseases, diabetes/kidney diseases, neoplasms, and tuberculosis-related respiratory infections (Supplementary Table 14).

Longitudinal analysis identified that particulate matter pollution, smoking, and high-sodium diets consistently ranked as the top three risk factors (level 3 risk factors) from 1990 to 2021, despite a declining proportional contribution over time. Concurrently, the ranking of diet low in whole grains significantly increase from 11st in 1990 to 7th in 2021, and the ranking of diet high in red meat significantly increase from 32nd in 1990 to 14th in 2021, underscoring the growing challenge of nutritionally imbalanced diets (Supplementary Figure 6, Supplementary Table 15).

### Decomposition analysis

Our decomposition analysis identified population growth as the dominant driver of increasing disease burden in the older adults population, surpassing the contribution from population aging (Supplementary Tables 16–18). From 1990 to 2021, DALYs attributed to all causes and level 2 causes, particularly those related to ageing, have shown a consistent upward trend (Supplementary Figure 7). Notably, NCDs exhibited the most pronounced increase in burden attributable to ageing population (Supplementary Table 19). Further decomposition of level 3 causes revealed that COPD, stroke, and IHD contributed most significantly to aging-attributable DALY growth (Supplementary Figure 7; Supplementary Table 20).

## Discussion

Our analysis reveals that COPD, stroke, and IHD currently exert the most substantial impact on China’s older adults population. Pulmonary function testing (PFT), the diagnostic gold standard for COPD, is critically underutilized, evidenced by a 2019–2020 national rate of only 6.7% among adults ≥40 years (11) and a mere 12.0% reporting prior testing among undiagnosed symptomatic older adults (PMID: 29650248) (12). China’s substantial burden of undiagnosed COPD—largely undetected by conventional surveillance systems—underscores a critical gap in the current prevention framework. Notably, pulmonologist-led management has been shown to reduce subsequent respiratory healthcare utilization by 22% among symptomatic, undiagnosed adults compared to routine care (13). Effective reduction of the COPD burden in China necessitates prioritizing the optimization of early detection pathways for high-risk, undiagnosed individuals. Stroke care in China reveals substantial systemic shortcomings: hypertension control rates in rural areas are only half those in urban centers; (14)screening coverage for high-risk populations remains inadequate; emergency care networks suffer from significant coverage gaps; and rehabilitation service access in central and western provinces remains below 60% (15, 16). Reducing the stroke burden in China necessitates a multi-faceted strategy encompassing strengthened risk factor prevention, improved access to timely acute stroke care, prioritization of secondary prevention, and expansion of rehabilitation services (17, 18). Effective control of IHD in China requires an evidence-based, multidimensional strategy—encompassing targeted risk factor screening, coordinated acute coronary care networks, and integrated rehabilitation pathways—supported by AI-enhanced electrocardiogram (AI-ECG) technologies and the optimized implementation of the tiered healthcare system (19). (see Table 1)

Our data highlights particulate matter pollution, smoking, and high sodium intake as the most common risk factors in the older adults. First of all, our previous research has shown a strong association between PM2.5 exposure and COPD, reduced lung function (20, 21), prediabetes and diabetes (22), Alzheimer’s disease and other dementias (23), and hypertension risk (24). More importantly, long-term exposure to ambient PM2.5 is linked to higher risks of all-cause mortality and deaths from cancer, natural causes, CVD, and influenza/pneumonia (25). This demonstrates the significant long-term health burden associated with cumulative air pollution exposure. In other words, modest reductions in air pollution can significantly improve health and equity, even in low-exposure environments (26). This compelling evidence justifies sustained enhancement of air quality regulations, stricter standards with robust enforcement, and targeted protection for older adults populations facing high cumulative exposure risks. In addition, the high prevalence of smoking significantly reduces both life expectancy and disease-free years (27). Despite declining smoking rates, a Burden of Proof study links secondhand smoke to increased risks of ischemic heart disease, stroke, type 2 diabetes, and lung cancer, with weaker associations for otitis media, asthma, respiratory infections, breast cancer, and COPD (28). Strengthening smoking cessation support—especially among older adults smokers—remains critical. Concurrently, expanding prevention efforts into public spaces is essential to limit involuntary secondhand smoke exposure. Furthermore, the DASH-Sodium Collaborative Research Group found that reducing sodium intake below the recommended 100 mmol per day significantly lowers blood pressure. Notably, high dietary salt intake may contribute to autoimmune disease development by promoting pathogenic TH17 cell activity. This compels salt reduction reformulation in the food industry, intensified public education on low-sodium diets, and personalized dietary guidance implementation.

Our data found an increased disease burden of sexually transmitted infections among the older adults, particularly HIV/AIDS. As the HIV-positive population ages, the patient demographic is shifting, with a growing number of older individuals experiencing multiple comorbidities (29). This suggests that older adults may be at increased risk due to factors such as lower awareness, limited preventive measures, biological susceptibility, or changes in sexual behavior. Furthermore, older adults may experience profound social isolation, making them less likely to form long-term stable partnerships and leading to increased sexual activity (30). Animal therapy and technology in long-term care may help to reduce loneliness and social isolation in older adults (31). The development of a potent and durable HIV-1 vaccine is challenging due to extraordinary genetic diversity of HIV-1 and its complex mechanisms of immune evasion, but germline-targeting vaccine design, especially mRNA-LNP platform, showed promise to induce broadly neutralizing antibodies to HIV in several phase 1 human clinical trials (32–34). It is necessary to strengthen targeted health education for the older adults, provide accessible screening services, implement risk behavior intervention, such as promoting the implementation of PrEP, and integrate the chronic disease comprehensive management model simultaneously (35, 36).

The emergence of COVID-19 had a profound impact on China’s older adults population. First, 6 months after acute infection, COVID-19 survivors commonly experienced fatigue or muscle weakness, sleep difficulties, and anxiety or depression (37). Second, vaccine hesitancy likely played a significant role in the heightened mortality among vulnerable populations following the end of China’s Zero-COVID policy (38). Up to 18% of unvaccinated individuals experienced post-COVID-19 conditions up to 2 years after infection, with a higher risk of symptoms compared to controls (39). Third, the COVID-19 pandemic placed immense pressure on the healthcare system, leading to delays in essential medical services, lower hospital admission rates, and increased mortality (40). Finally, the incidence of post-acute sequelae of SARS-CoV-2 (PASC) declined over the pandemic’s course but remained significant, even among vaccinated individuals infected during the Omicron era (41).

Essentially, the projections outline the coming disease burden landscape and serve as a strategic action blueprint. Critically, they evidence that mitigating aging-driven health threats requires highly targeted public health interventions and healthcare reforms, customized through rigorous age stratification and gender analysis.

Despite our comprehensive analysis of aging in China and the evolving disease burden, several limitations remain. First, analysis of granular subpopulations, such as subnational areas or specific demographic groups, was limited by data availability. Second, a key limitation is the inconsistent availability and quality of data for estimating relative risks and exposures, with disparities across socioeconomic factors, especially in conflict-affected areas (10). Third, the inherent limitations of model-based analysis affect the precision of our findings. Fourth, modeling COVID-19, long COVID, and related outcomes in GBD 2021 posed significant analytical challenges due to the complexities of data collection and the novelty of the disease (9).

## Conclusion

To address burden of ageing spectrum of diseases, China should leverage complementary roles of public and private insurance to manage cardiovascular and pulmonary disease, promote healthy environments and diets and prioritize prevention of HIV/AIDS.

## Data Availability

The original contributions presented in the study are included in the article/Supplementary material, further inquiries can be directed to the corresponding authors.

## Footnotes

^1^ http://ghdx.healthdata.org/gbd-results-tool

^2^ https://vizhub.healthdata.org/gbd-compare/
