# Supplementary material for: Burden of ageing spectrum of diseases in China, 1990–2021: a systematic analysis of global burden of disease study 2021
Source: Front Public Health. 2025 Jul 29;13:1611901. doi: 10.3389/fpubh.2025.1611901 (PMC12340997; doi:10.3389/fpubh.2025.1611901)
Supplement: Supplementary file 1 [file Supplementary_file_1.pdf]

Supplementary Materials

Burden of ageing spectrum of diseases in China, 1990–2021: a systematic analysis of global burden of disease study 2021

Supplementary figures

Supplementary Figure 1. Population distribution of different age groups in China.

Total number and percentage of different age groups based on 0-14 years, 15-64 years, 65+years aged population in China from 1990 to 2021 (A).

Population age structure pyramid of China in 1990 (B).

Population age structure pyramid of China in 2000 (C).

Population age structure pyramid of China in 2010 (D).

Population age structure pyramid of China in 2021 (E).

Supplementary Figure 2. Number and age-specific rate of disability-adjusted life year among 65+ years in China of all cause diseases, 1990-2021.

Supplementary Figure 3. Number and age-specific rate of disability-adjusted life year among 65+ years in China of communicable, maternal, neonatal, and nutritional diseases, 1990-2021.

Supplementary Figure 4. Number and age-specific rate of disability-adjusted life year among 65+ years in China of injuries, 1990-2021.

Supplementary Figure 5. Number and age-specific rate of disability-adjusted life year among 65+ years in China of non-communicable diseases, 1990-2021.

Supplementary Figure 6. Leading 30 level 3 risks by attributable to all cause disability-adjusted life year, 1990 and 2021 among elderly population.

Causes are connected by lines between time periods, where solid lines represent an increase or no change in rank and dashed lines represent a decrease in rank.

Supplementary Figure 7. Change in disability-adjusted life year associated with population ageing in China.

Changes in disability-adjusted life year for level 1 causes driven by population ageing, 1990-2021(A).

Top 15 diseases with the disability-adjusted life year change associated with population ageing between 1990 and 2021 in China (B).

Supplementary tables

Supplementary Table 1. The age specific rate and number of disability-adjusted life year for level 2 causes in 1990 and 2021 and average annual percent change between 1990 and 2021, by age group.

Supplementary Table 2. The age specific rate of disability-adjusted life year for level 3 causes in 1990 and 2021 and average annual percent change between 1990 and 2021, by age group.

Supplementary Table 3. The number of disability-adjusted life for level 3 causes in 1990 and 2021 and average annual percent change between 1990 and 2021, for both sexes, by age group.

Supplementary Table 4. Projection of number and rate of disease-adjusted life years aged 65-69 years attributable to all cause from 2022 to 2045.

Supplementary Table 5. Projection of number and rate of disease-adjusted life years aged 70-74 years attributable to all cause from 2022 to 2045.

Supplementary Table 6. Projection of number and rate of disease-adjusted life years aged 75-79 years attributable to all cause from 2022 to 2045.

Supplementary Table 7. Projection of number and rate of disease-adjusted life years aged 80-84 years attributable to all cause from 2022 to 2045.

Supplementary Table 8. Projection of number and rate of disease-adjusted life years aged 85-89 years attributable to all cause from 2022 to 2045.

Supplementary Table 9. Projection of number and rate of disease-adjusted life years aged 90-94 years attributable to all cause from 2022 to 2045.

Supplementary Table 10. Projection of number and rate of disease-adjusted life years aged 95+ years attributable to all cause from 2022 to 2045.

Supplementary Table 11. Percentage of disability-adjusted life attributable to level 1 risk factors, by level 1 causes and age group, 2021, both sexes, China.

Supplementary Table 12. Percentage of disability-adjusted life attributable to level 2 risk factors, by level 2 causes, 1990 and 2021, 65+ years, both sexes, China.

Supplementary Table 13. Percentage of disability-adjusted life attributable to level 2 risk factors, by level 2 causes and sex, 2021, 65+ years, China.

Supplementary Table 14. Percentage of disability-adjusted life attributable to level 3 risk factors, by level 2 causes, 1990 and 2021, 65+ years, both sexes, China.

Supplementary Table 15. Percentage of disability-adjusted life attributable to level 3 risk factors, by all cause, 1990 and 2021, 65+ years, both sexes, China.

Supplementary Table 16. The contribution of ageing, population and epidemiological change to disability-adjusted life for all cause and level 1 causes in China.

Supplementary Table 17. The contribution of ageing, population and epidemiological change to disability-adjusted life for level 2 causes in China.

Supplementary Table 18. The contribution of ageing, population and epidemiological change to disability-adjusted life for level 3 causes in China.

Supplementary Table 19. Change in disability-adjusted life associated with population ageing in China, 1990-2021.

Supplementary Table 20. Top 15 diseases with the disability-adjusted life change associated with population ageing between 1990 and 2021 in China.

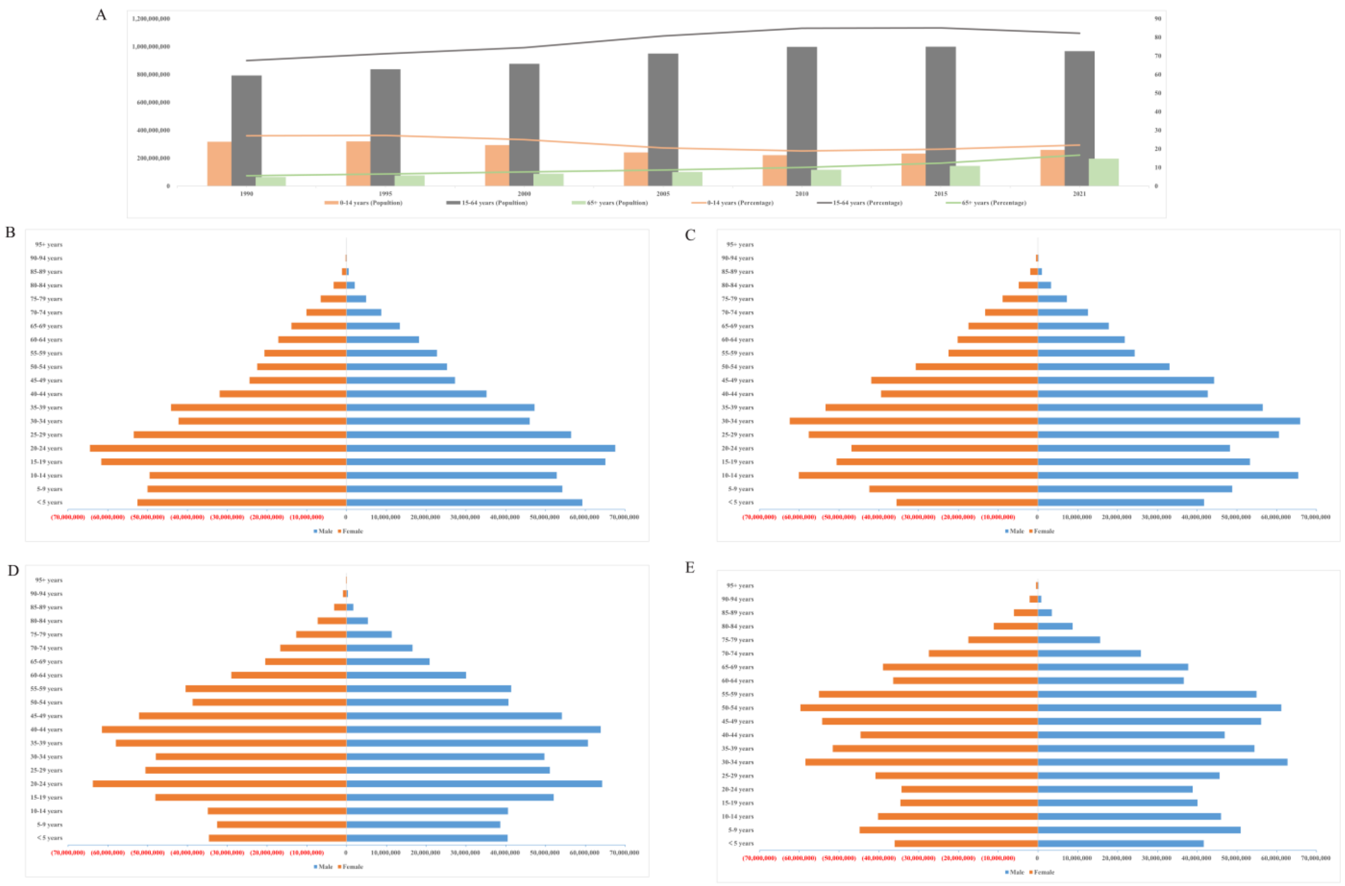

Supplementary Figure 1. Population distribution of different age groups in China.

Total number and percentage of different age groups based on 0-14 years, 15-64 years, 65+years aged population in China from 1990 to 2021 (A). Population age structure pyramid of China in 1990 (B). Population age structure pyramid of China in 2000 (C). Population age structure pyramid of China in 2010 (D). Population age structure pyramid of China in 2021 (E).

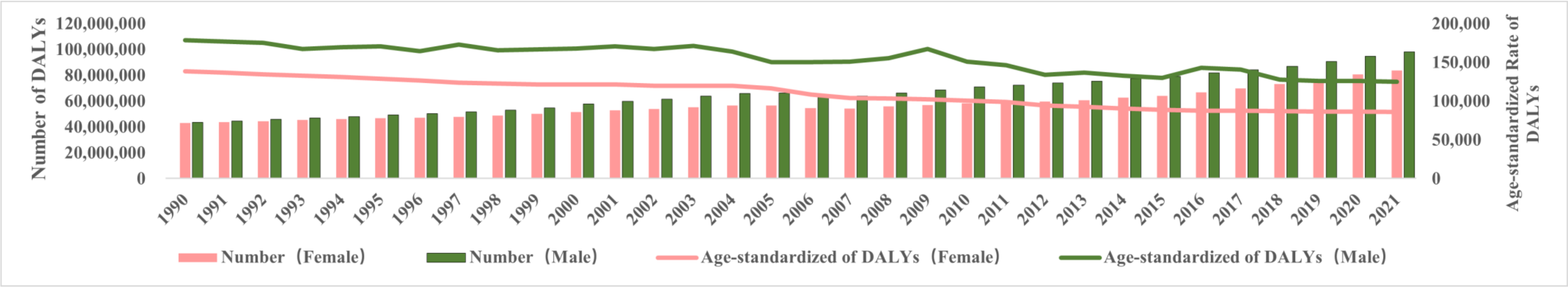

Supplementary Figure 2. Number and age-specific rate of disability-adjusted life year among 65+ years in China of all cause diseases, 1990-2021.

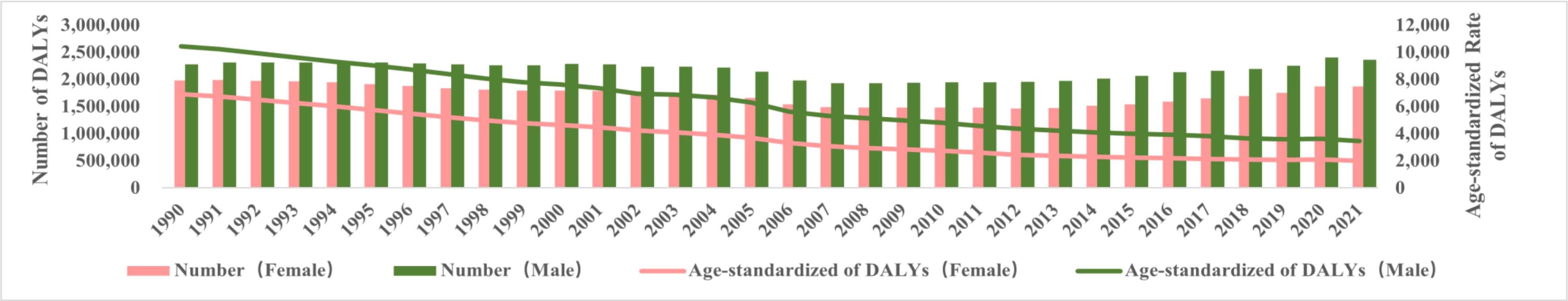

Supplementary Figure 3. Number and age-specific rate of disability-adjusted life year among 65+ years in China of communicable, maternal, neonatal, and nutritional diseases, 1990-2021.

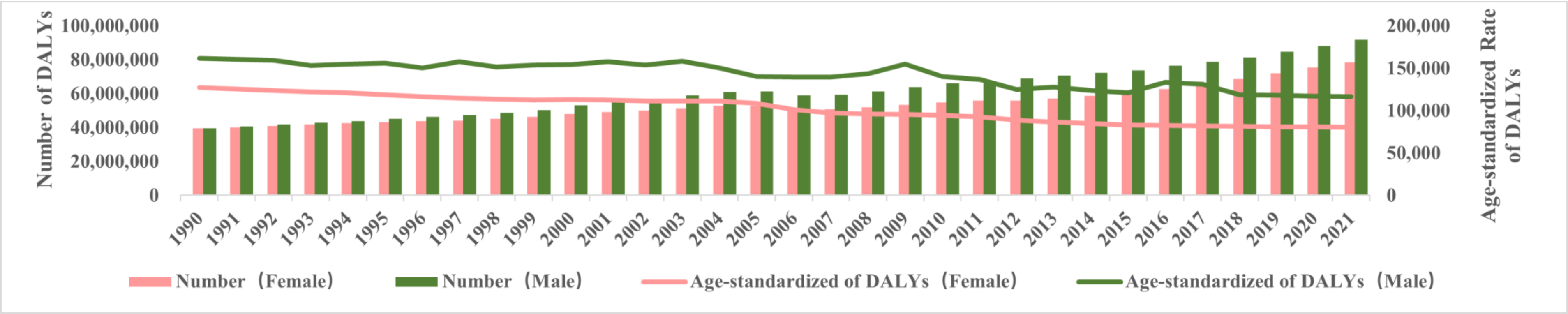

Supplementary Figure 4. Number and age-specific rate of disability-adjusted life year among 65+ years in China of injuries, 1990-2021.

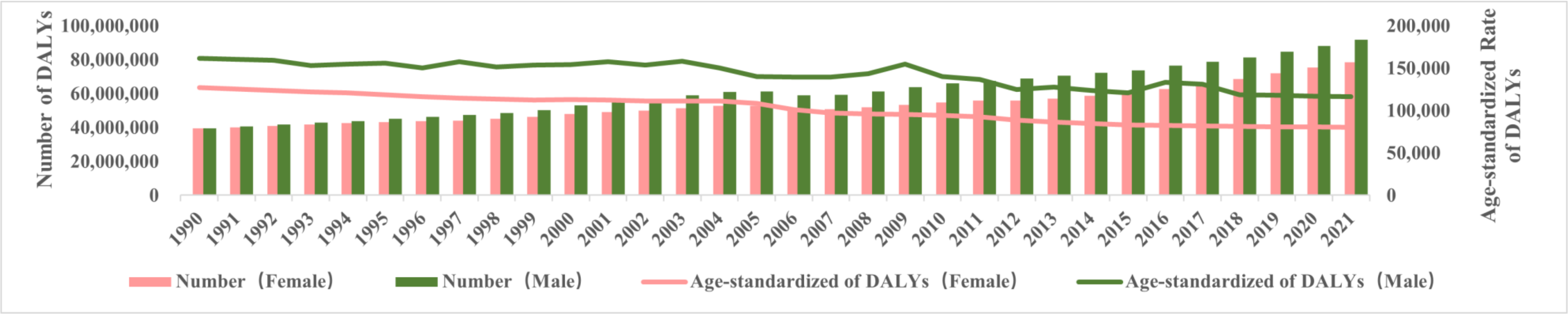

Supplementary Figure 5. Number and age-specific rate of disability-adjusted life year among 65+ years in China of non-communicable diseases, 1990-2021.

leading risks 1990	Percentage of DALYs
Particulate matter pollution	30.49(26.27,34.83)
Smoking	20.38(16.43,24.61)
Diet high in sodium	7.34(2.39,13.58)
Low temperature	5.18(4.52,5.84)
Secondhand smoke	4.29(2.15,6.63)
Occupational particulate matter, gases, and fumes	4.15(2.62,5.71)
Diet low in fruits	3.83(1.93,5.77)
Lead exposure	3.40(-0.33,7.09)
Diet low in vegetables	2.36(1.31,3.47)
Ambient ozone pollution	1.88(0.40,3.30)
Diet low in whole grains	1.39(0.37,2.43)
Diet low in fiber	0.81(0.10,1.75)
Diet low in nuts and seeds	0.66(0.18,1.16)
Diet low in polyunsaturated fatty acids	0.62(-1.69,2.41)
Diet low in seafood omega-3 fatty acids	0.62(0.12,1.12)
Occupational ergonomic factors	0.34(0.19,0.53)
Occupational injuries	0.32(0.25,0.41)
Iron deficiency	0.30(0.21,0.43)
Occupational carcinogens	0.29(0.22,0.37)
High temperature	0.28(0.08,0.60)
Occupational noise	0.26(0.16,0.40)
Diet low in legumes	0.25(-0.19,0.69)
Diet low in milk	0.23(0.08,0.36)
Diet low in calcium	0.20(0.14,0.26)
No access to handwashing facility	0.19(-0.09,0.54)
Child growth failure	0.14(0.12,0.16)
Unsafe water source	0.13(0.05,0.22)
Residential radon	0.12(-0.06,0.32)
Unsafe sanitation	0.12(0.06,0.18)
Diet high in processed meat	0.07(0.01,0.14)
Diet high in sugar-sweetened beverages	0.01(0.00,0.02)
Diet high in red meat	-0.11(-1.31,0.84)

leading risks 2021	Percentage of DALYs
Particulate matter pollution	17.15(13.39,21.86)
Smoking	16.90(13.11,21.41)
Diet high in sodium	6.33(1.62,12.55)
Low temperature	3.85(3.18,4.54)
Lead exposure	3.62(-0.43,7.55)
Secondhand smoke	3.46(1.71,5.46)
Diet low in whole grains	2.26(0.74,3.96)
Occupational particulate matter, gases, and fumes	2.08(1.28,2.96)
Diet low in fruits	2.08(0.97,3.37)
Diet low in polyunsaturated fatty acids	1.03(-2.62,4.06)
Ambient ozone pollution	0.89(0.18,1.62)
Diet low in nuts and seeds	0.71(0.17,1.39)
Occupational carcinogens	0.54(0.38,0.75)
Diet high in red meat	0.47(-0.37,1.05)
Diet low in seafood omega-3 fatty acids	0.45(0.08,0.95)
Diet low in fiber	0.44(0.08,1.13)
Occupational noise	0.42(0.27,0.62)
Diet low in legumes	0.35(-0.25,0.96)
High temperature	0.32(0.11,0.65)
Diet low in milk	0.32(0.11,0.51)
Diet low in vegetables	0.28(0.05,0.70)
Occupational ergonomic factors	0.25(0.14,0.42)
Residential radon	0.20(-0.10,0.56)
Diet high in processed meat	0.20(0.03,0.38)
Iron deficiency	0.19(0.12,0.28)
Diet low in calcium	0.13(0.09,0.18)
Occupational injuries	0.09(0.07,0.13)
Child growth failure	0.07(0.06,0.08)
Diet high in sugar-sweetened beverages	0.07(0.03,0.13)
No access to handwashing facility	0.04(-0.02,0.11)
Unsafe water source	0.02(0.01,0.03)
Unsafe sanitation	0.01(0.01,0.02)

Behavioral risks
Environmental/occupational risks
Metabolic risks

Supplementary Figure 6. Leading 30 level 3 risks by attributable to all cause disability-adjusted life, 1990 and 2021 among elderly population.

Causes are connected by lines between time periods, where solid lines represent an increase or no change in ranking and dashed lines represent a decrease in ranking.

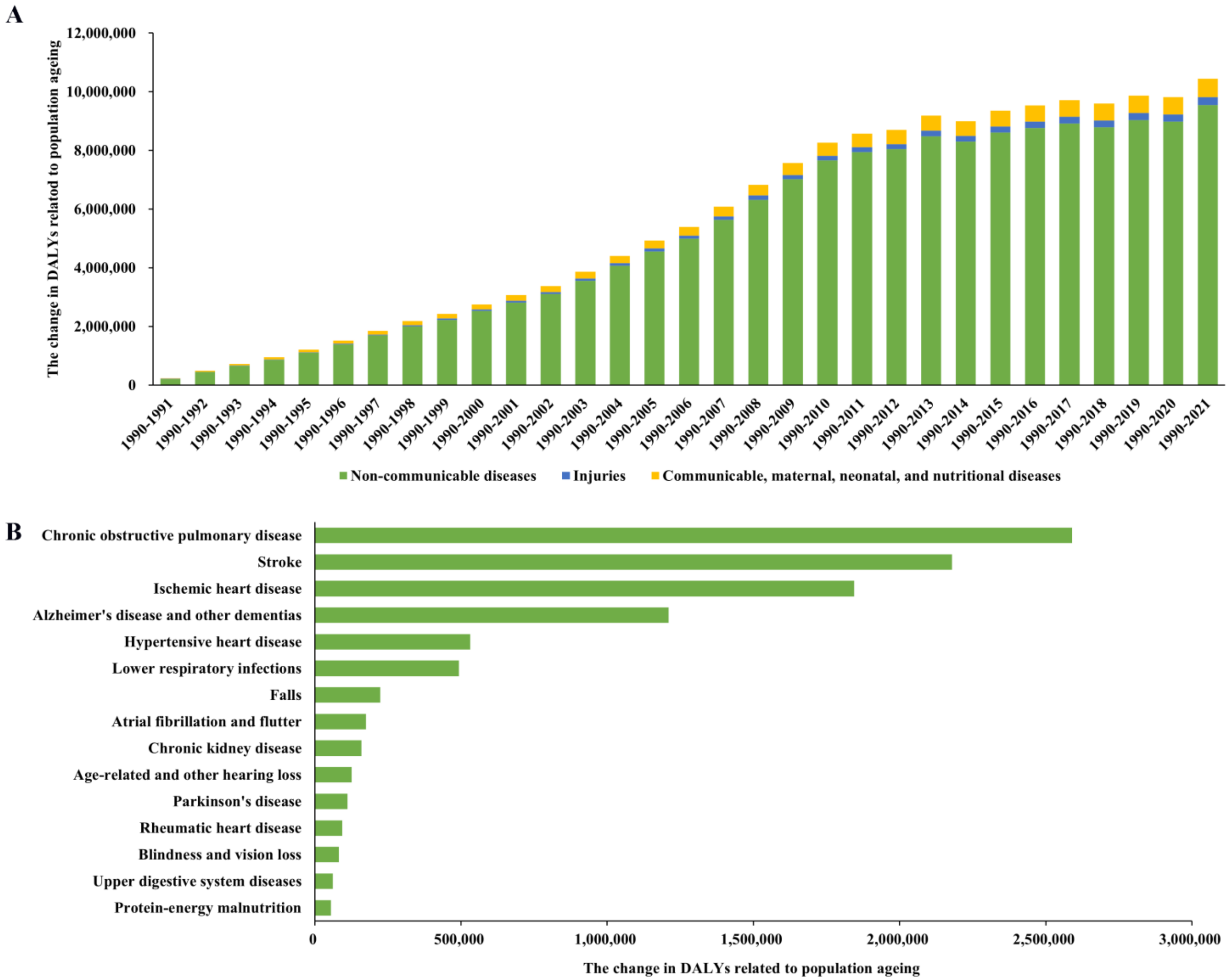

Supplementary Figure 7. Change in disability-adjusted life associated with population ageing in China.

Changes in disability-adjusted life for level 1 causes driven by population ageing, 1990-2021(A).

Top 15 diseases with the disability-adjusted life change associated with population ageing between 1990 and 2021 in China (B).

Supplementary Table 1. The age specific rate and number of disability-adjusted life for level 2 causes in 1990 and 2021 and average annual percent change between 1990 and 2021, by age group.

	1990	2021	1990-2021	1990-2000	2001-2011	2012-2021
Age group	**N(95%UI)**	**N(95%UI)**	**AAPC (95%CI)**	**APC (95%CI)**	**APC (95%CI)**	**APC (95%CI)**
			**Age specific rate**			
**Cardiovascular diseases**						
65-69 years	30,379.52(26,818.50-34,344.00)	17,225.45(14,478.36-20,220.56)	-1.79(-1.92, -1.68)	-1.42(-1.73, -1.14)	-2.3(-2.49, -2.11)	-1.88(-2.13, -1.59)
70-74 years	45,172.32(40,123.07-50,006.40)	28,072.17(23,723.80-32,623.99)	-1.6(-1.65, -1.55)	-1.13(-1.25, -1)	-1.91(-1.99, -1.84)	-2(-2.11, -1.88)
75-79 years	59,814.52(53,408.85-66,399.63)	40,370.39(34,135.38-46,775.32)	-1.2(-1.31, -1.09)	-0.97(-1.24, -0.71)	-1.38(-1.56, -1.22)	-1.49(-1.72, -1.13)
80-84 years	78,801.74(69,515.75-86,634.97)	61,543.84(52,602.12-70,672.09)	-0.79(-0.96, -0.66)	-0.58(-1.05, -0.19)	-0.6(-0.9, -0.24)	-1.39(-1.74, -1.16)
85-89 years	108,773.05(95,836.57-119,756.61)	93,526.46(79,261.06-106,124.73)	-0.52(-0.64, -0.38)	0.47(0.11, 0.81)	-0.82(-1.08, -0.62)	-1.61(-1.9, -1.17)
90-94 years	147,187.67(128,730.21-163,242.68)	128,096.14(104,820.53-147,518.03)	-0.42(-0.56, -0.31)	0.64(0.23, 0.97)	-0.44(-0.66, -0.08)	-1.76(-2.02, -1.55)
95+ years	169,868.99(137,132.11-192,368.05)	151,060.40(115,242.10-180,364.42)	-0.38(-0.51, -0.27)	-0.67(-1.06, -0.16)	0.17(-0.13, 0.4)	-1.26(-1.51, -1.06)
**Chronic respiratory diseases**						
65-69 years	14,705.56(12,686.46-16,576.34)	3,897.63(3,236.84-4,656.56)	-4.33(-4.56, -4.19)	-3.65(-3.86, -3.41)	-5.67(-5.85, -5.54)	-3.62(-4.42, -3.17)
70-74 years	27,361.05(23,590.48-30,656.84)	7,801.99(6,449.15-9,402.45)	-4.14(-4.26, -4.04)	-3.44(-3.57, -3.28)	-5.61(-5.75, -5.5)	-3.26(-3.65, -2.95)
75-79 years	38,616.53(33,538.17-42,893.36)	12,659.55(10,654.44-15,021.39)	-3.69(-3.88, -3.56)	-3.11(-3.3, -2.87)	-5.09(-5.31, -4.96)	-2.79(-3.42, -2.37)
80-84 years	53,692.30(45,497.15-59,179.99)	20,232.51(16,769.60-23,320.59)	-3.16(-3.36, -2.98)	-2.09(-2.57, -1.73)	-4.54(-4.83, -4.32)	-2.49(-3.11, -1.94)
85-89 years	79,898.59(68,156.67-88,839.12)	31,657.12(26,377.20-36,526.90)	-2.98(-3.12, -2.86)	-1.3(-1.48, -1.09)	-4.84(-5.02, -4.71)	-2.78(-3.21, -2.39)
90-94 years	108,623.56(90,489.45-122,614.81)	41,453.11(33,445.43-48,530.82)	-3.16(-3.32, -3.04)	-1.83(-2.1, -1.55)	-4.39(-4.6, -4.21)	-3.18(-3.68, -2.82)
95+ years	111,952.33(84,440.90-126,875.29)	45,875.79(34,218.04-56,576.13)	-2.88(-3.01, -2.73)	-2.2(-2.5, -1.83)	-3.66(-3.96, -3.51)	-2.14(-2.58, -1.71)
**Diabetes and kidney diseases**						

65-69 years	3,397.54(2,899.39-3,921.08)	3,202.00(2,650.06-3,845.16)	-0.21(-0.25, -0.17)	0.48(0.36, 0.59)	-0.73(-0.81, -0.65)	-0.55(-0.65, -0.45)
70-74 years	4,146.29(3,564.70-4,779.79)	4,079.49(3,442.61-4,812.58)	-0.07(-0.11, -0.04)	0.67(0.57, 0.76)	-0.47(-0.54, -0.4)	-0.59(-0.69, -0.49)
75-79 years	4,793.88(4,163.98-5,482.60)	4,762.33(4,040.29-5,562.09)	0.02(-0.03, 0.07)	0.55(0.41, 0.68)	-0.17(-0.28, -0.08)	-0.51(-0.61, -0.34)
80-84 years	4,961.70(4,273.96-5,672.57)	5,281.84(4,466.14-6,176.91)	0.2(0.09, 0.29)	0.57(0.32, 0.8)	0.55(0.29, 0.7)	-0.66(-0.98, -0.39)
85-89 years	6,405.40(5,626.19-7,318.57)	6,643.21(5,693.00-7,633.30)	0.16(0.09, 0.24)	0.91(0.69, 1.11)	-0.31(-0.45, -0.17)	-0.34(-0.49, -0.14)
90-94 years	8,647.78(7,465.74-9,929.35)	8,001.15(6,829.49-9,228.50)	-0.24(-0.29, -0.19)	0.44(0.34, 0.56)	-0.47(-0.58, -0.37)	-0.74(-0.94, -0.61)
95+ years	9,833.73(8,279.63-11,427.38)	8,862.92(7,116.45-10,670.91)	-0.29(-0.36, -0.21)	-0.06(-0.21, 0.16)	-0.71(-0.91, -0.59)	-0.12(-0.3, 0.18)
**Digestive diseases**						
65-69 years	3,675.75(3,150.78-4,221.08)	1,481.02(1,218.49-1,777.18)	-3.02(-3.1, -2.95)	-2.74(-2.95, -2.54)	-3.29(-3.44, -3.1)	-3.22(-3.39, -2.96)
70-74 years	4,369.38(3,760.96-4,989.55)	1,828.92(1,512.28-2,186.42)	-2.84(-2.93, -2.77)	-2.76(-2.93, -2.58)	-3.21(-3.37, -3.09)	-2.58(-2.86, -2.35)
75-79 years	5,110.10(4,435.18-5,852.24)	2,221.93(1,869.20-2,618.18)	-2.78(-2.85, -2.71)	-2.83(-3.04, -2.63)	-3(-3.15, -2.83)	-2.72(-2.91, -2.5)
80-84 years	5,668.68(4,866.31-6,534.02)	2,722.38(2,264.63-3,197.85)	-2.39(-2.51, -2.28)	-2.46(-2.73, -2.23)	-2.56(-2.75, -2.32)	-2.2(-2.55, -1.87)
85-89 years	7,395.57(6,244.12-8,647.99)	3,684.38(3,027.92-4,348.53)	-2.39(-2.52, -2.26)	-1.91(-2.28, -1.53)	-2.86(-3.11, -2.56)	-2.88(-3.19, -2.5)
90-94 years	9,503.39(7,896.68-11,296.34)	4,539.85(3,678.00-5,441.53)	-2.41(-2.52, -2.32)	-2.1(-2.36, -1.84)	-2.86(-3.05, -2.66)	-2.43(-2.75, -2.15)
95+ years	10,734.70(8,380.08-13,170.23)	5,180.33(3,881.68-6,420.31)	-2.34(-2.46, -2.23)	-3.17(-3.46, -2.79)	-2.76(-3.03, -2.29)	-1.21(-1.59, -0.85)
**Enteric infections**						
65-69 years	150.75(79.52-219.17)	18.13(12.83-27.04)	-6.72(-6.78, -6.66)	-8.02(-8.13, -7.92)	-7.6(-7.8, -7.48)	-4.18(-4.37, -3.9)
70-74 years	225.74(113.27-335.89)	22.98(16.21-36.34)	-7.2(-7.27, -7.12)	-8.37(-8.54, -8.16)	-8.41(-8.64, -8.29)	-4.44(-4.63, -4.17)
75-79 years	329.80(158.32-493.94)	30.69(20.16-51.14)	-7.43(-7.51, -7.33)	-8.71(-8.89, -8.43)	-8.61(-8.83, -8.5)	-4.57(-4.78, -4.31)
80-84 years	476.38(215.83-717.62)	42.98(27.80-75.20)	-7.57(-7.64, -7.51)	-8.51(-8.72, -8.34)	-9.13(-9.3, -9)	-4.51(-4.71, -4.29)
85-89 years	751.01(342.90-1,140.72)	69.49(44.56-123.38)	-7.52(-7.62, -7.44)	-8.12(-8.29, -7.92)	-9.23(-9.55, -9.09)	-4.88(-5.19, -4.52)
90-94 years	1,025.36(436.49-1,656.55)	95.53(57.51-175.22)	-7.5(-7.61, -7.41)	-8.35(-8.46, -8.25)	-8.35(-8.46, -8.25)	-5.39(-5.72, -5.11)

95+ years	1,184.80(469.91-2,029.25)	115.71(68.48-222.56)	-7.32(-7.4, -7.24)	-9.25(-9.51, -9.02)	-7.49(-7.75, -7.3)	-4.71(-4.99, -4.42)
**HIV/AIDS and sexually transmitted infections**						
65-69 years	22.02(12.42-31.12)	104.96(74.00-138.74)	5.13(4.79, 5.44)	7.37(6.67, 8.22)	0.75(0.21, 1.19)	8.26(7.18, 9.14)
70-74 years	19.57(12.37-27.73)	86.44(60.37-118.95)	5.22(4.96, 5.44)	9.11(8.7, 9.58)	0.4(0.01, 0.75)	7.23(6.35, 7.96)
75-79 years	26.67(20.06-35.38)	77.65(55.29-105.89)	4.34(3.75, 4.82)	6.19(5.59, 6.96)	4.11(3.1, 4.89)	3.3(1.23, 5.27)
80-84 years	17.55(9.78-38.37)	54.62(31.59-90.66)	3.79(2.81, 4.95)	5.43(2.74, 8.99)	-2.08(-3.11, -1.06)	8.28(6.07, 10.05)
85-89 years	17.91(10.54-34.07)	41.12(24.25-67.42)	2.7(1.98, 3.53)	3.8(1.86, 6.13)	-2.18(-3.06, -1.32)	6.58(4.7, 8.16)
90-94 years	19.60(11.96-32.96)	35.49(21.89-57.16)	1.93(1.27, 2.61)	2.37(0.79, 4.2)	-1.85(-2.65, -1.14)	5.38(3.61, 6.92)
95+ years	22.13(13.46-33.16)	29.16(19.33-45.12)	0.36(-0.2, 0.78)	-1.6(-3.04, -0.79)	-0.2(-1.1, 0.71)	3.27(1.5, 4.48)
**Maternal and neonatal disorders**						
65-69 years	39.27(27.31-57.55)	80.54(59.14-102.09)	2.36(2.33, 2.39)	1.9(1.83, 1.98)	2.95(2.91, 3)	2.1(2, 2.17)
70-74 years	22.64(16.05-31.91)	46.58(33.91-59.14)	2.37(2.35, 2.4)	1.32(1.22, 1.4)	3.22(3.13, 3.25)	2.55(2.48, 2.61)
75-79 years	15.16(10.40-22.02)	24.33(17.68-31.47)	1.55(1.52, 1.58)	-0.05(-0.14, 0.07)	2.43(2.36, 2.53)	2.16(2.08, 2.24)
80-84 years	12.69(8.34-18.57)	14.69(10.52-19.96)	0.42(0.36, 0.47)	-1.01(-1.17, -0.85)	1.11(0.95, 1.19)	1.25(1.08, 1.38)
85-89 years	11.69(7.95-17.02)	11.39(7.54-16.36)	-0.05(-0.08, -0.01)	-1.21(-1.31, -1.06)	0.26(0.19, 0.36)	0.84(0.77, 0.91)
90-94 years	11.15(7.38-16.37)	10.24(6.96-14.61)	-0.28(-0.3, -0.23)	-1.38(-1.5, -1.25)	-0.04(-0.1, 0.06)	0.65(0.58, 0.73)
95+ years	10.59(6.99-15.44)	9.39(6.28-13.48)	-0.37(-0.4, -0.32)	-1.35(-1.46, -1.2)	-0.39(-0.46, -0.3)	0.69(0.6, 0.79)
**Mental disorders**						
65-69 years	1,861.48(1,389.08-2,420.40)	2,049.94(1,539.40-2,650.72)	0.3(0.27, 0.33)	0.89(0.8, 0.99)	-0.21(-0.27, -0.17)	0.33(0.25, 0.41)
70-74 years	1,808.89(1,346.29-2,338.71)	1,995.41(1,489.13-2,571.25)	0.31(0.28, 0.34)	0.87(0.79, 0.96)	-0.15(-0.2, -0.11)	0.29(0.22, 0.36)
75-79 years	1,754.41(1,266.31-2,274.36)	1,927.71(1,387.18-2,502.97)	0.3(0.27, 0.33)	0.88(0.81, 0.99)	-0.06(-0.12, -0.02)	0.14(0.05, 0.21)
80-84 years	1,709.62(1,255.72-2,237.37)	1,855.26(1,359.53-2,410.32)	0.27(0.25, 0.3)	0.85(0.75, 0.94)	0(-0.03, 0.03)	0(-0.04, 0.02)

85-89 years	1,681.05(1,233.85-2,198.86)	1,773.30(1,323.30-2,292.37)	0.19(0.17, 0.2)	0.64(0.57, 0.68)	0.06(0, 0.11)	-0.09(-0.14, -0.03)
90-94 years	1,655.24(1,188.54-2,168.07)	1,695.61(1,230.30-2,215.72)	0.11(0.08, 0.15)	0.45(0.39, 0.52)	0.05(-0.06, 0.1)	-0.15(-0.28, -0.03)
95+ years	1,634.65(1,134.59-2,220.43)	1,620.51(1,146.11-2,182.33)	-0.01(-0.04, 0.02)	0.11(0.03, 0.17)	0.13(0.08, 0.18)	-0.23(-0.3, -0.15)
**Musculoskeletal disorders**						
65-69 years	4,371.83(3,003.07-6,150.20)	4,358.85(2,998.70-6,204.10)	-0.01(-0.02, 0.01)	-0.58(-0.62, -0.52)	0.37(0.34, 0.43)	0.1(0.07, 0.12)
70-74 years	4,677.12(3,199.16-6,556.21)	4,522.81(3,092.79-6,350.89)	-0.11(-0.12, -0.09)	-0.83(-0.87, -0.77)	0.34(0.31, 0.41)	0.07(0.05, 0.1)
75-79 years	4,827.23(3,453.11-6,831.47)	4,560.26(3,276.06-6,498.50)	-0.19(-0.21, -0.16)	-0.92(-0.98, -0.81)	0.25(0.2, 0.35)	-0.01(-0.05, 0.03)
80-84 years	4,736.97(3,363.89-6,743.27)	4,468.41(3,142.01-6,540.66)	-0.2(-0.23, -0.18)	-0.77(-0.83, -0.67)	0.24(0.19, 0.32)	-0.18(-0.22, -0.14)
85-89 years	4,569.62(3,211.81-6,580.99)	4,349.41(3,061.49-6,265.67)	-0.15(-0.18, -0.12)	-0.37(-0.47, -0.28)	0.26(0.2, 0.35)	-0.45(-0.51, -0.38)
90-94 years	4,187.95(2,894.50-5,832.03)	4,119.52(2,825.38-5,815.67)	-0.06(-0.09, -0.04)	-0.2(-0.27, -0.1)	0.51(0.44, 0.63)	-0.64(-0.73, -0.58)
95+ years	3,922.22(2,642.21-5,591.38)	3,959.66(2,738.28-5,703.05)	0.03(0.01, 0.05)	-0.09(-0.15, -0.03)	0.69(0.64, 0.74)	-0.55(-0.62, -0.48)
**Neglected tropical diseases and malaria**						
65-69 years	345.32(221.84-510.55)	123.41(74.92-193.50)	-3.41(-3.57, -3.3)	-4.73(-5.03, -4.38)	-3.49(-3.8, -3.02)	-2.61(-3.1, -2.21)
70-74 years	359.95(235.71-514.22)	127.63(76.89-194.79)	-3.42(-3.58, -3.31)	-4.63(-4.95, -4.28)	-3.52(-3.82, -3.04)	-2.52(-3.04, -2.11)
75-79 years	358.55(242.25-516.73)	130.15(78.69-196.20)	-3.1(-3.29, -2.98)	-4.07(-4.24, -3.91)	-3.62(-4.08, -3.33)	-1.73(-2.46, -1.21)
80-84 years	342.14(233.96-476.37)	131.24(84.18-194.06)	-2.96(-3.09, -2.87)	-3.84(-3.96, -3.75)	-3.84(-3.94, -3.52)	-1.29(-1.76, -0.78)
85-89 years	326.63(229.66-448.77)	129.26(84.80-189.42)	-3.04(-3.15, -2.94)	-3.84(-4.16, -3.55)	-3.55(-3.85, -3.21)	-1.77(-2.1, -1.34)
90-94 years	316.00(222.06-437.50)	128.46(85.55-191.52)	-3.01(-3.11, -2.92)	-3.84(-4.07, -3.54)	-3.44(-3.73, -3.14)	-1.83(-2.16, -1.41)
95+ years	275.58(181.59-389.93)	130.84(79.66-208.36)	-2.39(-2.55, -2.3)	-2.8(-3.09, -2.51)	-2.61(-2.9, -2.44)	-1.54(-2.05, -1.23)
**Neoplasms**						
65-69 years	19,619.64(16,963.77-22,378.52)	14,196.11(11,701.87-16,944.56)	-1.01(-1.15, -0.91)	-0.24(-0.42, -0.01)	-1.32(-1.55, -1.11)	-1.55(-2.07, -1.27)
70-74 years	21,956.63(19,219.35-24,784.31)	17,562.40(14,477.21-21,084.29)	-0.71(-0.8, -0.62)	-0.05(-0.23, 0.12)	-1.52(-1.67, -1.4)	-0.57(-0.86, -0.3)

75-79 years	21,011.89(18,619.75-23,423.60)	18,577.50(15,456.12-21,987.40)	-0.41(-0.51, -0.31)	0.3(0.08, 0.53)	-0.75(-0.92, -0.62)	-0.94(-1.16, -0.6)
80-84 years	18,321.19(16,252.33-20,317.65)	18,116.95(15,253.35-21,170.37)	-0.05(-0.19, 0.05)	0.49(0.14, 0.76)	0.11(-0.07, 0.35)	-0.94(-1.22, -0.72)
85-89 years	19,131.21(16,814.48-20,934.36)	19,374.96(16,292.65-22,191.42)	-0.01(-0.1, 0.09)	0.96(0.68, 1.2)	-0.57(-0.75, -0.38)	-0.7(-0.91, -0.41)
90-94 years	20,526.19(17,679.60-22,578.61)	18,100.85(14,830.23-21,025.60)	-0.43(-0.56, -0.33)	0.53(0.32, 0.78)	-1.14(-1.36, -0.97)	-0.69(-1.2, -0.34)
95+ years	15,815.33(12,758.78-17,945.96)	14,893.67(11,330.19-17,719.23)	-0.17(-0.3, -0.06)	-0.09(-0.31, 0.21)	-1.26(-1.64, -1.05)	1.13(0.66, 1.58)
**Neurological disorders**						
65-69 years	2,245.32(1,334.69-3,839.01)	2,368.09(1,423.59-4,096.90)	0.15(0.1, 0.18)	0.25(0.18, 0.35)	-0.07(-0.14, -0.02)	0.28(0.12, 0.38)
70-74 years	3,525.43(2,167.79-6,182.98)	3,753.33(2,390.18-6,650.57)	0.16(0.11, 0.19)	0.24(0.19, 0.31)	-0.19(-0.27, -0.14)	0.45(0.29, 0.56)
75-79 years	6,056.98(3,651.29-11,403.68)	6,419.33(4,068.51-11,900.84)	0.14(0.08, 0.17)	0.21(0.15, 0.31)	-0.21(-0.31, -0.16)	0.46(0.27, 0.57)
80-84 years	12,485.04(6,786.69-25,865.87)	13,073.96(7,379.67-25,202.14)	0.11(0.06, 0.15)	0.09(0.03, 0.2)	-0.17(-0.26, -0.11)	0.47(0.3, 0.58)
85-89 years	20,579.92(10,487.38-42,850.60)	21,540.83(11,348.94-43,212.19)	0.11(0.06, 0.14)	0.2(0.13, 0.29)	-0.29(-0.38, -0.23)	0.46(0.29, 0.57)
90-94 years	31,221.64(14,268.29-68,427.62)	33,048.20(16,078.22-68,493.64)	0.17(0.11, 0.22)	0.35(0.24, 0.47)	-0.16(-0.22, -0.12)	0.41(0.24, 0.57)
95+ years	48,198.73(19,365.35-110,448.29)	49,987.36(22,003.41-110,525.45)	0.07(-0.03, 0.12)	-0.06(-0.13, -0.01)	-0.06(-0.11, -0.01)	0.37(0.06, 0.57)
**Nutritional deficiencies**						
65-69 years	407.99(292.66-555.71)	154.70(102.63-229.41)	-3.13(-3.16, -3.11)	-2.98(-3.05, -2.9)	-3.91(-3.99, -3.85)	-2.44(-2.53, -2.35)
70-74 years	557.79(417.99-739.14)	206.65(145.08-291.74)	-3.22(-3.26, -3.19)	-3.26(-3.34, -3.17)	-3.9(-3.97, -3.83)	-2.4(-2.51, -2.29)
75-79 years	725.62(563.84-928.91)	279.37(204.65-378.32)	-3.05(-3.09, -3.01)	-3.24(-3.33, -3.14)	-3.79(-3.86, -3.73)	-1.99(-2.1, -1.88)
80-84 years	1,145.80(926.27-1,409.19)	470.09(365.50-608.61)	-2.84(-2.88, -2.81)	-3.18(-3.27, -3.1)	-3.47(-3.56, -3.39)	-1.67(-1.82, -1.56)
85-89 years	1,860.55(1,587.60-2,152.64)	780.53(640.81-963.43)	-2.76(-2.83, -2.69)	-3.29(-3.47, -3.11)	-3.15(-3.28, -3.02)	-1.71(-1.89, -1.51)
90-94 years	3,692.73(3,170.26-4,247.74)	1,337.11(1,106.54-1,597.93)	-3.28(-3.41, -3.16)	-4.7(-4.92, -4.43)	-2.89(-3.01, -2.76)	-2.07(-2.49, -1.78)
95+ years	6,333.43(5,271.44-7,455.96)	2,502.36(1,943.90-3,004.17)	-2.83(-2.97, -2.68)	-4.42(-4.75, -4)	-2.9(-3.15, -2.64)	-0.84(-1.09, -0.56)
**Other infectious diseases**						

65-69 years	374.72(312.66-442.77)	64.04(52.81-76.60)	-5.49(-5.56, -5.4)	-6.26(-6.54, -5.99)	-7.13(-7.28, -7.01)	-2.55(-2.68, -2.43)
70-74 years	397.36(332.80-471.13)	72.33(59.33-86.66)	-5.39(-5.47, -5.31)	-6.74(-6.96, -6.5)	-6.56(-6.74, -6.42)	-2.42(-2.6, -2.26)
75-79 years	426.68(357.25-495.59)	83.96(69.19-100.23)	-5.01(-5.11, -4.91)	-6.34(-6.63, -6.02)	-6.12(-6.28, -6.01)	-2.15(-2.32, -1.98)
80-84 years	437.27(363.02-507.25)	101.63(83.53-121.80)	-4.57(-4.65, -4.48)	-5.45(-5.67, -5.17)	-5.68(-5.81, -5.57)	-2.19(-2.35, -2.05)
85-89 years	558.86(465.00-647.80)	134.38(109.50-163.55)	-4.51(-4.59, -4.42)	-4.72(-4.91, -4.44)	-5.97(-6.1, -5.85)	-2.73(-2.88, -2.58)
90-94 years	684.24(553.79-792.33)	171.45(139.53-207.89)	-4.49(-4.58, -4.38)	-4.81(-5.03, -4.5)	-5.22(-5.43, -5.06)	-3.22(-3.46, -2.96)
95+ years	825.06(652.62-1,016.99)	241.23(185.21-296.12)	-3.88(-4.01, -3.73)	-4.45(-4.78, -4.06)	-4.49(-4.74, -4.27)	-2.4(-2.79, -2.07)
**Other non-communicable diseases**						
65-69 years	1,798.18(1,241.14-2,562.01)	1,432.05(962.40-2,088.67)	-0.64(-0.7, -0.57)	-1.31(-1.5, -1.17)	-0.85(-1, -0.69)	0.38(0.19, 0.59)
70-74 years	2,136.28(1,517.74-2,915.42)	1,733.53(1,197.47-2,420.23)	-0.58(-0.65, -0.51)	-1.27(-1.46, -1.12)	-0.48(-0.73, -0.34)	0.39(0.2, 0.63)
75-79 years	2,391.30(1,716.95-3,202.53)	2,012.14(1,419.67-2,730.45)	-0.47(-0.54, -0.4)	-1.1(-1.28, -0.95)	-0.52(-0.68, -0.29)	0.5(0.29, 0.71)
80-84 years	2,593.95(1,893.42-3,420.82)	2,269.69(1,620.73-3,023.85)	-0.37(-0.43, -0.31)	-0.84(-0.98, -0.69)	-0.39(-0.52, -0.2)	0.37(0.19, 0.54)
85-89 years	2,885.22(2,172.55-3,701.58)	2,556.81(1,907.89-3,283.64)	-0.34(-0.4, -0.29)	-0.38(-0.51, -0.25)	-0.71(-0.8, -0.62)	0.09(-0.07, 0.23)
90-94 years	3,358.70(2,574.93-4,257.73)	2,889.86(2,178.37-3,645.64)	-0.42(-0.48, -0.37)	-0.28(-0.35, -0.2)	-0.45(-0.59, -0.27)	-0.39(-0.59, -0.2)
95+ years	3,587.50(2,807.48-4,497.76)	3,180.33(2,448.23-3,996.21)	-0.35(-0.39, -0.29)	-0.28(-0.34, -0.2)	-0.47(-0.63, -0.4)	-0.09(-0.28, 0.11)
**Respiratory infections and tuberculosis**						
65-69 years	3,072.44(2,630.48-3,552.78)	608.64(506.18-733.40)	-5.1(-5.24, -4.97)	-4.59(-4.77, -4.38)	-6.64(-6.87, -6.51)	-3.93(-4.39, -3.49)
70-74 years	4,440.47(3,792.23-5,040.21)	1,026.23(854.25-1,222.20)	-4.63(-4.71, -4.54)	-4.16(-4.27, -4.04)	-5.94(-6.07, -5.85)	-3.67(-3.93, -3.39)
75-79 years	5,999.06(5,115.84-6,761.30)	1,556.63(1,284.64-1,883.43)	-4.38(-4.48, -4.3)	-4.24(-4.46, -3.99)	-5.24(-5.38, -5.08)	-3.75(-4.03, -3.51)
80-84 years	9,033.13(7,387.79-10,100.70)	2,860.69(2,346.70-3,454.80)	-3.71(-3.87, -3.56)	-3.33(-3.63, -2.98)	-5.18(-5.44, -4.93)	-2.49(-2.96, -1.97)
85-89 years	13,752.07(11,406.72-15,349.76)	5,240.78(4,338.18-6,368.49)	-3.1(-3.2, -2.99)	-1.88(-2.03, -1.72)	-4.83(-5, -4.71)	-2.53(-2.85, -2.17)
90-94 years	25,096.86(20,617.28-28,337.08)	9,390.08(7,346.21-11,669.22)	-3.22(-3.4, -3.06)	-1.79(-2.12, -1.4)	-4.83(-5.11, -4.54)	-2.97(-3.54, -2.45)

95+ years	34,942.55(25,051.10-40,904.96)	13,182.44(9,513.55-17,226.14)	-3.19(-3.35, -3.06)	-2.5(-2.78, -2.19)	-4.72(-5.14, -4.46)	-2.04(-2.61, -1.53)
**Self-harm and interpersonal violence**						
65-69 years	1,378.69(1,025.67-1,576.82)	518.98(433.95-621.73)	-3.16(-3.24, -3.09)	-1.75(-1.89, -1.59)	-4.81(-4.91, -4.71)	-2.88(-3.17, -2.7)
70-74 years	1,608.61(1,116.13-1,819.89)	631.47(528.07-754.77)	-3.04(-3.09, -2.99)	-1.51(-1.62, -1.4)	-5.19(-5.28, -5.04)	-2.32(-2.47, -2.16)
75-79 years	1,641.20(1,130.76-1,862.49)	682.56(572.93-808.86)	-2.87(-2.95, -2.79)	-1.01(-1.21, -0.8)	-4.82(-4.98, -4.69)	-2.62(-2.86, -2.41)
80-84 years	1,669.48(1,129.41-1,875.70)	729.39(610.90-857.47)	-2.71(-2.8, -2.62)	-0.82(-1.07, -0.59)	-4.32(-4.5, -4.13)	-2.74(-3.01, -2.52)
85-89 years	1,850.48(1,209.39-2,102.50)	856.80(714.76-1,035.05)	-2.53(-2.66, -2.4)	-0.28(-0.68, 0.06)	-4.48(-4.64, -4.17)	-2.75(-3.04, -2.5)
90-94 years	1,608.55(1,099.55-1,825.40)	720.68(596.87-852.33)	-2.54(-2.67, -2.44)	-0.72(-1, -0.39)	-4.17(-4.35, -4)	-2.58(-2.97, -2.34)
95+ years	1,115.14(703.97-1,299.37)	532.22(417.37-641.33)	-2.44(-2.53, -2.35)	-2.34(-2.68, -2.04)	-3.64(-3.98, -3.44)	-0.85(-1.13, -0.59)
**Sense organ diseases**						
65-69 years	3,374.22(2,215.38-4,863.13)	3,670.39(2,400.83-5,262.42)	0.32(0.29, 0.36)	0.38(0.29, 0.48)	-0.01(-0.1, 0.07)	0.58(0.46, 0.67)
70-74 years	4,332.70(2,973.76-6,028.05)	4,544.56(3,114.34-6,285.72)	0.2(0.17, 0.23)	0.35(0.26, 0.45)	-0.08(-0.16, 0.01)	0.26(0.16, 0.34)
75-79 years	5,410.20(3,877.43-7,346.80)	5,393.18(3,826.55-7,325.05)	0.02(-0.01, 0.05)	0.14(0.05, 0.25)	-0.08(-0.11, 0)	-0.08(-0.12, -0.05)
80-84 years	6,423.97(4,599.09-8,568.12)	6,152.64(4,382.54-8,150.98)	-0.12(-0.15, -0.09)	-0.07(-0.15, 0.01)	-0.1(-0.16, -0.05)	-0.24(-0.28, -0.2)
85-89 years	7,385.90(5,430.72-9,734.18)	6,887.61(5,064.82-9,061.46)	-0.22(-0.25, -0.19)	-0.16(-0.25, -0.08)	-0.21(-0.27, -0.16)	-0.34(-0.39, -0.3)
90-94 years	8,252.36(6,124.29-10,672.25)	7,564.56(5,618.44-9,945.36)	-0.25(-0.29, -0.22)	-0.26(-0.33, -0.18)	-0.29(-0.34, -0.25)	-0.22(-0.3, -0.14)
95+ years	8,984.19(6,728.91-11,927.16)	8,156.61(6,038.26-10,807.92)	-0.28(-0.31, -0.25)	-0.4(-0.49, -0.34)	-0.31(-0.35, -0.27)	-0.08(-0.15, 0.01)
**Skin and subcutaneous diseases**						
65-69 years	504.37(335.06-737.44)	529.18(347.24-794.69)	0.15(0.15, 0.16)	0.18(0.17, 0.19)	0.04(0.03, 0.04)	0.23(0.22, 0.23)
70-74 years	563.11(379.39-839.97)	575.49(374.28-882.98)	0.07(0.06, 0.07)	0.13(0.1, 0.15)	-0.15(-0.17, -0.12)	0.2(0.18, 0.22)
75-79 years	619.79(425.29-902.62)	607.37(397.35-912.54)	-0.08(-0.1, -0.06)	0.04(-0.02, 0.1)	-0.42(-0.46, -0.35)	0.11(0.05, 0.18)
80-84 years	720.54(506.13-1,012.85)	649.47(423.05-966.24)	-0.34(-0.36, -0.32)	-0.15(-0.21, -0.09)	-0.76(-0.8, -0.7)	-0.1(-0.15, -0.04)

85-89 years	970.40(717.57-1,309.00)	759.55(525.25-1,130.23)	-0.8(-0.85, -0.75)	-0.18(-0.3, -0.08)	-1.91(-2.03, -1.77)	-0.41(-0.56, -0.25)
90-94 years	1,399.73(1,115.07-1,777.79)	904.34(663.99-1,262.96)	-1.43(-1.5, -1.37)	-0.68(-0.84, -0.49)	-2.71(-2.84, -2.57)	-0.91(-1.11, -0.71)
95+ years	1,794.16(1,410.84-2,220.63)	1,074.76(818.68-1,410.55)	-1.69(-1.78, -1.61)	-0.74(-0.91, -0.56)	-3.33(-3.57, -3.17)	-0.69(-1.01, -0.37)
**Substance use disorders**						
65-69 years	325.88(268.57-388.76)	199.69(151.27-258.78)	-1.67(-1.78, -1.58)	-1.76(-1.94, -1.53)	-1.28(-1.45, -1.12)	-1.98(-2.33, -1.72)
70-74 years	268.55(218.24-328.99)	171.50(128.99-222.72)	-1.49(-1.56, -1.43)	-1.27(-1.4, -1.13)	-1.26(-1.37, -1.15)	-2.04(-2.24, -1.86)
75-79 years	252.77(207.38-305.69)	160.25(123.97-207.49)	-1.51(-1.59, -1.44)	-1.36(-1.49, -1.2)	-1.48(-1.59, -1.37)	-1.62(-1.86, -1.44)
80-84 years	218.14(174.87-268.22)	146.72(111.87-191.33)	-1.32(-1.39, -1.26)	-0.98(-1.13, -0.83)	-1.57(-1.79, -1.45)	-1.16(-1.43, -0.92)
85-89 years	209.89(169.84-252.51)	145.39(113.59-187.28)	-1.2(-1.24, -1.16)	0.13(0, 0.27)	-2.79(-2.89, -2.62)	-0.88(-0.98, -0.78)
90-94 years	211.25(173.57-257.75)	138.84(102.23-190.16)	-1.42(-1.51, -1.35)	-0.46(-0.6, -0.31)	-2.23(-2.38, -2.07)	-1.27(-1.57, -1.01)
95+ years	163.46(126.78-212.67)	130.72(93.56-183.50)	-0.74(-0.79, -0.7)	-1.07(-1.15, -0.97)	-0.75(-0.82, -0.69)	-0.37(-0.53, -0.26)
**Transport injuries**						
65-69 years	1,106.65(953.31-1,270.81)	957.39(810.19-1,119.15)	-0.47(-0.6, -0.35)	0.78(0.46, 1.1)	0.66(0.36, 0.93)	-3.52(-3.91, -3.03)
70-74 years	1,049.11(907.97-1,207.14)	931.87(794.94-1,091.07)	-0.32(-0.38, -0.28)	1.06(0.92, 1.19)	-0.04(-0.14, 0.07)	-2.35(-2.54, -2.2)
75-79 years	952.12(823.32-1,096.67)	858.56(733.57-1,004.50)	-0.37(-0.48, -0.29)	1.47(1.25, 1.73)	-0.1(-0.34, 0.04)	-2.83(-3.16, -2.56)
80-84 years	820.12(709.66-963.23)	773.67(660.53-910.91)	-0.21(-0.32, -0.12)	1.59(1.32, 1.85)	0.26(0.05, 0.47)	-2.85(-3.22, -2.59)
85-89 years	789.00(684.23-917.23)	810.96(692.31-947.85)	0.18(0.03, 0.32)	2.23(1.96, 2.49)	-0.03(-0.2, 0.12)	-2.19(-2.72, -1.78)
90-94 years	717.60(614.52-850.94)	732.88(621.07-860.83)	0.05(-0.06, 0.16)	1.87(1.57, 2.2)	0.29(0.18, 0.44)	-2.4(-2.57, -2.24)
95+ years	568.23(479.76-677.53)	611.84(498.75-721.57)	0.35(0.24, 0.48)	0.92(0.56, 1.25)	0.54(0.27, 0.8)	-0.91(-1.33, -0.55)
**Unintentional injuries**						
65-69 years	1,771.41(1,499.74-2,084.60)	1,413.07(1,099.26-1,767.03)	-0.74(-0.99, -0.51)	-0.74(-0.99, -0.51)	-0.74(-0.99, -0.51)	-0.74(-0.99, -0.51)
70-74 years	2,042.68(1,734.34-2,419.65)	1,725.58(1,344.26-2,131.01)	-0.52(-0.76, -0.3)	-0.52(-0.76, -0.3)	-0.52(-0.76, -0.3)	-0.52(-0.76, -0.3)

75-79 years	2,517.24(2,151.21-2,940.15)	2,260.18(1,734.26-2,790.56)	-0.31(-0.55, -0.09)	-0.31(-0.55, -0.09)	-0.31(-0.55, -0.09)	-0.31(-0.55, -0.09)
80-84 years	3,461.48(2,972.00-4,029.50)	3,358.63(2,590.69-4,085.90)	-0.03(-0.26, 0.22)	-0.17(-0.76, 0.4)	-0.5(-0.89, 0.24)	0.48(-0.41, 1.41)
85-89 years	5,296.39(4,597.57-6,228.92)	5,503.94(4,178.70-6,631.72)	0.2(0.07, 0.37)	0.39(0.06, 0.65)	-0.4(-0.75, -0.06)	0.52(-0.15, 1.1)
90-94 years	7,808.36(6,756.55-9,202.81)	8,255.87(5,982.68-10,071.65)	0.21(0.11, 0.32)	0.23(0.02, 0.43)	-0.31(-0.6, -0.06)	0.65(0.13, 1.02)
95+ years	9,259.99(7,728.07-10,908.05)	11,039.46(7,555.71-14,032.95)	0.69(0.58, 0.81)	-0.34(-0.6, 0.07)	0.11(-0.29, 0.49)	2.24(1.63, 2.7)
**Number**						
**Cardiovascular diseases**						
65-69 years	8,288,083(7,316,573-9,369,665)	13,212,527(11,105,407-15,509,876)	1.48(1.25,1.72)	1.07(0.86,1.28)	-0.03(-0.67,0.61)	3.33(3.23,3.43)
70-74 years	8,500,369(7,550,219-9,410,029)	14,961,469(12,643,939-17,387,426)	1.84(1.67,2.00)	2.04(1.78,2.31)	0.59(0.38,0.80)	3.11(2.84,3.37)
75-79 years	6,807,309(6,078,299-7,556,740)	13,370,244(11,305,275-15,491,487)	2.17(1.93,2.42)	2.94(2.69,3.18)	2.94(2.69,3.18)	0.59(-0.02,1.20)
80-84 years	4,174,227(3,682,336-4,589,163)	12,180,668(10,410,936-13,987,318)	3.70(3.51,3.89)	4.22(4.01,4.42)	4.22(4.01,4.42)	2.62(2.17,3.08)
85-89 years	1,834,844(1,616,624-2,020,121)	8,909,097(7,550,210-10,109,177)	5.12(4.57,5.67)	5.82(5.12,6.52)	4.54(3.11,5.98)	4.68(4.48,4.87)
90-94 years	451,604(394,972-500,864)	3,755,762(3,073,324-4,325,210)	7.09(6.54,7.63)	8.36(8.00,8.73)	7.05(5.44,8.68)	5.74(5.51,5.96)
95+ years	68,783(55,527-77,893)	965,424(736,510-1,152,706)	9.34(8.88,9.81)	8.38(7.79,8.97)	9.66(9.20,10.13)	9.91(8.98,10.85)
**Chronic respiratory diseases**						
65-69 years	4,011,942(3,461,094-4,522,326)	2,989,622(2,482,767-3,571,743)	-1.08(-1.46, -0.71)	-1.04(-1.26, -0.82)	-3.76(-4.68, -2.84)	1.89(1.36,2.42)
70-74 years	5,148,706(4,439,174-5,768,896)	4,158,184(3,437,167-5,011,170)	-0.77(-1.00, -0.55)	0.08(-0.35,0.51)	-3.35(-3.59, -3.11)	1.58(1.16,2.00)
75-79 years	4,394,830(3,816,877-4,881,563)	4,192,709(3,528,638-4,974,925)	-0.29(-0.60,0.02)	0.61(0.21,1.01)	-1.49(-1.74, -1.23)	0.09(-0.77,0.94)
80-84 years	2,844,148(2,410,041-3,134,838)	4,004,389(3,319,015-4,615,577)	1.08(0.81,1.36)	2.11(1.56,2.67)	-0.10(-0.38,0.19)	1.53(0.97,2.08)
85-89 years	1,347,773(1,149,704-1,498,587)	3,015,578(2,512,626-3,479,461)	2.54(2.15,2.92)	4.03(3.74,4.33)	0.22(-0.73,1.17)	3.47(2.95,3.99)
90-94 years	333,281(277,641-376,209)	1,215,400(980,615-1,422,917)	4.27(4.05,4.49)	5.98(5.59,6.36)	2.66(2.42,2.91)	4.55(4.11,5.00)
95+ years	45,331(34,191-51,374)	293,191(218,686-361,577)	6.41(5.92,6.89)	5.71(5.04,6.38)	5.09(3.84,6.36)	8.52(8.27,8.78)

**Diabetes and kidney diseases**						
65-69 years	926,911(791,007-1,069,740)	2,456,046(2,032,687-2,949,370)	3.28(2.89,3.67)	3.20(3.02,3.37)	1.21(0.06,2.37)	5.49(5.26,5.72)
70-74 years	780,233(670,793-899,443)	2,174,224(1,834,786-2,564,934)	3.39(3.25,3.53)	3.91(3.67,4.15)	2.06(1.88,2.23)	4.43(4.23,4.63)
75-79 years	545,576(473,890-623,958)	1,577,232(1,338,100-1,842,106)	3.38(3.04,3.72)	4.04(3.39,4.70)	3.93(3.40,4.45)	1.97(1.65,2.28)
80-84 years	262,827(226,396-300,483)	1,045,375(883,932-1,222,524)	4.59(4.45,4.73)	5.09(4.99,5.18)	5.09(4.99,5.18)	3.40(2.99,3.81)
85-89 years	108,049(94,905-123,453)	632,815(542,300-727,129)	5.82(5.36,6.28)	6.30(5.69,6.91)	5.03(3.86,6.22)	5.91(5.78,6.05)
90-94 years	26,533(22,906-30,465)	234,592(200,239-270,578)	7.36(7.16,7.55)	8.38(7.99,8.77)	6.74(6.47,7.01)	7.06(6.86,7.27)
95+ years	3,981(3,352-4,627)	56,642(45,481-68,197)	9.23(8.47,9.99)	8.01(7.49,8.54)	8.75(6.58,10.96)	10.84(10.32,11.36)
**Digestive diseases**						
65-69 years	1,002,810(859,590-1,151,588)	1,135,994(934,628-1,363,163)	0.41(0.04,0.77)	-0.04(-0.20,0.12)	-1.25(-2.25, -0.23)	2.79(2.47,3.11)
70-74 years	822,214(707,723-938,916)	974,749(805,991-1,165,282)	0.52(0.34,0.70)	0.35(0.13,0.58)	-0.70(-0.96, -0.43)	2.16(1.87,2.46)
75-79 years	581,565(504,753-666,025)	735,880(619,059-867,114)	0.71(0.34,1.07)	0.57(0.19,0.96)	0.80(0.38,1.22)	0.61(-0.28,1.50)
80-84 years	300,277(257,774-346,115)	538,809(448,211-632,913)	1.93(1.47,2.39)	1.88(1.62,2.14)	2.07(0.69,3.47)	1.83(1.68,1.97)
85-89 years	124,752(105,329-145,879)	350,965(288,431-414,230)	3.27(2.82,3.72)	3.41(2.65,4.19)	2.09(1.45,2.73)	4.30(3.80,4.80)
90-94 years	29,158(24,228-34,659)	133,107(107,838-159,545)	4.98(4.76,5.19)	5.55(5.27,5.82)	4.06(3.56,4.56)	5.27(4.99,5.56)
95+ years	4,346(3,393-5,332)	33,107(24,807-41,032)	6.94(6.06,7.82)	4.93(3.52,6.35)	6.39(4.47,8.35)	9.35(9.02,9.69)
**Enteric infections**						
65-69 years	41,127(21,694-59,792)	13,909(9,837-20,742)	-3.50(-3.88, -3.13)	-5.63(-5.78, -5.47)	-5.91(-6.79, -5.03)	1.88(1.43,2.32)
70-74 years	42,479(21,314-63,206)	12,248(8,640-19,367)	-4.02(-4.39, -3.66)	-5.37(-5.59, -5.15)	-6.37(-6.63, -6.11)	0.37(-0.61,1.37)
75-79 years	37,533(18,017-56,213)	10,163(6,676-16,937)	-4.17(-4.46, -3.88)	-5.42(-5.87, -4.96)	-4.98(-5.12, -4.84)	-1.81(-2.59, -1.02)
80-84 years	25,234(11,432-38,013)	8,507(5,502-14,884)	-3.48(-3.74, -3.21)	-4.52(-4.75, -4.29)	-4.91(-5.33, -4.48)	-0.50(-0.94, -0.06)
85-89 years	12,668(5,784-19,242)	6,619(4,244-11,753)	-2.26(-2.78, -1.73)	-3.19(-3.46, -2.92)	-4.40(-5.70, -3.09)	1.33(0.65,2.02)

90-94 years	3,146(1,339-5,082)	2,800(1,686-5,137)	-0.42(-0.70, -0.14)	-0.99(-1.39, -0.59)	-1.68(-2.07, -1.29)	1.94(1.44,2.43)
95+ years	479(190-821)	739(437-1,422)	1.49(1.04,1.93)	-2.11(-3.15, -1.06)	1.28(0.59,1.96)	5.93(5.69,6.16)
**HIV/AIDS and sexually transmitted infections**						
65-69 years	6,007(3,387-8,489)	80,505(56,761-106,415)	8.61(7.90,9.33)	10.44(9.53,11.35)	2.06(1.14,2.99)	15.09(13.24,16.97)
70-74 years	3,683(2,327-5,217)	46,069(32,175-63,393)	8.74(8.19,9.29)	12.60(12.11,13.09)	2.75(2.08,3.43)	12.42(10.91,13.96)
75-79 years	3,035(2,283-4,026)	25,716(18,310-35,068)	7.82(6.54,9.12)	10.42(9.57,11.28)	7.90(6.39,9.44)	5.25(2.11,8.49)
80-84 years	929(517-2,032)	10,811(6,252-17,942)	8.15(6.79,9.51)	10.04(6.55,13.64)	2.79(1.97,3.61)	11.91(10.13,13.73)
85-89 years	302(177-574)	3,916(2,310-6,422)	8.45(7.16,9.75)	9.29(5.98,12.71)	2.80(2.08,3.53)	13.41(11.62,15.23)
90-94 years	60(36-101)	1,040(641-1,676)	9.70(8.37,11.05)	10.28(6.74,13.95)	5.48(4.70,6.27)	13.60(12.09,15.13)
95+ years	8(5-13)	186(123-288)	9.97(8.95,11.00)	7.50(5.52,9.53)	9.13(7.56,10.72)	13.53(12.39,14.69)
**Maternal and neonatal disorders**						
65-69 years	10,714(7,451-15,701)	61,777(45,365-78,305)	5.92(5.73,6.11)	4.59(4.45,4.73)	5.04(4.91,5.17)	8.21(7.68,8.74)
70-74 years	4,259(3,019-6,004)	24,823(18,070-31,520)	5.92(5.69,6.15)	4.65(4.22,5.08)	5.63(5.30,5.96)	7.77(7.49,8.06)
75-79 years	1,725(1,183-2,505)	8,056(5,856-10,423)	5.05(4.92,5.18)	3.43(3.24,3.62)	6.63(6.34,6.92)	4.79(4.66,4.91)
80-84 years	672(441-983)	2,907(2,081-3,950)	4.78(4.53,5.02)	3.38(3.18,3.59)	5.64(5.00,6.29)	5.27(4.95,5.58)
85-89 years	197(134-287)	1,084(718-1,558)	5.65(5.45,5.85)	4.39(3.85,4.94)	5.63(5.55,5.71)	7.10(6.88,7.31)
90-94 years	34(22-50)	300(204-428)	7.34(7.20,7.48)	6.56(6.24,6.88)	7.26(7.13,7.39)	8.45(8.29,8.62)
95+ years	4(2-6)	59(40-86)	9.17(8.10,10.25)	7.00(6.20,7.80)	8.93(5.85,12.09)	11.60(10.99,12.22)
**Mental disorders**						
65-69 years	507,845(378,966-660,328)	1,572,378(1,180,770-2,033,197)	3.67(3.43,3.92)	3.45(3.16,3.74)	1.70(1.18,2.23)	6.47(6.23,6.71)
70-74 years	340,390(253,339-440,089)	1,063,480(793,655-1,370,386)	3.70(3.57,3.84)	3.97(3.71,4.22)	2.40(2.24,2.55)	5.21(4.99,5.42)
75-79 years	199,663(144,114-258,837)	638,438(459,419-828,957)	3.79(3.53,4.05)	4.40(3.73,5.08)	4.01(3.93,4.09)	2.83(2.37,3.28)

80-84 years	90,560(66,517-118,516)	367,190(269,075-477,047)	4.61(4.54,4.69)	5.22(5.08,5.36)	4.64(4.55,4.73)	3.93(3.81,4.06)
85-89 years	28,356(20,813-37,091)	168,919(126,054-218,365)	5.87(5.56,6.19)	5.97(5.73,6.21)	5.38(4.46,6.30)	6.26(6.17,6.36)
90-94 years	5,078(3,646-6,652)	49,715(36,072-64,964)	7.69(7.52,7.87)	8.40(8.06,8.74)	7.28(7.09,7.48)	7.61(7.38,7.85)
95+ years	661(459-899)	10,356(7,324-13,947)	9.66(8.76,10.55)	8.59(7.88,9.29)	9.53(6.98,12.14)	10.76(10.24,11.28)
**Musculoskeletal disorders**						
65-69 years	1,192,715(819,291-1,677,885)	3,343,391(2,300,106-4,758,764)	3.50(3.39,3.61)	2.10(1.97,2.22)	2.49(2.38,2.60)	6.10(5.86,6.34)
70-74 years	880,123(602,006-1,233,723)	2,410,497(1,648,345-3,384,799)	3.33(3.17,3.49)	2.50(2.17,2.84)	2.78(2.63,2.94)	5.03(4.79,5.28)
75-79 years	549,372(392,988-777,469)	1,510,310(1,084,996-2,152,233)	3.32(3.23,3.40)	2.71(2.57,2.86)	4.31(4.24,4.39)	2.90(2.69,3.11)
80-84 years	250,923(178,189-357,199)	884,382(621,861-1,294,517)	4.16(4.03,4.29)	3.66(3.29,4.03)	4.88(4.83,4.93)	3.85(3.72,3.99)
85-89 years	77,082(54,178-111,011)	414,313(291,629-596,851)	5.52(5.39,5.65)	4.94(4.62,5.26)	5.70(5.58,5.82)	5.94(5.86,6.01)
90-94 years	12,849(8,880-17,893)	120,783(82,839-170,514)	7.60(7.39,7.80)	7.82(7.41,8.24)	7.93(7.56,8.31)	7.22(7.07,7.36)
95+ years	1,588(1,069-2,264)	25,306(17,500-36,448)	9.62(9.31,9.93)	7.85(7.43,8.27)	10.49(10.11,10.87)	10.45(9.97,10.93)
**Neglected tropical diseases and malaria**						
65-69 years	94,208(60,521-139,288)	94,663(57,465-148,421)	0.06(-0.35,0.47)	-2.22(-2.33, -2.10)	-1.24(-1.40, -1.07)	3.53(2.24,4.84)
70-74 years	67,733(44,355-96,764)	68,021(40,977-103,815)	0.09(-0.76,0.95)	-1.20(-1.38, -1.02)	-1.20(-1.38, -1.02)	2.75(-0.01,5.59)
75-79 years	40,805(27,569-58,807)	43,103(26,061-64,980)	0.31(-0.40,1.03)	-0.51(-0.74, -0.29)	0.21(-0.15,0.57)	1.12(-1.11,3.40)
80-84 years	18,123(12,393-25,233)	25,975(16,660-38,407)	1.22(0.89,1.56)	0.27(0.16,0.37)	0.78(0.64,0.92)	2.44(1.39,3.49)
85-89 years	5,509(3,873-7,570)	12,313(8,078-18,043)	2.63(2.23,3.03)	1.42(1.30,1.54)	1.42(1.30,1.54)	5.08(3.81,6.36)
90-94 years	969(681-1,342)	3,766(2,508-5,615)	4.48(4.09,4.87)	4.01(3.05,4.98)	3.59(3.20,3.97)	6.04(5.70,6.38)
95+ years	111(73-157)	836(509-1,331)	6.87(6.63,7.11)	4.95(4.57,5.33)	6.64(6.40,6.89)	9.24(8.94,9.55)
**Neoplasms**						
65-69 years	5,352,594(4,628,024-6,105,265)	10,888,918(8,975,745-12,997,070)	2.30(1.97,2.62)	1.83(1.50,2.16)	0.71(-0.17,1.60)	4.36(4.23,4.50)

70-74 years	4,131,722(3,616,630-4,663,825)	9,360,132(7,715,839-11,237,178)	2.68(2.53,2.83)	3.14(2.87,3.41)	0.96(0.78,1.14)	4.30(4.09,4.51)
75-79 years	2,391,299(2,119,057-2,665,768)	6,152,668(5,118,901-7,281,991)	3.18(2.77,3.60)	4.21(3.87,4.56)	3.41(2.88,3.95)	1.70(0.56,2.85)
80-84 years	970,496(860,906-1,076,251)	3,585,681(3,018,921-4,190,010)	4.46(4.32,4.60)	5.14(4.97,5.31)	4.95(4.80,5.10)	3.24(2.96,3.53)
85-89 years	322,715(283,635-353,132)	1,845,610(1,551,997-2,113,899)	5.70(5.18,6.22)	6.36(5.59,7.14)	4.72(3.59,5.85)	5.73(5.52,5.94)
90-94 years	62,978(54,244-69,276)	530,714(434,820-616,467)	7.19(6.89,7.48)	8.61(8.08,9.13)	6.21(5.58,6.84)	7.04(6.81,7.26)
95+ years	6,403(5,166-7,266)	95,185(72,411-113,243)	9.34(8.66,10.02)	7.92(7.45,8.39)	8.39(6.47,10.34)	11.79(11.26,12.31)
**Neurological disorders**						
65-69 years	612,563(364,127-1,047,351)	1,816,407(1,091,947-3,142,465)	3.53(3.26,3.81)	2.72(2.58,2.85)	1.88(1.08,2.68)	6.14(6.01,6.28)
70-74 years	663,403(407,926-1,163,491)	2,000,393(1,273,878-3,544,519)	3.58(3.40,3.75)	3.42(3.30,3.53)	2.29(2.14,2.44)	5.45(4.91,5.99)
75-79 years	689,327(415,542-1,297,818)	2,126,014(1,347,445-3,941,432)	3.66(3.54,3.78)	3.79(3.74,3.85)	3.79(3.74,3.85)	3.32(2.93,3.72)
80-84 years	661,348(359,499-1,370,147)	2,587,578(1,460,572-4,987,971)	4.47(4.28,4.65)	4.57(4.20,4.93)	4.43(4.23,4.64)	4.37(4.08,4.66)
85-89 years	347,153(176,906-722,827)	2,051,926(1,081,071-4,116,285)	5.80(5.59,6.00)	5.55(5.33,5.77)	5.07(4.63,5.51)	6.86(6.46,7.26)
90-94 years	95,794(43,778-209,951)	968,969(471,411-2,008,225)	7.76(7.52,8.00)	8.27(7.75,8.78)	7.02(6.72,7.31)	8.19(7.95,8.43)
95+ years	19,516(7,841-44,722)	319,468(140,623-706,366)	9.69(9.15,10.23)	8.25(7.67,8.84)	9.38(7.96,10.83)	11.37(10.96,11.78)
**Nutritional deficiencies**						
65-69 years	111,306(79,842-151,607)	118,660(78,717-175,966)	0.23(0.07,0.40)	-0.43(-0.82,-0.05)	-2.19(-2.44,-1.94)	3.54(3.36,3.73)
70-74 years	104,962(78,655-139,088)	110,137(77,322-155,484)	0.15(-0.01,0.31)	-0.14(-0.34,0.05)	-1.47(-1.55,-1.39)	2.69(2.19,3.18)
75-79 years	82,580(64,169-105,716)	92,523(67,776-125,294)	0.27(-0.01,0.55)	0.22(-0.16,0.60)	0.12(-0.63,0.87)	0.56(0.37,0.76)
80-84 years	60,694(49,065-74,646)	93,038(72,340-120,456)	1.37(1.21,1.53)	1.10(0.82,1.39)	1.00(0.85,1.15)	2.08(1.74,2.43)
85-89 years	31,384(26,780-36,311)	74,351(61,041-91,773)	2.86(2.49,3.23)	2.14(1.19,3.10)	1.97(1.43,2.51)	4.66(4.49,4.83)
90-94 years	11,330(9,727-13,033)	39,203(32,443-46,851)	4.09(3.85,4.32)	2.66(2.18,3.13)	4.26(4.02,4.50)	5.68(5.33,6.03)
95+ years	2,564(2,134-3,019)	15,992(12,423-19,199)	6.12(5.83,6.40)	2.81(2.47,3.14)	6.01(5.29,6.73)	9.66(9.32,10.00)

**Other infectious diseases**						
65-69 years	102,230(85,298-120,794)	49,123(40,505-58,752)	1.50(1.02,1.98)	-3.84(-4.15, -3.52)	-5.38(-7.18, -3.54)	-2.33(-2.97, -1.70)
70-74 years	74,773(62,625-88,655)	38,548(31,621-46,189)	1.00(0.65,1.35)	-3.62(-3.87, -3.38)	-4.35(-4.81, -3.89)	3.15(2.73,3.57)
75-79 years	48,559(40,657-56,401)	27,806(22,913-33,196)	3.51(3.31,3.71)	-2.97(-3.68, -2.25)	-2.52(-3.04, -2.00)	-2.20(-2.45, -1.95)
80-84 years	23,162(19,229-26,869)	20,115(16,532-24,106)	2.87(2.68,3.07)	-1.39(-1.66, -1.13)	-1.38(-1.63, -1.14)	2.41(1.99,2.83)
85-89 years	9,427(7,843-10,927)	12,800(10,430-15,579)	4.65(4.32,4.99)	0.79(-0.09,1.67)	-1.15(-1.71, -0.60)	-1.77(-2.09, -1.44)
90-94 years	2,099(1,699-2,431)	5,026(4,091-6,095)	5.20(4.73,5.67)	2.70(2.36,3.04)	1.71(1.48,1.93)	0.61(0.39,0.84)
95+ years	334(264-411)	1,541(1,183-1,892)	8.09(7.54,8.64)	2.98(2.57,3.39)	4.65(3.41,5.90)	-0.51(-0.72, -0.30)
**Other non-communicable diseases**						
65-69 years	490,576(338,604-698,963)	1,098,436(738,194-1,602,080)	4.27(3.26,5.28)	1.15(1.03,1.27)	1.15(1.03,1.27)	2.65(2.23,3.08)
70-74 years	401,997(285,602-548,614)	923,909(638,209-1,289,895)	5.32(4.90,5.73)	2.32(2.19,2.46)	1.63(1.44,1.83)	6.03(4.71,7.38)
75-79 years	272,146(195,401-364,470)	666,399(470,180-904,295)	6.56(6.21,6.90)	2.34(2.05,2.63)	3.63(3.49,3.78)	2.83(2.52,3.14)
80-84 years	137,404(100,296-181,205)	449,214(320,772-598,476)	7.13(6.77,7.49)	3.90(3.82,3.97)	3.90(3.82,3.97)	5.13(4.14,6.12)
85-89 years	48,669(36,647-62,440)	243,555(181,741-312,791)	7.38(7.24,7.53)	4.94(3.97,5.91)	4.56(4.29,4.82)	2.96(2.76,3.16)
90-94 years	10,305(7,900-13,063)	84,730(63,869-106,889)	9.21(8.65,9.77)	7.54(7.06,8.01)	6.70(5.75,7.65)	3.15(2.69,3.61)
95+ years	1,452(1,136-1,821)	20,325(15,646-25,539)	10.82(10.29,11.34)	7.92(7.31,8.53)	8.87(7.38,10.37)	4.00(3.71,4.30)
**Respiratory infections and tuberculosis**						
65-69 years	838,215(717,643-969,263)	466,845(388,254-562,546)	-1.74(-2.69, -0.78)	-2.35(-2.70, -2.00)	-4.69(-7.23, -2.09)	2.45(1.51,3.40)
70-74 years	835,591(713,608-948,449)	546,945(455,285-651,386)	-1.28(-1.81, -0.75)	-1.14(-1.35, -0.92)	-3.56(-4.95, -2.14)	1.37(0.71,2.03)
75-79 years	682,735(582,218-769,483)	515,540(425,458-623,773)	-0.98(-1.49, -0.48)	-0.64(-0.87, -0.40)	-1.63(-3.12, -0.12)	-0.72(-0.98, -0.46)
80-84 years	478,496(391,340-535,046)	566,184(464,456-683,769)	0.56(0.07,1.06)	0.93(0.42,1.45)	-0.54(-1.89,0.82)	1.41(1.19,1.63)
85-89 years	231,977(192,414-258,928)	499,223(413,244-606,646)	2.40(1.95,2.85)	3.19(2.97,3.42)	0.37(-0.86,1.61)	3.75(3.30,4.20)

90-94 years	77,002(63,258-86,944)	275,315(215,389-342,140)	4.19(3.95,4.44)	5.96(5.52,6.39)	2.39(2.08,2.69)	4.68(4.28,5.08)
95+ years	14,148(10,143-16,563)	84,248(60,800-110,091)	6.14(5.42,6.87)	5.38(4.92,5.84)	4.62(2.53,6.75)	8.55(8.31,8.79)
**Self-harm and interpersonal violence**						
65-69 years	376,130(279,821-430,186)	398,073(332,852-476,889)	0.08(-0.33,0.48)	0.58(0.41,0.75)	-2.84(-3.92, -1.74)	2.86(2.49,3.24)
70-74 years	302,702(210,030-342,459)	336,553(281,444-402,263)	0.29(-0.02,0.59)	1.64(1.52,1.76)	-2.73(-2.92, -2.54)	2.63(1.61,3.65)
75-79 years	186,780(128,688-211,964)	226,056(189,748-267,884)	0.56(0.30,0.81)	2.40(2.16,2.65)	-0.95(-1.16, -0.75)	0.34(-0.37,1.06)
80-84 years	88,434(59,826-99,358)	144,359(120,907-169,708)	1.54(1.35,1.73)	3.62(3.40,3.83)	-0.12(-0.37,0.14)	1.25(0.84,1.65)
85-89 years	31,214(20,400-35,466)	81,616(68,085-98,596)	3.04(2.56,3.51)	5.06(4.12,6.00)	0.54(0.09,1.00)	3.61(2.92,4.32)
90-94 years	4,935(3,373-5,600)	21,130(17,500-24,990)	4.74(4.51,4.96)	7.05(6.55,7.56)	2.71(2.51,2.91)	4.93(4.47,5.39)
95+ years	451(285-526)	3,401(2,667-4,098)	7.04(6.32,7.77)	5.92(5.46,6.37)	5.45(3.38,7.55)	10.16(9.74,10.58)
**Sense organ diseases**						
65-69 years	920,547(604,394-1,326,750)	2,815,321(1,841,524-4,036,458)	3.76(3.51,4.02)	3.00(2.25,3.75)	1.77(1.37,2.18)	3.78(3.47,4.09)
70-74 years	815,313(559,592-1,134,336)	2,422,087(1,659,833-3,350,063)	5.47(5.21,5.74)	3.42(2.83,4.01)	2.56(2.39,2.73)	6.62(6.37,6.86)
75-79 years	615,718(441,278-836,116)	1,786,163(1,267,311-2,425,977)	6.06(5.97,6.15)	3.55(3.33,3.78)	4.18(4.11,4.25)	3.61(3.38,3.84)
80-84 years	340,285(243,619-453,863)	1,217,721(867,385-1,613,229)	7.38(7.19,7.57)	4.39(4.30,4.49)	4.57(4.48,4.66)	5.15(4.86,5.43)
85-89 years	124,589(91,608-164,201)	656,096(482,461-863,172)	7.60(7.41,7.78)	5.39(4.67,6.12)	5.10(4.79,5.41)	3.50(3.39,3.61)
90-94 years	25,320(18,790-32,744)	221,791(164,731-291,596)	9.39(8.72,10.06)	7.74(7.38,8.11)	6.97(6.71,7.23)	2.77(2.52,3.02)
95+ years	3,637(2,724-4,829)	52,128(38,590-69,073)	10.89(10.42,11.37)	7.97(7.26,8.68)	9.23(7.45,11.03)	4.28(4.18,4.38)
**Skin and subcutaneous diseases**						
65-69 years	137,601(91,411-201,186)	405,900(266,344-609,552)	3.61(3.26,3.96)	2.73(2.52,2.95)	1.98(0.95,3.01)	6.27(6.08,6.45)
70-74 years	105,963(71,391-158,062)	306,715(199,477-470,595)	3.48(3.26,3.70)	3.24(3.14,3.34)	2.37(2.14,2.60)	5.24(4.55,5.93)
75-79 years	70,536(48,401-102,724)	201,154(131,598-302,222)	3.43(3.27,3.59)	3.57(3.49,3.65)	3.85(3.49,4.22)	2.92(2.61,3.24)

80-84 years	38,167(26,810-53,651)	128,541(83,729-191,237)	4.03(3.93,4.13)	4.37(4.21,4.53)	3.86(3.68,4.03)	3.85(3.66,4.03)
85-89 years	16,369(12,104-22,080)	72,352(50,034-107,662)	4.92(4.49,5.36)	5.21(4.94,5.49)	3.67(2.46,4.91)	6.02(5.69,6.35)
90-94 years	4,294(3,421-5,454)	26,515(19,467-37,029)	6.07(5.90,6.24)	7.27(6.99,7.55)	4.48(4.17,4.79)	6.89(6.68,7.10)
95+ years	726(571-899)	6,868(5,232-9,014)	7.60(7.17,8.03)	6.91(6.07,7.76)	5.77(4.89,6.66)	10.02(9.88,10.16)
**Substance use disorders**						
65-69 years	88,906(73,271-106,061)	153,172(116,029-198,493)	1.70(1.51,1.89)	0.68(0.32,1.04)	0.86(0.69,1.04)	3.80(3.45,4.15)
70-74 years	50,535(41,067-61,908)	91,403(68,745-118,703)	1.87(1.69,2.06)	1.88(1.52,2.25)	1.22(1.05,1.39)	2.81(2.49,3.14)
75-79 years	28,767(23,600-34,789)	53,072(41,057-68,717)	1.91(1.68,2.14)	2.16(1.69,2.63)	2.54(2.18,2.90)	0.81(0.55,1.08)
80-84 years	11,555(9,263-14,208)	29,039(22,141-37,868)	2.95(2.77,3.12)	3.39(3.07,3.71)	2.75(2.55,2.94)	2.84(2.56,3.13)
85-89 years	3,540(2,864-4,259)	13,849(10,820-17,839)	4.51(4.23,4.80)	6.08(5.27,6.90)	2.55(2.24,2.86)	5.43(5.33,5.53)
90-94 years	648(532-790)	4,070(2,997-5,575)	6.10(5.91,6.28)	7.37(7.04,7.71)	4.95(4.67,5.24)	6.46(6.28,6.65)
95+ years	66(51-86)	835(597-1,172)	8.68(8.36,9.00)	6.80(6.37,7.23)	8.40(7.59,9.20)	10.60(10.32,10.89)
**Transport injuries**						
65-69 years	301,914(260,081-346,700)	734,352(621,441-858,430)	3.06(2.40,3.73)	3.29(2.54,4.05)	2.61(0.84,4.41)	3.04(2.77,3.30)
70-74 years	197,417(170,857-227,156)	496,651(423,673-581,502)	3.09(2.92,3.25)	4.30(3.98,4.61)	2.45(2.26,2.64)	2.46(2.20,2.71)
75-79 years	108,357(93,699-124,808)	284,347(242,950-332,679)	3.01(2.57,3.45)	4.98(4.06,5.92)	3.95(3.23,4.67)	-0.26(-0.68,0.18)
80-84 years	43,442(37,591-51,023)	153,124(130,732-180,286)	4.27(3.98,4.56)	6.08(5.47,6.70)	4.94(4.44,5.45)	1.49(1.27,1.71)
85-89 years	13,309(11,542-15,472)	77,249(65,947-90,290)	5.84(5.51,6.17)	7.67(6.95,8.40)	5.45(5.12,5.77)	4.01(3.58,4.43)
90-94 years	2,201(1,885-2,610)	21,488(18,209-25,239)	7.78(7.57,7.99)	10.01(9.70,10.32)	7.86(7.41,8.32)	5.30(5.07,5.53)
95+ years	230(194-274)	3,910(3,187-4,611)	9.59(8.48,10.71)	8.14(4.92,11.46)	10.72(10.46,10.98)	9.71(8.77,10.67)
**Unintentional injuries**						
65-69 years	483,273(409,156-568,715)	1,083,875(843,171-1,355,372)	2.71(2.32,3.11)	1.09(0.70,1.49)	1.09(0.70,1.49)	6.20(5.19,7.21)

70-74 years	384,385(326,362-455,321)	919,670(716,443-1,135,755)	2.98(2.65,3.30)	2.72(2.55,2.89)	1.18(0.50,1.87)	5.95(5.56,6.35)
75-79 years	286,479(244,823-334,609)	748,548(574,368-924,204)	3.18(2.98,3.38)	3.18(2.98,3.38)	3.18(2.98,3.38)	3.18(2.98,3.38)
80-84 years	183,359(157,430-213,447)	664,735(512,745-808,674)	4.48(4.31,4.66)	4.48(4.31,4.66)	4.48(4.31,4.66)	4.48(4.31,4.66)
85-89 years	89,342(77,554-105,072)	524,291(398,052-631,721)	5.89(5.18,6.60)	5.76(4.30,7.23)	4.86(3.97,5.77)	7.05(6.39,7.72)
90-94 years	23,957(20,730-28,236)	242,060(175,411-295,299)	7.97(7.61,8.33)	8.37(7.86,8.88)	7.01(6.20,7.83)	8.49(8.09,8.90)
95+ years	3,749(3,129-4,416)	70,553(48,288-89,684)	10.01(9.02,11.01)	7.48(4.90,10.12)	9.66(9.16,10.15)	13.39(11.88,14.92)

UI, Uncertainty Interval; CI, Confidence Interval.

Supplementary Table 2. The age specific rate of disability-adjusted life years for level 3 causes in 1990 and 2021 and average annual percent change between 1990 and 2021, by age group.

	1990	2021	1990-2021	1990-2000	2001-2011	2012-2021
**Causes**	**N(95% UI)**	**N(95% UI)**	**AAPC (95%CI)**	**APC (95%CI)**	**APC (95%CI)**	**APC (95%CI)**
			**Age specific rate**			
**65-69 years**						
Acne vulgaris	5.96(3.79-9.30)	7.18(4.54-11.21)	0.61(0.58, 0.63)	0.65(0.64, 0.67)	0.63(0.56, 0.70)	0.54(0.52,0.55)
Acute glomerulonephritis	73.87(53.35-99.10)	21.74(14.40-29.77)	-3.90(-4.41, -3.39)	-4.30(-5.18, -3.41)	-2.92(-3.18, -2.66)	-4.76(-6.08, -3.41)
Acute hepatitis	215.46(173.30-264.75)	15.02(11.73-19.49)	-8.33(-8.73, -7.93)	-7.76(-8.27, -7.25)	-11.72(-12.26 -11.17)	-4.61(-5.61, -3.60)
Adverse effects of medical treatment	50.84(33.95-64.59)	11.97(9.40-16.34)	-4.65(-4.91, -4.39)	-4.30(-4.51, -4.09)	-6.47(-7.15, -5.79)	-3.18(-3.46, -2.90)
Age-related and other hearing loss	2, 088.43(1, 318.53-3, 078.76)	2, 363.39(1, 522.90-3, 480.47)	0.45(0.34, 0.56)	0.00(-0.12, 0.13)	0.38(0.14, 0.63)	1.02(0.88, 1.17)
Alcohol use disorders	135.82(100.35-176.61)	143.04(103.52-192.10)	0.11(-0.21, 0.42)	-0.33(-1.15, 0.51)	2.28(2.04, 2.51)	-2.25(-2.72, -1.78)
Alopecia areata	7.64(4.89-10.86)	7.64(4.91-11.01)	0.00(-0.03, 0.02)	-0.09(-0.13, -0.06)	0.03(-0.01, 0.07)	0.05(-0.01, 0.10)
Alzheimer's disease and other dementias	1, 292.90(556.59-2, 821.59)	1, 347.96(632.09-3, 067.69)	0.00(-0.03, 0.03)	0.00(-0.03, 0.03)	0.00(-0.03, 0.03)	0.00(-0.03, 0.03)
Animal contact	51.76(38.84-61.39)	11.75(9.49-14.66)	-4.88(-5.25, -4.51)	-3.47(-4.06, -2.88)	-6.95(-7.74, -6.16)	-4.11(-4.40, -3.82)
Anxiety disorders	508.44(327.83-750.17)	514.37(335.48-752.98)	-0.03(-0.44, 0.38)	0.23(-0.18, 0.64)	-1.09(-1.44, -0.74)	1.40(0.38, 2.43)
Aortic aneurysm	22.99(17.75-29.62)	30.99(24.15-40.08)	0.91(0.67, 1.16)	0.19(0.06, 0.32)	2.61(1.87, 3.34)	0.04(-0.17, 0.24)
Appendicitis	26.69(19.23-32.88)	6.01(4.43-7.79)	-4.75(-5.03, -4.47)	-3.82(-4.39, -3.25)	-5.93(-6.20, -5.65)	-4.39(-4.88, -3.88)
Asthma	505.55(391.55-678.13)	126.19(97.37-160.49)	-4.40(-4.67, -4.13)	-3.82(-3.95, -3.69)	-5.72(-6.33, -5.10)	-3.63(-4.23, -3.04)
Atrial fibrillation and flutter	232.77(167.71-308.11)	245.37(171.87-334.62)	0.14(-0.02, 0.30)	-0.71(-0.87, -0.55)	0.51(0.20, 0.82)	0.49(0.25, 0.74)
Attention-deficit/hyperactivity disorder	1.73(0.81-3.16)	1.51(0.66-2.80)	-0.45(-0.51, -0.38)	-1.01(-1.18, -0.84)	-0.31(-0.35, -0.27)	-0.02(-0.09, 0.05)
Autism spectrum disorders	87.23(60.00-122.93)	99.03(68.05-140.15)	0.41(0.39, 0.44)	0.60(0.54, 0.66)	0.38(0.35, 0.42)	0.26(0.24, 0.27)

Bacterial skin diseases	34.12(26.93-40.87)	7.35(5.56-9.84)	-4.82(-5.16, -4.48)	-3.25(-3.87, -2.63)	-10.90(-11.32, -10.47)	-0.26(-0.78, 0.26)
Bipolar disorder	47.57(29.81-71.97)	47.86(30.23-72.90)	0.02(-0.01, 0.05)	-0.01(-0.08, 0.06)	0.01(-0.02, 0.03)	0.05(0.01, 0.10)
Bladder cancer	294.47(221.55-350.73)	184.37(139.86-240.85)	-1.68(-2.19, -1.17)	-1.07(-1.47, -0.67)	-2.53(-3.96, -1.08)	-1.50(-1.93, -1.06)
Blindness and vision loss	1, 129.00(746.25-1, 731.84)	1, 144.22(704.41-1, 878.51)	0.06(-0.18, 0.30)	1.24(0.53, 1.95)	-0.50(-0.60, -0.39)	-0.50(-0.60, -0.39)
Brain and central nervous system cancer	301.34(229.53-394.60)	304.54(227.18-395.21)	0.00(-0.18, 0.18)	0.74(0.51, 0.98)	-0.44(-0.93, 0.06)	-0.41(-0.49, -0.33)
Breast cancer	371.46(309.03-442.17)	420.26(331.74-529.48)	0.39(-0.25, 1.04)	0.02(-0.16, 0.21)	0.56(-0.88, 2.02)	0.97(-0.29, 2.25)
Cardiomyopathy and myocarditis	90.41(73.11-131.86)	81.35(64.49-104.66)	-0.17(-0.53, 0.20)	2.55(2.18, 2.91)	-0.25(-0.77, 0.29)	-2.84(-3.64, -2.04)
Cervical cancer	302.85(247.35-365.14)	214.40(158.39-278.71)	-0.97(-1.31, -0.62)	-2.58(-2.90, -2.26)	-0.90(-1.80, 0.00)	0.23(0.00, 0.45)
Chronic kidney disease	1, 366.72(1, 166.73-1, 598.68)	1, 038.10(855.15-1, 244.53)	-0.83(-1.13, -0.53)	-0.87(-1.16, -0.58)	-0.90(-1.72, -0.09)	-1.03(-1.19, -0.87)
Chronic obstructive pulmonary disease	14, 046.89(12, 115.96-15, 770.37)	3, 673.83(3, 043.68-4, 405.90)	-4.38(-4.82, -3.93)	-3.67(-3.87, -3.47)	-5.74(-6.61, -4.87)	-3.66(-4.77, -2.54)
Cirrhosis and other chronic liver diseases	1, 765.76(1, 491.61-2, 056.49)	645.43(511.61-802.02)	-3.39(-3.77, -3.01)	-2.60(-2.95, -2.25)	-3.81(-4.81, -2.81)	-3.96(-4.18, -3.73)
Colon and rectum cancer	1, 441.57(1, 215.25-1, 659.87)	1, 293.51(1, 040.44-1, 568.74)	-0.36(-0.88, 0.17)	0.04(-0.17, 0.26)	-0.51(-2.04, 1.04)	-0.65(-1.06, -0.24)
Conflict and terrorism	0.20(0.14-0.30)	0.20(0.15-0.27)	-0.10(-3.44, 3.36)	-0.10(-3.44, 3.36)	-0.10(-3.44, 3.36)	-0.10(-3.44, 3.36)
Congenital birth defects	51.32(38.70-67.20)	45.05(33.66-60.33)	-0.49(-0.74, -0.24)	0.97(0.52, 1.42)	-1.03(-1.60, -0.46)	-1.49(-1.67, -1.31)
Cystic echinococcosis	2.21(1.52-2.94)	0.92(0.56-1.48)	-2.81(-3.07, -2.54)	-5.38(-5.58, -5.18)	-1.34(-2.04, -0.65)	-2.33(-2.52, -2.14)
Cysticercosis	57.86(26.94-107.19)	28.59(11.42-62.28)	-2.18(-2.59, -1.76)	-1.45(-2.01, -0.88)	-3.59(-3.79, -3.39)	-1.17(-2.35, 0.02)
Decubitus ulcer	3.26(1.92-6.10)	5.98(3.33-7.78)	2.78(1.64, 3.93)	7.69(6.68, 8.71)	2.88(0.91, 4.89)	-2.10(-4.63, 0.49)
Dengue	0.04(0.02-0.07)	0.02(0.01-0.05)	-2.02(-2.84, -1.20)	-4.65(-5.79, -3.49)	-1.77(-3.37, -0.14)	-0.26(-0.91, 0.40)
Depressive disorders	813.77(563.84-1, 109.78)	980.95(689.77-1, 343.65)	0.62(0.51, 0.73)	1.71(1.41, 2.02)	0.29(0.25, 0.33)	-0.13(-0.28, 0.01)
Dermatitis	96.71(55.15-164.69)	96.80(54.87-165.44)	0.00(-0.01, 0.01)	-0.01(-0.03, 0.02)	0.01(0.00, 0.02)	-0.01(-0.02, 0.01)
Diabetes mellitus	1, 956.95(1, 621.64-2, 358.63)	2, 142.16(1, 686.61-2, 693.04)	0.26(0.00, 0.51)	1.36(1.25, 1.48)	-0.62(-1.30, 0.08)	-0.03(-0.36, 0.30)
Diarrheal diseases	145.25(73.80-211.61)	15.67(10.70-24.38)	-7.04(-7.38, -6.70)	-8.24(-8.38, -8.11)	-8.09(-9.01, -7.16)	-4.43(-4.79, -4.08)

Dietary iron deficiency	313.40(211.05-454.15)	117.41(74.19-177.87)	-3.17(-3.30, -3.05)	-2.34(-2.68, -2.00)	-4.54(-4.66, -4.41)	-2.48(-2.61, -2.35)
Drowning	206.37(177.28-236.70)	116.64(97.64-138.69)	-1.62(-2.27, -0.96)	-2.55(-3.53, -1.57)	-0.97(-1.87, -0.06)	-1.67(-2.04, -1.30)
Drug use disorders	190.06(159.46-224.15)	56.66(42.49-72.15)	-4.00(-4.42, -3.58)	-3.63(-4.06, -3.19)	-6.49(-6.99, -6.00)	-1.00(-1.99, 0.01)
Encephalitis	17.81(14.26-21.31)	11.88(9.63-14.59)	-1.37(-1.60, -1.14)	-0.83(-1.26, -0.40)	-1.27(-1.57, -0.97)	-2.25(-2.41, -2.09)
Endocarditis	15.70(8.59-20.45)	5.01(3.83-7.39)	-3.62(-3.85, -3.39)	-1.30(-1.62, -0.99)	-7.29(-7.68, -6.91)	-1.28(-1.63, -0.92)
Endocrine, metabolic, blood, and immune disorders	314.75(199.83-492.38)	298.30(187.26-458.36)	-0.12(-1.04, 0.80)	1.25(0.70, 1.81)	-0.89(-3.18, 1.47)	-1.02(-2.64, 0.63)
Environmental heat and cold exposure	47.15(23.71-61.51)	16.27(9.10-23.30)	-3.40(-3.61, -3.19)	-3.31(-3.64, -2.98)	-4.47(-4.96, -3.98)	-2.33(-2.47, -2.20)
Esophageal cancer	3, 172.78(2, 542.15-3, 711.12)	1, 597.51(1, 240.94-1, 971.30)	-2.36(-3.00, -1.71)	-1.57(-2.24, -0.90)	-2.96(-4.64, -1.25)	-2.78(-3.14, -2.42)
Exposure to forces of nature	8.23(7.44-9.05)	3.85(3.21-4.78)	-0.69(-5.08, 3.90)	-0.69(-5.08, 3.90)	-0.69(-5.08, 3.90)	-0.69(-5.08, 3.90)
Exposure to mechanical forces	107.43(83.54-153.73)	103.13(74.57-136.34)	0.05(-0.28, 0.38)	-0.35(-0.81, 0.12)	2.00(1.51, 2.48)	-2.15(-2.69, -1.59)
Eye cancer	4.10(2.51-5.59)	2.62(1.53-3.59)	-1.45(-1.89, -1.00)	-0.92(-1.14, -0.70)	-1.54(-1.88, -1.20)	-1.96(-3.38, -0.51)
Falls	925.53(732.85-1, 145.79)	926.57(696.87-1, 194.38)	0.04(-0.12, 0.21)	-0.17(-0.52, 0.18)	-1.61(-1.89, -1.32)	1.89(1.76, 2.01)
Fire, heat, and hot substances	104.46(82.92-125.57)	48.50(36.66-63.13)	-2.51(-2.68, -2.33)	-2.88(-3.31, -2.45)	-2.33(-2.49, -2.17)	-2.33(-2.49, -2.17)
Food-borne trematodiases	159.07(64.76-322.66)	77.08(37.85-139.76)	-2.43(-3.28, -1.57)	-3.82(-4.93, -2.71)	-2.40(-2.71, -2.09)	-2.18(-4.01, -0.31)
Foreign body	49.86(39.15-64.33)	40.47(27.65-53.40)	-0.75(-1.05, -0.44)	-0.83(-1.07, -0.58)	-2.07(-2.91, -1.22)	0.68(0.34, 1.03)
Fungal skin diseases	34.57(13.54-75.08)	33.84(13.42-73.81)	-0.07(-0.08, -0.06)	-0.04(-0.05, -0.03)	-0.09(-0.09, -0.09)	-0.08(-0.10, -0.06)
Gallbladder and biliary diseases	445.22(305.97-618.37)	311.57(205.13-452.28)	-1.18(-1.54, -0.82)	-1.44(-1.55, -1.33)	-0.63(-1.58, 0.33)	-1.34(-1.88, -0.79)
Gallbladder and biliary tract cancer	236.35(179.14-315.42)	183.73(126.42-242.71)	-0.75(-1.45, -0.05)	-0.61(-0.77, -0.44)	-0.61(-0.77, -0.44)	-1.10(-3.45, 1.31)
Gout	70.21(41.66-108.54)	91.46(53.90-140.12)	0.90(0.80, 1.00)	-0.69(-0.94, -0.43)	2.34(2.22, 2.46)	0.93(0.82, 1.05)
Gynecological diseases	177.63(113.68-269.85)	115.30(73.04-169.06)	-1.34(-1.49, -1.20)	-0.30(-0.34, -0.25)	-3.92(-4.29, -3.55)	0.26(0.16, 0.36)
Headache disorders	464.07(144.16-930.02)	493.65(144.13-1, 018.03)	0.20(0.14, 0.25)	-0.12(-0.15, -0.08)	0.55(0.40, 0.71)	0.19(0.13, 0.25)

Hemoglobinopathies and hemolytic anemias	187.27(142.44-244.69)	83.29(64.38-110.44)	-2.60(-2.68, -2.53)	-2.26(-2.35, -2.18)	-3.08(-3.13, -3.03)	-2.43(-2.66, -2.20)
HIV/AIDS	8.29(0.96-13.13)	96.34(67.76-129.56)	7.43(6.05, 8.83)	13.47(12.09, 14.86)	0.48(-0.58, 1.55)	9.21(5.01, 13.58)
Hodgkin lymphoma	40.74(15.66-57.44)	11.17(6.49-14.67)	-4.15(-4.46, -3.84)	-4.03(-4.34, -3.72)	-5.81(-6.44, -5.17)	-2.46(-3.07, -1.84)
Hypertensive heart disease	2, 667.27(1, 623.34-3, 206.48)	877.99(635.84-1, 143.28)	-3.57(-3.71, -3.43)	-4.78(-4.98, -4.58)	-3.91(-4.25, -3.57)	-1.64(-1.84, -1.43)
Idiopathic developmental intellectual disability	8.23(2.14-17.08)	5.87(1.51-12.55)	-1.11(-1.21, -1.01)	0.18(-0.09, 0.45)	-1.90(-1.97, -1.84)	-1.47(-1.60, -1.34)
Idiopathic epilepsy	111.78(76.44-160.99)	84.59(51.32-138.45)	-0.87(-1.18, -0.55)	-0.04(-0.28, 0.20)	-2.23(-2.37, -2.10)	-0.18(-1.21, 0.85)
Inflammatory bowel disease	32.98(20.44-42.74)	17.29(13.04-22.94)	-2.14(-2.47, -1.80)	-1.22(-1.61, -0.83)	-2.74(-3.61, -1.85)	-2.63(-2.77, -2.49)
Inguinal, femoral, and abdominal hernia	22.22(14.79-32.11)	14.40(8.51-22.80)	-1.41(-1.82, -1.00)	-1.12(-1.24, -1.00)	-1.12(-1.24, -1.00)	-2.13(-3.50, -0.74)
Interpersonal violence	187.37(156.15-224.58)	86.36(69.06-106.92)	-2.47(-2.59, -2.34)	-1.91(-2.10, -1.72)	-3.02(-3.10, -2.94)	-2.38(-2.70, -2.06)
Interstitial lung disease and pulmonary sarcoidosis	48.38(36.01-69.95)	46.65(33.84-62.71)	-0.14(-0.29, 0.00)	-0.38(-0.58, -0.19)	1.52(1.16, 1.88)	-1.57(-1.70, -1.45)
Intestinal nematode infections	67.99(34.98-109.00)	1.29(0.63-2.15)	-12.12(-12.49, -11.75)	-12.43(-13.02, -11.83)	-15.98(-16.59, -15.38)	-5.96(-6.57, -5.35)
Invasive Non-typhoidal Salmonella (iNTS)	3.50(0.92-7.65)	1.85(0.48-4.19)	-2.01(-2.12, -1.90)	-1.49(-1.69, -1.28)	-2.58(-2.74, -2.43)	-1.82(-2.07, -1.57)
Iodine deficiency	19.30(10.34-34.64)	20.12(9.45-38.67)	0.14(0.04, 0.23)	-0.20(-0.30, -0.10)	1.62(1.44, 1.80)	-1.66(-1.80, -1.51)
Ischemic heart disease	6, 283.76(5, 495.98-7, 201.08)	5, 696.24(4, 715.20-6, 762.95)	-0.22(-0.77, 0.34)	0.08(-0.95, 1.12)	-0.10(-0.70, 0.50)	-0.98(-1.35, -0.62)
Kidney cancer	87.37(74.88-99.38)	112.21(90.81-135.44)	0.76(0.24, 1.29)	0.60(-0.11, 1.32)	0.67(0.32, 1.02)	0.69(-0.70, 2.10)
Larynx cancer	194.38(154.78-233.26)	113.05(85.28-147.26)	-1.80(-2.05, -1.55)	-1.97(-2.09, -1.86)	-1.56(-2.28, -0.84)	-1.85(-2.11, -1.59)
Leishmaniasis	1.10(0.06-3.38)	0.25(0.04-0.65)	-4.72(-5.23, -4.21)	-6.38(-7.01, -5.76)	-1.04(-1.83, -0.24)	-6.92(-7.76, -6.07)
Leprosy	0.23(0.14-0.35)	0.06(0.04-0.10)	-4.09(-4.19, -3.99)	-3.49(-3.58, -3.39)	-5.81(-5.94, -5.67)	-2.92(-3.22, -2.63)
Leukemia	310.73(251.50-387.84)	223.96(161.49-284.61)	-1.01(-1.30, -0.71)	-0.75(-1.07, -0.43)	-1.42(-2.21, -0.63)	-0.96(-1.11, -0.80)
Lip and oral cavity cancer	124.93(104.41-145.83)	118.89(90.94-153.22)	-0.27(-0.65, 0.11)	-1.05(-1.17, -0.93)	1.23(0.89, 1.58)	-1.38(-2.57, -0.18)

Liver cancer	1, 052.53(887.52-1, 239.86)	827.73(660.27-1, 029.90)	-0.67(-1.12, -0.22)	0.69(0.24, 1.14)	-1.83(-2.51, -1.14)	-1.31(-2.45, -0.15)
Low back pain	1, 974.90(1, 186.55-2, 979.40)	1, 575.52(952.93-2, 350.20)	-0.72(-0.75, -0.70)	-1.45(-1.53, -1.36)	-0.38(-0.39, -0.37)	-0.38(-0.39, -0.37)
Lower extremity peripheral arterial disease	45.87(23.88-90.17)	40.95(21.60-79.62)	-0.37(-0.47, -0.28)	-0.03(-0.16, 0.11)	-0.51(-0.57, -0.45)	-0.57(-0.86, -0.29)
Lower respiratory infections	1, 093.14(878.70-1, 274.37)	326.15(262.82-408.93)	-3.95(-4.28, -3.62)	-3.21(-3.63, -2.79)	-5.34(-6.18, -4.50)	-3.19(-3.39, -3.00)
Malignant neoplasm of bone and articular cartilage	52.31(31.75-94.29)	89.39(52.26-123.43)	1.81(1.38, 2.24)	3.12(1.85, 4.40)	2.97(2.73, 3.22)	-1.37(-1.51, -1.23)
Malignant skin melanoma	21.28(14.70-27.98)	18.84(9.52-25.04)	-0.60(-1.41, 0.22)	-0.02(-0.57, 0.53)	-0.65(-2.98, 1.74)	-0.98(-1.55, -0.40)
Maternal disorders	0.00(0.00-0.00)	0.00(0.00-0.00)	-3.39(-6.87, 0.23)	-4.77(-13.08, 4.32)	1.58(-3.54, 6.98)	-8.72(-10.20, -7.23)
Meningitis	65.19(55.71-75.55)	15.87(12.87-19.04)	-4.48(-4.95, -4.00)	-5.37(-6.14, -4.60)	-4.73(-5.51, -3.94)	-3.33(-4.18, -2.48)
Mesothelioma	11.51(9.47-14.26)	11.49(9.12-14.16)	-0.02(-0.24, 0.21)	-0.19(-0.45, 0.08)	1.43(1.25, 1.62)	-1.33(-1.95, -0.70)
Motor neuron disease	11.81(6.66-15.49)	18.18(12.01-23.56)	1.27(0.62, 1.91)	3.37(2.91, 3.83)	-1.65(-3.20, -0.08)	3.21(2.98, 3.43)
Multiple myeloma	23.29(15.35-47.88)	74.04(45.45-99.04)	3.96(3.06, 4.87)	10.67(9.56, 11.79)	0.58(-1.78, 3.00)	1.55(0.96, 2.13)
Multiple sclerosis	0.76(0.52-1.08)	1.31(0.93-1.80)	1.71(1.53, 1.88)	1.61(1.53, 1.69)	1.62(1.17, 2.08)	2.11(1.84, 2.37)
Nasopharynx cancer	368.16(305.21-430.89)	146.20(114.45-183.36)	-3.17(-3.42, -2.91)	-2.63(-3.09, -2.16)	-4.69(-5.14, -4.24)	-1.81(-2.13, -1.49)
Neck pain	494.56(260.20-796.31)	558.55(296.73-887.85)	0.41(0.35, 0.48)	0.14(0.11, 0.17)	1.07(0.88, 1.27)	0.06(0.02, 0.10)
Neonatal disorders	39.27(27.31-57.55)	80.54(59.14-102.09)	2.36(2.30, 2.43)	1.90(1.81, 1.99)	2.95(2.92, 2.99)	2.10(1.93, 2.27)
Neuroblastoma and other peripheral nervous cell tumors	1.01(0.70-1.48)	3.79(2.53-4.90)	4.43(4.24, 4.63)	2.48(2.21, 2.75)	7.70(7.21, 8.19)	3.17(2.94, 3.40)
Non-Hodgkin lymphoma	238.46(203.51-296.99)	216.17(164.79-265.23)	-0.35(-0.57, -0.13)	-0.54(-0.76, -0.31)	0.66(0.08, 1.25)	-0.94(-1.21, -0.68)
Non-melanoma skin cancer	73.02(58.24-94.46)	76.38(58.81-96.67)	0.04(-0.41, 0.49)	-0.24(-0.68, 0.20)	1.93(0.67, 3.22)	-1.62(-2.01, -1.23)
Non-rheumatic valvular heart disease	22.84(16.77-30.08)	15.09(10.93-21.19)	-1.32(-1.49, -1.14)	-0.42(-0.50, -0.34)	-2.42(-2.90, -1.95)	-1.08(-1.29, -0.87)
Oral disorders	779.75(460.16-1, 211.74)	706.57(430.43-1, 073.62)	-0.21(-0.55, 0.13)	-2.74(-3.16, -2.33)	0.42(-0.19, 1.04)	1.90(1.28, 2.53)

Osteoarthritis	912.85(436.85-1, 831.59)	1, 076.10(514.46-2, 183.72)	0.60(0.47, 0.74)	-0.27(-0.41, -0.12)	0.93(0.64, 1.23)	1.06(0.88, 1.24)
Other cardiovascular and circulatory diseases	108.05(74.38-154.15)	94.69(58.37-149.07)	-0.42(-0.56, -0.28)	-0.30(-0.41, -0.20)	-0.53(-0.92, -0.13)	-0.43(-0.56, -0.30)
Other chronic respiratory diseases	11.34(8.51-18.48)	14.37(10.79-18.20)	0.86(0.57, 1.14)	2.18(1.90, 2.46)	2.67(2.18, 3.15)	-2.61(-2.98, -2.24)
Other digestive diseases	150.06(104.25-184.59)	42.56(34.02-53.03)	-4.14(-4.50, -3.77)	-4.54(-5.04, -4.05)	-5.00(-5.95, -4.03)	-2.53(-2.80, -2.25)
Other intestinal infectious diseases	0.67(0.03-1.75)	0.20(0.01-0.53)	-3.90(-4.23, -3.57)	-3.74(-4.07, -3.41)	-4.51(-5.38, -3.64)	-3.67(-3.82, -3.52)
Other malignant neoplasms	315.21(213.82-397.79)	187.12(141.48-240.46)	-1.72(-1.83, -1.61)	-2.50(-2.64, -2.37)	-1.36(-1.64, -1.07)	-1.06(-1.18, -0.93)
Other mental disorders	194.98(124.94-290.78)	194.82(126.02-292.15)	-0.01(-0.02, 0.01)	0.06(0.04, 0.08)	0.00(-0.01, 0.01)	-0.08(-0.09, -0.06)
Other musculoskeletal disorders	776.78(470.75-1, 238.94)	923.19(565.28-1, 464.79)	0.55(0.50, 0.59)	0.65(0.54, 0.76)	0.75(0.68, 0.82)	0.16(0.13, 0.19)
Other neglected tropical diseases	15.74(11.71-20.83)	7.20(5.46-9.62)	-2.58(-2.76, -2.40)	-2.33(-2.61, -2.05)	-2.92(-3.18, -2.66)	-2.61(-2.86, -2.36)
Other neoplasms	23.02(15.09-38.35)	34.96(23.34-55.01)	1.38(1.31, 1.46)	1.14(1.08, 1.20)	2.85(2.64, 3.05)	0.20(0.12, 0.29)
Other neurological disorders	21.62(15.54-28.88)	62.09(48.56-77.52)	3.50(3.10, 3.89)	4.75(4.53, 4.97)	2.93(1.93, 3.95)	2.62(1.96, 3.29)
Other nutritional deficiencies	9.19(7.17-12.06)	4.36(3.29-5.68)	-2.15(-2.77, -1.53)	-5.00(-6.03, -3.95)	0.44(-0.52, 1.42)	-2.84(-3.23, -2.45)
Other pharynx cancer	56.76(45.49-71.54)	29.55(22.64-38.39)	-2.25(-2.55, -1.95)	-2.48(-3.09, -1.87)	-3.48(-3.85, -3.12)	-0.32(-0.63, 0.00)
Other sense organ diseases	156.78(74.58-294.47)	162.78(78.60-306.01)	0.12(0.08, 0.16)	0.12(0.10, 0.13)	0.17(0.05, 0.29)	0.07(0.04, 0.09)
Other skin and subcutaneous diseases	75.37(37.69-135.16)	99.80(50.49-180.31)	0.92(0.90, 0.94)	0.77(0.73, 0.81)	1.08(1.06, 1.10)	0.86(0.84, 0.89)
Other transport injuries	65.50(54.25-77.52)	33.01(26.98-39.90)	-2.06(-2.61, -1.51)	-0.56(-0.98, -0.14)	-2.26(-2.91, -1.60)	-3.26(-4.71, -1.79)
Other unintentional injuries	142.89(106.61-185.28)	67.26(48.66-91.52)	-2.35(-2.56, -2.13)	-2.23(-2.45, -2.00)	-2.51(-2.67, -2.34)	-2.43(-3.04, -1.82)
Other unspecified infectious diseases	31.17(17.63-41.67)	11.17(7.62-16.28)	-3.36(-3.72, -3.01)	-3.45(-3.71, -3.19)	-5.15(-6.04, -4.26)	-1.25(-1.70, -0.79)
Otitis media	23.17(12.32-41.56)	14.79(7.51-25.80)	-1.46(-1.50, -1.42)	-1.37(-1.40, -1.34)	-1.56(-1.60, -1.51)	-1.43(-1.53, -1.34)
Ovarian cancer	131.47(103.80-166.35)	128.76(91.88-168.14)	-0.02(-0.47, 0.43)	0.76(0.31, 1.20)	-1.90(-2.65, -1.15)	1.78(0.85, 2.72)
Pancreatic cancer	523.40(440.43-611.04)	634.25(492.50-791.95)	0.57(-0.02, 1.17)	0.94(0.69, 1.20)	0.77(-0.97, 2.53)	-0.03(-0.51, 0.45)
Pancreatitis	79.95(59.73-104.19)	49.45(38.43-66.73)	-1.58(-1.70, -1.47)	-1.27(-1.38, -1.16)	-1.36(-1.46, -1.26)	-2.13(-2.41, -1.86)

Paralytic ileus and intestinal obstruction	106.43(85.15-134.97)	40.80(31.70-59.31)	-3.03(-3.28, -2.78)	-2.20(-2.37, -2.04)	-3.60(-3.72, -3.48)	-3.31(-4.10, -2.51)
Parkinson's disease	342.39(289.03-393.59)	360.30(292.80-431.26)	0.13(-0.16, 0.43)	1.02(0.91, 1.12)	-0.35(-1.22, 0.54)	-0.36(-0.54, -0.17)
Pneumoconiosis	93.40(73.23-116.47)	36.59(27.36-48.33)	-2.99(-3.29, -2.68)	-2.72(-2.99, -2.46)	-3.70(-4.52, -2.88)	-2.70(-2.86, -2.53)
Poisonings	76.90(56.63-138.97)	66.66(35.10-87.12)	-0.32(-0.74, 0.11)	-0.43(-1.17, 0.31)	2.51(1.90, 3.14)	-4.14(-4.79, -3.50)
Police conflict and executions	2.06(0.46-3.51)	5.74(1.71-10.00)	3.28(2.98, 3.57)	2.44(1.56, 3.33)	3.68(3.58, 3.77)	3.68(3.58, 3.77)
Prostate cancer	135.36(98.91-176.84)	132.93(92.60-183.85)	-0.07(-0.73, 0.61)	1.21(0.75, 1.68)	-1.07(-1.42, -0.72)	-0.13(-2.31, 2.11)
Protein-energy malnutrition	64.43(54.37-74.68)	11.14(8.86-14.01)	-5.70(-6.06, -5.34)	-7.94(-8.48, -7.40)	-6.15(-6.42, -5.87)	-2.72(-3.64, -1.79)
Pruritus	21.07(9.43-43.79)	24.58(10.93-51.08)	0.49(0.48, 0.51)	0.32(0.31, 0.34)	0.64(0.61, 0.66)	0.49(0.48, 0.51)
Psoriasis	61.56(43.37-80.36)	81.87(58.62-106.85)	0.94(0.89, 0.99)	1.40(1.25, 1.54)	0.81(0.77, 0.86)	0.63(0.58, 0.67)
Pulmonary Arterial Hypertension	32.73(24.44-44.48)	21.46(14.31-27.80)	-1.38(-1.70, -1.07)	-2.38(-2.56, -2.19)	2.51(1.57, 3.45)	-4.06(-4.30, -3.83)
Rabies	3.39(1.58-5.35)	1.77(0.75-2.93)	-2.18(-3.42, -0.93)	-4.80(-6.39, -3.19)	6.11(4.95, 7.29)	-9.56(-12.59, -6.43)
Rheumatic heart disease	1, 432.84(1, 157.07-1, 697.96)	304.90(238.77-385.80)	-5.16(-5.65, -4.67)	-5.02(-5.48, -4.55)	-5.48(-6.77, -4.16)	-5.23(-5.51, -4.95)
Rheumatoid arthritis	142.53(111.97-183.76)	134.03(101.52-172.86)	-0.19(-0.38, 0.01)	-0.24(-0.42, -0.06)	0.02(-0.51, 0.54)	-0.58(-0.67, -0.49)
Road injuries	1, 041.16(896.51-1, 196.27)	924.38(783.67-1, 080.91)	-0.38(-0.73, -0.02)	0.88(0.24, 1.52)	0.81(0.33, 1.29)	-3.51(-4.09, -2.94)
Scabies	80.37(40.47-142.24)	80.18(40.90-141.38)	-0.01(-0.03, 0.01)	0.01(0.00, 0.02)	-0.03(-0.05, -0.02)	0.00(-0.05, 0.05)
Schistosomiasis	31.82(23.66-42.73)	5.86(3.47-10.17)	-5.48(-5.84, -5.11)	-7.11(-7.33, -6.88)	-5.81(-6.82, -4.80)	-3.50(-3.72, -3.28)
Schizophrenia	199.54(146.09-249.38)	205.54(151.58-262.98)	0.10(0.07, 0.13)	0.01(-0.04, 0.05)	-0.05(-0.08, -0.02)	0.40(0.34, 0.46)
Self-harm	1, 189.05(836.44-1, 378.57)	426.68(348.40-528.27)	-3.30(-3.59, -3.01)	-1.72(-1.88, -1.56)	-5.14(-5.89, -4.39)	-3.02(-3.44, -2.60)
Sexually transmitted infections excluding HIV	13.73(8.56-21.31)	8.61(4.58-15.51)	-1.47(-1.90, -1.04)	-2.65(-3.87, -1.41)	-1.38(-1.60, -1.15)	-0.38(-0.72, -0.04)
Soft tissue and other extraosseous sarcomas	37.20(24.44-48.03)	20.07(13.47-28.96)	-2.09(-2.51, -1.67)	-1.43(-1.79, -1.06)	-2.90(-4.04, -1.74)	-2.12(-2.32, -1.91)
Stomach cancer	5, 260.21(4, 354.24-6, 262.42)	2, 256.83(1, 719.78-2, 843.85)	-2.75(-3.21, -2.28)	-1.99(-2.41, -1.57)	-3.36(-4.62, -2.09)	-3.16(-3.42, -2.89)

Stroke	19, 424.31(16, 983.04-22, 105.58)	9, 811.40(8, 227.46-11, 575.47)	-2.22(-2.55, -1.90)	-1.35(-1.71, -1.00)	-2.99(-3.82, -2.16)	-2.55(-2.76, -2.34)
Testicular cancer	5.28(4.09-6.73)	4.07(3.01-5.57)	-0.97(-1.35, -0.58)	-0.30(-1.15, 0.57)	-2.60(-3.00, -2.20)	0.39(-0.07, 0.85)
Tetanus	26.47(11.78-38.95)	0.88(0.26-2.11)	-10.61(-11.28, -9.93)	-10.65(-12.25, -9.02)	-10.36(-11.03, -9.68)	-11.16(-11.47, -10.84)
Thyroid cancer	40.58(32.40-49.21)	30.15(23.89-37.70)	-1.04(-1.37, -0.71)	-1.32(-1.44, -1.20)	-1.32(-1.44, -1.20)	-0.35(-1.43, 0.74)
Tracheal, bronchus, and lung cancer	4, 233.86(3, 570.71-4, 943.42)	4, 417.16(3, 514.75-5, 407.03)	0.17(-0.25, 0.59)	1.33(1.11, 1.54)	0.19(-0.99, 1.39)	-1.11(-1.58, -0.63)
Trachoma	3.52(1.42-7.05)	0.37(0.13-0.77)	-6.96(-7.63, -6.28)	-3.64(-4.89, -2.38)	-10.31(-11.83, -8.77)	-7.57(-7.89, -7.25)
Tuberculosis	1, 858.50(1, 533.74-2, 258.65)	232.38(180.99-294.63)	-6.68(-6.96, -6.40)	-6.13(-6.53, -5.73)	-8.23(-8.75, -7.70)	-6.00(-6.40, -5.61)
Typhoid and paratyphoid	1.33(0.53-2.69)	0.41(0.17-0.78)	-3.65(-3.99, -3.30)	-4.09(-4.22, -3.95)	-2.85(-3.87, -1.82)	-3.96(-4.20, -3.71)
Upper digestive system diseases	1, 039.74(884.57-1, 215.96)	350.77(274.42-457.38)	-3.51(-3.70, -3.33)	-3.73(-3.86, -3.60)	-3.63(-4.14, -3.12)	-3.55(-3.65, -3.44)
Upper respiratory infections	97.62(40.17-142.19)	32.69(19.43-52.65)	-3.52(-3.63, -3.40)	-3.85(-4.00, -3.70)	-5.04(-5.23, -4.84)	-1.16(-1.35, -0.98)
Urinary diseases and male infertility	287.45(204.04-404.04)	183.54(121.27-272.58)	-1.50(-1.70, -1.29)	-1.69(-1.98, -1.39)	-1.66(-2.16, -1.16)	-1.30(-1.45, -1.14)
Urticaria	36.34(21.86-56.12)	36.35(21.67-55.65)	0.00(-0.01, 0.01)	-0.05(-0.06, -0.04)	0.03(0.01, 0.06)	0.02(0.01, 0.04)
Uterine cancer	132.65(96.63-169.47)	76.05(55.06-103.41)	-1.70(-2.14, -1.26)	-0.40(-0.66, -0.14)	-2.57(-3.17, -1.97)	-1.69(-2.77, -0.60)
Varicella and herpes zoster	18.62(13.81-24.38)	9.22(5.28-14.40)	-2.23(-2.39, -2.08)	-2.75(-2.90, -2.60)	-3.90(-4.23, -3.58)	-0.14(-0.36, 0.08)
Vascular intestinal disorders	6.70(5.26-7.99)	2.74(2.21-3.41)	-2.82(-3.14, -2.51)	-0.66(-0.87, -0.44)	-5.36(-6.05, -4.67)	-2.28(-2.79, -1.76)
Viral skin diseases	47.42(30.20-70.46)	47.62(29.98-70.68)	0.01(0.00, 0.02)	0.13(0.10, 0.15)	0.00(-0.01, 0.02)	-0.11(-0.12, -0.09)
Vitamin A deficiency	1.68(0.87-2.86)	1.67(0.82-2.80)	0.15(-0.07, 0.38)	0.78(0.17, 1.39)	-0.28(-0.39, -0.17)	-0.28(-0.39, -0.17)
**70-74 years**						
Acne vulgaris	5.12(3.26-7.85)	6.11(3.83-9.45)	0.58(0.55, 0.60)	0.65(0.63, 0.67)	0.59(0.52, 0.65)	0.50(0.49, 0.52)
Acute glomerulonephritis	86.91(63.04-117.18)	27.01(17.66-36.78)	-3.72(-4.15, -3.29)	-4.25(-4.96, -3.55)	-2.58(-2.82, -2.33)	-4.64(-5.78, -3.49)
Acute hepatitis	213.49(168.99-267.79)	15.65(12.21-20.30)	-7.93(-8.75, -7.10)	-8.57(-9.19, -7.95)	-11.58(-13.88, -9.23)	-3.38(-3.87, -2.89)
Adverse effects of medical treatment	62.36(41.49-79.35)	15.99(12.54-22.19)	-4.32(-4.67, -3.97)	-4.27(-4.45, -4.09)	-6.41(-7.22, -5.59)	-1.98(-2.41, -1.56)

Age-related and other hearing loss	2, 628.44(1, 725.65-3, 837.09)	2, 964.21(1, 987.12-4, 138.63)	0.44(0.34, 0.54)	-0.04(-0.16, 0.09)	0.50(0.30, 0.69)	0.88(0.74, 1.02)
Alcohol use disorders	111.53(83.78-151.18)	122.27(85.99-168.82)	0.25(-0.07, 0.57)	0.29(-0.52, 1.10)	2.14(1.92, 2.37)	-2.26(-2.78, -1.74)
Alopecia areata	6.86(4.47-10.19)	6.75(4.46-9.78)	-0.06(-0.08, -0.03)	-0.14(-0.17, -0.12)	-0.06(-0.09, -0.02)	0.03(-0.03, 0.09)
Alzheimer's disease and other dementias	2, 240.82(1, 042.43-4, 972.24)	2, 428.04(1, 229.64-5, 195.87)	0.15(0.01, 0.30)	0.00(-0.04, 0.03)	0.00(-0.04, 0.03)	0.54(0.06, 1.02)
Animal contact	47.83(36.77-55.45)	11.77(9.52-14.51)	-4.45(-4.63, -4.26)	-3.41(-3.84, -2.99)	-6.96(-7.18, -6.74)	-2.92(-3.18, -2.66)
Anxiety disorders	514.65(345.01-733.48)	518.99(350.19-733.76)	-0.05(-0.39, 0.28)	0.31(-0.04, 0.66)	-1.01(-1.29, -0.72)	1.11(0.28, 1.96)
Aortic aneurysm	32.98(26.26-41.86)	43.58(34.20-55.40)	0.84(0.74, 0.95)	0.47(0.40, 0.54)	1.95(1.81, 2.08)	0.34(0.12, 0.55)
Appendicitis	44.01(31.69-53.86)	8.72(6.75-11.35)	-5.18(-5.40, -4.97)	-4.04(-4.46, -3.63)	-6.99(-7.36, -6.63)	-4.72(-5.01, -4.44)
Asthma	822.18(644.12-1, 094.95)	201.15(157.27-255.42)	-4.49(-4.63, -4.36)	-3.48(-3.56, -3.40)	-6.02(-6.37, -5.67)	-3.90(-4.06, -3.73)
Atrial fibrillation and flutter	401.11(299.68-527.15)	402.16(300.25-527.56)	-0.01(-0.12, 0.09)	-0.64(-0.75, -0.53)	-0.02(-0.23, 0.18)	0.60(0.46, 0.75)
Attention-deficit/hyperactivity disorder	0.74(0.25-1.61)	0.65(0.20-1.43)	-0.39(-0.53, -0.26)	-1.10(-1.26, -0.93)	-0.26(-0.63, 0.12)	0.15(0.07, 0.23)
Autism spectrum disorders	72.33(49.51-102.09)	88.45(59.86-123.30)	0.65(0.61, 0.69)	0.81(0.74, 0.89)	0.74(0.67, 0.80)	0.35(0.31, 0.38)
Bacterial skin diseases	54.97(42.19-65.97)	10.65(8.00-15.29)	-5.11(-5.47, -4.76)	-3.28(-3.58, -2.99)	-11.52(-12.22, -10.81)	-0.37(-1.19, 0.46)
Bipolar disorder	43.17(26.80-63.89)	43.42(27.77-65.29)	0.02(-0.01, 0.05)	0.03(0.01, 0.05)	-0.07(-0.15, 0.02)	0.10(0.07, 0.13)
Bladder cancer	433.37(320.82-509.29)	291.01(227.35-377.32)	-1.30(-1.43, -1.16)	-0.90(-1.03, -0.76)	-2.50(-2.76, -2.23)	-0.40(-0.68, -0.11)
Blindness and vision loss	1, 507.98(1, 039.94-2, 112.20)	1, 377.51(890.76-2, 110.46)	-0.25(-0.44, -0.07)	1.16(0.61, 1.72)	-0.92(-1.00, -0.84)	-0.92(-1.00, -0.84)
Brain and central nervous system cancer	294.63(230.30-375.88)	326.35(244.23-420.38)	0.36(0.28, 0.44)	1.07(0.90, 1.25)	-0.24(-0.34, -0.15)	0.26(0.13, 0.39)
Breast cancer	360.17(300.26-427.61)	386.31(312.43-470.16)	0.24(0.05, 0.44)	0.23(-0.09, 0.55)	-0.20(-0.30, -0.11)	0.86(0.33, 1.39)
Cardiomyopathy and myocarditis	98.96(80.42-157.35)	107.25(85.23-136.33)	0.58(0.23, 0.93)	4.30(4.01, 4.60)	-0.24(-0.77, 0.29)	-2.45(-3.36, -1.54)
Cervical cancer	316.13(261.46-390.89)	214.63(161.78-274.64)	-1.11(-1.44, -0.78)	-3.00(-3.36, -2.64)	-0.97(-1.72, -0.21)	0.36(0.04, 0.68)
Chronic kidney disease	1, 780.55(1, 526.46-2, 122.70)	1, 476.03(1, 211.53-1, 749.24)	-0.59(-0.74, -0.44)	-0.73(-0.87, -0.58)	-0.43(-0.84, -0.02)	-0.92(-0.99, -0.84)
Chronic obstructive pulmonary disease	26, 327.56(22, 670.65-29, 531.33)	7, 460.52(6, 175.95-8, 990.80)	-4.16(-4.47, -3.85)	-3.46(-3.63, -3.30)	-5.66(-6.38, -4.93)	-3.26(-3.85, -2.67)

Cirrhosis and other chronic liver diseases	1, 815.74(1, 552.60-2, 121.96)	718.37(572.59-882.31)	-3.05(-3.24, -2.86)	-2.53(-2.77, -2.28)	-3.64(-3.93, -3.35)	-3.03(-3.37, -2.68)
Colon and rectum cancer	1, 733.33(1, 492.50-1, 987.26)	1, 710.03(1, 363.96-2, 076.71)	-0.02(-0.18, 0.15)	0.21(-0.04, 0.46)	-0.54(-0.71, -0.38)	0.34(-0.03, 0.70)
Conflict and terrorism	0.16(0.10-0.23)	0.14(0.10-0.19)	-0.31(-14.33, 16.00)	16.28(5.31, 28.39)	-5.13(-38.95, 47.42)	-13.71(-23.29, -2.94)
Congenital birth defects	29.58(19.72-42.63)	28.98(19.31-41.60)	-0.11(-0.35, 0.14)	1.74(1.29, 2.19)	-0.86(-1.41, -0.31)	-1.27(-1.47, -1.07)
Cystic echinococcosis	2.73(1.80-3.73)	0.99(0.59-1.63)	-3.22(-3.41, -3.03)	-5.10(-5.28, -4.92)	-2.66(-3.09, -2.23)	-2.40(-2.65, -2.15)
Cysticercosis	67.36(28.38-117.77)	33.14(13.44-66.06)	-2.19(-2.61, -1.77)	-1.72(-2.08, -1.35)	-3.78(-3.98, -3.58)	-0.80(-2.14, 0.57)
Decubitus ulcer	5.33(3.18-11.65)	11.45(6.21-14.82)	3.41(2.32, 4.50)	9.79(8.55, 11.05)	2.44(0.50, 4.42)	-1.84(-3.84, 0.20)
Dengue	0.14(0.07-0.23)	0.05(0.02-0.08)	-3.53(-4.52, -2.52)	-8.32(-10.93, -5.64)	-0.49(-1.10, 0.13)	-2.80(-3.10, -2.50)
Depressive disorders	820.66(568.20-1, 103.39)	981.72(681.69-1, 334.96)	0.59(0.49, 0.69)	1.56(1.28, 1.84)	0.35(0.33, 0.38)	-0.16(-0.30, -0.02)
Dermatitis	100.14(57.11-173.23)	99.92(57.11-172.42)	-0.01(-0.01, 0.00)	0.00(-0.02, 0.02)	-0.03(-0.03, -0.02)	0.01(0.00, 0.02)
Diabetes mellitus	2, 278.83(1, 934.70-2, 745.52)	2, 576.45(2, 100.77-3, 155.17)	0.43(0.30, 0.57)	1.83(1.54, 2.13)	-0.45(-0.61, -0.28)	-0.05(-0.27, 0.17)
Diarrheal diseases	221.04(108.81-329.59)	20.68(14.26-33.44)	-7.46(-7.68, -7.24)	-8.53(-8.78, -8.28)	-8.76(-9.05, -8.47)	-4.68(-5.09, -4.28)
Dietary iron deficiency	405.03(274.43-576.86)	156.53(103.36-225.88)	-3.03(-3.14, -2.92)	-2.37(-2.51, -2.22)	-4.12(-4.21, -4.03)	-2.40(-2.70, -2.10)
Drowning	243.90(212.22-278.55)	148.96(123.24-175.61)	-1.40(-1.68, -1.11)	-1.87(-2.23, -1.51)	-1.43(-2.02, -0.83)	-1.27(-1.57, -0.98)
Drug use disorders	157.03(126.98-192.70)	49.23(37.51-64.34)	-3.79(-4.10, -3.48)	-2.88(-3.24, -2.52)	-6.40(-6.79, -6.02)	-1.23(-1.93, -0.53)
Encephalitis	16.38(13.50-19.75)	11.10(9.04-13.19)	-1.29(-1.46, -1.12)	-0.83(-1.11, -0.55)	-1.39(-1.58, -1.20)	-1.75(-2.09, -1.42)
Endocarditis	21.42(11.41-28.02)	6.99(5.32-10.46)	-3.56(-3.76, -3.36)	-1.61(-1.89, -1.32)	-6.93(-7.30, -6.56)	-1.10(-1.40, -0.79)
Endocrine, metabolic, blood, and immune disorders	334.39(216.90-489.93)	311.75(198.62-464.46)	-0.17(-1.04, 0.71)	1.10(0.58, 1.62)	-0.96(-3.10, 1.24)	-0.94(-2.53, 0.68)
Environmental heat and cold exposure	56.66(26.80-73.11)	20.32(10.76-28.19)	-3.25(-3.64, -2.85)	-3.46(-3.93, -2.99)	-4.29(-5.34, -3.22)	-1.84(-2.04, -1.65)
Esophageal cancer	3, 564.81(2, 892.26-4, 132.25)	2, 004.99(1, 601.00-2, 462.56)	-1.75(-2.14, -1.36)	-0.61(-0.79, -0.43)	-3.19(-4.19, -2.18)	-1.34(-1.84, -0.83)
Exposure to forces of nature	9.32(8.40-10.27)	4.13(3.39-5.10)	-0.59(-5.10, 4.14)	-0.59(-5.10, 4.14)	-0.59(-5.10, 4.14)	-0.59(-5.10, 4.14)

Exposure to mechanical forces	96.15(75.21-129.34)	100.17(73.05-133.73)	0.31(0.03, 0.60)	-0.01(-0.37, 0.35)	2.00(1.61, 2.38)	-1.61(-2.21, -1.00)
Eye cancer	4.97(3.09-6.49)	3.58(2.01-4.84)	-1.04(-1.15, -0.92)	-0.83(-1.02, -0.64)	-1.17(-1.28, -1.06)	-1.16(-1.41, -0.92)
Falls	1, 123.87(900.61-1, 383.85)	1, 166.01(867.18-1, 479.39)	0.23(0.05, 0.40)	-0.05(-0.44, 0.34)	-1.49(-1.73, -1.24)	2.19(1.98, 2.40)
Fire, heat,and hot substances	131.49(103.63-155.60)	61.26(46.55-78.33)	-2.49(-2.78, -2.21)	-3.20(-3.58, -2.82)	-1.56(-2.33, -0.78)	-2.57(-2.67, -2.47)
Food-borne trematodiases	149.14(60.94-300.66)	74.50(37.13-134.71)	-2.32(-3.18, -1.45)	-3.66(-4.81, -2.51)	-2.08(-2.39, -1.76)	-2.14(-3.99, -0.26)
Foreign body	57.18(44.66-71.05)	50.23(33.40-63.09)	-0.36(-0.51, -0.21)	-0.55(-0.68, -0.41)	-1.49(-1.76, -1.22)	1.07(0.71, 1.43)
Fungal skin diseases	43.65(18.31-93.09)	42.99(17.95-91.04)	-0.05(-0.06, -0.04)	-0.02(-0.03, -0.01)	-0.07(-0.07, -0.06)	-0.07(-0.09, -0.05)
Gallbladder and biliary diseases	581.04(394.07-764.07)	394.31(263.96-552.93)	-1.28(-1.55, -1.01)	-1.55(-1.63, -1.47)	-0.97(-1.67, -0.27)	-1.17(-1.56, -0.77)
Gallbladder and biliary tract cancer	301.35(235.35-391.97)	244.78(172.75-326.55)	-0.66(-0.76, -0.56)	-0.61(-0.76, -0.46)	-0.86(-0.99, -0.73)	-0.52(-0.73, -0.32)
Gout	77.47(43.71-122.71)	104.05(60.23-163.82)	0.99(0.91, 1.08)	-0.74(-0.95, -0.53)	2.53(2.42, 2.63)	1.03(0.96, 1.11)
Gynecological diseases	166.06(111.57-246.73)	110.83(73.84-161.04)	-1.27(-1.44, -1.10)	-0.44(-0.49, -0.38)	-3.49(-3.92, -3.06)	0.18(0.06, 0.30)
Headache disorders	386.38(115.14-806.34)	407.04(114.33-849.97)	0.15(0.10, 0.20)	-0.09(-0.13, -0.06)	0.50(0.34, 0.67)	0.07(0.05, 0.09)
Hemoglobinopathies and hemolytic anemias	230.41(172.81-304.67)	108.24(83.86-138.92)	-2.41(-2.46, -2.35)	-2.17(-2.22, -2.11)	-2.80(-2.82, -2.77)	-2.22(-2.39, -2.04)
HIV/AIDS	5.79(0.92-9.24)	78.27(53.78-109.95)	7.96(6.69, 9.24)	16.02(14.94, 17.11)	0.80(-0.62, 2.24)	8.25(4.63, 11.99)
Hodgkin lymphoma	43.38(17.90-59.74)	13.31(8.01-17.68)	-3.75(-3.87, -3.63)	-3.35(-3.48, -3.22)	-5.62(-5.87, -5.37)	-2.11(-2.29, -1.93)
Hypertensive heart disease	4, 572.81(2, 954.23-5, 448.39)	1, 665.55(1, 198.48-2, 136.10)	-3.23(-3.35, -3.11)	-4.65(-4.86, -4.45)	-3.89(-4.12, -3.66)	-0.73(-0.85, -0.62)
Idiopathic developmental intellectual disability	7.35(2.25-14.65)	4.94(1.29-10.76)	-1.31(-1.39, -1.23)	0.25(0.03, 0.47)	-2.33(-2.41, -2.25)	-1.71(-1.79, -1.63)
Idiopathic epilepsy	125.89(85.60-181.03)	95.59(61.16-147.50)	-0.82(-1.09, -0.56)	-0.14(-0.32, 0.05)	-2.24(-2.35, -2.12)	0.11(-0.75, 0.98)
Inflammatory bowel disease	55.70(33.28-72.45)	27.34(20.37-36.50)	-2.30(-2.50, -2.10)	-1.47(-1.85, -1.09)	-3.10(-3.34, -2.87)	-2.33(-2.63, -2.03)
Inguinal,femoral, and abdominal hernia	28.63(20.64-38.75)	17.63(11.24-26.95)	-1.58(-1.80, -1.37)	-1.57(-1.75, -1.40)	-1.53(-1.75, -1.32)	-1.80(-2.41, -1.19)
Interpersonal violence	176.73(146.59-211.26)	85.17(68.53-105.24)	-2.34(-2.42, -2.26)	-1.73(-1.87, -1.59)	-2.96(-3.02, -2.89)	-2.25(-2.45, -2.06)

Interstitial lung disease and pulmonary sarcoidosis	64.44(49.72-103.96)	64.98(45.04-86.78)	-0.02(-0.16, 0.12)	-0.44(-0.62, -0.26)	1.89(1.57, 2.20)	-1.57(-1.68, -1.45)
Intestinal nematode infections	65.48(34.39-103.91)	1.32(0.67-2.24)	-11.99(-12.36, -11.61)	-12.39(-12.97, -11.80)	-15.93(-16.65, -15.20)	-6.46(-6.96, -5.94)
Invasive Non-typhoidal Salmonella (iNTS)	3.22(0.86-7.19)	1.81(0.49-4.15)	-1.82(-1.90, -1.75)	-1.75(-1.85, -1.65)	-2.10(-2.23, -1.96)	-1.57(-1.73, -1.41)
Iodine deficiency	17.91(9.61-31.55)	18.25(8.52-35.33)	0.03(-0.18, 0.24)	-0.72(-0.85, -0.59)	1.80(1.21, 2.39)	-1.70(-1.79, -1.62)
Ischemic heart disease	8, 621.39(7, 506.25-9, 724.35)	9, 134.98(7, 587.48-10, 747.47)	0.15(-0.10, 0.40)	0.67(0.29, 1.05)	0.76(0.45, 1.08)	-1.45(-1.92, -0.97)
Kidney cancer	104.83(90.09-117.93)	135.00(108.99-162.70)	0.79(0.55, 1.02)	0.92(0.56, 1.29)	0.61(0.44, 0.78)	0.60(-0.01, 1.21)
Larynx cancer	217.37(175.70-255.50)	134.41(105.11-170.64)	-1.55(-1.67, -1.42)	-1.91(-2.10, -1.73)	-1.87(-2.13, -1.60)	-0.91(-1.00, -0.81)
Leishmaniasis	0.94(0.06-2.77)	0.23(0.04-0.59)	-4.49(-5.05, -3.92)	-6.66(-7.41, -5.90)	0.88(-0.48, 2.26)	-7.56(-8.06, -7.06)
Leprosy	0.25(0.15-0.38)	0.07(0.04-0.11)	-4.01(-4.20, -3.81)	-3.54(-3.73, -3.36)	-5.30(-5.62, -4.98)	-3.11(-3.60, -2.61)
Leukemia	333.47(272.93-417.02)	256.39(187.33-325.63)	-0.79(-0.94, -0.64)	-0.47(-0.74, -0.19)	-1.38(-1.57, -1.20)	-0.54(-0.77, -0.30)
Lip and oral cavity cancer	142.42(119.90-165.93)	144.46(112.47-180.73)	0.03(-0.10, 0.15)	-0.62(-0.70, -0.55)	1.06(0.75, 1.37)	-0.63(-0.83, -0.42)
Liver cancer	1, 019.20(865.54-1, 188.50)	808.09(650.63-995.70)	-0.65(-0.90, -0.41)	1.16(0.82, 1.50)	-2.49(-3.07, -1.92)	-0.77(-1.00, -0.55)
Low back pain	2, 129.93(1, 347.48-3, 158.03)	1, 622.36(1, 027.56-2, 397.33)	-0.87(-0.92, -0.81)	-1.81(-1.91, -1.71)	-0.31(-0.43, -0.19)	-0.53(-0.59, -0.47)
Lower extremity peripheral arterial disease	72.29(36.42-138.07)	65.51(35.14-122.03)	-0.33(-0.38, -0.27)	0.00(-0.10, 0.10)	-0.60(-0.64, -0.57)	-0.35(-0.47, -0.22)
Lower respiratory infections	2, 036.00(1, 628.53-2, 321.53)	655.19(531.06-818.90)	-3.80(-4.22, -3.38)	-3.08(-3.59, -2.56)	-4.89(-5.99, -3.78)	-3.40(-3.65, -3.15)
Malignant neoplasm of bone and articular cartilage	63.67(40.31-114.19)	108.85(65.68-147.10)	1.79(1.39, 2.18)	3.50(2.31, 4.70)	2.71(2.52, 2.90)	-1.52(-1.65, -1.38)
Malignant skin melanoma	25.31(17.20-36.05)	24.20(12.82-32.06)	-0.17(-0.46, 0.12)	0.10(-0.13, 0.32)	-0.17(-0.98, 0.65)	-0.36(-0.57, -0.14)
Maternal disorders	0.00(0.00-0.00)	0.00(0.00-0.00)	2.53(-2.67, 8.01)	3.99(-5.14, 14.00)	-1.89(-3.55, -0.20)	8.36(-5.06, 23.68)
Meningitis	77.00(66.49-88.70)	19.62(16.04-23.56)	-4.15(-4.90, -3.41)	-5.88(-6.92, -4.82)	-4.41(-5.90, -2.90)	-1.29(-1.72, -0.86)
Mesothelioma	12.91(10.58-15.56)	13.24(10.48-16.20)	0.10(-0.08, 0.27)	-0.27(-0.46, -0.07)	1.98(1.81, 2.15)	-1.43(-1.92, -0.95)

Motor neuron disease	9.38(5.63-12.18)	14.93(9.49-19.92)	1.42(1.10, 1.74)	3.36(3.01, 3.71)	-1.32(-1.92, -0.71)	3.41(2.94, 3.89)
Multiple myeloma	27.05(18.13-56.64)	90.38(59.30-120.07)	4.42(3.65, 5.20)	11.69(9.34, 14.10)	0.49(-0.18, 1.16)	1.99(1.72, 2.26)
Multiple sclerosis	0.70(0.47-0.98)	1.21(0.87-1.66)	1.76(1.62, 1.91)	1.80(1.66, 1.94)	1.90(1.64, 2.16)	1.80(1.47, 2.12)
Nasopharynx cancer	353.22(298.47-407.97)	141.29(114.33-173.43)	-2.98(-3.12, -2.83)	-2.71(-2.89, -2.52)	-4.49(-4.71, -4.27)	-1.23(-1.57, -0.89)
Neck pain	554.24(279.12-926.26)	599.12(306.67-1, 000.10)	0.26(0.11, 0.41)	-0.45(-0.69, -0.21)	1.25(0.87, 1.63)	0.08(-0.03, 0.19)
Neonatal disorders	22.64(16.05-31.91)	46.58(33.91-59.14)	2.37(2.31, 2.43)	1.32(1.19, 1.46)	3.22(3.19, 3.25)	2.55(2.44, 2.66)
Neuroblastoma and other peripheral nervous cell tumors	1.19(0.84-1.75)	4.62(3.15-5.79)	4.55(4.43, 4.67)	2.87(2.74, 3.01)	7.23(6.93, 7.52)	3.16(2.98, 3.33)
Non-Hodgkin lymphoma	268.66(229.69-343.02)	259.57(203.38-315.02)	-0.08(-0.22, 0.06)	-0.30(-0.47, -0.13)	0.22(-0.12, 0.57)	-0.03(-0.20, 0.14)
Non-melanoma skin cancer	93.34(74.38-117.75)	103.62(80.93-128.17)	0.23(0.01, 0.46)	0.01(-0.43, 0.45)	1.72(1.49, 1.94)	-1.46(-1.95, -0.97)
Non-rheumatic valvular heart disease	39.43(29.55-53.77)	29.98(21.46-43.08)	-0.90(-1.02, -0.78)	-0.20(-0.26, -0.14)	-1.48(-1.55, -1.40)	-0.97(-1.36, -0.58)
Oral disorders	972.80(602.57-1, 489.33)	891.29(558.21-1, 371.00)	-0.11(-0.48, 0.26)	-2.34(-2.77, -1.90)	0.44(-0.10, 0.98)	1.98(1.23, 2.73)
Osteoarthritis	984.05(463.60-2, 005.90)	1, 154.12(542.62-2, 347.56)	0.57(0.42, 0.72)	-0.10(-0.28, 0.08)	0.32(-0.03, 0.66)	1.34(1.17, 1.52)
Other cardiovascular and circulatory diseases	168.76(120.46-236.45)	142.28(89.45-222.24)	-0.57(-0.81, -0.32)	-0.25(-0.40, -0.10)	-0.81(-1.53, -0.07)	-0.68(-0.83, -0.52)
Other chronic respiratory diseases	15.54(11.61-25.57)	20.84(15.50-25.95)	0.99(0.80, 1.19)	2.39(2.01, 2.77)	3.68(3.33, 4.03)	-3.10(-3.43, -2.78)
Other digestive diseases	227.39(158.17-279.89)	66.32(53.75-80.99)	-3.92(-4.07, -3.78)	-4.81(-5.08, -4.53)	-4.97(-5.05, -4.88)	-1.62(-1.98, -1.25)
Other intestinal infectious diseases	0.48(0.01-1.32)	0.15(0.01-0.41)	-3.65(-3.88, -3.41)	-3.56(-3.93, -3.20)	-4.64(-5.06, -4.23)	-2.90(-3.18, -2.62)
Other malignant neoplasms	364.38(246.18-453.15)	232.56(179.78-294.56)	-1.49(-1.59, -1.38)	-2.35(-2.49, -2.21)	-1.27(-1.54, -1.01)	-0.62(-0.75, -0.48)
Other mental disorders	193.20(122.67-291.84)	194.05(125.23-290.08)	0.02(0.01, 0.03)	0.08(0.05, 0.10)	0.03(0.03, 0.04)	-0.08(-0.10, -0.06)
Other musculoskeletal disorders	767.68(449.71-1, 254.61)	886.88(526.75-1, 435.04)	0.44(0.35, 0.52)	0.56(0.35, 0.77)	0.70(0.57, 0.82)	-0.05(-0.10, 0.00)
Other neglected tropical diseases	18.62(13.70-24.91)	8.44(6.35-11.33)	-2.63(-2.82, -2.43)	-2.40(-2.66, -2.13)	-2.95(-3.27, -2.63)	-2.65(-2.92, -2.38)
Other neoplasms	28.28(18.06-46.23)	42.93(29.34-68.45)	1.36(1.26, 1.47)	1.29(1.17, 1.41)	2.61(2.34, 2.89)	0.15(0.07, 0.22)

Other neurological disorders	22.74(16.34-31.00)	73.55(57.38-91.98)	3.96(3.70, 4.22)	5.45(4.86, 6.05)	2.53(2.21, 2.84)	4.14(3.80, 4.49)
Other nutritional deficiencies	12.32(9.97-15.10)	7.75(5.84-9.88)	-1.27(-1.69, -0.86)	-3.66(-4.29, -3.03)	1.35(0.68, 2.03)	-2.62(-2.88, -2.36)
Other pharynx cancer	59.04(48.88-71.04)	34.14(26.64-42.93)	-1.83(-2.02, -1.64)	-2.02(-2.51, -1.52)	-2.81(-3.01, -2.61)	-0.14(-0.25, -0.03)
Other sense organ diseases	196.28(94.25-349.63)	202.84(98.92-358.82)	0.12(0.08, 0.15)	0.09(0.01, 0.17)	0.20(0.15, 0.26)	0.07(0.04, 0.10)
Other skin and subcutaneous diseases	88.17(45.78-160.98)	113.68(57.82-209.59)	0.83(0.81, 0.85)	0.64(0.59, 0.69)	1.01(0.99, 1.02)	0.79(0.77, 0.82)
Other transport injuries	66.29(55.03-78.22)	33.64(27.56-40.97)	-2.16(-2.49, -1.84)	-0.68(-1.31, -0.03)	-3.40(-3.63, -3.17)	-2.47(-3.17, -1.77)
Other unintentional injuries	124.65(96.26-160.59)	65.99(47.85-90.15)	-1.99(-2.22, -1.75)	-1.84(-2.12, -1.56)	-2.18(-2.40, -1.95)	-2.08(-2.66, -1.49)
Other unspecified infectious diseases	39.44(22.43-52.40)	14.60(9.73-20.12)	-3.15(-3.41, -2.88)	-2.99(-3.64, -2.33)	-5.00(-5.39, -4.61)	-1.35(-1.57, -1.14)
Otitis media	20.64(9.94-37.08)	13.65(6.33-24.54)	-1.33(-1.37, -1.28)	-1.29(-1.39, -1.19)	-1.37(-1.39, -1.36)	-1.29(-1.39, -1.20)
Ovarian cancer	127.05(100.29-163.24)	122.19(90.96-156.75)	-0.11(-0.24, 0.03)	0.31(0.12, 0.50)	-1.36(-1.51, -1.21)	1.21(0.93, 1.48)
Pancreatic cancer	591.11(506.45-693.97)	774.46(610.85-947.60)	0.91(0.77, 1.06)	1.27(0.99, 1.55)	0.43(0.32, 0.54)	1.07(0.73, 1.42)
Pancreatitis	100.87(76.62-131.60)	65.48(49.56-92.21)	-1.42(-1.54, -1.30)	-1.46(-1.63, -1.28)	-1.17(-1.36, -0.98)	-1.81(-1.88, -1.74)
Paralytic ileus and intestinal obstruction	186.05(148.95-236.23)	72.30(55.40-101.21)	-3.03(-3.19, -2.87)	-2.56(-2.75, -2.37)	-3.41(-3.63, -3.19)	-3.26(-3.52, -3.01)
Parkinson's disease	739.52(641.44-855.40)	732.98(609.23-871.33)	-0.01(-0.16, 0.14)	0.55(0.27, 0.84)	-0.59(-0.74, -0.45)	0.02(-0.28, 0.32)
Pneumoconiosis	131.32(105.39-160.89)	54.50(41.13-69.81)	-2.94(-3.17, -2.72)	-2.37(-2.68, -2.07)	-3.62(-4.14, -3.10)	-3.04(-3.22, -2.86)
Poisonings	89.28(66.70-157.40)	80.75(41.32-105.89)	-0.16(-0.46, 0.15)	-0.65(-1.17, -0.13)	2.67(2.22, 3.12)	-3.60(-4.07, -3.12)
Police conflict and executions	1.19(0.25-2.05)	4.05(1.23-7.03)	4.07(3.45, 4.69)	2.67(0.88, 4.48)	4.39(4.11, 4.66)	5.17(4.86, 5.48)
Prostate cancer	221.57(166.15-295.29)	246.62(178.75-347.12)	0.41(0.23, 0.58)	1.81(1.42, 2.20)	-0.93(-1.06, -0.79)	0.26(-0.07, 0.58)
Protein-energy malnutrition	120.80(104.72-137.59)	22.42(18.04-27.48)	-5.41(-5.90, -4.92)	-7.96(-8.70, -7.23)	-5.84(-6.12, -5.57)	-2.12(-3.48, -0.74)
Pruritus	23.76(10.41-46.52)	27.64(11.99-53.55)	0.49(0.47, 0.50)	0.34(0.30, 0.38)	0.58(0.57, 0.59)	0.52(0.49, 0.55)
Psoriasis	64.20(47.03-84.12)	85.13(62.90-111.86)	0.92(0.89, 0.95)	1.36(1.29, 1.43)	0.75(0.73, 0.77)	0.66(0.61, 0.70)
Pulmonary Arterial Hypertension	51.33(37.52-70.57)	36.09(23.00-45.40)	-1.27(-1.57, -0.98)	-2.62(-2.97, -2.27)	2.87(2.15, 3.59)	-4.00(-4.36, -3.63)

Rabies	2.84(1.26-4.55)	1.47(0.63-2.54)	-2.14(-3.44, -0.81)	-4.61(-6.29, -2.89)	6.23(4.98, 7.50)	-9.75(-12.92, -6.47)
Rheumatic heart disease	1, 918.44(1, 581.46-2, 270.67)	430.90(338.44-555.34)	-4.93(-5.28, -4.59)	-4.97(-5.31, -4.63)	-5.22(-6.12, -4.31)	-4.85(-5.05, -4.65)
Rheumatoid arthritis	163.74(130.83-205.84)	156.27(121.46-198.24)	-0.13(-0.24, -0.01)	-0.26(-0.45, -0.06)	0.08(-0.09, 0.24)	-0.40(-0.58, -0.21)
Road injuries	982.81(851.64-1, 132.39)	898.22(765.26-1, 051.00)	-0.20(-0.34, -0.05)	1.15(0.94, 1.36)	0.16(-0.12, 0.45)	-2.29(-2.46, -2.13)
Scabies	89.95(45.37-165.12)	90.03(45.59-164.02)	0.00(-0.01, 0.00)	0.00(-0.01, 0.00)	0.00(-0.01, 0.00)	0.00(-0.01, 0.00)
Schistosomiasis	43.75(32.14-58.15)	6.82(4.47-11.05)	-5.83(-6.16, -5.49)	-6.85(-6.99, -6.72)	-7.15(-8.01, -6.28)	-3.15(-3.63, -2.68)
Schizophrenia	156.78(114.51-198.38)	163.18(120.67-206.03)	0.13(0.11, 0.15)	0.01(0.00, 0.03)	-0.01(-0.03, 0.01)	0.44(0.39, 0.49)
Self-harm	1, 430.54(941.22-1, 636.43)	542.12(437.08-669.75)	-3.15(-3.29, -3.01)	-1.47(-1.59, -1.35)	-5.51(-5.72, -5.29)	-2.37(-2.68, -2.06)
Sexually transmitted infections excluding HIV	13.79(8.89-21.16)	8.17(4.46-14.32)	-1.68(-2.17, -1.19)	-2.84(-4.24, -1.42)	-1.60(-1.87, -1.34)	-0.61(-1.06, -0.15)
Soft tissue and other extraosseous sarcomas	42.67(29.56-57.30)	25.21(17.42-34.90)	-1.65(-1.98, -1.31)	-0.83(-0.97, -0.69)	-2.76(-3.65, -1.87)	-1.28(-1.66, -0.90)
Stomach cancer	5, 978.83(5, 078.85-7, 239.43)	2, 921.50(2, 239.97-3, 693.85)	-2.28(-2.48, -2.08)	-1.32(-1.61, -1.04)	-3.37(-3.70, -3.04)	-2.12(-2.49, -1.75)
Stroke	29, 173.41(25, 669.43-32, 930.94)	16, 006.92(13, 528.17-18, 658.97)	-2.06(-2.38, -1.75)	-1.06(-1.42, -0.71)	-2.72(-3.53, -1.91)	-2.63(-2.83, -2.42)
Testicular cancer	5.91(4.69-7.34)	5.03(3.67-6.75)	-0.42(-0.58, -0.27)	0.56(0.27, 0.85)	-2.46(-2.67, -2.25)	1.02(0.81, 1.24)
Tetanus	29.10(11.24-43.37)	1.02(0.25-2.63)	-10.44(-11.22, -9.65)	-10.71(-12.51, -8.87)	-10.00(-10.82, -9.18)	-11.09(-11.45, -10.73)
Thyroid cancer	50.06(40.43-60.51)	37.67(30.70-47.07)	-0.91(-1.00, -0.82)	-0.87(-1.03, -0.71)	-1.39(-1.45, -1.33)	-0.31(-0.54, -0.08)
Tracheal, bronchus, and lung cancer	4, 642.82(3, 965.98-5, 291.38)	5, 628.22(4, 476.87-6, 875.06)	0.58(0.35, 0.81)	1.76(1.45, 2.07)	0.17(-0.42, 0.76)	-0.43(-0.58, -0.28)
Trachoma	5.71(2.76-9.70)	0.60(0.26-1.13)	-6.95(-8.07, -5.82)	-3.75(-5.78, -1.67)	-9.92(-12.46, -7.31)	-7.89(-8.45, -7.32)
Tuberculosis	2, 228.85(1, 855.70-2, 641.11)	320.39(248.67-398.83)	-6.17(-6.37, -5.98)	-5.55(-5.83, -5.26)	-7.16(-7.60, -6.71)	-6.09(-6.27, -5.90)
Typhoid and paratyphoid	1.01(0.33-2.15)	0.34(0.14-0.63)	-3.46(-3.65, -3.27)	-4.33(-4.56, -4.10)	-2.59(-3.11, -2.08)	-3.49(-3.60, -3.37)
Upper digestive system diseases	1, 319.14(1, 131.03-1, 529.04)	453.84(366.56-574.75)	-3.41(-3.53, -3.28)	-3.73(-3.85, -3.61)	-3.51(-3.81, -3.21)	-3.43(-3.53, -3.33)
Upper respiratory infections	154.99(46.54-223.24)	32.74(19.80-51.80)	-4.99(-5.16, -4.81)	-4.76(-4.98, -4.53)	-7.71(-7.98, -7.43)	-1.73(-2.00, -1.45)

Urinary diseases and male infertility	403.03(277.66-575.33)	282.43(184.15-415.80)	-1.12(-1.25, -0.99)	-1.27(-1.52, -1.03)	-1.33(-1.52, -1.14)	-0.84(-1.07, -0.61)
Urticaria	36.49(22.05-55.75)	36.02(21.79-56.01)	-0.04(-0.06, -0.02)	-0.11(-0.17, -0.05)	-0.04(-0.06, -0.02)	0.04(0.02, 0.05)
Uterine cancer	130.12(97.36-164.03)	72.78(53.12-102.54)	-1.85(-2.03, -1.67)	-0.82(-0.92, -0.71)	-2.58(-2.80, -2.36)	-1.78(-2.23, -1.33)
Varicella and herpes zoster	21.95(16.65-28.32)	10.35(6.15-16.14)	-2.43(-2.59, -2.26)	-2.96(-3.13, -2.79)	-3.77(-4.16, -3.38)	-0.60(-0.80, -0.40)
Vascular intestinal disorders	10.81(8.44-12.83)	4.61(3.75-5.74)	-2.81(-3.02, -2.59)	-0.65(-1.01, -0.29)	-5.56(-5.84, -5.27)	-1.87(-2.23, -1.52)
Viral skin diseases	44.48(28.17-67.70)	45.12(28.33-69.15)	0.05(0.04, 0.06)	0.14(0.13, 0.15)	0.08(0.06, 0.09)	-0.10(-0.12, -0.08)
Vitamin A deficiency	1.72(0.94-2.76)	1.71(0.88-2.82)	0.04(-0.16, 0.25)	0.58(0.19, 0.98)	0.28(-0.06, 0.61)	-0.86(-1.12, -0.60)
**75-79 years**						
Acne vulgaris	4.29(2.73-6.57)	5.07(3.21-7.86)	0.54(0.53, 0.55)	0.59(0.58, 0.60)	0.59(0.58, 0.60)	0.44(0.41, 0.47)
Acute glomerulonephritis	101.80(73.89-140.41)	34.80(22.38-48.78)	-3.40(-3.71, -3.10)	-4.28(-4.76, -3.79)	-2.07(-2.36, -1.78)	-4.25(-4.92, -3.57)
Acute hepatitis	201.60(164.71-248.55)	15.72(12.44-20.02)	-8.01(-8.50, -7.52)	-8.15(-8.72, -7.57)	-10.62(-11.24, -9.99)	-4.56(-5.86, -3.23)
Adverse effects of medical treatment	66.10(43.04-83.96)	17.88(13.97-24.34)	-4.21(-4.53, -3.90)	-4.26(-4.52, -4.00)	-5.84(-6.72, -4.96)	-2.50(-2.72, -2.28)
Age-related and other hearing loss	3, 224.43(2, 236.09-4, 492.61)	3, 525.30(2, 451.22-4, 822.00)	0.33(0.25, 0.41)	-0.39(-0.48, -0.30)	0.73(0.57, 0.88)	0.60(0.48, 0.71)
Alcohol use disorders	97.46(74.17-128.76)	112.53(81.55-151.15)	0.47(0.21, 0.72)	0.37(-0.28, 1.03)	2.34(2.13, 2.54)	-1.87(-2.27, -1.48)
Alopecia areata	5.56(3.56-8.07)	5.43(3.43-7.95)	-0.07(-0.11, -0.04)	-0.08(-0.16, -0.01)	-0.12(-0.13, -0.10)	0.00(-0.07, 0.06)
Alzheimer's disease and other dementias	4, 279.13(1, 982.02-9, 704.12)	4, 621.82(2, 286.20-10, 064.76)	0.14(0.00, 0.28)	-0.04(-0.08, -0.01)	-0.04(-0.08, -0.01)	0.58(0.12, 1.04)
Animal contact	43.20(32.91-50.26)	11.85(9.72-14.81)	-4.13(-4.33, -3.92)	-3.41(-3.85, -2.97)	-6.18(-6.51, -5.84)	-2.68(-2.91, -2.44)
Anxiety disorders	518.85(361.71-726.73)	520.51(353.02-725.59)	-0.08(-0.38, 0.23)	0.62(0.31, 0.93)	-0.99(-1.29, -0.68)	0.62(-0.13, 1.38)
Aortic aneurysm	39.56(32.22-49.13)	54.06(41.87-68.84)	0.92(0.83, 1.01)	0.69(0.61, 0.78)	2.42(2.20, 2.63)	-0.31(-0.45, -0.16)
Appendicitis	59.17(41.42-74.91)	11.90(8.78-15.83)	-5.09(-5.31, -4.87)	-4.06(-4.46, -3.67)	-7.31(-7.68, -6.93)	-4.10(-4.38, -3.81)
Asthma	1, 124.39(878.96-1, 543.67)	306.21(245.29-374.99)	-4.15(-4.30, -4.01)	-3.15(-3.25, -3.05)	-5.69(-6.06, -5.32)	-3.58(-3.78, -3.38)
Atrial fibrillation and flutter	687.00(519.63-888.38)	657.27(505.36-851.55)	-0.16(-0.30, -0.02)	-0.47(-0.67, -0.26)	-0.35(-0.53, -0.17)	0.39(0.13, 0.65)

Attention-deficit/hyperactivity disorder	0.25(0.05-0.62)	0.22(0.04-0.55)	-0.35(-0.46, -0.25)	-1.16(-1.28, -1.05)	-0.16(-0.45, 0.14)	0.23(0.17, 0.29)
Autism spectrum disorders	56.68(38.50-80.13)	75.40(50.96-106.13)	0.93(0.91, 0.96)	1.01(0.97, 1.05)	1.22(1.20, 1.24)	0.52(0.48, 0.56)
Bacterial skin diseases	88.11(68.09-105.93)	15.23(10.86-23.52)	-5.56(-5.94, -5.19)	-3.22(-3.83, -2.60)	-12.38(-12.90, -11.85)	-1.06(-1.73, -0.39)
Bipolar disorder	39.78(25.05-57.88)	39.36(24.93-58.31)	-0.04(-0.08, 0.01)	-0.10(-0.16, -0.04)	-0.06(-0.12, 0.01)	0.03(0.00, 0.06)
Bladder cancer	543.07(416.53-627.13)	387.92(307.03-487.42)	-1.15(-1.37, -0.94)	-0.57(-0.76, -0.38)	-2.01(-2.59, -1.44)	-0.80(-0.91, -0.68)
Blindness and vision loss	1, 951.98(1, 392.41-2, 706.30)	1, 626.86(1, 133.12-2, 364.66)	-0.53(-0.71, -0.36)	1.00(0.46, 1.53)	-1.25(-1.33, -1.18)	-1.25(-1.33, -1.18)
Brain and central nervous system cancer	263.03(204.94-341.55)	318.33(231.56-398.25)	0.63(0.54, 0.72)	1.31(1.07, 1.55)	0.53(0.47, 0.59)	-0.08(-0.14, -0.01)
Breast cancer	356.10(293.22-426.43)	338.71(269.09-415.03)	-0.14(-0.56, 0.29)	0.52(0.04, 0.99)	-1.04(-1.53, -0.55)	0.39(-0.63, 1.43)
Cardiomyopathy and myocarditis	128.16(103.66-211.33)	156.60(119.59-197.16)	0.63(0.00, 1.26)	3.69(1.76, 5.65)	-0.04(-0.31, 0.24)	-1.90(-2.07, -1.74)
Cervical cancer	296.96(242.14-359.30)	195.22(144.01-247.56)	-1.32(-1.59, -1.04)	-2.77(-3.00, -2.54)	-1.03(-1.76, -0.29)	-0.53(-0.69, -0.37)
Chronic kidney disease	2, 251.68(1, 941.46-2, 646.01)	1, 923.16(1, 582.00-2, 261.38)	-0.48(-0.67, -0.28)	-0.79(-1.20, -0.38)	-0.07(-0.27, 0.13)	-0.77(-0.92, -0.61)
Chronic obstructive pulmonary disease	37, 249.46(32, 243.00-41, 287.97)	12, 169.13(10, 238.53-14, 476.37)	-3.71(-4.14, -3.28)	-3.13(-3.34, -2.92)	-5.13(-6.11, -4.13)	-2.78(-3.63, -1.92)
Cirrhosis and other chronic liver diseases	1, 845.49(1, 573.41-2, 152.43)	778.11(626.91-947.63)	-2.92(-3.23, -2.61)	-2.58(-2.89, -2.26)	-3.32(-4.06, -2.56)	-3.10(-3.32, -2.88)
Colon and rectum cancer	1, 940.40(1, 668.34-2, 216.29)	1, 971.34(1, 605.21-2, 356.98)	0.05(-0.17, 0.26)	0.12(-0.29, 0.53)	0.17(-0.10, 0.43)	-0.25(-0.42, -0.08)
Conflict and terrorism	0.10(0.06-0.14)	0.11(0.08-0.16)	1.66(-1.60, 5.02)	11.43(3.09, 20.45)	-3.35(-6.29, -0.32)	-3.35(-6.29, -0.32)
Congenital birth defects	25.16(16.38-36.54)	25.17(17.17-36.54)	-0.01(-0.27, 0.26)	1.65(1.17, 2.14)	-0.53(-0.66, -0.41)	-1.18(-1.85, -0.51)
Cystic echinococcosis	2.76(1.88-3.77)	0.93(0.59-1.47)	-3.54(-3.83, -3.25)	-4.78(-5.02, -4.53)	-3.11(-3.85, -2.36)	-3.17(-3.36, -2.97)
Cysticercosis	77.59(34.13-139.13)	38.47(15.39-73.06)	-2.15(-2.59, -1.70)	-1.89(-2.28, -1.50)	-3.82(-4.04, -3.60)	-0.38(-1.79, 1.05)
Decubitus ulcer	8.19(4.63-18.90)	20.34(11.36-26.06)	4.00(2.89, 5.13)	11.76(10.36, 13.18)	2.51(0.53, 4.52)	-2.10(-4.20, 0.04)
Dengue	0.13(0.06-0.20)	0.05(0.02-0.09)	-2.89(-4.01, -1.76)	-9.05(-11.96, -6.05)	1.26(0.48, 2.05)	-1.64(-2.05, -1.23)
Depressive disorders	819.80(560.33-1, 123.32)	969.09(660.73-1, 353.99)	0.54(0.45, 0.63)	1.45(1.29, 1.60)	0.44(0.42, 0.46)	-0.33(-0.57, -0.10)
Dermatitis	104.47(57.07-177.51)	103.84(57.54-176.44)	-0.02(-0.03, -0.01)	0.00(-0.02, 0.02)	-0.03(-0.04, -0.03)	-0.02(-0.04, 0.00)

Diabetes mellitus	2, 440.40(2, 073.39-2, 867.26)	2, 804.37(2, 314.96-3, 374.24)	0.45(0.26, 0.64)	1.78(1.68, 1.87)	-0.17(-0.73, 0.38)	-0.38(-0.49, -0.28)
Diarrheal diseases	325.59(154.83-485.42)	28.56(18.75-49.38)	-7.60(-7.84, -7.36)	-8.83(-9.29, -8.36)	-8.81(-9.02, -8.60)	-4.76(-5.17, -4.35)
Dietary iron deficiency	462.29(315.27-640.80)	197.34(130.23-281.64)	-2.76(-2.88, -2.64)	-1.71(-2.04, -1.39)	-4.10(-4.24, -3.96)	-2.30(-2.42, -2.17)
Drowning	271.18(236.96-308.10)	177.52(147.91-208.56)	-1.33(-1.53, -1.14)	-1.04(-1.23, -0.85)	-1.39(-1.91, -0.88)	-2.02(-2.13, -1.91)
Drug use disorders	155.32(126.19-187.39)	47.72(36.63-60.00)	-3.91(-4.25, -3.57)	-2.90(-3.32, -2.49)	-6.84(-7.34, -6.34)	-1.02(-1.64, -0.39)
Encephalitis	16.56(13.58-19.86)	11.45(9.41-14.29)	-1.21(-1.50, -0.92)	-0.76(-1.18, -0.33)	-1.08(-1.34, -0.82)	-1.88(-2.58, -1.17)
Endocarditis	30.91(16.10-40.72)	10.32(7.88-15.13)	-3.47(-3.69, -3.25)	-1.48(-1.78, -1.18)	-6.94(-7.43, -6.45)	-1.26(-1.51, -1.01)
Endocrine, metabolic, blood, and immune disorders	339.47(233.23-490.01)	316.09(214.86-464.25)	-0.15(-0.84, 0.53)	0.93(0.51, 1.36)	-1.08(-2.87, 0.76)	-0.58(-1.59, 0.44)
Environmental heat and cold exposure	74.91(33.29-97.10)	28.10(13.46-40.24)	-3.12(-3.32, -2.91)	-3.11(-3.57, -2.66)	-4.49(-4.85, -4.14)	-1.63(-1.78, -1.48)
Esophageal cancer	3, 160.24(2, 559.24-3, 629.64)	2, 091.81(1, 664.98-2, 545.53)	-1.25(-1.71, -0.80)	0.20(-0.01, 0.42)	-2.65(-3.82, -1.45)	-1.29(-1.96, -0.62)
Exposure to forces of nature	12.07(10.90-13.39)	5.03(4.05-6.22)	0.28(-4.40, 5.19)	0.28(-4.40, 5.19)	0.28(-4.40, 5.19)	0.28(-4.40, 5.19)
Exposure to mechanical forces	92.69(72.66-125.41)	102.04(74.11-134.91)	0.38(-0.02, 0.78)	0.22(-0.19, 0.62)	2.56(2.29, 2.83)	-1.98(-3.14, -0.81)
Eye cancer	5.76(3.48-7.63)	4.56(2.30-6.32)	-0.77(-1.03, -0.51)	-0.84(-0.93, -0.75)	-0.49(-1.26, 0.29)	-0.97(-1.17, -0.77)
Falls	1, 513.78(1, 224.71-1, 864.01)	1, 620.79(1, 197.60-2, 041.17)	0.31(0.17, 0.45)	0.24(-0.07, 0.55)	-1.23(-1.42, -1.04)	1.80(1.64, 1.97)
Fire, heat, and hot substances	168.94(129.85-198.09)	78.08(61.29-96.07)	-2.52(-2.90, -2.13)	-3.43(-3.99, -2.87)	-1.26(-2.28, -0.23)	-2.67(-2.81, -2.52)
Food-borne trematodiases	138.96(58.71-276.74)	70.14(36.03-123.58)	-2.27(-3.14, -1.40)	-3.63(-4.76, -2.49)	-1.92(-2.24, -1.61)	-2.11(-3.96, -0.22)
Foreign body	66.70(50.41-79.57)	63.15(41.09-77.78)	-0.21(-0.62, 0.20)	-0.28(-0.48, -0.09)	-0.55(-1.75, 0.67)	0.21(-0.09, 0.50)
Fungal skin diseases	52.14(20.83-108.24)	51.49(20.66-106.55)	-0.04(-0.05, -0.04)	-0.02(-0.03, -0.01)	-0.06(-0.06, -0.06)	-0.05(-0.06, -0.04)
Gallbladder and biliary diseases	748.12(515.06-958.33)	489.43(342.62-697.87)	-1.40(-1.67, -1.13)	-1.61(-1.70, -1.53)	-1.32(-2.02, -0.62)	-1.11(-1.56, -0.66)
Gallbladder and biliary tract cancer	340.91(259.80-435.59)	286.91(204.60-379.55)	-0.66(-0.88, -0.44)	-0.36(-0.72, -0.01)	-0.43(-0.79, -0.07)	-1.34(-1.50, -1.17)
Gout	81.39(49.05-124.26)	111.50(65.90-173.55)	1.07(0.96, 1.18)	-0.71(-0.99, -0.44)	2.47(2.31, 2.63)	1.26(1.18, 1.34)

Gynecological diseases	153.93(94.50-233.16)	107.58(68.77-161.08)	-1.14(-1.28, -1.00)	-0.49(-0.54, -0.45)	-2.87(-3.23, -2.52)	0.00(-0.10, 0.10)
Headache disorders	327.85(89.29-696.01)	342.00(90.02-752.43)	0.13(0.12, 0.14)	-0.10(-0.12, -0.08)	0.40(0.37, 0.43)	-0.01(-0.02, 0.00)
Hemoglobinopathies and hemolytic anemias	258.10(196.57-333.71)	130.16(100.88-164.59)	-2.19(-2.25, -2.14)	-2.01(-2.07, -1.96)	-2.50(-2.52, -2.47)	-2.04(-2.22, -1.87)
HIV/AIDS	12.65(8.60-16.55)	69.82(48.39-97.80)	6.52(4.39, 8.69)	10.30(9.60, 11.01)	5.90(3.86, 7.97)	3.89(-2.08, 10.22)
Hodgkin lymphoma	41.79(17.74-57.43)	13.72(7.80-17.98)	-3.60(-3.76, -3.44)	-3.31(-3.49, -3.13)	-5.17(-5.45, -4.89)	-2.28(-2.67, -1.90)
Hypertensive heart disease	6, 329.30(4, 415.06-7, 417.78)	2, 553.23(1, 855.17-3, 285.45)	-2.96(-3.15, -2.77)	-4.28(-4.50, -4.06)	-3.28(-3.59, -2.95)	-1.04(-1.46, -0.61)
Idiopathic developmental intellectual disability	6.76(2.34-13.49)	4.34(1.20-9.18)	-1.44(-1.54, -1.35)	0.67(0.42, 0.92)	-2.94(-3.05, -2.83)	-1.87(-1.99, -1.75)
Idiopathic epilepsy	134.76(89.59-192.88)	106.00(63.57-161.80)	-0.66(-0.88, -0.43)	-0.10(-0.26, 0.06)	-2.12(-2.22, -2.03)	0.49(-0.24, 1.23)
Inflammatory bowel disease	79.76(45.46-104.23)	37.89(28.92-52.30)	-2.41(-2.65, -2.16)	-1.54(-1.99, -1.08)	-3.60(-3.91, -3.29)	-1.96(-2.29, -1.63)
Inguinal, femoral, and abdominal hernia	31.04(23.78-41.30)	17.82(13.17-24.18)	-1.81(-2.07, -1.54)	-1.49(-1.77, -1.22)	-2.03(-2.23, -1.83)	-1.96(-2.71, -1.21)
Interpersonal violence	161.54(134.33-195.62)	81.33(65.90-101.31)	-2.19(-2.29, -2.09)	-1.67(-1.79, -1.54)	-2.78(-2.87, -2.68)	-2.03(-2.27, -1.78)
Interstitial lung disease and pulmonary sarcoidosis	79.63(61.29-127.25)	85.41(57.96-115.41)	0.23(0.04, 0.41)	-0.45(-0.66, -0.23)	2.58(2.09, 3.06)	-1.51(-1.65, -1.36)
Intestinal nematode infections	62.46(32.72-99.73)	1.34(0.72-2.20)	-11.79(-12.13, -11.44)	-12.42(-13.02, -11.82)	-15.73(-16.33, -15.13)	-5.95(-6.42, -5.48)
Invasive Non-typhoidal Salmonella (iNTS)	2.63(0.70-6.15)	1.56(0.42-3.87)	-1.66(-1.76, -1.55)	-1.27(-1.47, -1.08)	-2.40(-2.57, -2.24)	-1.20(-1.37, -1.02)
Iodine deficiency	16.05(8.65-27.43)	16.67(7.88-31.61)	0.12(-0.03, 0.28)	-0.75(-0.87, -0.62)	1.59(1.17, 2.01)	-1.09(-1.20, -0.98)
Ischemic heart disease	11, 664.44(10, 267.41-13, 223.95)	13, 434.69(11, 314.93-15, 770.79)	0.47(0.08, 0.85)	0.79(0.21, 1.38)	1.68(1.18, 2.18)	-1.66(-2.40, -0.91)
Kidney cancer	122.34(105.77-136.97)	160.12(130.33-191.45)	0.80(0.51, 1.10)	0.88(0.40, 1.36)	1.97(1.59, 2.35)	-0.90(-1.30, -0.50)
Larynx cancer	197.58(162.71-226.68)	131.15(105.13-162.17)	-1.30(-1.51, -1.09)	-1.73(-1.86, -1.61)	-0.83(-1.13, -0.53)	-1.49(-1.98, -0.99)
Leishmaniasis	0.69(0.06-2.00)	0.24(0.05-0.59)	-3.45(-3.81, -3.10)	-5.78(-6.21, -5.34)	-1.05(-1.41, -0.69)	-3.61(-4.49, -2.73)
Leprosy	0.26(0.15-0.39)	0.07(0.04-0.12)	-3.97(-4.07, -3.87)	-3.81(-3.89, -3.72)	-5.29(-5.41, -5.17)	-2.76(-3.07, -2.46)

Leukemia	324.59(263.78-393.16)	252.41(184.87-324.11)	-0.81(-1.02, -0.61)	-0.49(-0.98, 0.00)	-1.05(-1.14, -0.96)	-1.05(-1.14, -0.96)
Lip and oral cavity cancer	140.90(121.88-160.89)	147.92(118.53-181.09)	0.09(-0.10, 0.28)	-0.56(-0.69, -0.43)	1.12(0.87, 1.38)	-0.56(-1.04, -0.09)
Liver cancer	875.87(736.55-1, 015.67)	768.27(630.58-936.37)	-0.36(-0.64, -0.07)	1.36(0.66, 2.06)	-1.71(-2.16, -1.25)	-0.98(-1.16, -0.81)
Low back pain	2, 278.54(1, 407.37-3, 362.80)	1, 734.97(1, 078.04-2, 528.84)	-0.86(-0.89, -0.83)	-1.80(-1.89, -1.71)	-0.41(-0.43, -0.40)	-0.41(-0.43, -0.40)
Lower extremity peripheral arterial disease	97.19(50.61-173.13)	90.18(48.29-155.93)	-0.25(-0.35, -0.15)	0.17(-0.05, 0.38)	-0.54(-0.57, -0.50)	-0.32(-0.57, -0.08)
Lower respiratory infections	3, 339.24(2, 708.87-3, 827.39)	1, 171.21(946.69-1, 457.01)	-3.42(-3.74, -3.10)	-2.97(-3.42, -2.52)	-4.24(-5.06, -3.40)	-3.04(-3.22, -2.86)
Malignant neoplasm of bone and articular cartilage	61.83(39.02-107.79)	110.12(67.71-146.95)	1.97(1.72, 2.22)	3.55(2.82, 4.28)	3.03(2.85, 3.20)	-1.37(-1.47, -1.27)
Malignant skin melanoma	30.19(21.04-39.76)	29.55(15.32-39.14)	-0.06(-0.23, 0.12)	0.08(-0.20, 0.36)	0.60(0.22, 0.98)	-0.75(-0.98, -0.52)
Meningitis	91.74(79.47-105.93)	23.41(19.66-28.09)	-4.26(-4.87, -3.65)	-6.18(-7.30, -5.05)	-3.86(-4.60, -3.12)	-2.63(-3.78, -1.46)
Mesothelioma	15.55(12.54-17.90)	16.69(13.30-20.05)	0.14(-0.04, 0.32)	-0.29(-0.56, -0.01)	2.65(2.31, 2.98)	-2.02(-2.28, -1.76)
Motor neuron disease	6.99(4.31-9.11)	11.24(7.06-14.78)	1.36(1.05, 1.68)	3.76(3.28, 4.23)	-1.12(-1.76, -0.48)	2.28(2.06, 2.50)
Multiple myeloma	24.73(16.38-49.92)	83.58(56.07-109.89)	4.44(3.72, 5.17)	11.47(10.40, 12.55)	1.29(-0.51, 3.13)	1.37(0.91, 1.84)
Multiple sclerosis	0.78(0.51-1.10)	1.21(0.89-1.59)	1.42(1.25, 1.58)	2.29(2.23, 2.35)	0.98(0.90, 1.06)	1.23(0.71, 1.76)
Nasopharynx cancer	289.79(247.99-329.53)	118.54(96.75-143.56)	-2.83(-3.08, -2.57)	-2.63(-3.28, -1.98)	-4.04(-4.37, -3.71)	-1.41(-1.58, -1.24)
Neck pain	554.75(291.33-933.07)	574.89(302.16-955.83)	0.11(-0.05, 0.28)	-0.66(-0.97, -0.34)	1.17(0.85, 1.49)	-0.04(-0.20, 0.12)
Neonatal disorders	15.16(10.40-22.02)	24.33(17.68-31.47)	1.55(1.47, 1.62)	-0.05(-0.15, 0.05)	2.43(2.29, 2.57)	2.16(2.03, 2.29)
Neuroblastoma and other peripheral nervous cell tumors	1.08(0.70-1.61)	4.10(2.84-5.14)	4.45(4.30, 4.61)	2.92(2.78, 3.06)	7.38(6.98, 7.78)	2.51(2.31, 2.71)
Non-Hodgkin lymphoma	241.56(206.60-299.23)	237.00(188.79-285.10)	-0.10(-0.28, 0.08)	0.07(-0.12, 0.26)	0.45(-0.01, 0.90)	-0.65(-0.86, -0.43)
Non-melanoma skin cancer	88.92(71.76-111.27)	107.80(85.48-131.11)	0.54(0.22, 0.87)	0.55(0.03, 1.06)	1.93(1.46, 2.39)	-1.38(-1.84, -0.91)
Non-rheumatic valvular heart disease	56.43(41.73-78.64)	46.12(32.66-66.42)	-0.60(-0.91, -0.29)	-0.06(-0.17, 0.05)	-0.75(-1.68, 0.20)	-1.04(-1.21, -0.87)

Oral disorders	1, 128.46(708.96-1, 661.13)	1, 071.55(677.40-1, 547.83)	-0.02(-0.36, 0.32)	-1.70(-2.11, -1.28)	0.28(-0.17, 0.72)	1.74(1.06, 2.43)
Osteoarthritis	1, 063.59(505.29-2, 169.11)	1, 239.88(590.61-2, 531.01)	0.56(0.42, 0.71)	-0.06(-0.24, 0.12)	0.26(-0.02, 0.55)	1.59(1.39, 1.80)
Other cardiovascular and circulatory diseases	214.92(155.55-298.38)	176.70(121.75-265.30)	-0.61(-0.74, -0.49)	-0.35(-0.47, -0.24)	-0.63(-0.97, -0.28)	-0.88(-1.03, -0.73)
Other chronic respiratory diseases	21.04(15.91-36.32)	28.00(20.70-35.53)	1.12(0.82, 1.41)	2.32(1.70, 2.95)	4.12(3.70, 4.55)	-2.97(-3.29, -2.65)
Other digestive diseases	306.32(209.78-380.96)	92.15(75.99-112.75)	-3.86(-4.02, -3.71)	-4.93(-5.25, -4.62)	-4.98(-5.08, -4.88)	-1.25(-1.57, -0.93)
Other intestinal infectious diseases	0.63(0.02-1.84)	0.21(0.01-0.61)	-3.51(-3.84, -3.19)	-3.55(-3.86, -3.24)	-4.00(-4.83, -3.16)	-3.22(-3.37, -3.07)
Other malignant neoplasms	381.75(261.69-478.64)	259.34(200.64-323.88)	-1.28(-1.39, -1.16)	-2.20(-2.37, -2.04)	-0.66(-0.91, -0.42)	-1.06(-1.24, -0.87)
Other mental disorders	192.83(125.57-287.60)	194.54(127.18-287.97)	0.03(0.02, 0.04)	0.08(0.05, 0.11)	0.05(0.04, 0.05)	-0.05(-0.07, -0.02)
Other musculoskeletal disorders	663.52(399.22-1, 040.92)	719.41(428.19-1, 155.37)	0.24(0.14, 0.35)	0.42(0.16, 0.68)	0.69(0.54, 0.85)	-0.55(-0.61, -0.49)
Other neglected tropical diseases	20.30(14.67-27.24)	9.86(7.26-12.93)	-2.38(-2.56, -2.20)	-1.84(-2.09, -1.60)	-2.91(-3.21, -2.61)	-2.42(-2.66, -2.18)
Other neoplasms	33.08(21.72-58.41)	52.36(35.73-83.88)	1.52(1.45, 1.58)	1.31(1.21, 1.42)	3.15(3.01, 3.30)	0.05(-0.01, 0.11)
Other neurological disorders	28.00(20.35-37.03)	82.03(64.04-104.49)	3.58(3.30, 3.87)	4.64(4.39, 4.90)	2.51(2.30, 2.72)	3.63(2.74, 4.53)
Other nutritional deficiencies	21.61(17.34-26.51)	16.33(12.57-20.67)	-0.71(-1.57, 0.15)	-3.28(-5.49, -1.02)	2.14(1.14, 3.14)	-2.18(-2.45, -1.92)
Other pharynx cancer	51.48(43.23-60.24)	32.14(25.53-39.12)	-1.55(-1.82, -1.29)	-1.99(-2.39, -1.59)	-1.86(-2.41, -1.30)	-0.43(-0.81, -0.05)
Other sense organ diseases	233.80(113.63-429.24)	241.02(116.39-441.82)	0.09(0.04, 0.14)	0.08(0.07, 0.10)	0.15(0.03, 0.27)	0.04(-0.06, 0.13)
Other skin and subcutaneous diseases	98.05(51.04-170.28)	123.56(64.39-217.03)	0.75(0.73, 0.78)	0.56(0.49, 0.64)	0.96(0.94, 0.99)	0.68(0.65, 0.71)
Other transport injuries	63.56(52.48-74.70)	33.34(26.87-40.80)	-2.03(-2.43, -1.63)	-0.61(-1.33, 0.12)	-3.23(-3.48, -2.98)	-2.28(-3.22, -1.33)
Other unintentional injuries	115.86(87.90-152.42)	67.16(48.24-91.33)	-1.71(-1.90, -1.51)	-1.51(-1.76, -1.27)	-1.86(-2.05, -1.67)	-1.90(-2.39, -1.41)
Other unspecified infectious diseases	47.71(26.72-63.25)	19.32(12.43-27.07)	-2.81(-3.14, -2.49)	-2.74(-3.51, -1.96)	-4.59(-5.07, -4.10)	-1.01(-1.28, -0.74)
Otitis media	18.42(9.73-32.02)	12.94(6.43-22.66)	-1.14(-1.18, -1.10)	-1.05(-1.14, -0.96)	-1.25(-1.28, -1.22)	-1.10(-1.15, -1.05)
Ovarian cancer	114.96(91.48-144.03)	102.01(75.79-137.25)	-0.39(-0.68, -0.11)	0.67(0.30, 1.05)	-1.67(-1.96, -1.39)	0.21(-0.45, 0.87)
Pancreatic cancer	565.31(485.86-659.54)	761.37(608.90-924.27)	1.06(0.69, 1.43)	1.86(1.62, 2.10)	0.98(0.60, 1.37)	0.37(-0.67, 1.43)

Pancreatitis	118.09(90.39-159.43)	80.13(61.97-106.10)	-1.28(-1.45, -1.10)	-1.46(-1.66, -1.25)	-0.97(-1.33, -0.60)	-1.58(-1.67, -1.50)
Paralytic ileus and intestinal obstruction	285.16(228.66-355.20)	119.92(93.23-163.93)	-2.78(-2.95, -2.61)	-2.13(-2.41, -1.85)	-3.28(-3.46, -3.11)	-2.98(-3.29, -2.67)
Parkinson's disease	1, 279.47(1, 103.08-1, 458.90)	1, 255.05(1, 065.93-1, 474.89)	-0.05(-0.33, 0.23)	0.58(0.47, 0.69)	-0.41(-1.24, 0.44)	-0.38(-0.55, -0.20)
Pneumoconiosis	142.02(113.94-174.14)	70.82(55.61-89.86)	-2.26(-2.43, -2.08)	-1.92(-2.08, -1.76)	-2.47(-2.93, -2.01)	-2.59(-2.68, -2.51)
Poisonings	91.82(67.99-176.94)	88.58(48.37-114.26)	0.05(-0.32, 0.42)	-0.17(-0.86, 0.53)	2.85(2.32, 3.38)	-3.66(-4.18, -3.14)
Police conflict and executions	0.77(0.17-1.33)	2.80(0.80-5.02)	4.30(3.86, 4.74)	1.88(0.69, 3.08)	4.42(4.08, 4.77)	6.67(6.25, 7.09)
Prostate cancer	326.71(248.06-467.61)	389.03(289.17-536.49)	0.54(0.30, 0.78)	2.01(1.71, 2.31)	0.01(-0.44, 0.46)	-0.40(-0.74, -0.06)
Protein-energy malnutrition	224.01(196.72-256.72)	47.43(38.78-56.74)	-4.97(-5.32, -4.61)	-7.36(-7.95, -6.78)	-5.52(-5.73, -5.31)	-1.68(-2.63, -0.72)
Pruritus	25.03(10.64-51.17)	28.64(12.30-58.15)	0.43(0.42, 0.44)	0.34(0.33, 0.36)	0.52(0.51, 0.52)	0.43(0.40, 0.46)
Psoriasis	62.31(45.22-83.57)	82.02(60.15-110.60)	0.89(0.84, 0.95)	1.36(1.20, 1.51)	0.68(0.66, 0.69)	0.68(0.66, 0.69)
Pulmonary Arterial Hypertension	75.76(53.63-106.36)	54.26(32.69-69.29)	-1.19(-1.52, -0.86)	-2.45(-2.63, -2.26)	3.13(2.13, 4.14)	-3.99(-4.28, -3.70)
Rabies	1.92(0.97-2.90)	1.22(0.61-1.95)	-1.41(-2.51, -0.29)	-3.84(-5.27, -2.39)	6.32(5.23, 7.42)	-8.33(-10.99, -5.59)
Rheumatic heart disease	2, 444.19(2, 017.15-2, 956.92)	581.83(456.39-741.94)	-4.71(-5.04, -4.38)	-5.19(-5.53, -4.86)	-4.94(-5.79, -4.08)	-4.21(-4.40, -4.02)
Rheumatoid arthritis	185.43(150.90-225.44)	179.61(141.53-224.02)	-0.09(-0.23, 0.04)	-0.21(-0.43, 0.02)	0.07(-0.10, 0.24)	-0.33(-0.57, -0.09)
Road injuries	888.56(767.94-1, 024.38)	825.23(705.48-965.20)	-0.27(-0.50, -0.05)	1.61(1.19, 2.02)	0.05(-0.34, 0.43)	-2.82(-3.13, -2.51)
Scabies	98.30(51.17-164.89)	98.31(50.65-162.65)	-0.01(-0.02, 0.01)	0.02(0.00, 0.04)	0.05(0.00, 0.09)	-0.08(-0.09, -0.06)
Schistosomiasis	44.30(31.86-60.52)	7.05(4.76-11.29)	-5.78(-6.28, -5.28)	-6.57(-6.77, -6.36)	-7.45(-8.74, -6.14)	-2.96(-3.64, -2.27)
Schizophrenia	119.45(88.71-150.95)	124.25(91.23-155.96)	0.13(0.11, 0.16)	0.00(-0.03, 0.04)	0.09(0.07, 0.12)	0.35(0.29, 0.41)
Self-harm	1, 478.80(983.61-1, 691.78)	598.32(493.61-721.63)	-2.95(-3.26, -2.65)	-0.94(-1.10, -0.78)	-5.06(-5.91, -4.20)	-2.71(-2.97, -2.44)
Sexually transmitted infections excluding HIV	14.02(9.04-21.10)	7.83(4.48-13.06)	-1.86(-2.31, -1.41)	-3.09(-4.38, -1.79)	-1.59(-1.83, -1.36)	-0.90(-1.26, -0.55)
Soft tissue and other extraosseous sarcomas	43.38(28.36-56.65)	27.39(18.15-37.37)	-1.56(-1.91, -1.21)	-0.95(-1.22, -0.68)	-2.29(-3.24, -1.33)	-1.64(-1.80, -1.48)

Stomach cancer	5, 579.96(4, 829.70-6, 827.59)	3, 113.27(2, 425.01-3, 846.23)	-1.95(-2.30, -1.60)	-0.91(-1.23, -0.59)	-2.47(-3.43, -1.51)	-2.79(-2.98, -2.59)
Stroke	38, 046.68(33, 698.30-42, 593.03)	22, 555.13(18, 958.20-26, 128.32)	-1.71(-1.97, -1.46)	-0.87(-1.16, -0.59)	-2.28(-2.93, -1.62)	-2.21(-2.37, -2.05)
Testicular cancer	6.88(5.39-8.58)	6.57(4.78-8.76)	-0.06(-0.51, 0.39)	1.38(0.80, 1.96)	-2.32(-2.67, -1.97)	0.86(-0.11, 1.84)
Tetanus	32.10(15.03-50.29)	1.21(0.39-3.02)	-10.21(-11.09, -9.32)	-10.48(-12.58, -8.34)	-9.83(-10.71, -8.95)	-10.67(-11.06, -10.27)
Thyroid cancer	74.88(62.41-87.13)	70.13(54.07-87.13)	-0.21(-0.37, -0.04)	-0.51(-0.75, -0.26)	0.93(0.65, 1.22)	-1.37(-1.68, -1.06)
Tracheal, bronchus and lung cancer	4, 342.79(3, 766.33-4, 955.02)	5, 920.39(4, 784.99-7, 161.02)	1.11(0.82, 1.39)	2.16(1.50, 2.83)	1.00(0.75, 1.24)	-0.07(-0.30, 0.16)
Trachoma	7.68(4.15-13.13)	0.78(0.38-1.51)	-7.06(-7.76, -6.36)	-4.27(-5.54, -2.98)	-9.78(-11.36, -8.16)	-7.72(-8.07, -7.38)
Tuberculosis	2, 390.86(1, 999.44-2, 818.34)	332.23(266.06-418.11)	-6.16(-6.41, -5.90)	-5.89(-6.23, -5.55)	-7.15(-7.73, -6.57)	-5.74(-5.97, -5.51)
Typhoid and paratyphoid	0.95(0.36-1.94)	0.35(0.15-0.70)	-3.14(-3.29, -2.98)	-4.44(-4.80, -4.07)	-2.27(-2.52, -2.03)	-2.61(-2.76, -2.47)
Upper digestive system diseases	1, 620.31(1, 391.27-1, 867.42)	587.28(485.31-721.96)	-3.24(-3.43, -3.05)	-3.82(-4.02, -3.62)	-3.15(-3.60, -2.69)	-3.22(-3.37, -3.07)
Upper respiratory infections	250.55(60.24-368.32)	35.09(21.36-61.73)	-6.26(-6.49, -6.02)	-4.96(-5.26, -4.66)	-10.21(-10.58, -9.85)	-2.44(-2.84, -2.05)
Urinary diseases and male infertility	486.18(346.90-654.04)	361.59(249.66-511.49)	-0.94(-1.10, -0.78)	-1.11(-1.33, -0.90)	-1.07(-1.47, -0.68)	-0.83(-0.95, -0.71)
Urticaria	34.01(20.28-53.10)	33.20(19.86-52.16)	-0.08(-0.10, -0.06)	-0.09(-0.13, -0.04)	-0.11(-0.11, -0.10)	-0.04(-0.05, -0.03)
Uterine cancer	127.53(94.35-158.57)	67.73(50.55-96.93)	-2.00(-2.29, -1.72)	-0.68(-0.87, -0.48)	-2.78(-3.15, -2.40)	-2.25(-3.02, -1.48)
Varicella and herpes zoster	36.98(28.34-46.84)	12.84(8.22-19.67)	-3.41(-3.55, -3.27)	-3.95(-4.08, -3.81)	-4.95(-5.25, -4.64)	-1.34(-1.51, -1.17)
Vascular intestinal disorders	16.66(12.04-19.89)	7.29(5.95-8.85)	-2.67(-2.89, -2.45)	-0.62(-1.05, -0.18)	-4.51(-4.76, -4.26)	-2.81(-3.14, -2.47)
Viral skin diseases	39.35(25.35-58.82)	40.25(25.80-60.35)	0.07(0.06, 0.08)	0.10(0.08, 0.11)	0.13(0.11, 0.15)	-0.02(-0.05, 0.00)
Vitamin A deficiency	1.65(0.94-2.61)	1.60(0.91-2.62)	-0.07(-0.24, 0.10)	0.67(0.28, 1.07)	-0.08(-0.32, 0.16)	-0.93(-1.11, -0.75)
**80-84 years**						
Acne vulgaris	3.45(2.20-5.32)	4.03(2.53-6.28)	0.51(0.49, 0.52)	0.52(0.49, 0.56)	0.59(0.57, 0.61)	0.40(0.38, 0.43)
Acute glomerulonephritis	102.29(73.00-141.57)	40.73(26.88-53.80)	-2.94(-3.39, -2.50)	-3.45(-4.21, -2.69)	-1.81(-2.07, -1.54)	-3.88(-5.03, -2.71)
Acute hepatitis	157.63(125.96-195.61)	14.50(11.12-18.36)	-7.30(-8.02, -6.57)	-7.58(-8.03, -7.13)	-10.45(-12.52, -8.33)	-3.70(-4.00, -3.40)

Adverse effects of medical treatment	69.07(43.06-89.23)	20.77(16.46-28.12)	-3.92(-4.36, -3.48)	-3.88(-4.20, -3.57)	-5.41(-6.69, -4.12)	-2.45(-2.67, -2.22)
Age-related and other hearing loss	3, 855.13(2, 730.12-5, 155.67)	4, 062.46(2, 933.11-5, 411.46)	0.20(0.15, 0.25)	-0.51(-0.59, -0.43)	0.71(0.60, 0.82)	0.31(0.27, 0.35)
Alcohol use disorders	81.27(57.13-111.18)	101.61(71.18-137.98)	0.65(0.41, 0.89)	0.66(0.43, 0.89)	2.54(2.23, 2.85)	-1.37(-1.98, -0.76)
Alopecia areata	4.60(2.97-6.67)	4.44(2.88-6.46)	-0.11(-0.12, -0.11)	-0.11(-0.12, -0.11)	-0.11(-0.12, -0.11)	-0.11(-0.12, -0.11)
Alzheimer's disease and other dementias	10, 226.14(4, 350.56-23, 760.52)	10, 737.28(4, 961.95-23, 032.81)	0.10(0.00, 0.20)	0.01(-0.03, 0.05)	-0.19(-0.44, 0.07)	0.54(0.35, 0.73)
Animal contact	38.49(29.79-45.89)	12.26(9.94-14.85)	-3.64(-3.93, -3.35)	-2.74(-3.44, -2.05)	-5.48(-5.73, -5.22)	-2.29(-2.74, -1.85)
Anxiety disorders	517.60(350.75-728.18)	517.39(356.71-725.20)	-0.04(-0.29, 0.20)	0.82(0.56, 1.08)	-1.00(-1.36, -0.65)	0.21(-0.36, 0.79)
Aortic aneurysm	38.81(31.41-46.23)	53.56(43.00-65.47)	1.09(0.75, 1.43)	0.84(0.72, 0.97)	2.39(1.79, 3.00)	0.25(-0.22, 0.72)
Appendicitis	74.27(51.08-93.70)	17.89(13.41-23.93)	-4.51(-4.76, -4.26)	-3.56(-3.89, -3.22)	-5.96(-6.37, -5.55)	-3.90(-4.27, -3.53)
Asthma	1, 385.48(1, 058.65-1, 947.11)	448.80(361.65-536.95)	-3.58(-3.84, -3.31)	-2.39(-2.60, -2.18)	-5.02(-5.70, -4.34)	-3.27(-3.62, -2.92)
Atrial fibrillation and flutter	1, 364.04(1, 091.48-1, 722.80)	1, 269.30(1, 015.76-1, 573.52)	-0.19(-0.41, 0.03)	-0.21(-0.31, -0.11)	-0.31(-0.45, -0.18)	0.08(-0.46, 0.63)
Attention-deficit/hyperactivity disorder	0.02(0.00-0.09)	0.02(0.00-0.08)	-0.51(-0.65, -0.37)	-1.61(-1.75, -1.47)	-0.21(-0.60, 0.19)	0.23(0.15, 0.32)
Autism spectrum disorders	41.59(27.96-59.91)	61.04(41.72-85.70)	1.26(1.23, 1.28)	1.20(1.16, 1.24)	1.63(1.61, 1.65)	0.87(0.82, 0.93)
Bacterial skin diseases	162.26(121.81-198.15)	23.59(15.83-40.87)	-5.99(-6.59, -5.40)	-3.36(-3.61, -3.11)	-13.18(-14.71, -11.63)	-1.26(-2.10, -0.41)
Bipolar disorder	36.01(22.86-51.52)	35.51(22.62-51.53)	-0.04(-0.08, -0.01)	-0.10(-0.14, -0.05)	0.02(-0.07, 0.12)	-0.04(-0.06, -0.02)
Bladder cancer	620.87(482.39-715.14)	486.31(392.21-599.78)	-0.88(-1.17, -0.59)	-0.64(-1.33, 0.06)	-1.38(-1.65, -1.10)	-0.53(-0.82, -0.23)
Blindness and vision loss	2, 367.54(1, 680.40-3, 261.19)	1, 883.65(1, 322.90-2, 698.90)	-0.69(-0.86, -0.53)	0.80(0.30, 1.31)	-1.40(-1.47, -1.33)	-1.40(-1.47, -1.33)
Brain and central nervous system cancer	213.54(162.59-275.76)	267.63(186.15-336.95)	0.77(0.63, 0.90)	0.96(0.63, 1.29)	1.45(1.36, 1.55)	-0.17(-0.38, 0.04)
Breast cancer	355.02(296.58-412.04)	325.34(257.62-397.36)	-0.27(-0.77, 0.24)	0.70(0.18, 1.21)	-0.52(-0.70, -0.33)	-0.42(-1.57, 0.75)
Cardiomyopathy and myocarditis	188.84(152.00-304.88)	252.84(190.79-307.25)	1.14(0.83, 1.46)	5.35(4.82, 5.90)	0.08(-0.51, 0.68)	-2.27(-2.74, -1.80)
Cervical cancer	250.77(205.09-297.81)	177.87(133.94-222.68)	-1.11(-1.60, -0.62)	-1.43(-1.78, -1.08)	-0.73(-2.12, 0.68)	-1.55(-1.80, -1.29)
Chronic kidney disease	2, 602.13(2, 232.39-3, 085.19)	2, 417.65(1, 984.55-2, 823.13)	-0.25(-0.48, -0.03)	-0.59(-1.10, -0.08)	0.59(0.38, 0.81)	-0.86(-1.21, -0.51)

Chronic obstructive pulmonary disease	52, 076.81(44, 116.18-57, 392.23)	19, 571.69(16, 166.98-22, 626.60)	-3.17(-3.55, -2.79)	-2.10(-2.65, -1.56)	-4.57(-4.82, -4.32)	-2.48(-3.58, -1.36)
Cirrhosis and other chronic liver diseases	1, 661.03(1, 402.84-1, 954.64)	783.27(622.71-956.05)	-2.43(-2.83, -2.03)	-2.37(-2.78, -1.96)	-2.77(-3.45, -2.08)	-2.20(-2.98, -1.41)
Colon and rectum cancer	1, 930.20(1, 666.26-2, 185.16)	2, 086.56(1, 692.15-2, 428.47)	0.29(-0.12, 0.70)	0.26(-0.07, 0.59)	0.97(0.78, 1.17)	-0.15(-1.10, 0.80)
Conflict and terrorism	0.05(0.03-0.08)	0.09(0.06-0.13)	2.07(1.14, 3.01)	9.68(7.28, 12.12)	-1.89(-2.73, -1.04)	-1.89(-2.73, -1.04)
Congenital birth defects	21.93(14.87-31.58)	22.71(15.52-32.79)	0.06(-0.17, 0.30)	1.36(0.86, 1.86)	-0.15(-0.55, 0.25)	-1.25(-1.34, -1.15)
Cystic echinococcosis	2.81(1.81-4.03)	0.90(0.60-1.29)	-3.60(-3.91, -3.30)	-4.02(-4.41, -3.62)	-3.63(-4.10, -3.17)	-3.25(-3.73, -2.77)
Cysticercosis	82.84(41.03-145.00)	41.13(17.97-75.92)	-2.13(-2.53, -1.72)	-1.90(-2.25, -1.54)	-3.92(-4.12, -3.73)	-0.17(-1.48, 1.16)
Decubitus ulcer	15.21(8.55-37.36)	40.94(22.37-53.36)	4.39(3.18, 5.62)	13.00(11.46, 14.58)	3.26(1.08, 5.48)	-2.78(-4.88, -0.63)
Dengue	0.10(0.05-0.16)	0.05(0.02-0.08)	-2.74(-3.32, -2.15)	-5.02(-6.08, -3.96)	-0.66(-1.41, 0.11)	-3.29(-3.68, -2.91)
Depressive disorders	827.11(572.01-1, 144.29)	948.87(656.40-1, 321.77)	0.44(0.39, 0.49)	1.17(1.08, 1.26)	0.50(0.48, 0.52)	-0.45(-0.57, -0.33)
Dermatitis	99.62(54.25-177.10)	98.43(53.37-175.90)	-0.04(-0.05, -0.03)	-0.01(-0.03, 0.02)	-0.03(-0.04, -0.03)	-0.08(-0.10, -0.06)
Diabetes mellitus	2, 257.28(1, 909.78-2, 668.47)	2, 823.47(2, 364.41-3, 352.10)	0.70(0.36, 1.05)	1.92(1.79, 2.05)	0.62(-0.40, 1.65)	-0.53(-0.72, -0.34)
Diarrheal diseases	472.50(211.95-714.28)	41.00(25.80-73.69)	-7.69(-7.92, -7.46)	-8.58(-8.84, -8.31)	-9.30(-9.67, -8.93)	-4.62(-5.05, -4.19)
Dietary iron deficiency	657.48(445.55-899.36)	300.27(206.44-420.50)	-2.55(-2.68, -2.42)	-1.42(-1.80, -1.05)	-3.88(-4.01, -3.74)	-2.20(-2.33, -2.06)
Drowning	318.73(274.96-364.13)	224.45(188.30-263.05)	-1.12(-1.41, -0.84)	-0.60(-1.05, -0.14)	-0.55(-1.00, -0.09)	-2.80(-2.98, -2.61)
Drug use disorders	136.88(109.28-168.67)	45.11(35.06-56.05)	-3.65(-3.97, -3.32)	-2.26(-2.67, -1.85)	-6.91(-7.29, -6.53)	-0.85(-1.58, -0.12)
Encephalitis	13.12(10.83-16.14)	8.38(6.76-10.51)	-1.39(-1.76, -1.01)	-0.32(-0.90, 0.27)	-1.32(-1.71, -0.93)	-2.65(-3.44, -1.84)
Endocarditis	40.45(20.99-54.61)	14.63(10.93-21.33)	-3.18(-3.41, -2.95)	-0.79(-1.12, -0.45)	-6.81(-7.24, -6.37)	-0.86(-1.18, -0.54)
Endocrine, metabolic, blood, and immune disorders	350.99(252.14-489.88)	333.13(233.67-456.85)	-0.11(-0.48, 0.26)	0.67(0.29, 1.05)	-0.69(-1.31, -0.07)	-0.07(-0.86, 0.73)
Environmental heat and cold exposure	120.59(48.37-157.25)	46.19(20.75-64.84)	-3.04(-3.46, -2.62)	-3.48(-3.99, -2.98)	-4.04(-5.16, -2.91)	-1.44(-1.64, -1.23)
Esophageal cancer	2, 542.31(2, 117.07-2, 900.74)	1, 966.07(1, 596.11-2, 360.14)	-0.78(-1.15, -0.41)	0.34(-0.21, 0.90)	-1.43(-1.80, -1.07)	-1.40(-2.21, -0.59)

Exposure to forces of nature	14.36(12.98-16.01)	7.34(5.77-9.49)	0.27(-4.38, 5.15)	0.27(-4.38, 5.15)	0.27(-4.38, 5.15)	0.27(-4.38, 5.15)
Exposure to mechanical forces	95.25(75.00-129.40)	109.05(80.08-144.03)	0.51(0.08, 0.93)	0.33(-0.10, 0.77)	2.84(2.56, 3.12)	-1.98(-3.22, -0.73)
Eye cancer	8.10(4.55-10.87)	6.20(3.08-8.34)	-0.80(-1.33, -0.27)	-0.42(-0.99, 0.16)	-1.31(-1.52, -1.10)	-0.11(-1.30, 1.11)
Falls	2, 257.24(1, 866.36-2, 759.92)	2, 568.72(1, 931.23-3, 211.38)	0.50(0.35, 0.65)	0.44(0.17, 0.71)	-0.42(-0.71, -0.13)	1.38(1.19, 1.56)
Fire, heat, and hot substances	259.22(185.18-301.72)	118.70(91.71-144.05)	-2.69(-3.19, -2.18)	-3.67(-4.01, -3.32)	-1.29(-2.77, 0.23)	-3.00(-3.23, -2.77)
Food-borne trematodiases	125.36(55.04-247.36)	65.48(33.10-114.95)	-2.18(-2.82, -1.53)	-3.48(-4.53, -2.42)	-1.89(-2.19, -1.60)	-1.74(-3.20, -0.25)
Foreign body	81.50(62.65-96.86)	84.75(54.75-101.92)	0.15(-0.22, 0.51)	-0.14(-0.60, 0.32)	0.37(0.18, 0.56)	0.32(-0.64, 1.28)
Fungal skin diseases	66.60(26.56-137.22)	65.81(26.10-135.81)	-0.04(-0.05, -0.03)	-0.03(-0.05, -0.02)	-0.04(-0.04, -0.03)	-0.05(-0.06, -0.04)
Gallbladder and biliary diseases	929.95(639.75-1, 187.66)	636.28(461.27-891.82)	-1.24(-1.44, -1.03)	-1.37(-1.43, -1.30)	-1.12(-1.64, -0.61)	-1.06(-1.38, -0.74)
Gallbladder and biliary tract cancer	338.97(252.34-431.61)	292.77(198.51-365.42)	-0.43(-0.96, 0.10)	0.12(-0.02, 0.26)	0.12(-0.02, 0.26)	-1.26(-2.50, 0.00)
Gout	82.32(46.75-132.57)	113.46(64.16-182.43)	1.11(1.00, 1.22)	-0.58(-0.71, -0.44)	2.30(2.10, 2.49)	1.47(1.26, 1.67)
Gynecological diseases	146.98(83.71-234.69)	109.73(70.46-165.94)	-0.93(-1.01, -0.84)	-0.48(-0.55, -0.41)	-2.00(-2.24, -1.76)	-0.38(-0.42, -0.33)
Headache disorders	289.77(79.62-614.75)	300.93(80.55-646.21)	0.13(0.10, 0.16)	-0.12(-0.20, -0.05)	0.46(0.43, 0.49)	-0.06(-0.07, -0.05)
Hemoglobinopathies and hemolytic anemias	304.28(226.41-394.59)	166.81(129.27-213.89)	-1.94(-2.04, -1.84)	-1.70(-1.86, -1.54)	-2.35(-2.40, -2.29)	-1.65(-1.94, -1.37)
HIV/AIDS	4.83(1.02-23.75)	47.35(24.28-83.39)	6.91(2.31, 11.71)	13.02(-0.54, 28.44)	-1.78(-3.54, 0.01)	10.53(6.43, 14.78)
Hodgkin lymphoma	36.92(15.18-50.20)	12.50(7.12-16.39)	-3.50(-3.87, -3.12)	-2.67(-3.24, -2.10)	-5.47(-6.12, -4.81)	-1.78(-2.38, -1.18)
Hypertensive heart disease	8, 691.05(6, 385.93-10, 200.18)	4, 278.46(2, 949.61-5, 522.82)	-2.24(-2.42, -2.07)	-3.64(-3.82, -3.46)	-2.05(-2.48, -1.63)	-0.64(-0.86, -0.42)
Idiopathic developmental intellectual disability	6.07(2.36-11.81)	4.05(1.37-8.14)	-1.32(-1.41, -1.22)	1.05(0.81, 1.29)	-2.71(-2.77, -2.65)	-2.03(-2.21, -1.86)
Idiopathic epilepsy	137.07(88.98-202.88)	111.87(65.82-173.36)	-0.53(-0.69, -0.38)	-0.03(-0.14, 0.09)	-1.97(-2.03, -1.90)	0.65(0.15, 1.16)
Inflammatory bowel disease	128.74(72.15-172.61)	60.56(46.12-84.81)	-2.40(-2.64, -2.15)	-1.08(-1.57, -0.58)	-3.95(-4.18, -3.72)	-1.99(-2.39, -1.59)
Inguinal, femoral, and abdominal hernia	40.65(31.44-52.51)	22.92(18.05-29.27)	-1.81(-2.34, -1.27)	-0.99(-1.31, -0.68)	-1.84(-2.34, -1.34)	-2.37(-3.57, -1.17)

Interpersonal violence	126.52(100.14-156.68)	69.49(54.38-87.97)	-1.94(-2.01, -1.87)	-1.51(-1.61, -1.40)	-2.59(-2.65, -2.54)	-1.55(-1.74, -1.35)
Interstitial lung disease and pulmonary sarcoidosis	72.03(55.04-114.29)	86.53(55.72-114.89)	0.60(0.47, 0.73)	-0.74(-0.89, -0.60)	3.65(3.29, 4.01)	-1.17(-1.27, -1.08)
Intestinal nematode infections	55.05(29.51-86.90)	1.29(0.71-2.09)	-11.57(-11.94, -11.19)	-12.14(-12.80, -11.48)	-15.41(-16.05, -14.77)	-5.94(-6.44, -5.43)
Invasive Non-typhoidal Salmonella (iNTS)	2.12(0.55-5.27)	1.31(0.31-3.36)	-1.56(-1.77, -1.35)	-1.17(-1.75, -0.58)	-2.06(-2.17, -1.95)	-1.32(-1.59, -1.05)
Iodine deficiency	14.17(7.69-23.81)	14.97(6.92-27.61)	0.16(0.02, 0.31)	-0.69(-0.84, -0.54)	1.44(1.13, 1.75)	-0.77(-0.97, -0.57)
Ischemic heart disease	17, 303.31(15, 209.95-19, 313.68)	22, 839.92(19, 233.34-26, 336.36)	0.90(0.52, 1.29)	0.99(0.42, 1.56)	2.56(1.86, 3.26)	-1.21(-1.78, -0.63)
Kidney cancer	121.23(105.85-136.02)	157.77(128.59-186.82)	0.75(0.37, 1.14)	0.99(0.18, 1.81)	1.50(1.04, 1.96)	-0.42(-0.84, -0.01)
Larynx cancer	171.97(140.13-200.63)	127.15(103.74-152.43)	-1.02(-1.40, -0.64)	-1.42(-1.53, -1.30)	-0.29(-0.86, 0.28)	-1.05(-1.85, -0.24)
Leishmaniasis	0.61(0.07-1.62)	0.23(0.05-0.59)	-2.95(-3.62, -2.28)	-5.01(-5.25, -4.77)	-2.75(-4.53, -0.94)	-0.48(-1.22, 0.27)
Leprosy	0.26(0.16-0.41)	0.08(0.05-0.12)	-3.85(-3.92, -3.77)	-3.55(-3.63, -3.46)	-5.26(-5.35, -5.16)	-2.44(-2.64, -2.24)
Leukemia	263.17(210.08-318.55)	211.58(161.40-275.12)	-0.71(-0.92, -0.49)	-0.01(-0.51, 0.49)	-0.91(-1.09, -0.73)	-1.34(-1.50, -1.18)
Lip and oral cavity cancer	130.85(113.47-148.07)	141.90(116.30-168.74)	0.21(-0.18, 0.61)	0.14(-0.61, 0.90)	1.12(0.53, 1.73)	-0.44(-1.03, 0.15)
Liver cancer	680.02(576.58-792.50)	840.89(695.05-1, 026.86)	0.66(-0.19, 1.52)	0.16(-1.67, 2.02)	3.42(2.88, 3.96)	-2.36(-4.12, -0.56)
Low back pain	2, 272.04(1, 490.14-3, 326.80)	1, 813.98(1, 183.26-2, 650.41)	-0.71(-0.77, -0.66)	-1.34(-1.50, -1.17)	-0.41(-0.43, -0.39)	-0.41(-0.43, -0.39)
Lower extremity peripheral arterial disease	118.87(62.85-204.48)	113.56(64.81-192.21)	-0.19(-0.26, -0.11)	0.28(0.10, 0.45)	-0.31(-0.37, -0.25)	-0.51(-0.61, -0.41)
Lower respiratory infections	6, 148.16(4, 751.86-7, 034.02)	2, 435.10(1, 964.32-2, 976.36)	-3.22(-3.54, -2.89)	-2.47(-3.06, -1.88)	-4.12(-4.62, -3.62)	-2.87(-3.24, -2.49)
Malignant neoplasm of bone and articular cartilage	55.46(34.55-100.66)	101.62(61.99-136.31)	2.21(1.90, 2.52)	4.11(3.58, 4.64)	3.57(2.88, 4.26)	-1.74(-1.99, -1.48)
Malignant skin melanoma	32.50(23.46-47.54)	32.82(16.73-42.83)	-0.06(-0.48, 0.37)	0.87(-0.35, 2.09)	-0.50(-0.71, -0.29)	-0.50(-0.71, -0.29)
Meningitis	110.21(94.88-127.06)	31.89(26.65-37.25)	-3.90(-4.48, -3.32)	-5.64(-6.59, -4.69)	-3.22(-4.43, -2.00)	-2.23(-3.04, -1.41)
Mesothelioma	13.14(10.50-15.05)	15.41(12.47-18.34)	0.40(0.09, 0.70)	-0.69(-1.03, -0.35)	3.29(2.48, 4.11)	-1.54(-1.84, -1.23)

Motor neuron disease	3.89(2.62-5.07)	6.46(4.31-8.34)	1.54(1.25, 1.85)	3.21(2.76, 3.66)	-0.35(-0.96, 0.27)	2.42(2.21, 2.62)
Multiple myeloma	19.03(11.92-42.19)	65.88(41.25-85.72)	4.56(3.20, 5.94)	13.09(10.07, 16.20)	0.41(-1.58, 2.44)	1.53(1.22, 1.84)
Multiple sclerosis	0.70(0.46-0.97)	1.05(0.80-1.42)	1.39(1.17, 1.62)	2.31(2.23, 2.39)	1.04(0.93, 1.16)	1.04(0.31, 1.77)
Nasopharynx cancer	238.52(202.09-273.54)	101.10(83.42-119.82)	-2.89(-3.23, -2.54)	-2.09(-2.80, -1.37)	-4.36(-4.79, -3.93)	-1.79(-2.12, -1.45)
Neck pain	511.29(266.78-882.06)	522.50(283.90-890.30)	0.07(-0.06, 0.20)	-0.52(-0.77, -0.26)	0.90(0.65, 1.15)	-0.07(-0.20, 0.06)
Neonatal disorders	12.69(8.34-18.57)	14.69(10.52-19.96)	0.42(0.27, 0.57)	-1.01(-1.17, -0.85)	1.11(0.81, 1.40)	1.25(0.95, 1.54)
Neuroblastoma and other peripheral nervous cell tumors	0.95(0.57-1.54)	3.64(2.59-4.52)	4.36(4.09, 4.63)	3.00(2.61, 3.38)	6.82(6.28, 7.36)	3.45(3.03, 3.87)
Non-Hodgkin lymphoma	216.50(184.89-271.01)	211.69(169.72-256.53)	-0.13(-0.60, 0.35)	0.50(-0.05, 1.07)	0.26(-1.01, 1.55)	-1.07(-1.41, -0.72)
Non-melanoma skin cancer	108.04(88.72-136.45)	144.40(107.66-175.60)	0.83(0.48, 1.18)	1.33(0.73, 1.93)	2.15(1.55, 2.76)	-1.26(-1.66, -0.86)
Non-rheumatic valvular heart disease	72.08(53.26-101.13)	62.66(45.47-90.67)	-0.47(-0.67, -0.28)	0.09(0.00, 0.17)	-0.67(-0.78, -0.57)	-0.88(-1.51, -0.24)
Oral disorders	1, 246.25(788.82-1, 807.14)	1, 226.69(784.94-1, 748.10)	0.07(-0.21, 0.34)	-1.09(-1.43, -0.76)	0.40(0.05, 0.75)	1.18(0.63, 1.72)
Osteoarthritis	1, 150.63(556.23-2, 313.26)	1, 329.67(643.83-2, 670.63)	0.52(0.41, 0.64)	0.00(-0.15, 0.14)	0.36(0.14, 0.59)	1.28(1.12, 1.44)
Other cardiovascular and circulatory diseases	276.45(204.08-392.71)	226.05(165.58-319.00)	-0.65(-0.73, -0.58)	-0.51(-0.58, -0.44)	-0.89(-1.10, -0.68)	-0.57(-0.64, -0.50)
Other chronic respiratory diseases	31.94(23.84-56.35)	44.99(31.36-57.76)	1.27(0.93, 1.62)	2.59(1.81, 3.39)	4.33(3.82, 4.85)	-2.90(-3.23, -2.56)
Other digestive diseases	421.64(280.99-532.43)	140.54(113.37-172.13)	-3.52(-3.75, -3.30)	-4.61(-5.11, -4.11)	-4.64(-4.83, -4.46)	-0.77(-1.13, -0.40)
Other intestinal infectious diseases	1.06(0.03-2.96)	0.41(0.02-1.06)	-3.14(-3.75, -2.52)	-3.05(-3.71, -2.39)	-3.42(-5.00, -1.80)	-3.12(-3.40, -2.83)
Other malignant neoplasms	358.64(244.46-442.46)	260.18(206.34-318.51)	-1.09(-1.29, -0.88)	-1.78(-1.99, -1.57)	-0.33(-0.90, 0.24)	-1.02(-1.17, -0.87)
Other mental disorders	194.96(127.29-284.18)	196.90(128.27-287.48)	0.03(0.01, 0.05)	0.04(-0.02, 0.09)	0.05(0.04, 0.05)	-0.01(-0.02, 0.01)
Other musculoskeletal disorders	528.44(265.23-929.75)	482.50(225.31-905.84)	-0.48(-0.88, -0.08)	-0.09(-0.40, 0.23)	1.00(-0.17, 2.18)	-2.37(-2.65, -2.08)
Other neglected tropical diseases	26.61(18.80-35.10)	13.30(10.02-17.74)	-2.28(-2.37, -2.18)	-1.47(-1.58, -1.36)	-2.96(-3.10, -2.83)	-2.34(-2.56, -2.12)
Other neoplasms	33.69(21.29-57.91)	53.37(35.51-93.69)	1.49(1.39, 1.59)	1.27(1.15, 1.39)	3.26(3.02, 3.51)	-0.16(-0.25, -0.07)

Other neurological disorders	28.14(19.23-37.81)	83.78(63.93-104.62)	3.54(2.93, 4.16)	3.68(3.36, 4.01)	3.03(2.70, 3.36)	4.03(2.03, 6.06)
Other nutritional deficiencies	34.58(27.81-42.45)	34.89(27.22-44.09)	0.31(-0.32, 0.95)	-1.95(-2.93, -0.95)	3.08(2.06, 4.10)	-1.44(-1.83, -1.04)
Other pharynx cancer	42.48(36.25-51.18)	29.37(23.67-35.26)	-1.33(-1.76, -0.90)	-1.52(-2.45, -0.59)	-1.88(-2.32, -1.44)	-0.11(-0.41, 0.19)
Other sense organ diseases	201.29(99.96-387.49)	206.52(102.32-398.30)	0.09(0.05, 0.14)	0.11(0.03, 0.19)	0.08(-0.01, 0.16)	0.11(0.09, 0.12)
Other skin and subcutaneous diseases	112.94(59.23-195.24)	136.05(71.00-243.08)	0.61(0.56, 0.65)	0.32(0.20, 0.45)	0.92(0.88, 0.95)	0.55(0.51, 0.58)
Other transport injuries	62.06(50.59-74.05)	35.43(28.28-43.59)	-1.76(-2.19, -1.31)	-0.49(-1.32, 0.35)	-2.92(-3.18, -2.66)	-1.96(-2.96, -0.95)
Other unintentional injuries	117.81(89.38-154.88)	71.28(51.83-96.91)	-1.60(-1.76, -1.43)	-1.26(-1.49, -1.03)	-1.68(-1.83, -1.53)	-1.94(-2.32, -1.56)
Other unspecified infectious diseases	62.85(35.78-83.48)	28.41(18.11-39.18)	-2.54(-2.87, -2.20)	-2.41(-2.76, -2.05)	-4.60(-5.32, -3.87)	-0.31(-0.77, 0.15)
Otitis media	17.83(8.16-32.29)	12.88(5.95-24.09)	-1.06(-1.08, -1.03)	-1.07(-1.11, -1.02)	-1.15(-1.16, -1.14)	-0.92(-0.99, -0.85)
Ovarian cancer	97.38(76.15-125.96)	79.15(59.12-101.70)	-0.65(-1.57, 0.28)	1.33(0.25, 2.42)	-1.67(-2.10, -1.25)	-0.59(-2.63, 1.49)
Pancreatic cancer	518.80(441.68-610.10)	703.00(566.79-829.72)	1.05(0.71, 1.39)	1.60(1.52, 1.68)	1.60(1.52, 1.68)	0.21(-0.60, 1.03)
Pancreatitis	125.03(92.16-167.81)	93.98(71.79-122.82)	-0.91(-1.13, -0.68)	-0.94(-1.26, -0.62)	-0.36(-0.58, -0.14)	-1.38(-1.85, -0.90)
Paralytic ileus and intestinal obstruction	392.75(312.73-507.36)	184.29(144.69-243.38)	-2.47(-2.72, -2.22)	-1.95(-2.28, -1.62)	-2.19(-2.71, -1.66)	-3.44(-3.66, -3.23)
Parkinson's disease	1, 799.32(1, 541.48-2, 029.36)	1, 832.59(1, 538.07-2, 141.60)	0.05(-0.08, 0.18)	0.41(0.12, 0.71)	-0.14(-0.28, -0.01)	-0.14(-0.28, -0.01)
Pneumoconiosis	126.05(102.90-153.15)	80.50(63.31-99.81)	-1.47(-1.89, -1.06)	-1.12(-1.65, -0.59)	-1.68(-2.27, -1.10)	-1.73(-2.51, -0.95)
Poisonings	89.21(66.10-174.59)	95.11(54.14-120.93)	0.32(-0.13, 0.78)	0.25(-0.71, 1.21)	3.46(2.76, 4.16)	-3.38(-3.87, -2.88)
Police conflict and executions	1.45(0.29-2.32)	2.00(0.67-3.61)	0.66(-0.30, 1.62)	-2.58(-3.17, -2.00)	0.01(-2.78, 2.88)	5.03(4.54, 5.51)
Prostate cancer	402.50(312.10-601.77)	492.87(329.26-667.67)	0.74(0.37, 1.11)	2.48(1.69, 3.28)	-0.57(-0.87, -0.27)	0.58(-0.20, 1.36)
Protein-energy malnutrition	438.04(375.12-500.54)	118.53(97.38-145.07)	-4.05(-4.36, -3.74)	-6.79(-7.50, -6.07)	-4.55(-4.81, -4.29)	-0.21(-0.53, 0.10)
Pruritus	25.95(11.36-53.70)	29.36(12.82-61.16)	0.40(0.39, 0.41)	0.37(0.35, 0.38)	0.47(0.47, 0.48)	0.35(0.32, 0.37)
Psoriasis	56.56(41.46-73.68)	73.63(53.88-95.63)	0.87(0.83, 0.90)	1.35(1.27, 1.43)	0.68(0.65, 0.72)	0.58(0.56, 0.61)
Pulmonary Arterial Hypertension	108.39(74.33-164.02)	81.73(54.46-101.66)	-1.04(-1.60, -0.48)	-1.99(-2.26, -1.72)	2.77(1.05, 4.51)	-3.70(-4.04, -3.36)

Rabies	0.97(0.47-1.53)	0.69(0.35-1.10)	-1.24(-2.46, -0.02)	-2.79(-4.55, -0.99)	7.16(6.09, 8.23)	-9.10(-11.93, -6.18)
Rheumatic heart disease	2, 812.04(2, 339.02-3, 413.67)	782.82(610.06-995.56)	-4.14(-4.53, -3.74)	-4.46(-5.01, -3.90)	-4.39(-4.82, -3.96)	-3.50(-4.38, -2.62)
Rheumatoid arthritis	192.24(158.04-235.41)	206.30(159.95-252.04)	0.24(0.07, 0.41)	0.27(-0.07, 0.62)	0.55(0.37, 0.73)	-0.32(-0.45, -0.20)
Road injuries	758.06(656.35-890.67)	738.24(632.21-867.51)	-0.11(-0.36, 0.15)	1.75(1.32, 2.18)	0.41(-0.08, 0.90)	-2.85(-3.14, -2.57)
Scabies	111.13(58.53-182.47)	111.09(58.46-182.41)	-0.01(-0.03, 0.02)	0.04(0.01, 0.06)	0.02(-0.04, 0.07)	-0.07(-0.09, -0.05)
Schistosomiasis	37.83(27.77-51.01)	7.13(4.88-10.85)	-5.28(-5.66, -4.91)	-5.73(-5.89, -5.56)	-6.75(-7.72, -5.77)	-3.05(-3.59, -2.51)
Schizophrenia	86.27(62.93-110.43)	91.48(67.06-113.88)	0.19(0.15, 0.22)	0.02(-0.01, 0.04)	0.24(0.17, 0.31)	0.36(0.28, 0.43)
Self-harm	1, 541.45(1, 013.60-1, 744.82)	657.80(542.43-783.63)	-2.78(-2.98, -2.58)	-0.73(-0.98, -0.49)	-4.49(-4.72, -4.26)	-2.86(-3.30, -2.42)
Sexually transmitted infections excluding HIV	12.72(8.24-19.18)	7.28(4.15-12.30)	-1.81(-2.12, -1.51)	-2.85(-3.75, -1.95)	-1.31(-1.42, -1.21)	-1.31(-1.42, -1.21)
Soft tissue and other extraosseous sarcomas	54.42(37.26-71.10)	37.35(26.83-52.21)	-1.19(-1.43, -0.96)	-0.54(-0.74, -0.33)	-1.90(-2.16, -1.64)	-1.02(-1.62, -0.42)
Stomach cancer	4, 771.79(4, 097.23-5, 885.04)	3, 036.79(2, 315.36-3, 655.90)	-1.41(-1.82, -1.01)	-0.72(-1.30, -0.14)	-1.55(-1.99, -1.11)	-2.22(-3.10, -1.33)
Stroke	47, 787.41(42, 188.29-53, 478.61)	31, 568.30(26, 580.05-36, 094.89)	-1.35(-1.57, -1.13)	-0.15(-0.52, 0.21)	-1.68(-1.91, -1.46)	-2.33(-2.63, -2.04)
Testicular cancer	8.16(6.54-9.74)	7.35(5.61-9.27)	-0.26(-0.82, 0.31)	1.59(0.38, 2.81)	-3.34(-3.90, -2.78)	1.43(0.45, 2.42)
Tetanus	26.11(9.91-40.12)	1.13(0.33-2.92)	-9.86(-10.76, -8.96)	-9.80(-11.92, -7.62)	-9.66(-10.56, -8.76)	-10.47(-10.86, -10.07)
Thyroid cancer	70.47(59.99-88.29)	66.09(50.18-79.55)	-0.19(-0.49, 0.11)	-0.19(-0.72, 0.35)	0.57(0.05, 1.09)	-0.77(-1.19, -0.35)
Tracheal, bronchus, and lung cancer	3, 501.80(3, 030.65-3, 991.35)	5, 512.36(4, 521.93-6, 602.21)	1.45(1.14, 1.76)	2.36(1.89, 2.83)	2.26(1.68, 2.84)	-0.51(-0.90, -0.11)
Trachoma	8.74(4.77-14.35)	0.97(0.45-1.73)	-7.09(-7.82, -6.35)	-4.15(-5.48, -2.82)	-10.17(-11.83, -8.48)	-7.42(-7.78, -7.06)
Tuberculosis	2, 370.63(1, 987.10-2, 817.68)	358.03(291.91-452.20)	-6.10(-6.44, -5.77)	-5.86(-6.30, -5.42)	-7.21(-7.97, -6.45)	-5.53(-5.80, -5.25)
Typhoid and paratyphoid	0.70(0.25-1.49)	0.27(0.11-0.53)	-2.86(-3.12, -2.61)	-3.81(-3.96, -3.66)	-1.97(-2.70, -1.24)	-2.70(-2.94, -2.46)
Upper digestive system diseases	1, 873.62(1, 608.01-2, 183.32)	772.17(640.23-930.43)	-2.93(-3.32, -2.53)	-3.08(-3.42, -2.74)	-2.75(-3.81, -1.68)	-3.42(-3.63, -3.22)
Upper respiratory infections	496.51(95.83-765.07)	44.30(26.77-79.28)	-7.66(-7.92, -7.40)	-4.42(-4.76, -4.09)	-13.15(-13.52, -12.78)	-3.83(-4.33, -3.32)

Urinary diseases and male infertility	523.51(374.10-678.07)	410.61(288.40-552.52)	-0.75(-0.96, -0.54)	-0.76(-1.13, -0.40)	-1.22(-1.40, -1.04)	-0.42(-0.80, -0.04)
Urticaria	29.73(17.71-44.84)	28.73(17.34-43.37)	-0.11(-0.13, -0.09)	-0.10(-0.11, -0.10)	-0.10(-0.11, -0.10)	-0.14(-0.21, -0.06)
Uterine cancer	113.00(83.47-144.45)	61.99(44.16-86.59)	-1.91(-2.18, -1.64)	-0.02(-0.18, 0.14)	-2.29(-2.63, -1.96)	-2.84(-3.50, -2.18)
Varicella and herpes zoster	67.35(51.86-84.56)	17.32(11.56-25.74)	-4.31(-4.43, -4.20)	-4.34(-4.40, -4.28)	-6.35(-6.59, -6.11)	-1.94(-2.12, -1.76)
Vascular intestinal disorders	21.00(13.72-25.54)	10.46(8.36-12.48)	-2.25(-2.43, -2.06)	-0.52(-0.82, -0.21)	-3.65(-3.89, -3.40)	-2.65(-2.89, -2.41)
Viral skin diseases	32.51(21.02-48.10)	33.37(21.52-49.36)	0.09(0.07, 0.10)	0.09(0.05, 0.12)	0.11(0.09, 0.14)	0.05(0.03, 0.07)
Vitamin A deficiency	1.52(0.86-2.45)	1.43(0.79-2.27)	-0.15(-0.22, -0.08)	0.50(0.40, 0.61)	-0.17(-0.25, -0.10)	-0.84(-0.95, -0.73)
**85-89 years**						
Acne vulgaris	3.45(2.20-5.32)	3.02(1.91-4.62)	0.46(0.44, 0.48)	0.42(0.38, 0.45)	0.57(0.53, 0.62)	0.39(0.37, 0.41)
Acute glomerulonephritis	102.29(73.00-141.57)	59.30(40.29-77.90)	-2.66(-3.31, -2.00)	-2.39(-3.42, -1.36)	-2.07(-2.59, -1.55)	-4.16(-5.36, -2.96)
Acute hepatitis	157.63(125.96-195.61)	16.63(13.11-20.88)	-7.37(-7.71, -7.03)	-6.43(-6.79, -6.06)	-10.82(-11.35, -10.29)	-4.96(-5.84, -4.07)
Adverse effects of medical treatment	69.07(43.06-89.23)	30.56(23.31-43.32)	-3.68(-4.23, -3.13)	-2.92(-3.20, -2.65)	-5.70(-7.12, -4.27)	-2.30(-2.95, -1.66)
Age-related and other hearing loss	3, 855.13(2, 730.12-5, 155.67)	4, 508.05(3, 269.33-5, 947.03)	0.09(0.04, 0.13)	-0.47(-0.58, -0.36)	0.53(0.47, 0.59)	0.14(0.11, 0.16)
Alcohol use disorders	81.27(57.13-111.18)	99.31(72.10-135.28)	0.78(0.60, 0.97)	1.27(0.99, 1.55)	1.77(1.51, 2.02)	-1.12(-1.43, -0.81)
Alopecia areata	4.60(2.97-6.67)	3.48(2.30-5.08)	-0.11(-0.15, -0.06)	-0.11(-0.15, -0.08)	-0.03(-0.17, 0.11)	-0.17(-0.19, -0.15)
Alzheimer's disease and other dementias	10, 226.14(4, 350.56-23, 760.52)	18, 653.89(8, 375.45-40, 667.66)	0.08(-0.05, 0.20)	0.01(-0.04, 0.05)	-0.24(-0.55, 0.08)	0.53(0.30, 0.76)
Animal contact	38.49(29.79-45.89)	13.50(10.78-16.17)	-3.58(-3.86, -3.30)	-2.15(-2.29, -2.01)	-5.84(-6.60, -5.07)	-2.49(-2.79, -2.20)
Anxiety disorders	517.60(350.75-728.18)	488.01(327.26-684.89)	0.01(-0.25, 0.26)	1.01(0.73, 1.28)	-0.84(-1.28, -0.39)	-0.07(-0.63, 0.49)
Aortic aneurysm	38.81(31.41-46.23)	71.55(56.85-87.80)	1.31(1.07, 1.55)	1.15(0.64, 1.66)	2.17(1.89, 2.45)	0.51(0.13, 0.89)
Appendicitis	74.27(51.08-93.70)	24.41(18.22-32.15)	-4.29(-4.59, -3.98)	-2.53(-3.03, -2.03)	-6.34(-6.80, -5.87)	-3.88(-4.34, -3.42)
Asthma	1, 385.48(1, 058.65-1, 947.11)	628.10(499.61-745.01)	-3.59(-4.18, -2.99)	-2.04(-3.13, -0.95)	-5.04(-6.21, -3.86)	-4.10(-4.44, -3.76)
Atrial fibrillation and flutter	1, 364.04(1, 091.48-1, 722.80)	2, 243.60(1, 782.45-2, 723.77)	-0.39(-0.74, -0.03)	0.13(-0.43, 0.70)	-1.14(-1.52, -0.76)	-0.19(-0.80, 0.41)

Attention-deficit/hyperactivity disorder	0.02(0.00-0.09)	0.00(0.00-0.00)	-0.71(-0.92, -0.50)	-2.00(-2.18, -1.82)	-0.22(-0.81, 0.38)	-0.03(-0.15, 0.09)
Autism spectrum disorders	41.59(27.96-59.91)	45.59(30.83-65.35)	1.60(1.55, 1.65)	1.50(1.47, 1.53)	2.07(1.97, 2.18)	1.19(1.10, 1.27)
Bacterial skin diseases	162.26(121.81-198.15)	50.35(31.91-94.50)	-6.13(-6.70, -5.55)	-3.44(-3.78, -3.10)	-12.84(-13.77, -11.91)	-0.56(-2.20, 1.11)
Bipolar disorder	36.01(22.86-51.52)	31.67(19.19-46.31)	-0.04(-0.12, 0.03)	-0.10(-0.33, 0.13)	-0.04(-0.07, -0.01)	0.03(0.00, 0.05)
Bladder cancer	620.87(482.39-715.14)	662.94(544.60-804.77)	-0.65(-1.12, -0.18)	0.19(-0.10, 0.48)	-1.93(-3.26, -0.57)	-0.29(-0.58, 0.00)
Blindness and vision loss	2, 367.54(1, 680.40-3, 261.19)	2, 179.34(1, 560.93-3, 049.57)	-0.70(-0.89, -0.52)	0.51(0.05, 0.96)	-1.48(-1.57, -1.40)	-1.00(-1.38, -0.61)
Brain and central nervous system cancer	213.54(162.59-275.76)	263.62(183.39-331.66)	0.95(0.73, 1.17)	1.54(1.16, 1.93)	1.10(0.79, 1.41)	-0.06(-0.42, 0.31)
Breast cancer	355.02(296.58-412.04)	369.18(278.61-455.04)	-0.22(-0.91, 0.46)	1.00(0.34, 1.67)	-0.81(-1.10, -0.53)	-0.27(-1.83, 1.32)
Cardiomyopathy and myocarditis	188.84(152.00-304.88)	458.92(330.25-564.54)	1.10(0.55, 1.65)	4.59(3.01, 6.20)	-0.35(-1.01, 0.31)	-1.36(-1.56, -1.17)
Cervical cancer	250.77(205.09-297.81)	170.28(123.43-217.47)	-1.06(-1.52, -0.60)	-0.79(-1.34, -0.24)	-1.53(-2.25, -0.80)	-1.09(-1.89, -0.30)
Chronic kidney disease	2, 602.13(2, 232.39-3, 085.19)	3, 500.55(2, 916.64-4, 095.08)	-0.19(-0.52, 0.14)	0.08(-0.21, 0.37)	-0.39(-1.30, 0.52)	-0.52(-0.68, -0.36)
Chronic obstructive pulmonary disease	52, 076.81(44, 116.18-57, 392.23)	30, 757.40(25, 595.96-35, 464.16)	-3.00(-3.36, -2.64)	-1.32(-1.49, -1.14)	-4.86(-5.74, -3.98)	-2.77(-3.40, -2.14)
Cirrhosis and other chronic liver diseases	1, 661.03(1, 402.84-1, 954.64)	874.14(699.13-1, 059.85)	-2.26(-2.60, -1.92)	-1.54(-2.09, -0.99)	-2.99(-3.46, -2.52)	-2.37(-2.91, -1.83)
Colon and rectum cancer	1, 930.20(1, 666.26-2, 185.16)	2, 379.84(1, 952.07-2, 801.03)	0.05(-0.39, 0.49)	0.50(0.06, 0.94)	-0.53(-1.70, 0.66)	-0.02(-0.26, 0.23)
Conflict and terrorism	0.05(0.03-0.08)	0.08(0.05-0.12)	3.90(1.41, 6.46)	14.89(13.05, 16.76)	-0.31(-7.04, 6.91)	-3.63(-5.58, -1.64)
Congenital birth defects	21.93(14.87-31.58)	20.80(13.73-30.63)	0.19(-0.07, 0.44)	1.29(0.91, 1.68)	0.20(-0.48, 0.87)	-0.97(-1.05, -0.88)
Cystic echinococcosis	2.81(1.81-4.03)	1.03(0.65-1.43)	-3.88(-4.39, -3.37)	-3.20(-3.84, -2.56)	-4.61(-5.31, -3.90)	-4.08(-4.74, -3.42)
Cysticercosis	82.84(41.03-145.00)	41.22(16.97-79.52)	-2.23(-2.60, -1.86)	-2.20(-2.35, -2.05)	-4.02(-4.72, -3.31)	-0.10(-1.04, 0.85)
Decubitus ulcer	15.21(8.55-37.36)	102.50(56.94-131.70)	4.53(3.22, 5.85)	13.87(12.36, 15.39)	2.45(-0.10, 5.07)	-2.44(-4.57, -0.26)
Dengue	0.10(0.05-0.16)	0.12(0.06-0.19)	-2.66(-3.69, -1.62)	-7.47(-10.16, -4.70)	0.51(-0.14, 1.16)	-2.23(-2.55, -1.90)
Depressive disorders	827.11(572.01-1, 144.29)	935.32(667.36-1, 289.31)	0.26(0.20, 0.31)	0.72(0.62, 0.82)	0.37(0.36, 0.39)	-0.41(-0.55, -0.26)
Dermatitis	99.62(54.25-177.10)	87.34(51.93-140.34)	-0.04(-0.05, -0.03)	-0.03(-0.04, -0.02)	0.01(-0.01, 0.02)	-0.10(-0.12, -0.07)

Diabetes mellitus	2, 257.28(1, 909.78-2, 668.47)	3, 083.35(2, 617.45-3, 594.73)	0.72(0.45, 0.99)	2.29(2.14, 2.43)	-0.17(-0.95, 0.62)	-0.10(-0.26, 0.06)
Diarrheal diseases	472.50(211.95-714.28)	66.77(41.49-118.96)	-7.63(-7.97, -7.28)	-8.21(-8.39, -8.03)	-9.36(-10.17, -8.55)	-4.97(-5.48, -4.46)
Dietary iron deficiency	657.48(445.55-899.36)	354.63(243.08-504.06)	-2.17(-2.27, -2.07)	-0.67(-0.94, -0.40)	-3.32(-3.44, -3.20)	-2.37(-2.48, -2.26)
Drowning	318.73(274.96-364.13)	303.52(252.16-352.66)	-0.85(-1.25, -0.45)	-0.17(-0.72, 0.37)	-0.61(-1.18, -0.03)	-2.35(-3.07, -1.63)
Drug use disorders	136.88(109.28-168.67)	46.08(36.93-55.78)	-3.51(-3.86, -3.15)	-0.67(-1.09, -0.25)	-7.70(-8.19, -7.21)	-1.02(-1.75, -0.28)
Encephalitis	13.12(10.83-16.14)	10.46(8.07-12.88)	-1.32(-1.68, -0.96)	-0.27(-0.85, 0.33)	-1.46(-1.83, -1.09)	-2.51(-3.26, -1.74)
Endocarditis	40.45(20.99-54.61)	23.59(17.94-32.26)	-3.21(-3.47, -2.95)	0.01(-0.38, 0.40)	-8.10(-8.63, -7.57)	-0.06(-0.39, 0.27)
Endocrine, metabolic, blood, and immune disorders	350.99(252.14-489.88)	409.26(279.00-519.51)	-0.12(-0.28, 0.05)	0.05(-0.10, 0.20)	-0.91(-1.25, -0.58)	0.32(-0.02, 0.67)
Environmental heat and cold exposure	120.59(48.37-157.25)	75.27(33.28-103.08)	-2.98(-3.42, -2.54)	-3.09(-3.41, -2.76)	-4.11(-5.31, -2.90)	-1.86(-2.06, -1.66)
Esophageal cancer	2, 542.31(2, 117.07-2, 900.74)	2, 088.89(1, 706.44-2, 459.16)	-0.59(-0.95, -0.22)	0.97(0.44, 1.50)	-1.49(-1.95, -1.03)	-1.44(-2.12, -0.76)
Exposure to forces of nature	14.36(12.98-16.01)	8.19(6.43-10.56)	0.18(-4.66, 5.26)	0.18(-4.66, 5.26)	0.18(-4.66, 5.26)	0.18(-4.66, 5.26)
Exposure to mechanical forces	95.25(75.00-129.40)	127.80(90.89-163.75)	0.57(0.26, 0.88)	0.61(0.19, 1.03)	2.31(1.81, 2.81)	-2.00(-2.49, -1.50)
Eye cancer	8.10(4.55-10.87)	8.51(3.89-11.81)	-0.32(-0.76, 0.12)	0.38(-0.06, 0.82)	-1.39(-2.05, -0.73)	0.23(-0.76, 1.24)
Falls	2, 257.24(1, 866.36-2, 759.92)	4, 438.94(3, 235.46-5, 452.08)	0.64(0.43, 0.86)	0.90(0.62, 1.19)	-0.49(-0.96, -0.02)	1.19(0.94, 1.44)
Fire, heat, and hot substances	259.22(185.18-301.72)	173.03(131.08-210.41)	-2.37(-2.91, -1.82)	-2.90(-3.27, -2.53)	-1.05(-2.47, 0.38)	-2.99(-3.83, -2.13)
Food-borne trematodiases	125.36(55.04-247.36)	60.53(31.93-104.62)	-2.06(-2.78, -1.33)	-3.32(-4.47, -2.16)	-1.91(-2.24, -1.58)	-1.38(-3.03, 0.31)
Foreign body	81.50(62.65-96.86)	145.05(85.33-174.19)	0.53(0.05, 1.01)	1.01(0.62, 1.40)	0.21(-1.11, 1.54)	0.03(-0.21, 0.27)
Fungal skin diseases	66.60(26.56-137.22)	87.72(33.91-178.01)	-0.05(-0.05, -0.04)	-0.04(-0.05, -0.03)	-0.03(-0.03, -0.03)	-0.07(-0.08, -0.07)
Gallbladder and biliary diseases	929.95(639.75-1, 187.66)	887.60(684.46-1, 210.01)	-1.45(-1.71, -1.19)	-1.31(-1.72, -0.90)	-1.92(-2.20, -1.64)	-1.16(-1.61, -0.69)
Gallbladder and biliary tract cancer	338.97(252.34-431.61)	332.37(212.71-417.79)	-0.22(-0.60, 0.16)	0.43(-0.08, 0.94)	-0.74(-1.39, -0.09)	-0.35(-0.93, 0.24)
Gout	82.32(46.75-132.57)	109.74(68.60-170.43)	1.04(0.91, 1.18)	-0.46(-0.62, -0.29)	2.05(1.81, 2.30)	1.43(1.21, 1.65)

Gynecological diseases	146.98(83.71-234.69)	124.13(80.77-181.77)	-0.70(-0.75, -0.65)	-0.45(-0.49, -0.40)	-1.37(-1.50, -1.24)	-0.38(-0.41, -0.35)
Headache disorders	289.77(79.62-614.75)	267.39(74.91-575.53)	0.13(0.11, 0.15)	-0.13(-0.16, -0.11)	0.49(0.44, 0.53)	-0.09(-0.10, -0.07)
Hemoglobinopathies and hemolytic anemias	304.28(226.41-394.59)	201.58(154.76-256.20)	-1.63(-1.72, -1.55)	-1.09(-1.18, -0.99)	-2.17(-2.23, -2.12)	-1.62(-1.87, -1.37)
HIV/AIDS	4.83(1.02-23.75)	33.42(16.99-59.43)	6.75(2.18, 11.54)	13.36(-0.13, 28.66)	-1.75(-3.53, 0.05)	9.59(5.28, 14.08)
Hodgkin lymphoma	36.92(15.18-50.20)	12.56(7.29-16.84)	-3.18(-3.61, -2.75)	-1.65(-2.35, -0.95)	-6.23(-7.08, -5.38)	-1.75(-2.37, -1.12)
Hypertensive heart disease	8, 691.05(6, 385.93-10, 200.18)	7, 400.31(4, 907.47-9, 847.23)	-1.99(-2.50, -1.47)	-2.86(-3.10, -2.62)	-2.22(-3.71, -0.71)	-0.78(-1.25, -0.30)
Idiopathic developmental intellectual disability	6.07(2.36-11.81)	3.78(1.32-7.37)	-1.12(-1.25, -0.98)	1.26(0.93, 1.59)	-2.59(-2.69, -2.49)	-2.04(-2.26, -1.81)
Idiopathic epilepsy	137.07(88.98-202.88)	127.84(78.00-198.65)	-0.52(-0.73, -0.31)	0.00(-0.13, 0.14)	-1.87(-1.96, -1.79)	0.56(-0.13, 1.25)
Inflammatory bowel disease	128.74(72.15-172.61)	106.92(83.09-157.64)	-2.29(-2.68, -1.91)	0.09(-0.61, 0.80)	-4.75(-5.24, -4.25)	-2.06(-2.62, -1.50)
Inguinal, femoral, and abdominal hernia	40.65(31.44-52.51)	38.05(30.27-47.11)	-1.44(-1.83, -1.05)	0.02(-0.68, 0.72)	-2.35(-2.80, -1.90)	-2.25(-2.80, -1.70)
Interpersonal violence	126.52(100.14-156.68)	62.56(48.81-79.14)	-1.92(-2.07, -1.78)	-1.49(-1.62, -1.35)	-3.10(-3.26, -2.94)	-1.01(-1.46, -0.56)
Interstitial lung disease and pulmonary sarcoidosis	72.03(55.04-114.29)	103.01(63.39-137.23)	0.97(0.77, 1.17)	0.05(-0.24, 0.35)	3.82(3.38, 4.26)	-1.08(-1.26, -0.89)
Intestinal nematode infections	55.05(29.51-86.90)	1.17(0.64-1.88)	-11.41(-11.75, -11.08)	-12.16(-12.79, -11.52)	-14.97(-15.46, -14.47)	-5.88(-6.32, -5.44)
Invasive Non-typhoidal Salmonella (iNTS)	2.12(0.55-5.27)	1.31(0.30-3.44)	-1.60(-1.99, -1.21)	-1.12(-1.46, -0.79)	-2.01(-3.09, -0.93)	-2.01(-2.19, -1.83)
Iodine deficiency	14.17(7.69-23.81)	12.97(5.96-24.02)	0.11(-0.06, 0.28)	-0.85(-1.02, -0.68)	1.61(1.28, 1.94)	-0.99(-1.26, -0.72)
Ischemic heart disease	17, 303.31(15, 209.95-19, 313.68)	37, 646.30(31, 539.83-43, 320.61)	1.22(0.74, 1.70)	2.21(1.49, 2.94)	2.27(1.60, 2.94)	-1.56(-2.41, -0.69)
Kidney cancer	121.23(105.85-136.02)	209.61(173.56-249.57)	0.10(-0.27, 0.48)	1.26(0.62, 1.89)	-1.92(-2.62, -1.21)	0.89(0.32, 1.45)
Larynx cancer	171.97(140.13-200.63)	128.68(104.41-151.91)	-0.68(-1.15, -0.21)	0.35(0.09, 0.62)	-1.64(-2.98, -0.28)	-0.84(-1.12, -0.55)
Leishmaniasis	0.61(0.07-1.62)	0.16(0.04-0.41)	-3.73(-4.63, -2.82)	-4.98(-7.29, -2.60)	-3.85(-4.08, -3.62)	-2.16(-3.80, -0.50)
Leprosy	0.26(0.16-0.41)	0.08(0.05-0.12)	-3.85(-3.94, -3.76)	-3.16(-3.25, -3.07)	-5.57(-5.66, -5.48)	-2.53(-2.80, -2.26)

Leukemia	263.17(210.08-318.55)	214.01(165.91-279.43)	-0.60(-0.91, -0.30)	0.77(0.39, 1.15)	-1.54(-2.28, -0.78)	-1.26(-1.45, -1.07)
Lip and oral cavity cancer	130.85(113.47-148.07)	166.33(136.98-195.75)	0.61(0.25, 0.97)	1.25(0.25, 2.27)	0.31(0.11, 0.50)	0.31(0.11, 0.50)
Liver cancer	680.02(576.58-792.50)	823.45(665.28-991.09)	-0.09(-1.35, 1.20)	-0.84(-1.36, -0.31)	1.92(-1.80, 5.79)	-1.24(-2.22, -0.24)
Low back pain	2, 272.04(1, 490.14-3, 326.80)	1, 862.14(1, 225.67-2, 835.17)	-0.53(-0.56, -0.50)	-0.84(-0.89, -0.78)	-0.22(-0.28, -0.15)	-0.56(-0.59, -0.53)
Lower extremity peripheral arterial disease	118.87(62.85-204.48)	135.44(81.33-217.90)	-0.08(-0.27, 0.11)	0.52(0.42, 0.62)	-0.39(-0.95, 0.18)	-0.43(-0.52, -0.34)
Lower respiratory infections	6, 148.16(4, 751.86-7, 034.02)	4, 826.71(3, 986.10-5, 950.72)	-2.62(-3.16, -2.08)	-1.00(-1.25, -0.74)	-3.94(-5.50, -2.36)	-3.05(-3.35, -2.75)
Malignant neoplasm of bone and articular cartilage	55.46(34.55-100.66)	99.82(59.46-136.02)	2.22(1.94, 2.49)	4.50(4.00, 5.00)	3.39(2.84, 3.95)	-1.98(-2.26, -1.70)
Malignant skin melanoma	32.50(23.46-47.54)	40.52(22.20-53.10)	0.22(-0.23, 0.68)	1.63(0.34, 2.93)	-0.44(-0.69, -0.18)	-0.44(-0.69, -0.18)
Meningitis	110.21(94.88-127.06)	43.14(35.64-51.79)	-3.47(-3.97, -2.97)	-4.25(-5.01, -3.47)	-3.03(-4.17, -1.87)	-2.71(-3.35, -2.07)
Mesothelioma	13.14(10.50-15.05)	16.93(13.73-20.13)	0.50(0.13, 0.87)	0.31(-0.08, 0.70)	2.57(2.32, 2.83)	-2.01(-3.11, -0.89)
Motor neuron disease	3.89(2.62-5.07)	5.91(3.88-7.53)	1.71(1.41, 2.01)	3.42(3.06, 3.79)	-0.40(-0.89, 0.09)	2.67(2.06, 3.28)
Multiple myeloma	19.03(11.92-42.19)	66.17(40.34-85.68)	4.95(3.90, 6.01)	14.18(10.93, 17.51)	-0.16(-1.05, 0.73)	2.02(1.48, 2.55)
Multiple sclerosis	0.70(0.46-0.97)	0.99(0.74-1.32)	1.26(1.06, 1.47)	2.50(2.12, 2.89)	0.77(0.57, 0.96)	0.56(0.15, 0.97)
Nasopharynx cancer	238.52(202.09-273.54)	97.29(80.18-115.95)	-3.01(-3.39, -2.63)	-1.39(-2.15, -0.62)	-5.89(-6.50, -5.26)	-1.28(-1.84, -0.72)
Neck pain	511.29(266.78-882.06)	429.38(227.69-718.67)	0.02(-0.06, 0.09)	-0.29(-0.44, -0.15)	0.46(0.32, 0.61)	-0.07(-0.15, 0.00)
Neonatal disorders	12.69(8.34-18.57)	11.39(7.54-16.36)	-0.05(-0.19, 0.09)	-1.21(-1.55, -0.87)	0.26(0.06, 0.46)	0.84(0.76, 0.92)
Neuroblastoma and other peripheral nervous cell tumors	0.95(0.57-1.54)	4.44(2.95-5.43)	4.71(4.25, 5.18)	4.13(3.43, 4.83)	6.05(5.63, 6.47)	3.75(2.66, 4.86)
Non-Hodgkin lymphoma	216.50(184.89-271.01)	217.27(172.61-260.63)	0.08(-0.37, 0.53)	1.12(0.76, 1.49)	-0.63(-1.89, 0.65)	-0.36(-0.81, 0.10)
Non-melanoma skin cancer	108.04(88.72-136.45)	211.04(160.00-260.46)	0.95(0.52, 1.39)	1.80(1.08, 2.52)	1.74(1.06, 2.42)	-1.28(-1.79, -0.77)
Non-rheumatic valvular heart disease	72.08(53.26-101.13)	86.20(63.39-117.90)	-0.44(-0.67, -0.21)	0.46(0.12, 0.79)	-1.20(-1.70, -0.71)	-0.73(-0.95, -0.51)

Oral disorders	1, 246.25(788.82-1, 807.14)	1, 328.76(856.54-1, 879.39)	0.12(-0.10, 0.34)	-0.65(-0.92, -0.37)	0.56(0.27, 0.84)	0.65(0.21, 1.10)
Osteoarthritis	1, 150.63(556.23-2, 313.26)	1, 407.26(697.16-2, 816.54)	0.51(0.42, 0.60)	0.01(-0.10, 0.12)	0.62(0.47, 0.78)	0.88(0.76, 1.01)
Other cardiovascular and circulatory diseases	276.45(204.08-392.71)	281.04(212.29-374.37)	-0.76(-1.03, -0.49)	-0.22(-0.54, 0.11)	-1.40(-2.11, -0.70)	-0.78(-0.93, -0.64)
Other chronic respiratory diseases	31.94(23.84-56.35)	71.55(49.13-90.87)	1.29(1.04, 1.54)	3.25(2.75, 3.76)	3.33(2.87, 3.79)	-2.78(-3.08, -2.48)
Other digestive diseases	421.64(280.99-532.43)	238.50(194.64-294.39)	-3.42(-3.89, -2.95)	-3.67(-4.64, -2.70)	-5.06(-6.00, -4.10)	-1.31(-1.62, -1.00)
Other intestinal infectious diseases	1.06(0.03-2.96)	1.19(0.06-2.96)	-3.00(-3.62, -2.39)	-2.24(-2.89, -1.57)	-3.76(-5.35, -2.14)	-3.37(-3.64, -3.09)
Other malignant neoplasms	358.64(244.46-442.46)	297.96(232.60-366.03)	-0.83(-1.08, -0.57)	-0.60(-0.87, -0.33)	-0.69(-1.37, 0.00)	-1.22(-1.49, -0.94)
Other mental disorders	194.96(127.29-284.18)	202.63(134.47-296.39)	0.00(-0.01, 0.01)	0.01(-0.01, 0.03)	0.02(0.01, 0.03)	-0.04(-0.06, -0.02)
Other musculoskeletal disorders	528.44(265.23-929.75)	296.31(164.31-518.74)	-1.45(-2.02, -0.87)	-0.33(-0.85, 0.20)	1.53(-0.13, 3.22)	-5.52(-5.98, -5.05)
Other neglected tropical diseases	26.61(18.80-35.10)	15.56(11.53-21.12)	-1.97(-2.14, -1.80)	-0.88(-1.09, -0.66)	-2.59(-2.86, -2.33)	-2.49(-2.76, -2.21)
Other neoplasms	33.69(21.29-57.91)	60.05(37.82-105.12)	1.66(1.43, 1.89)	1.69(1.43, 1.95)	2.78(2.36, 3.21)	0.23(0.05, 0.40)
Other neurological disorders	28.14(19.23-37.81)	91.51(70.16-115.49)	3.34(3.00, 3.67)	3.92(3.64, 4.19)	2.76(2.50, 3.02)	3.37(2.33, 4.41)
Other nutritional deficiencies	34.58(27.81-42.45)	74.78(59.34-95.19)	0.55(-0.43, 1.54)	-0.19(-2.72, 2.39)	2.75(1.77, 3.73)	-2.23(-2.58, -1.87)
Other pharynx cancer	42.48(36.25-51.18)	32.81(27.14-38.37)	-0.93(-1.32, -0.54)	-0.44(-1.33, 0.46)	-2.20(-2.59, -1.81)	0.14(-0.27, 0.55)
Other sense organ diseases	201.29(99.96-387.49)	200.22(101.52-359.56)	0.06(0.04, 0.09)	0.07(0.01, 0.13)	0.06(0.05, 0.08)	0.04(0.03, 0.05)
Other skin and subcutaneous diseases	112.94(59.23-195.24)	154.74(83.74-263.48)	0.37(0.25, 0.48)	0.07(-0.24, 0.38)	0.56(0.48, 0.65)	0.42(0.37, 0.47)
Other transport injuries	62.06(50.59-74.05)	41.57(33.18-51.26)	-1.44(-1.77, -1.11)	-0.16(-0.75, 0.44)	-2.37(-2.60, -2.15)	-1.92(-2.68, -1.16)
Other unintentional injuries	117.81(89.38-154.88)	80.20(59.67-107.40)	-1.74(-1.96, -1.51)	-1.10(-1.42, -0.77)	-1.93(-2.20, -1.66)	-2.47(-2.89, -2.05)
Other unspecified infectious diseases	62.85(35.78-83.48)	38.44(24.19-53.37)	-2.30(-2.72, -1.89)	-1.76(-2.08, -1.44)	-4.49(-5.52, -3.45)	-0.35(-0.88, 0.18)
Otitis media	17.83(8.16-32.29)	13.80(6.84-25.81)	-0.89(-0.91, -0.87)	-0.86(-0.88, -0.84)	-0.98(-1.00, -0.96)	-0.82(-0.86, -0.77)
Ovarian cancer	97.38(76.15-125.96)	74.19(53.41-98.14)	-0.56(-1.26, 0.16)	1.83(0.56, 3.11)	-3.13(-3.92, -2.33)	0.31(-1.05, 1.68)
Pancreatic cancer	518.80(441.68-610.10)	745.77(600.37-897.77)	1.17(0.90, 1.44)	1.95(1.74, 2.17)	0.62(0.30, 0.93)	0.99(0.30, 1.69)

Pancreatitis	125.03(92.16-167.81)	124.16(93.01-163.53)	-0.74(-0.99, -0.49)	-0.79(-1.12, -0.46)	-0.41(-0.78, -0.03)	-1.39(-1.78, -0.99)
Paralytic ileus and intestinal obstruction	392.75(312.73-507.36)	318.75(241.93-413.45)	-2.33(-2.56, -2.10)	-2.02(-2.38, -1.67)	-2.56(-3.03, -2.10)	-2.87(-3.08, -2.65)
Parkinson's disease	1, 799.32(1, 541.48-2, 029.36)	2, 393.31(2, 008.92-2, 779.78)	0.08(-0.27, 0.43)	0.93(0.58, 1.28)	-0.64(-1.57, 0.30)	-0.32(-0.50, -0.14)
Pneumoconiosis	126.05(102.90-153.15)	97.08(77.85-121.34)	-0.98(-1.39, -0.57)	0.49(0.12, 0.86)	-1.74(-2.84, -0.63)	-2.17(-2.37, -1.98)
Poisonings	89.21(66.10-174.59)	107.87(56.78-136.80)	0.54(0.06, 1.04)	1.20(0.18, 2.23)	2.89(2.21, 3.58)	-3.69(-4.34, -3.04)
Police conflict and executions	1.45(0.29-2.32)	1.12(0.39-1.97)	0.20(-0.19, 0.59)	-1.32(-2.35, -0.28)	-2.25(-2.47, -2.03)	4.55(4.05, 5.06)
Prostate cancer	402.50(312.10-601.77)	716.57(500.31-966.04)	0.65(-0.01, 1.32)	3.63(2.87, 4.39)	-2.34(-3.24, -1.43)	0.20(-1.33, 1.75)
Protein-energy malnutrition	438.04(375.12-500.54)	336.90(283.67-396.91)	-3.81(-4.21, -3.41)	-5.66(-6.40, -4.91)	-4.22(-4.47, -3.97)	-1.32(-2.26, -0.38)
Pruritus	25.95(11.36-53.70)	29.89(14.38-58.29)	0.42(0.39, 0.45)	0.37(0.35, 0.39)	0.55(0.52, 0.58)	0.31(0.23, 0.40)
Psoriasis	56.56(41.46-73.68)	62.23(45.97-79.92)	0.87(0.84, 0.91)	1.36(1.27, 1.45)	0.72(0.67, 0.76)	0.55(0.52, 0.57)
Pulmonary Arterial Hypertension	108.39(74.33-164.02)	122.96(82.83-152.60)	-1.02(-1.65, -0.39)	-1.55(-2.22, -0.88)	2.19(0.42, 4.00)	-3.79(-4.23, -3.35)
Rabies	0.97(0.47-1.53)	0.61(0.30-0.99)	-1.22(-2.85, 0.43)	-2.02(-4.18, 0.18)	6.48(5.24, 7.72)	-9.81(-13.86, -5.56)
Rheumatic heart disease	2, 812.04(2, 339.02-3, 413.67)	1, 156.08(888.14-1, 467.99)	-4.14(-4.75, -3.53)	-3.68(-4.33, -3.02)	-4.87(-6.47, -3.25)	-4.22(-4.56, -3.88)
Rheumatoid arthritis	192.24(158.04-235.41)	244.58(191.01-295.27)	0.38(0.12, 0.63)	0.71(0.33, 1.09)	0.40(0.03, 0.78)	-0.33(-0.72, 0.07)
Road injuries	758.06(656.35-890.67)	769.39(659.81-897.46)	0.29(-0.08, 0.67)	2.41(1.92, 2.91)	0.10(-0.24, 0.45)	-2.21(-3.11, -1.30)
Scabies	111.13(58.53-182.47)	126.17(66.51-218.00)	-0.02(-0.04, 0.01)	0.03(-0.01, 0.08)	0.01(-0.04, 0.06)	-0.09(-0.12, -0.06)
Schistosomiasis	37.83(27.77-51.01)	7.69(5.44-11.03)	-5.33(-6.13, -4.53)	-5.21(-5.99, -4.42)	-6.64(-8.78, -4.44)	-4.42(-4.81, -4.02)
Schizophrenia	86.27(62.93-110.43)	66.30(48.55-86.18)	0.34(0.28, 0.40)	0.12(-0.03, 0.27)	0.40(0.37, 0.43)	0.55(0.45, 0.65)
Self-harm	1, 541.45(1, 013.60-1, 744.82)	793.04(652.85-964.71)	-2.58(-2.93, -2.23)	-0.21(-0.86, 0.46)	-4.59(-4.98, -4.20)	-2.87(-3.36, -2.37)
Sexually transmitted infections excluding HIV	12.72(8.24-19.18)	7.70(4.64-12.65)	-2.06(-2.56, -1.56)	-2.92(-4.14, -1.68)	-1.63(-1.92, -1.33)	-1.99(-2.16, -1.82)
Soft tissue and other extraosseous sarcomas	54.42(37.26-71.10)	45.56(31.86-64.75)	-1.17(-1.67, -0.67)	-0.06(-0.56, 0.45)	-2.19(-3.42, -0.96)	-1.63(-1.97, -1.29)

Stomach cancer	4, 771.79(4, 097.23-5, 885.04)	3, 090.92(2, 260.63-3, 670.66)	-1.27(-1.79, -0.73)	-0.18(-0.70, 0.34)	-1.71(-3.13, -0.26)	-2.44(-2.69, -2.18)
Stroke	47, 787.41(42, 188.29-53, 478.61)	43, 900.47(36, 823.16-50, 076.75)	-1.24(-1.76, -0.72)	0.55(0.00, 1.10)	-2.13(-3.48, -0.76)	-2.55(-2.85, -2.25)
Testicular cancer	8.16(6.54-9.74)	9.19(7.37-11.09)	0.18(-0.70, 1.07)	3.30(1.61, 5.02)	-4.12(-5.46, -2.77)	2.04(0.44, 3.66)
Tetanus	26.11(9.91-40.12)	1.62(0.45-4.00)	-9.71(-11.10, -8.30)	-9.97(-11.03, -8.90)	-9.93(-13.65, -6.04)	-9.95(-10.76, -9.13)
Thyroid cancer	70.47(59.99-88.29)	73.65(54.54-88.14)	-0.01(-0.46, 0.46)	0.89(0.33, 1.45)	-0.23(-0.89, 0.44)	-0.73(-1.77, 0.32)
Tracheal, bronchus,and lung cancer	3, 501.80(3, 030.65-3, 991.35)	5, 587.28(4, 602.70-6, 541.27)	1.44(1.00, 1.88)	2.61(2.20, 3.02)	1.00(-0.20, 2.21)	0.39(0.16, 0.62)
Trachoma	8.74(4.77-14.35)	1.09(0.50-2.00)	-6.24(-7.07, -5.41)	-3.36(-4.06, -2.65)	-9.81(-11.37, -8.22)	-5.48(-7.41, -3.50)
Tuberculosis	2, 370.63(1, 987.10-2, 817.68)	311.70(257.12-385.90)	-6.30(-6.64, -5.97)	-5.17(-5.39, -4.94)	-8.88(-9.61, -8.15)	-4.63(-5.16, -4.10)
Typhoid and paratyphoid	0.70(0.25-1.49)	0.23(0.11-0.43)	-2.70(-3.15, -2.25)	-3.33(-3.81, -2.85)	-2.46(-3.69, -1.22)	-2.48(-2.68, -2.28)
Upper digestive system diseases	1, 873.62(1, 608.01-2, 183.32)	1, 052.09(857.97-1, 252.82)	-2.74(-3.08, -2.40)	-2.18(-2.58, -1.79)	-3.11(-3.90, -2.31)	-3.65(-3.90, -3.40)
Upper respiratory infections	496.51(95.83-765.07)	68.33(42.38-137.38)	-8.66(-9.04, -8.27)	-4.14(-4.72, -3.56)	-16.13(-16.61, -15.66)	-4.40(-5.39, -3.41)
Urinary diseases and male infertility	523.51(374.10-678.07)	472.27(367.00-611.62)	-0.97(-1.29, -0.65)	0.29(-0.27, 0.85)	-2.62(-3.06, -2.18)	-0.64(-1.17, -0.11)
Urticaria	29.73(17.71-44.84)	24.76(14.96-38.21)	-0.10(-0.12, -0.08)	-0.13(-0.16, -0.09)	-0.06(-0.10, -0.02)	-0.13(-0.15, -0.12)
Uterine cancer	113.00(83.47-144.45)	57.28(39.31-79.62)	-1.79(-2.29, -1.28)	0.50(0.25, 0.74)	-2.52(-3.05, -1.99)	-2.71(-3.85, -1.55)
Varicella and herpes zoster	67.35(51.86-84.56)	24.09(17.06-33.91)	-5.18(-5.44, -4.92)	-4.79(-4.93, -4.64)	-7.59(-8.21, -6.96)	-2.82(-3.16, -2.48)
Vascular intestinal disorders	21.00(13.72-25.54)	19.76(15.17-24.12)	-1.99(-2.26, -1.72)	0.23(-0.22, 0.69)	-4.10(-4.49, -3.71)	-2.09(-2.44, -1.74)
Viral skin diseases	32.51(21.02-48.10)	27.36(17.76-41.70)	0.06(0.04, 0.07)	0.08(0.06, 0.10)	0.05(0.04, 0.07)	0.04(0.02, 0.07)
Vitamin A deficiency	1.52(0.86-2.45)	1.25(0.67-2.03)	-0.15(-0.27, -0.03)	0.18(0.11, 0.25)	-0.26(-0.34, -0.17)	-0.32(-0.65, 0.01)
**90-94 years**						
Acne vulgaris	1.82(1.17-2.87)	2.11(1.32-3.29)	0.47(0.45, 0.49)	0.48(0.43, 0.52)	0.54(0.48, 0.59)	0.39(0.38, 0.41)
Acute glomerulonephritis	151.05(108.45-206.72)	67.53(45.70-89.42)	-2.57(-2.96, -2.18)	-2.52(-3.18, -1.86)	-1.03(-1.48, -0.57)	-4.73(-5.46, -3.99)
Acute hepatitis	173.04(139.98-209.40)	16.00(12.48-19.86)	-7.53(-7.77, -7.29)	-6.92(-7.22, -6.61)	-9.40(-9.78, -9.01)	-5.68(-6.21, -5.14)

Adverse effects of medical treatment	122.28(75.91-162.74)	38.04(27.89-52.78)	-3.89(-4.32, -3.45)	-3.34(-3.61, -3.07)	-5.14(-6.37, -3.90)	-3.22(-3.51, -2.92)
Age-related and other hearing loss	4, 744.15(3, 497.31-6, 295.75)	4, 734.37(3, 484.81-6, 252.28)	0.01(-0.03, 0.04)	-0.43(-0.52, -0.34)	0.40(0.36, 0.45)	-0.01(-0.04, 0.01)
Alcohol use disorders	79.05(52.68-118.91)	95.28(64.07-141.59)	0.60(0.46, 0.75)	0.78(0.52, 1.03)	1.83(1.62, 2.05)	-1.20(-1.43, -0.98)
Alopecia areata	2.36(1.53-3.40)	2.31(1.52-3.32)	-0.07(-0.12, -0.02)	-0.06(-0.11, 0.00)	-0.03(-0.13, 0.07)	-0.16(-0.23, -0.10)
Alzheimer's disease and other dementias	28, 368.09(11, 292.91-65, 664.00)	30, 169.00(13, 031.89-65, 754.16)	0.18(0.06, 0.30)	0.31(0.13, 0.48)	-0.14(-0.18, -0.10)	0.47(0.12, 0.83)
Animal contact	36.89(29.11-45.37)	12.68(9.89-15.68)	-3.47(-3.81, -3.12)	-2.95(-3.19, -2.71)	-4.61(-5.58, -3.64)	-2.49(-2.85, -2.12)
Anxiety disorders	430.06(280.25-606.37)	436.40(287.38-611.34)	-0.01(-0.27, 0.24)	0.83(0.38, 1.28)	-0.65(-0.81, -0.48)	-0.24(-0.83, 0.36)
Aortic aneurysm	66.30(53.90-79.85)	72.19(55.53-91.54)	0.27(0.04, 0.51)	0.56(0.46, 0.65)	0.34(0.17, 0.51)	0.06(-0.47, 0.59)
Appendicitis	148.34(97.65-191.99)	37.56(27.23-49.18)	-4.43(-4.66, -4.19)	-2.93(-3.35, -2.50)	-5.98(-6.29, -5.66)	-4.37(-4.72, -4.01)
Asthma	2, 267.97(1, 692.40-3, 451.02)	763.93(584.23-934.00)	-3.56(-3.95, -3.17)	-2.75(-3.55, -1.94)	-4.28(-4.71, -3.85)	-3.74(-4.28, -3.20)
Atrial fibrillation and flutter	5, 629.66(4, 390.68-6, 903.21)	4, 986.34(3, 909.23-5, 995.86)	-0.39(-0.69, -0.09)	0.32(-0.21, 0.86)	-1.24(-1.55, -0.93)	-0.22(-0.77, 0.33)
Attention-deficit/hyperactivity disorder	0.00(0.00-0.00)	0.00(0.00-0.00)	-0.75(-0.86, -0.64)	-1.76(-1.87, -1.65)	-0.02(-0.17, 0.13)	-0.59(-0.88, -0.30)
Autism spectrum disorders	18.07(11.17-27.73)	31.21(20.02-44.87)	1.80(1.73, 1.86)	1.50(1.42, 1.59)	2.49(2.37, 2.60)	1.26(1.17, 1.35)
Bacterial skin diseases	709.50(515.94-896.13)	80.18(44.22-164.45)	-6.90(-7.58, -6.21)	-4.64(-5.03, -4.26)	-14.52(-15.85, -13.17)	-1.23(-2.88, 0.44)
Bipolar disorder	28.35(16.88-42.38)	27.79(16.37-41.17)	-0.05(-0.14, 0.04)	-0.11(-0.36, 0.15)	0.04(0.01, 0.06)	0.04(0.01, 0.06)
Bladder cancer	976.63(722.73-1, 116.27)	700.48(566.66-873.41)	-1.15(-1.42, -0.88)	-0.09(-0.59, 0.42)	-2.30(-2.72, -1.88)	-0.78(-1.03, -0.54)
Blindness and vision loss	3, 353.91(2, 476.71-4, 603.12)	2, 673.33(1, 936.42-3, 700.43)	-0.67(-0.86, -0.47)	0.03(-0.39, 0.46)	-1.50(-1.62, -1.39)	-0.58(-0.97, -0.20)
Brain and central nervous system cancer	180.63(132.71-222.70)	220.17(145.21-279.33)	0.62(0.46, 0.79)	0.97(0.70, 1.24)	1.46(1.36, 1.57)	-0.60(-0.94, -0.26)
Breast cancer	482.54(393.78-573.61)	452.15(345.41-547.10)	-0.20(-0.51, 0.12)	2.03(1.54, 2.53)	-3.18(-3.55, -2.80)	0.82(0.04, 1.61)
Cardiomyopathy and myocarditis	649.25(506.05-1, 049.18)	890.70(606.64-1, 131.16)	1.08(0.06, 2.10)	4.79(4.29, 5.30)	1.35(-1.63, 4.42)	-3.12(-3.75, -2.49)
Cervical cancer	211.60(170.34-259.05)	169.35(121.64-211.50)	-0.62(-1.08, -0.15)	0.23(-0.46, 0.92)	-2.25(-2.99, -1.51)	0.19(-0.62, 1.01)
Chronic kidney disease	5, 820.61(4, 948.66-6, 926.43)	4, 884.41(4, 084.35-5, 689.93)	-0.55(-0.95, -0.15)	-0.16(-0.30, -0.02)	-0.45(-1.65, 0.76)	-1.09(-1.30, -0.88)

Chronic obstructive pulmonary disease	106, 088.76(88, 283.37-119, 194.03)	40, 391.12(32, 540.43-47, 328.24)	-3.17(-3.43, -2.91)	-1.84(-2.09, -1.59)	-4.42(-4.63, -4.21)	-3.17(-3.94, -2.40)
Cirrhosis and other chronic liver diseases	1, 566.54(1, 265.02-1, 870.69)	781.44(596.34-962.80)	-2.32(-2.65, -1.98)	-1.77(-2.20, -1.34)	-2.70(-3.21, -2.19)	-2.70(-3.24, -2.15)
Colon and rectum cancer	2, 789.69(2, 336.57-3, 174.90)	2, 358.39(1, 853.90-2, 793.68)	-0.59(-0.80, -0.38)	0.04(-0.34, 0.43)	-1.87(-2.20, -1.54)	0.22(-0.15, 0.60)
Conflict and terrorism	0.02(0.01-0.04)	0.08(0.05-0.12)	3.72(2.30, 5.15)	8.95(5.33, 12.69)	2.07(0.88, 3.29)	-1.65(-3.42, 0.16)
Congenital birth defects	17.40(10.93-26.11)	19.24(12.34-28.51)	0.31(0.08, 0.54)	1.07(0.59, 1.56)	0.30(-0.08, 0.68)	-0.67(-0.76, -0.58)
Cystic echinococcosis	4.28(2.17-6.65)	1.22(0.72-1.81)	-3.97(-4.26, -3.69)	-3.03(-3.48, -2.58)	-4.53(-4.90, -4.16)	-4.44(-4.88, -4.01)
Cysticercosis	92.94(45.74-168.95)	46.75(19.48-95.34)	-2.15(-2.41, -1.88)	-2.02(-2.13, -1.90)	-3.93(-4.36, -3.50)	-0.17(-0.89, 0.56)
Decubitus ulcer	98.89(55.19-237.68)	219.26(140.20-282.43)	3.57(2.40, 4.75)	12.38(10.81, 13.98)	1.46(-0.66, 3.63)	-2.90(-4.84, -0.92)
Dengue	0.23(0.12-0.36)	0.11(0.06-0.21)	-2.33(-3.42, -1.22)	-5.72(-8.39, -2.96)	-0.63(-1.24, -0.02)	-1.37(-1.70, -1.04)
Depressive disorders	916.11(628.91-1, 295.56)	933.96(653.92-1, 315.37)	0.07(0.01, 0.13)	0.37(0.35, 0.39)	0.22(0.18, 0.27)	-0.41(-0.60, -0.23)
Dermatitis	81.78(45.03-134.99)	80.89(44.75-132.44)	-0.04(-0.05, -0.02)	-0.05(-0.06, -0.03)	0.01(-0.01, 0.03)	-0.10(-0.13, -0.07)
Diabetes mellitus	2, 676.11(2, 294.34-3, 072.49)	3, 049.21(2, 558.24-3, 585.04)	0.47(0.11, 0.83)	1.95(1.69, 2.22)	-0.66(-1.65, 0.33)	0.13(-0.04, 0.30)
Diarrheal diseases	1, 020.04(432.35-1, 648.56)	92.88(54.67-172.59)	-7.58(-7.74, -7.41)	-8.43(-8.53, -8.33)	-8.43(-8.53, -8.33)	-5.47(-5.99, -4.96)
Dietary iron deficiency	534.24(337.18-789.44)	282.61(157.98-466.18)	-2.06(-2.18, -1.93)	-1.08(-1.41, -0.74)	-3.11(-3.24, -2.98)	-1.72(-1.80, -1.65)
Drowning	400.17(342.75-467.18)	297.91(242.95-345.98)	-1.08(-1.68, -0.48)	-0.66(-1.57, 0.26)	-0.02(-1.48, 1.47)	-2.87(-3.07, -2.67)
Drug use disorders	132.20(110.93-154.04)	43.56(35.57-53.48)	-3.58(-3.84, -3.32)	-1.38(-1.69, -1.08)	-6.99(-7.27, -6.72)	-1.33(-1.97, -0.69)
Encephalitis	23.17(18.76-29.00)	15.75(12.12-19.89)	-1.27(-1.70, -0.83)	-1.11(-1.75, -0.47)	-0.31(-0.69, 0.06)	-2.38(-3.44, -1.32)
Endocarditis	113.02(51.32-158.54)	37.77(26.33-49.19)	-3.44(-3.74, -3.14)	-0.05(-0.56, 0.47)	-7.66(-8.20, -7.12)	-1.48(-1.87, -1.10)
Endocrine, metabolic, blood, and immune disorders	617.59(471.63-799.48)	564.40(380.78-713.58)	-0.26(-0.41, -0.12)	-0.38(-0.55, -0.21)	-0.32(-0.53, -0.10)	-0.07(-0.35, 0.21)
Environmental heat and cold exposure	299.69(109.17-408.32)	111.26(46.89-151.03)	-3.18(-3.52, -2.84)	-3.15(-3.38, -2.92)	-4.12(-5.05, -3.18)	-2.27(-2.54, -2.00)
Esophageal cancer	2, 507.43(1, 973.35-2, 849.70)	1, 823.55(1, 414.86-2, 157.37)	-0.98(-1.27, -0.69)	0.84(0.65, 1.02)	-1.75(-2.37, -1.12)	-1.94(-2.46, -1.42)

Exposure to forces of nature	14.74(12.81-17.25)	8.14(6.06-10.91)	-6.45(-15.66, 3.77)	-6.58(-11.15, -1.78)	20.38(-9.80, 60.66)	-27.19(-34.30, -19.31)
Exposure to mechanical forces	128.51(100.68-183.06)	141.71(100.99-180.59)	0.51(0.13, 0.88)	0.45(0.10, 0.81)	2.93(1.92, 3.95)	-2.11(-2.56, -1.65)
Eye cancer	11.10(6.06-14.62)	10.19(4.34-14.60)	-0.28(-1.13, 0.58)	-0.44(-1.07, 0.19)	-0.56(-2.96, 1.90)	0.05(-0.61, 0.72)
Falls	5, 913.15(5, 029.36-7, 130.68)	7, 015.33(4, 943.52-8, 660.70)	0.62(0.44, 0.79)	0.66(0.43, 0.89)	-0.43(-0.81, -0.04)	1.25(0.99, 1.51)
Fire, heat, and hot substances	472.67(339.45-548.67)	231.52(172.70-276.94)	-2.48(-2.87, -2.09)	-3.16(-3.46, -2.86)	-0.87(-2.01, 0.29)	-3.33(-3.50, -3.16)
Food-borne trematodiases	96.68(43.85-183.51)	54.52(28.94-95.83)	-1.87(-2.52, -1.21)	-2.84(-4.02, -1.65)	-1.91(-2.16, -1.65)	-1.21(-2.63, 0.24)
Foreign body	185.10(137.98-220.98)	218.43(138.07-266.82)	0.44(0.19, 0.68)	1.04(0.51, 1.58)	0.68(0.43, 0.93)	-0.63(-0.86, -0.40)
Fungal skin diseases	109.39(43.10-235.34)	107.44(42.17-230.92)	-0.06(-0.06, -0.05)	-0.05(-0.07, -0.04)	-0.02(-0.02, -0.01)	-0.11(-0.12, -0.10)
Gallbladder and biliary diseases	1, 968.90(1, 205.11-2, 524.53)	1, 177.47(921.22-1, 569.75)	-1.71(-1.90, -1.52)	-1.62(-1.97, -1.28)	-2.29(-2.51, -2.07)	-1.14(-1.45, -0.82)
Gallbladder and biliary tract cancer	355.77(244.75-436.38)	306.27(195.51-395.60)	-0.47(-0.80, -0.13)	0.16(-0.10, 0.42)	-1.31(-2.17, -0.44)	-0.31(-0.73, 0.12)
Gout	81.11(47.92-131.07)	105.54(63.30-170.91)	0.89(0.78, 1.01)	-0.52(-0.70, -0.33)	1.91(1.67, 2.16)	1.14(1.03, 1.26)
Gynecological diseases	165.09(103.66-260.93)	147.31(94.28-229.40)	-0.36(-0.41, -0.31)	-0.23(-0.29, -0.17)	-0.80(-0.91, -0.69)	-0.21(-0.27, -0.16)
Headache disorders	203.66(51.15-458.65)	213.22(47.34-480.12)	0.14(0.11, 0.17)	-0.10(-0.14, -0.07)	0.55(0.49, 0.60)	-0.17(-0.19, -0.16)
Hemoglobinopathies and hemolytic anemias	415.90(301.46-557.02)	260.66(183.15-359.30)	-1.51(-1.61, -1.41)	-0.70(-0.83, -0.57)	-2.21(-2.26, -2.16)	-1.47(-1.76, -1.18)
HIV/AIDS	2.93(0.65-13.29)	27.05(13.65-47.89)	7.33(2.04, 12.89)	14.63(-1.23, 33.04)	-1.13(-2.78, 0.55)	9.57(5.28, 14.05)
Hodgkin lymphoma	23.27(9.26-32.09)	8.82(4.82-11.59)	-3.12(-3.42, -2.82)	-2.05(-2.69, -1.41)	-5.25(-5.75, -4.75)	-1.76(-2.20, -1.33)
Hypertensive heart disease	19, 855.94(15, 450.18-22, 737.43)	10, 674.97(7, 112.20-13, 842.63)	-1.92(-2.16, -1.67)	-2.74(-3.02, -2.47)	-1.44(-1.97, -0.91)	-1.28(-1.69, -0.86)
Idiopathic developmental intellectual disability	4.55(2.11-8.39)	3.51(1.36-6.57)	-0.85(-1.03, -0.67)	1.27(0.86, 1.67)	-1.99(-2.12, -1.87)	-1.81(-2.14, -1.48)
Idiopathic epilepsy	169.68(109.00-247.82)	140.20(86.94-211.66)	-0.51(-0.63, -0.40)	-0.22(-0.31, -0.13)	-1.78(-1.83, -1.73)	0.71(0.34, 1.08)
Inflammatory bowel disease	356.67(197.81-471.54)	168.56(125.40-253.29)	-2.45(-2.71, -2.20)	-0.22(-0.67, 0.24)	-4.50(-4.79, -4.20)	-2.59(-3.00, -2.17)
Inguinal, femoral, and abdominal hernia	89.72(69.18-126.71)	48.49(37.38-60.20)	-2.05(-2.33, -1.78)	-0.85(-1.46, -0.25)	-2.67(-3.03, -2.31)	-2.75(-3.13, -2.37)

Interpersonal violence	105.64(81.04-134.96)	54.00(41.43-70.16)	-2.14(-2.26, -2.02)	-1.79(-1.92, -1.66)	-3.67(-3.86, -3.47)	-0.84(-1.14, -0.55)
Interstitial lung disease and pulmonary sarcoidosis	93.94(71.84-150.22)	110.34(66.42-144.35)	0.52(0.27, 0.78)	-0.47(-0.88, -0.06)	3.25(2.79, 3.70)	-1.57(-2.04, -1.10)
Intestinal nematode infections	48.83(26.49-75.80)	1.39(0.78-2.18)	-10.99(-11.33, -10.66)	-11.77(-12.43, -11.12)	-13.76(-14.19, -13.33)	-5.63(-6.12, -5.13)
Invasive Non-typhoidal Salmonella (iNTS)	2.14(0.44-5.81)	1.31(0.21-3.85)	-1.57(-1.80, -1.33)	-1.11(-1.45, -0.78)	-1.92(-2.25, -1.60)	-1.76(-2.16, -1.36)
Iodine deficiency	11.12(6.09-18.46)	11.35(5.28-20.68)	0.07(-0.04, 0.18)	-1.05(-1.16, -0.94)	1.78(1.56, 1.99)	-1.10(-1.30, -0.91)
Ischemic heart disease	42, 122.97(36, 976.36-47, 426.67)	58, 392.90(47, 510.31-67, 630.25)	1.07(0.68, 1.46)	2.17(1.60, 2.74)	2.34(1.62, 3.07)	-1.75(-2.31, -1.18)
Kidney cancer	189.84(159.10-215.24)	178.25(144.72-212.73)	-0.19(-0.63, 0.25)	0.30(-0.30, 0.90)	-1.70(-2.77, -0.62)	0.79(0.41, 1.18)
Larynx cancer	138.89(117.44-162.53)	102.08(79.88-121.83)	-0.98(-1.26, -0.70)	-0.49(-1.28, 0.32)	-1.21(-1.37, -1.06)	-1.21(-1.37, -1.06)
Leishmaniasis	0.90(0.19-2.48)	0.12(0.03-0.34)	-6.39(-6.79, -5.99)	-8.46(-9.57, -7.34)	-5.39(-5.58, -5.20)	-5.39(-5.58, -5.20)
Leprosy	0.27(0.16-0.41)	0.08(0.05-0.12)	-3.89(-3.98, -3.81)	-3.20(-3.30, -3.11)	-5.32(-5.42, -5.21)	-2.87(-3.09, -2.64)
Leukemia	243.02(188.02-298.20)	182.75(141.51-244.30)	-0.94(-1.05, -0.84)	0.91(0.66, 1.16)	-1.95(-2.05, -1.84)	-1.95(-2.05, -1.84)
Lip and oral cavity cancer	149.19(127.17-170.49)	158.58(128.16-185.24)	0.19(-0.26, 0.64)	0.80(0.03, 1.57)	0.26(-0.16, 0.67)	-0.50(-1.47, 0.48)
Liver cancer	829.86(670.02-991.16)	822.88(645.36-1, 014.22)	-0.04(-1.44, 1.38)	0.26(-0.78, 1.32)	0.03(-4.03, 4.25)	-0.43(-1.26, 0.41)
Low back pain	1, 909.56(1, 223.05-2, 687.78)	1, 713.25(1, 112.95-2, 401.64)	-0.36(-0.38, -0.33)	-0.34(-0.41, -0.28)	-0.16(-0.21, -0.11)	-0.57(-0.59, -0.55)
Lower extremity peripheral arterial disease	168.60(96.45-273.81)	172.62(107.62-267.57)	0.05(-0.06, 0.15)	0.77(0.70, 0.84)	-0.18(-0.37, 0.01)	-0.43(-0.65, -0.21)
Lower respiratory infections	21, 198.23(17, 485.94-24, 178.75)	8, 991.46(7, 025.11-11, 265.64)	-3.06(-3.27, -2.85)	-1.17(-1.70, -0.63)	-3.94(-4.15, -3.73)	-3.94(-4.15, -3.73)
Malignant neoplasm of bone and articular cartilage	48.07(30.32-85.19)	82.87(50.43-109.37)	1.96(1.49, 2.43)	4.56(3.87, 5.26)	3.03(1.86, 4.20)	-2.37(-2.65, -2.08)
Malignant skin melanoma	42.68(30.87-63.42)	46.85(23.08-62.11)	0.42(0.01, 0.82)	1.34(0.36, 2.33)	-0.37(-0.72, -0.02)	0.38(-0.11, 0.87)
Meningitis	170.89(147.24-196.98)	60.58(47.90-73.39)	-3.30(-3.95, -2.64)	-3.51(-4.33, -2.69)	-3.07(-4.66, -1.47)	-2.85(-3.56, -2.14)
Mesothelioma	12.81(10.01-14.97)	14.90(11.27-17.81)	0.47(0.08, 0.87)	-0.08(-0.32, 0.17)	3.83(3.29, 4.38)	-2.06(-3.15, -0.96)

Motor neuron disease	2.87(2.17-3.72)	4.69(3.47-5.99)	1.57(1.36, 1.79)	2.85(2.57, 3.12)	-0.46(-0.82, -0.10)	2.91(2.49, 3.33)
Multiple myeloma	14.81(8.98-32.59)	49.12(30.09-65.36)	4.39(3.79, 4.99)	12.30(10.69, 13.95)	0.02(-0.59, 0.62)	1.76(1.17, 2.35)
Multiple sclerosis	1.46(0.83-2.10)	1.73(1.39-2.12)	0.59(0.20, 0.98)	2.87(2.61, 3.12)	-0.39(-1.40, 0.64)	-0.49(-0.93, -0.06)
Nasopharynx cancer	244.61(204.49-291.13)	87.67(70.74-103.55)	-3.36(-3.70, -3.01)	-1.55(-2.28, -0.82)	-5.97(-6.40, -5.54)	-2.13(-2.62, -1.63)
Neck pain	344.80(184.72-586.99)	346.46(186.58-577.71)	0.02(0.00, 0.03)	-0.05(-0.06, -0.04)	0.19(0.16, 0.22)	-0.10(-0.12, -0.07)
Neonatal disorders	11.15(7.38-16.37)	10.24(6.96-14.61)	-0.28(-0.40, -0.15)	-1.38(-1.71, -1.05)	-0.04(-0.17, 0.09)	0.65(0.59, 0.71)
Neuroblastoma and other peripheral nervous cell tumors	0.98(0.58-1.53)	4.11(2.79-5.14)	4.63(4.22, 5.05)	3.17(2.58, 3.76)	7.31(6.93, 7.69)	3.11(2.10, 4.12)
Non-Hodgkin lymphoma	199.84(168.30-239.76)	192.78(153.66-233.98)	-0.16(-0.43, 0.12)	0.89(0.59, 1.19)	-0.86(-1.52, -0.19)	-0.20(-0.52, 0.12)
Non-melanoma skin cancer	208.74(167.50-286.99)	298.39(217.35-363.03)	1.14(0.79, 1.49)	2.03(1.51, 2.55)	2.35(1.65, 3.06)	-1.43(-1.80, -1.07)
Non-rheumatic valvular heart disease	149.87(112.81-194.17)	114.80(87.84-151.10)	-0.83(-0.99, -0.66)	0.21(-0.05, 0.47)	-2.19(-2.50, -1.88)	-0.63(-0.85, -0.42)
Oral disorders	1, 349.19(850.18-1, 906.03)	1, 387.30(901.45-1, 941.21)	0.17(-0.02, 0.35)	-0.34(-0.57, -0.11)	0.68(0.45, 0.92)	0.31(-0.06, 0.67)
Osteoarthritis	1, 282.84(647.24-2, 567.90)	1, 470.71(747.45-2, 945.93)	0.48(0.41, 0.54)	0.01(-0.09, 0.10)	0.82(0.73, 0.92)	0.56(0.45, 0.67)
Other cardiovascular and circulatory diseases	419.75(312.56-582.60)	303.27(231.92-401.26)	-1.07(-1.26, -0.89)	-0.51(-0.75, -0.27)	-1.69(-1.87, -1.51)	-0.97(-1.44, -0.50)
Other chronic respiratory diseases	80.06(56.75-152.38)	119.91(78.76-155.53)	1.43(0.94, 1.93)	3.79(2.60, 4.99)	4.78(4.28, 5.28)	-4.03(-4.43, -3.64)
Other digestive diseases	1, 017.25(661.32-1, 321.56)	371.25(293.86-489.46)	-3.28(-3.54, -3.01)	-3.58(-4.08, -3.08)	-4.58(-4.88, -4.28)	-1.21(-1.68, -0.72)
Other intestinal infectious diseases	2.82(0.09-8.57)	1.18(0.05-3.23)	-2.80(-3.14, -2.46)	-2.02(-2.64, -1.40)	-3.49(-3.90, -3.09)	-2.92(-3.43, -2.41)
Other malignant neoplasms	411.82(271.68-528.27)	292.11(225.81-358.62)	-1.15(-1.37, -0.93)	-1.05(-1.41, -0.69)	-0.67(-1.15, -0.19)	-1.55(-1.79, -1.32)
Other mental disorders	218.33(145.12-316.79)	215.33(144.26-312.65)	-0.04(-0.05, -0.03)	-0.07(-0.09, -0.04)	0.02(0.01, 0.04)	-0.09(-0.11, -0.07)
Other musculoskeletal disorders	327.77(169.85-571.74)	222.44(132.91-375.10)	-1.64(-2.45, -0.82)	-0.18(-0.94, 0.60)	2.46(0.08, 4.89)	-7.08(-7.69, -6.48)
Other neglected tropical diseases	24.11(16.55-33.78)	14.03(9.67-20.94)	-1.84(-2.10, -1.57)	-1.37(-1.66, -1.08)	-2.04(-2.56, -1.52)	-2.24(-2.63, -1.85)
Other neoplasms	35.08(20.24-52.42)	53.67(35.06-82.42)	1.39(1.24, 1.55)	1.35(1.05, 1.66)	2.51(2.30, 2.71)	0.20(-0.03, 0.43)

Other neurological disorders	34.66(24.74-46.03)	114.20(90.86-140.07)	4.06(3.74, 4.39)	5.51(5.24, 5.78)	3.02(2.68, 3.35)	3.71(2.76, 4.68)
Other nutritional deficiencies	142.21(110.24-182.25)	173.29(128.46-224.07)	0.82(0.14, 1.51)	-0.19(-1.11, 0.74)	4.13(2.72, 5.56)	-2.76(-3.37, -2.14)
Other pharynx cancer	38.65(33.16-45.50)	27.77(22.45-33.04)	-1.01(-1.40, -0.61)	-0.92(-1.79, -0.04)	-1.61(-1.97, -1.26)	-0.06(-0.36, 0.24)
Other sense organ diseases	154.30(79.88-273.34)	156.86(81.11-281.34)	0.05(0.04, 0.06)	0.07(0.05, 0.09)	0.11(0.10, 0.13)	-0.03(-0.05, -0.01)
Other skin and subcutaneous diseases	152.58(87.04-247.85)	155.43(83.59-268.70)	0.09(-0.01, 0.19)	-0.41(-0.52, -0.29)	0.43(0.33, 0.53)	0.27(0.04, 0.49)
Other transport injuries	73.93(60.20-89.25)	49.70(39.24-62.13)	-1.25(-1.64, -0.85)	-0.23(-0.92, 0.46)	-1.65(-1.87, -1.42)	-1.98(-2.97, -0.98)
Other unintentional injuries	154.38(117.52-208.13)	89.20(69.07-116.67)	-1.67(-1.97, -1.37)	-1.05(-1.40, -0.70)	-1.43(-1.78, -1.07)	-2.81(-3.52, -2.08)
Other unspecified infectious diseases	104.91(50.99-144.11)	48.05(29.19-68.34)	-2.58(-2.97, -2.19)	-1.75(-2.24, -1.26)	-4.93(-5.26, -4.60)	-0.30(-1.48, 0.89)
Otitis media	19.42(10.27-34.96)	15.13(7.69-28.70)	-0.81(-0.88, -0.75)	-0.82(-0.89, -0.74)	-0.85(-0.91, -0.78)	-0.79(-0.86, -0.73)
Ovarian cancer	87.88(67.26-111.08)	77.43(53.25-99.17)	-0.33(-1.03, 0.38)	2.36(0.99, 3.76)	-3.77(-4.60, -2.93)	1.20(0.01, 2.41)
Pancreatic cancer	534.45(453.27-633.66)	665.15(518.81-806.37)	0.73(0.53, 0.94)	1.33(1.02, 1.64)	-0.36(-0.63, -0.08)	1.35(0.85, 1.85)
Pancreatitis	192.44(137.11-266.48)	142.78(103.48-189.69)	-0.96(-1.22, -0.69)	-0.98(-1.34, -0.63)	-0.79(-1.21, -0.38)	-1.40(-1.78, -1.03)
Paralytic ileus and intestinal obstruction	976.42(745.58-1, 249.90)	434.98(324.09-567.06)	-2.61(-2.89, -2.32)	-2.63(-3.09, -2.16)	-2.92(-3.42, -2.42)	-2.66(-3.03, -2.30)
Parkinson's disease	2, 441.22(2, 002.65-2, 771.34)	2, 405.17(1, 981.62-2, 832.46)	-0.09(-0.22, 0.04)	0.80(0.55, 1.04)	-0.43(-0.55, -0.30)	-0.73(-0.88, -0.58)
Pneumoconiosis	92.84(75.46-113.26)	67.81(53.70-83.69)	-1.02(-1.23, -0.81)	-0.39(-0.53, -0.25)	-0.39(-0.53, -0.25)	-2.55(-3.17, -1.92)
Poisonings	80.77(57.88-160.58)	91.64(53.52-116.55)	0.53(0.05, 1.02)	1.36(0.44, 2.30)	3.03(2.19, 3.89)	-3.68(-4.19, -3.17)
Police conflict and executions	4.47(1.03-7.17)	1.55(0.84-2.31)	-3.53(-4.11, -2.95)	-3.18(-4.42, -1.92)	-5.87(-7.00, -4.72)	-1.39(-1.74, -1.04)
Prostate cancer	741.90(478.20-949.94)	692.65(477.04-942.78)	-0.32(-1.07, 0.45)	1.93(0.93, 2.94)	-1.63(-2.70, -0.55)	-0.86(-2.32, 0.61)
Protein-energy malnutrition	3, 004.04(2, 544.90-3, 472.35)	868.85(696.33-1, 045.35)	-4.00(-4.25, -3.76)	-6.03(-6.43, -5.63)	-3.58(-3.76, -3.41)	-2.10(-2.69, -1.50)
Pruritus	25.62(11.58-50.39)	29.17(13.02-57.65)	0.43(0.41, 0.45)	0.33(0.28, 0.38)	0.62(0.59, 0.65)	0.34(0.32, 0.37)
Psoriasis	38.25(28.91-47.87)	50.47(37.66-63.19)	0.90(0.82, 0.97)	1.46(1.28, 1.64)	0.75(0.64, 0.86)	0.47(0.43, 0.51)
Pulmonary Arterial Hypertension	201.89(137.18-299.51)	166.30(108.19-209.43)	-0.58(-1.00, -0.16)	-1.53(-1.86, -1.19)	3.95(3.30, 4.62)	-3.69(-4.73, -2.64)

Rabies	1.25(0.52-2.19)	0.98(0.44-1.69)	-1.06(-2.51, 0.41)	-1.74(-3.99, 0.56)	8.55(7.26, 9.85)	-11.02(-14.26, -7.66)
Rheumatic heart disease	5, 043.30(4, 131.05-6, 320.47)	1, 522.11(1, 108.36-1, 958.82)	-3.87(-4.27, -3.47)	-3.35(-3.94, -2.76)	-4.30(-4.79, -3.81)	-4.05(-4.84, -3.26)
Rheumatoid arthritis	241.87(202.10-296.15)	261.11(196.88-315.90)	0.27(0.04, 0.49)	0.46(0.09, 0.82)	0.53(0.17, 0.89)	-0.58(-0.96, -0.20)
Road injuries	643.67(549.63-763.10)	683.18(580.14-796.66)	0.19(-0.23, 0.60)	1.85(1.20, 2.51)	0.64(-0.42, 1.71)	-2.25(-2.38, -2.11)
Scabies	134.72(71.07-228.43)	132.73(70.57-226.02)	-0.04(-0.06, -0.01)	0.02(-0.01, 0.05)	-0.02(-0.09, 0.05)	-0.09(-0.11, -0.07)
Schistosomiasis	36.62(22.95-53.15)	8.07(5.48-11.82)	-4.94(-5.43, -4.45)	-5.61(-6.37, -4.83)	-5.97(-6.68, -5.26)	-3.13(-3.81, -2.44)
Schizophrenia	39.76(28.39-51.09)	47.40(33.99-60.90)	0.57(0.49, 0.65)	0.40(0.18, 0.63)	0.64(0.62, 0.67)	0.72(0.70, 0.74)
Self-harm	1, 498.41(988.00-1, 698.48)	665.05(542.49-791.30)	-2.56(-2.97, -2.15)	-0.59(-0.83, -0.35)	-4.24(-4.44, -4.04)	-2.70(-4.03, -1.37)
Sexually transmitted infections excluding HIV	16.67(10.98-25.47)	8.44(5.48-13.01)	-2.29(-2.68, -1.91)	-3.61(-4.60, -2.61)	-1.47(-1.75, -1.19)	-2.13(-2.28, -1.99)
Soft tissue and other extraosseous sarcomas	62.78(41.08-80.92)	43.83(30.67-61.48)	-1.19(-1.54, -0.83)	-0.24(-0.49, 0.00)	-1.77(-2.76, -0.77)	-1.47(-1.85, -1.09)
Stomach cancer	4, 627.71(3, 794.53-5, 723.17)	2, 952.63(2, 275.52-3, 535.89)	-1.47(-1.78, -1.17)	-0.19(-0.71, 0.33)	-1.95(-2.27, -1.63)	-2.46(-3.06, -1.87)
Stroke	72, 767.12(61, 445.25-81, 953.64)	50, 762.16(41, 197.19-58, 738.91)	-1.13(-1.55, -0.71)	0.68(0.01, 1.36)	-1.86(-2.16, -1.56)	-2.36(-3.38, -1.33)
Testicular cancer	9.01(7.43-10.51)	7.37(5.76-9.26)	-0.74(-1.78, 0.31)	1.54(0.21, 2.88)	-3.11(-3.78, -2.43)	-0.75(-3.48, 2.06)
Tetanus	31.89(9.97-47.77)	1.47(0.39-3.18)	-9.71(-10.78, -8.62)	-9.31(-11.11, -7.46)	-9.43(-11.27, -7.55)	-11.07(-11.45, -10.68)
Thyroid cancer	72.35(58.46-87.48)	69.10(50.86-83.23)	-0.14(-0.53, 0.24)	0.87(0.32, 1.42)	-0.40(-0.70, -0.09)	-0.99(-1.88, -0.10)
Tracheal, bronchus, and lung cancer	3, 952.46(3, 412.86-4, 518.03)	4, 889.59(3, 902.01-5, 798.01)	0.67(0.53, 0.81)	2.14(1.78, 2.51)	-0.03(-0.16, 0.11)	-0.03(-0.16, 0.11)
Trachoma	9.15(4.65-16.44)	1.20(0.52-2.34)	-6.26(-6.88, -5.64)	-4.51(-6.01, -2.99)	-9.09(-9.41, -8.77)	-4.32(-5.57, -3.05)
Tuberculosis	2, 098.36(1, 751.37-2, 453.35)	217.62(177.69-265.10)	-7.07(-7.42, -6.71)	-5.20(-5.43, -4.97)	-10.49(-11.27, -9.70)	-4.99(-5.59, -4.39)
Typhoid and paratyphoid	0.37(0.15-0.79)	0.16(0.08-0.31)	-2.59(-2.95, -2.24)	-4.72(-5.21, -4.22)	-0.71(-1.02, -0.40)	-2.30(-3.04, -1.56)
Upper digestive system diseases	3, 132.83(2, 627.84-3, 672.21)	1, 350.24(1, 068.45-1, 622.66)	-2.72(-3.03, -2.40)	-2.31(-2.72, -1.90)	-2.76(-3.30, -2.21)	-3.54(-4.04, -3.05)
Upper respiratory infections	1, 780.86(323.71-2, 751.00)	114.08(68.98-277.65)	-8.76(-9.22, -8.30)	-4.68(-5.25, -4.12)	-14.58(-15.17, -13.99)	-5.56(-6.43, -4.68)

Urinary diseases and male infertility	793.53(583.50-1, 023.96)	510.95(410.55-664.10)	-1.44(-1.75, -1.12)	-0.17(-0.76, 0.43)	-2.69(-2.92, -2.47)	-1.63(-2.21, -1.05)
Urticaria	22.39(13.12-34.72)	21.97(12.69-33.83)	-0.06(-0.09, -0.04)	-0.02(-0.05, 0.01)	-0.06(-0.12, -0.01)	-0.12(-0.16, -0.08)
Uterine cancer	90.06(66.64-121.47)	58.94(40.64-80.44)	-1.33(-1.84, -0.81)	1.49(1.24, 1.75)	-3.05(-3.55, -2.53)	-1.93(-3.10, -0.75)
Varicella and herpes zoster	180.34(124.42-244.62)	29.62(20.32-41.78)	-5.79(-6.06, -5.53)	-6.13(-6.45, -5.81)	-7.05(-7.49, -6.62)	-3.96(-4.35, -3.56)
Vascular intestinal disorders	54.27(30.88-71.02)	27.07(17.83-34.77)	-2.22(-2.43, -2.01)	-0.15(-0.49, 0.19)	-4.68(-4.98, -4.37)	-1.69(-1.96, -1.42)
Viral skin diseases	22.42(14.28-33.57)	22.38(14.07-33.51)	-0.01(-0.03, 0.01)	-0.08(-0.10, -0.05)	0.07(0.03, 0.10)	-0.03(-0.06, -0.01)
Vitamin A deficiency	1.12(0.59-1.90)	1.02(0.52-1.77)	-0.30(-0.50, -0.09)	-0.53(-0.80, -0.25)	-0.43(-0.64, -0.21)	0.20(-0.29, 0.68)
**95+ years**						
Acne vulgaris	0.93(0.59-1.46)	1.07(0.67-1.66)	0.46(0.45, 0.47)	0.52(0.51, 0.53)	0.51(0.50, 0.52)	0.36(0.33, 0.38)
Acute glomerulonephritis	146.10(100.21-198.36)	70.80(47.49-92.62)	-2.11(-2.53, -1.68)	-3.42(-4.29, -2.55)	0.30(-0.24, 0.84)	-3.49(-4.08, -2.89)
Acute hepatitis	126.98(100.26-155.81)	13.63(10.11-17.19)	-7.06(-7.41, -6.71)	-7.19(-7.54, -6.84)	-9.03(-9.75, -8.30)	-5.03(-5.74, -4.31)
Adverse effects of medical treatment	152.63(88.91-204.15)	52.21(36.41-72.62)	-3.41(-3.92, -2.90)	-3.90(-4.15, -3.65)	-4.97(-6.35, -3.58)	-0.95(-1.51, -0.38)
Age-related and other hearing loss	4, 671.68(3, 408.71-6, 185.29)	4, 566.63(3, 314.77-5, 985.06)	-0.06(-0.09, -0.03)	-0.46(-0.54, -0.39)	0.39(0.35, 0.42)	-0.17(-0.19, -0.14)
Alcohol use disorders	83.58(54.06-124.72)	99.55(65.08-150.49)	0.57(0.48, 0.67)	0.25(0.09, 0.42)	1.43(1.32, 1.54)	-0.20(-0.39, -0.02)
Alopecia areata	1.40(0.85-2.08)	1.36(0.84-2.02)	-0.08(-0.15, -0.02)	-0.02(-0.10, 0.08)	0.05(-0.02, 0.12)	-0.28(-0.44, -0.13)
Alzheimer's disease and other dementias	45, 959.51(16, 779.34-108, 738.18)	47, 470.20(19, 240.98-108, 365.49)	0.06(-0.14, 0.26)	-0.13(-0.27, 0.01)	-0.05(-0.38, 0.29)	0.37(0.01, 0.72)
Animal contact	45.05(34.61-55.97)	16.09(12.21-20.15)	-3.34(-3.86, -2.83)	-3.20(-3.55, -2.85)	-4.20(-5.65, -2.72)	-2.25(-2.75, -1.75)
Anxiety disorders	370.34(240.79-535.92)	363.68(241.10-523.06)	-0.08(-0.32, 0.16)	0.26(-0.12, 0.65)	-0.11(-0.24, 0.02)	-0.35(-0.99, 0.29)
Aortic aneurysm	54.56(37.17-75.01)	66.32(46.63-84.14)	0.61(0.19, 1.04)	2.80(1.82, 3.79)	-1.24(-1.59, -0.89)	0.70(-0.09, 1.49)
Appendicitis	126.11(72.08-169.70)	33.17(21.22-45.21)	-4.25(-4.53, -3.96)	-4.14(-4.34, -3.94)	-5.30(-6.11, -4.49)	-2.69(-2.94, -2.44)
Asthma	2, 150.71(1, 430.43-3, 197.15)	832.22(598.25-1, 070.17)	-3.01(-3.61, -2.39)	-2.65(-3.23, -2.07)	-3.77(-5.44, -2.07)	-2.33(-2.89, -1.76)
Atrial fibrillation and flutter	7, 995.06(5, 786.29-10, 220.89)	7, 593.70(5, 635.41-9, 486.85)	-0.14(-0.58, 0.30)	0.52(0.30, 0.75)	-0.52(-1.12, 0.08)	-0.02(-0.76, 0.73)

Attention-deficit/hyperactivity disorder	0.00(0.00-0.00)	0.00(0.00-0.00)	-0.79(-1.16, -0.42)	-1.64(-2.02, -1.25)	1.48(0.92, 2.05)	-1.92(-2.84, -0.98)
Autism spectrum disorders	10.59(5.90-17.74)	20.52(12.49-31.19)	2.15(2.07, 2.24)	2.13(1.98, 2.29)	2.30(2.23, 2.38)	1.91(1.75, 2.07)
Bacterial skin diseases	1, 049.44(708.29-1, 381.89)	129.91(64.69-291.07)	-6.39(-6.73, -6.04)	-3.57(-4.04, -3.10)	-13.67(-14.22, -13.11)	0.12(-0.71, 0.96)
Bipolar disorder	24.15(13.30-37.39)	23.92(13.75-37.47)	-0.03(-0.14, 0.07)	0.23(0.12, 0.33)	-0.31(-0.60, -0.01)	-0.05(-0.10, 0.01)
Bladder cancer	649.42(500.98-755.78)	516.63(395.96-621.20)	-0.67(-0.93, -0.42)	-1.44(-2.05, -0.84)	-2.38(-2.60, -2.17)	2.15(1.81, 2.49)
Blindness and vision loss	4, 206.87(3, 051.50-5, 641.26)	3, 482.65(2, 506.78-4, 684.50)	-0.50(-0.65, -0.34)	-0.32(-0.45, -0.20)	-1.03(-1.18, -0.88)	0.03(-0.38, 0.43)
Brain and central nervous system cancer	149.38(111.37-188.17)	199.26(120.88-256.11)	1.04(0.80, 1.28)	-0.31(-0.72, 0.09)	2.39(1.96, 2.83)	0.88(0.51, 1.26)
Breast cancer	653.47(512.47-767.86)	593.93(422.16-759.43)	-0.19(-0.65, 0.27)	1.65(0.91, 2.40)	-2.58(-3.04, -2.12)	1.09(0.10, 2.10)
Cardiomyopathy and myocarditis	946.65(693.77-1, 387.03)	1, 481.64(984.44-2, 044.30)	1.68(0.37, 3.01)	4.95(4.32, 5.57)	2.70(-1.21, 6.77)	-2.76(-3.46, -2.06)
Cervical cancer	246.69(187.72-307.57)	206.10(138.43-269.45)	-0.45(-0.85, -0.04)	-0.25(-0.65, 0.15)	-1.12(-1.93, -0.30)	-0.25(-0.82, 0.32)
Chronic kidney disease	7, 244.49(6, 110.12-8, 715.60)	6, 036.96(4, 728.03-7, 496.31)	-0.59(-0.96, -0.23)	-0.68(-0.98, -0.39)	-0.69(-1.70, 0.33)	-0.50(-0.67, -0.33)
Chronic obstructive pulmonary disease	109, 549.36(82, 254.47-124, 574.79)	44, 670.33(33, 333.27-55, 211.91)	-2.90(-3.48, -2.31)	-2.20(-2.84, -1.56)	-3.70(-4.26, -3.13)	-2.14(-3.00, -1.28)
Cirrhosis and other chronic liver diseases	1, 456.95(1, 071.09-1, 914.08)	709.93(512.84-916.33)	-2.32(-2.66, -1.99)	-3.12(-3.52, -2.72)	-3.02(-3.70, -2.35)	-0.90(-1.32, -0.48)
Colon and rectum cancer	2, 460.83(1, 970.41-2, 907.64)	2, 074.80(1, 518.74-2, 555.45)	-0.42(-0.67, -0.17)	-0.35(-0.89, 0.19)	-2.34(-2.51, -2.17)	2.12(1.59, 2.66)
Conflict and terrorism	0.02(0.01-0.04)	0.07(0.04-0.10)	4.09(2.17, 6.06)	4.59(-0.36, 9.79)	7.39(5.82, 8.99)	-0.21(-1.87, 1.49)
Congenital birth defects	15.70(9.63-23.60)	18.35(11.91-27.15)	0.45(0.12, 0.78)	1.13(0.91, 1.35)	0.33(-0.64, 1.31)	-0.17(-0.35, 0.01)
Cystic echinococcosis	5.86(2.38-10.64)	1.74(0.86-2.77)	-3.83(-4.36, -3.30)	-3.87(-4.42, -3.31)	-4.15(-5.25, -3.05)	-3.59(-4.15, -3.03)
Cysticercosis	118.04(47.07-223.05)	61.88(20.49-138.95)	-2.02(-2.31, -1.72)	-1.99(-2.07, -1.90)	-3.30(-4.00, -2.60)	-0.48(-1.09, 0.12)
Decubitus ulcer	156.21(83.27-399.42)	351.69(200.16-470.76)	3.41(2.16, 4.67)	10.82(9.47, 12.19)	1.56(-0.83, 4.00)	-1.89(-3.84, 0.09)
Dengue	0.20(0.08-0.36)	0.20(0.09-0.46)	-0.37(-1.49, 0.76)	-4.93(-6.30, -3.54)	-0.62(-1.52, 0.28)	4.07(1.21, 7.00)
Depressive disorders	959.12(599.64-1, 430.54)	939.20(569.19-1, 393.85)	-0.02(-0.21, 0.18)	0.25(0.20, 0.30)	0.15(0.08, 0.23)	-0.46(-1.07, 0.17)
Dermatitis	81.11(43.59-141.87)	79.74(42.77-138.50)	-0.06(-0.06, -0.05)	-0.05(-0.06, -0.04)	0.02(0.00, 0.04)	-0.14(-0.15, -0.13)

Diabetes mellitus	2, 443.14(2, 049.11-2, 820.53)	2, 755.16(2, 192.30-3, 279.23)	0.50(-0.20, 1.20)	1.39(0.55, 2.25)	-0.78(-2.65, 1.13)	0.91(0.57, 1.25)
Diarrheal diseases	1, 177.47(461.79-2, 026.53)	111.95(64.15-218.35)	-7.40(-7.56, -7.25)	-9.32(-9.52, -9.12)	-7.59(-7.82, -7.35)	-4.79(-5.13, -4.46)
Dietary iron deficiency	929.30(566.23-1, 352.60)	660.44(375.15-990.92)	-1.10(-1.19, -1.02)	0.85(0.63, 1.07)	-2.48(-2.59, -2.36)	-1.47(-1.55, -1.40)
Drowning	356.63(288.70-417.17)	295.80(229.62-356.01)	-0.49(-0.96, -0.01)	-0.79(-1.11, -0.46)	0.39(-0.96, 1.75)	-0.67(-1.17, -0.17)
Drug use disorders	79.89(63.17-98.89)	31.17(24.29-39.58)	-3.06(-3.25, -2.86)	-2.76(-3.03, -2.48)	-5.04(-5.41, -4.67)	-0.68(-0.97, -0.39)
Encephalitis	17.67(13.96-21.46)	15.35(11.52-19.64)	-0.54(-1.22, 0.14)	0.13(-0.68, 0.95)	0.50(-0.44, 1.45)	-1.99(-3.01, -0.96)
Endocarditis	133.42(57.97-189.79)	50.67(30.79-72.58)	-3.07(-3.46, -2.69)	-0.37(-0.83, 0.09)	-7.59(-8.52, -6.66)	-0.83(-1.29, -0.37)
Endocrine, metabolic, blood, and immune disorders	841.30(610.97-1, 134.75)	757.47(502.43-991.15)	-0.31(-0.56, -0.05)	-1.22(-1.69, -0.74)	-0.10(-0.29, 0.10)	0.44(-0.13, 1.01)
Environmental heat and cold exposure	346.01(120.20-501.82)	140.32(58.53-191.77)	-2.85(-3.54, -2.15)	-4.67(-5.14, -4.19)	-2.95(-4.94, -0.92)	-0.31(-0.91, 0.29)
Esophageal cancer	1, 766.51(1, 127.94-2, 161.55)	1, 288.84(862.86-1, 629.15)	-0.99(-1.42, -0.56)	-1.08(-1.35, -0.81)	-1.47(-2.71, -0.22)	-0.03(-0.38, 0.32)
Exposure to forces of nature	17.43(14.89-20.90)	9.20(6.87-12.34)	3.92(-1.83, 9.99)	3.92(-1.83, 9.99)	3.92(-1.83, 9.99)	3.92(-1.83, 9.99)
Exposure to mechanical forces	128.86(101.71-183.63)	158.34(108.31-198.48)	0.76(0.45, 1.08)	-0.48(-0.87, -0.09)	2.83(2.29, 3.37)	-0.85(-1.45, -0.25)
Eye cancer	10.51(6.22-16.22)	11.49(3.89-18.21)	0.34(-0.27, 0.96)	-1.52(-2.16, -0.87)	2.15(1.02, 3.29)	0.81(-0.50, 2.14)
Falls	7, 270.10(5, 901.05-8, 857.60)	9, 673.72(6, 475.75-12, 439.42)	1.06(0.77, 1.36)	0.47(0.20, 0.75)	-0.05(-0.61, 0.52)	2.89(2.42, 3.35)
Fire, heat, and hot substances	499.57(358.11-602.29)	263.96(188.33-326.98)	-2.15(-2.69, -1.61)	-3.91(-4.36, -3.45)	-0.61(-2.03, 0.82)	-1.51(-2.19, -0.82)
Food-borne trematodiases	25.62(15.90-37.11)	25.46(15.81-36.64)	-0.04(-0.16, 0.09)	1.18(1.06, 1.29)	-0.68(-0.89, -0.47)	-0.58(-0.83, -0.33)
Foreign body	203.46(158.45-243.30)	249.83(155.33-319.63)	0.73(0.31, 1.15)	-0.09(-0.47, 0.30)	1.02(0.22, 1.83)	1.01(0.31, 1.71)
Fungal skin diseases	127.51(47.75-281.85)	124.65(46.84-276.24)	-0.08(-0.08, -0.07)	-0.07(-0.07, -0.06)	-0.01(-0.02, -0.01)	-0.16(-0.17, -0.14)
Gallbladder and biliary diseases	2, 423.92(1, 430.01-3, 194.82)	1, 422.67(1, 078.38-1, 988.29)	-1.76(-2.04, -1.49)	-2.37(-2.69, -2.05)	-2.46(-3.00, -1.90)	-0.32(-0.69, 0.05)
Gallbladder and biliary tract cancer	276.74(172.09-353.81)	248.35(150.39-324.44)	-0.39(-1.05, 0.28)	-1.05(-1.50, -0.60)	-0.96(-2.82, 0.95)	1.31(0.66, 1.98)
Gout	84.50(47.13-141.25)	105.01(58.98-172.33)	0.78(0.63, 0.93)	-0.58(-0.75, -0.41)	1.54(1.23, 1.85)	1.24(1.04, 1.44)

Gynecological diseases	179.47(91.90-314.40)	173.19(90.27-295.77)	-0.11(-0.13, -0.08)	-0.19(-0.22, -0.16)	0.32(0.29, 0.35)	-0.52(-0.58, -0.46)
Headache disorders	148.35(17.80-363.31)	155.57(16.62-367.99)	0.15(0.10, 0.20)	-0.05(-0.08, -0.02)	0.76(0.60, 0.91)	-0.24(-0.26, -0.21)
Hemoglobinopathies and hemolytic anemias	507.42(361.03-676.30)	367.23(257.34-498.09)	-1.07(-1.17, -0.97)	-0.16(-0.27, -0.05)	-1.62(-1.72, -1.52)	-1.28(-1.49, -1.06)
HIV/AIDS	2.04(0.49-8.26)	17.76(8.92-32.53)	6.98(2.82, 11.32)	15.39(2.80, 29.53)	-0.43(-1.77, 0.93)	6.82(3.02, 10.75)
Hodgkin lymphoma	15.90(5.77-23.47)	6.82(3.53-9.41)	-2.69(-2.96, -2.43)	-3.01(-3.67, -2.34)	-4.31(-4.48, -4.13)	-0.14(-0.58, 0.31)
Hypertensive heart disease	22, 651.55(16, 885.05-27, 529.60)	13, 009.38(8, 261.00-17, 625.20)	-1.76(-2.15, -1.36)	-3.57(-3.79, -3.35)	-0.98(-2.06, 0.10)	-0.50(-0.98, -0.02)
Idiopathic developmental intellectual disability	3.75(1.98-6.72)	3.20(1.31-5.82)	-0.59(-0.76, -0.42)	1.55(1.09, 2.02)	-1.33(-1.50, -1.15)	-1.92(-2.05, -1.78)
Idiopathic epilepsy	181.63(107.31-279.62)	164.28(93.23-266.69)	-0.26(-0.37, -0.15)	0.19(0.12, 0.26)	-1.53(-1.63, -1.42)	0.80(0.45, 1.16)
Inflammatory bowel disease	514.68(239.11-727.81)	237.05(160.60-381.31)	-2.51(-2.82, -2.21)	-1.34(-1.73, -0.96)	-4.57(-5.07, -4.07)	-1.58(-2.01, -1.14)
Inguinal, femoral, and abdominal hernia	68.68(52.37-96.10)	44.42(32.74-57.46)	-1.39(-2.19, -0.59)	-2.17(-2.83, -1.51)	-2.55(-4.12, -0.95)	0.63(-0.03, 1.28)
Interpersonal violence	112.81(88.98-140.96)	54.25(42.03-69.20)	-2.42(-2.68, -2.16)	-2.42(-2.59, -2.24)	-4.24(-4.82, -3.66)	-0.33(-0.67, 0.02)
Interstitial lung disease and pulmonary sarcoidosis	102.51(73.95-155.22)	124.71(73.69-172.51)	0.68(0.32, 1.04)	-1.49(-2.04, -0.93)	2.72(1.91, 3.53)	1.06(0.58, 1.55)
Intestinal nematode infections	39.15(22.89-59.56)	1.32(0.80-2.01)	-10.49(-10.78, -10.21)	-12.26(-12.88, -11.63)	-12.35(-12.74, -11.96)	-5.71(-6.05, -5.38)
Invasive Non-typhoidal Salmonella (iNTS)	2.01(0.06-6.75)	1.47(0.03-5.77)	-0.95(-1.33, -0.56)	-1.98(-2.36, -1.60)	-0.98(-1.77, -0.17)	-0.05(-0.56, 0.47)
Iodine deficiency	10.39(5.77-17.27)	9.73(4.53-18.03)	-0.18(-0.41, 0.05)	-2.47(-2.98, -1.95)	2.03(1.66, 2.40)	-0.69(-0.80, -0.57)
Ischemic heart disease	58, 955.84(47, 876.51-68, 139.71)	75, 341.52(56, 777.13-90, 381.29)	0.84(0.27, 1.42)	0.03(-0.40, 0.45)	2.52(1.30, 3.75)	-0.90(-1.33, -0.46)
Kidney cancer	107.85(85.80-130.58)	116.60(89.43-141.08)	0.27(-0.12, 0.67)	-0.65(-1.68, 0.38)	-1.13(-1.36, -0.89)	2.82(2.35, 3.30)
Larynx cancer	74.74(57.22-95.37)	64.38(45.48-83.11)	-0.37(-1.13, 0.39)	-1.78(-3.97, 0.46)	-0.48(-0.87, -0.09)	1.22(0.83, 1.61)
Leishmaniasis	1.14(0.26-3.15)	0.13(0.03-0.34)	-6.61(-7.38, -5.84)	-10.88(-12.32, -9.41)	-2.18(-3.73, -0.60)	-5.81(-6.22, -5.40)
Leprosy	0.27(0.16-0.41)	0.08(0.05-0.12)	-3.87(-4.01, -3.73)	-3.51(-3.63, -3.39)	-4.97(-5.13, -4.80)	-3.09(-3.51, -2.67)

Leukemia	169.23(127.60-212.26)	136.38(98.33-174.83)	-0.59(-0.79, -0.39)	0.57(0.33, 0.81)	-1.87(-2.16, -1.57)	-0.37(-0.82, 0.08)
Lip and oral cavity cancer	129.56(102.23-154.76)	134.98(101.27-163.75)	0.15(-0.53, 0.83)	-0.38(-2.35, 1.63)	-0.51(-0.90, -0.12)	1.47(1.08, 1.87)
Liver cancer	353.87(270.10-442.84)	532.50(398.16-660.56)	1.64(0.30, 2.99)	-0.12(-0.67, 0.44)	3.77(-0.17, 7.87)	1.47(0.44, 2.51)
Low back pain	1, 680.24(1, 040.05-2, 490.11)	1, 534.80(958.38-2, 241.98)	-0.31(-0.36, -0.27)	-0.26(-0.29, -0.24)	-0.01(-0.11, 0.08)	-0.68(-0.77, -0.59)
Lower extremity peripheral arterial disease	189.90(110.75-307.28)	213.98(133.80-328.15)	0.39(0.15, 0.63)	1.52(1.29, 1.76)	-0.12(-0.54, 0.30)	-0.19(-0.65, 0.28)
Lower respiratory infections	29, 886.18(21, 194.01-35, 477.83)	12, 689.90(9, 216.20-16, 661.15)	-2.85(-3.19, -2.52)	-2.09(-2.42, -1.77)	-4.12(-4.63, -3.60)	-2.13(-2.88, -1.37)
Malignant neoplasm of bone and articular cartilage	35.58(20.17-66.61)	64.89(38.13-91.18)	2.23(1.59, 2.88)	3.44(2.65, 4.24)	3.88(2.44, 5.34)	-1.73(-2.10, -1.35)
Malignant skin melanoma	38.55(25.79-69.75)	46.94(20.36-65.49)	0.48(0.06, 0.90)	0.08(-0.14, 0.31)	0.08(-0.14, 0.31)	1.45(0.14, 2.77)
Meningitis	240.08(195.74-293.45)	94.51(69.90-114.39)	-2.89(-3.69, -2.09)	-3.98(-5.03, -2.92)	-2.35(-4.45, -0.20)	-2.06(-2.59, -1.53)
Mesothelioma	11.56(8.33-14.49)	13.49(9.40-17.23)	0.45(0.04, 0.87)	-0.24(-0.48, 0.00)	2.90(2.07, 3.75)	-1.00(-1.85, -0.14)
Motor neuron disease	2.23(1.62-3.02)	4.02(2.88-5.12)	1.88(1.52, 2.24)	1.56(1.15, 1.98)	0.13(-0.60, 0.86)	4.63(4.11, 5.16)
Multiple myeloma	9.17(4.78-22.55)	31.06(16.80-43.29)	4.51(3.41, 5.62)	12.54(9.17, 16.02)	-0.72(-1.27, -0.18)	3.09(1.96, 4.23)
Multiple sclerosis	0.94(0.56-1.42)	1.31(0.97-1.69)	1.10(0.51, 1.70)	2.61(2.40, 2.82)	0.35(-0.54, 1.26)	1.19(-0.13, 2.53)
Nasopharynx cancer	183.43(143.69-220.87)	86.90(63.58-108.89)	-2.41(-2.82, -2.00)	-0.19(-1.18, 0.81)	-6.33(-6.86, -5.80)	-0.63(-1.07, -0.18)
Neck pain	303.36(165.07-536.46)	301.00(164.87-533.79)	-0.01(-0.09, 0.07)	0.08(-0.17, 0.33)	-0.05(-0.07, -0.03)	-0.05(-0.07, -0.03)
Neonatal disorders	10.59(6.99-15.44)	9.39(6.28-13.48)	-0.37(-0.52, -0.22)	-1.35(-1.76, -0.93)	-0.39(-0.53, -0.25)	0.69(0.59, 0.79)
Neuroblastoma and other peripheral nervous cell tumors	0.90(0.48-1.81)	4.25(2.14-6.02)	5.11(4.54, 5.68)	2.66(2.08, 3.23)	7.35(6.18, 8.54)	5.05(4.46, 5.63)
Non-Hodgkin lymphoma	176.57(138.45-215.50)	179.01(125.71-221.49)	0.09(-0.19, 0.37)	0.34(-0.28, 0.95)	-1.16(-1.35, -0.96)	1.50(0.92, 2.09)
Non-melanoma skin cancer	227.90(170.88-310.51)	385.74(257.77-479.37)	1.72(1.06, 2.39)	1.49(0.92, 2.07)	3.18(1.72, 4.66)	-0.47(-0.82, -0.12)
Non-rheumatic valvular heart disease	200.55(145.87-262.03)	168.85(126.75-222.62)	-0.57(-0.77, -0.37)	0.41(0.28, 0.54)	-1.98(-2.16, -1.79)	0.09(-0.52, 0.72)

Oral disorders	1, 360.52(837.36-1, 945.64)	1, 404.46(905.17-1, 971.72)	0.17(0.02, 0.32)	-0.07(-0.26, 0.12)	0.70(0.50, 0.89)	-0.01(-0.31, 0.29)
Osteoarthritis	1, 341.35(671.54-2, 654.56)	1, 524.90(773.32-3, 037.59)	0.41(0.36, 0.46)	-0.10(-0.23, 0.03)	1.03(0.97, 1.10)	0.20(0.16, 0.24)
Other cardiovascular and circulatory diseases	437.76(317.69-617.37)	349.50(247.18-468.47)	-0.73(-1.21, -0.25)	-0.98(-1.39, -0.58)	-1.55(-2.48, -0.61)	0.36(-0.09, 0.82)
Other chronic respiratory diseases	102.28(65.26-245.82)	206.72(127.36-285.83)	2.42(1.84, 3.00)	4.64(3.13, 6.17)	6.07(5.46, 6.67)	-3.21(-3.56, -2.87)
Other digestive diseases	1, 306.16(732.22-1, 710.86)	495.53(357.43-735.67)	-3.08(-3.51, -2.65)	-3.67(-4.67, -2.67)	-4.35(-4.76, -3.94)	-0.56(-1.27, 0.16)
Other intestinal infectious diseases	5.10(0.13-17.34)	2.14(0.07-6.46)	-2.74(-3.33, -2.15)	-2.98(-3.59, -2.37)	-3.09(-4.32, -1.84)	-2.28(-2.89, -1.67)
Other malignant neoplasms	315.55(194.17-429.53)	227.68(167.78-292.07)	-0.98(-1.29, -0.67)	-2.01(-2.75, -1.26)	-1.33(-1.62, -1.04)	0.64(0.39, 0.89)
Other mental disorders	242.25(156.84-357.26)	237.07(153.58-349.60)	-0.08(-0.10, -0.05)	-0.10(-0.11, -0.10)	-0.05(-0.06, -0.03)	-0.10(-0.19, -0.02)
Other musculoskeletal disorders	249.31(92.11-536.10)	208.28(134.35-320.97)	-0.67(-1.27, -0.08)	0.18(-0.76, 1.13)	3.60(2.91, 4.29)	-5.59(-6.64, -4.52)
Other neglected tropical diseases	39.51(27.08-54.95)	28.71(18.81-40.91)	-1.02(-1.27, -0.76)	0.38(-0.26, 1.03)	-1.91(-2.20, -1.61)	-1.48(-1.81, -1.16)
Other neoplasms	29.47(19.32-40.26)	49.94(35.48-71.41)	1.71(1.15, 2.27)	0.58(0.06, 1.11)	2.46(1.37, 3.57)	1.71(1.51, 1.92)
Other neurological disorders	33.83(24.85-44.98)	121.92(95.94-148.99)	4.40(3.79, 5.02)	5.44(5.01, 5.88)	3.54(2.70, 4.39)	4.52(3.66, 5.39)
Other nutritional deficiencies	232.45(166.12-310.14)	292.51(200.41-385.76)	1.10(0.21, 2.00)	-1.54(-2.67, -0.40)	4.61(2.72, 6.53)	-1.38(-1.82, -0.94)
Other pharynx cancer	23.74(17.84-31.28)	21.12(14.00-27.52)	-0.30(-1.05, 0.45)	-1.73(-3.69, 0.27)	-0.29(-0.75, 0.18)	1.29(0.19, 2.40)
Other sense organ diseases	105.64(54.45-188.18)	107.33(54.42-189.37)	0.04(0.01, 0.06)	0.08(0.01, 0.15)	0.15(0.12, 0.18)	-0.14(-0.16, -0.12)
Other skin and subcutaneous diseases	144.16(83.16-238.56)	144.25(79.02-238.32)	0.05(-0.06, 0.15)	-0.85(-0.95, -0.75)	0.45(0.24, 0.66)	0.43(0.29, 0.57)
Other transport injuries	79.77(61.72-102.08)	58.50(43.75-75.92)	-1.01(-1.20, -0.82)	-0.83(-1.29, -0.36)	-0.85(-1.00, -0.70)	-1.53(-1.68, -1.39)
Other unintentional injuries	179.67(137.00-260.97)	106.61(80.12-138.10)	-1.66(-1.93, -1.38)	-1.50(-1.91, -1.10)	-1.17(-1.60, -0.73)	-2.63(-3.07, -2.18)
Other unspecified infectious diseases	119.69(62.65-169.67)	70.90(44.20-99.04)	-1.79(-2.09, -1.48)	-1.75(-1.98, -1.52)	-3.43(-3.67, -3.18)	0.27(-0.70, 1.24)
Otitis media	19.31(9.66-36.46)	15.87(8.01-29.53)	-0.64(-0.66, -0.62)	-0.77(-0.81, -0.74)	-0.48(-0.51, -0.46)	-0.65(-0.67, -0.63)
Ovarian cancer	91.83(67.52-120.98)	79.09(52.39-103.42)	-0.38(-1.13, 0.38)	2.07(0.57, 3.59)	-3.41(-4.09, -2.73)	1.06(-0.43, 2.56)
Pancreatic cancer	416.44(327.74-497.98)	552.30(400.67-689.26)	0.92(0.66, 1.19)	0.86(0.54, 1.18)	-1.04(-1.42, -0.66)	3.51(2.90, 4.13)

Pancreatitis	219.71(136.05-334.17)	162.71(107.35-228.98)	-0.94(-1.34, -0.54)	-2.03(-2.46, -1.60)	-1.09(-1.93, -0.25)	0.12(-0.32, 0.56)
Paralytic ileus and intestinal obstruction	1, 023.98(745.21-1, 323.19)	457.46(317.56-606.73)	-2.50(-3.10, -1.89)	-4.37(-4.84, -3.91)	-3.14(-4.34, -1.93)	-0.30(-0.82, 0.23)
Parkinson's disease	1, 872.24(1, 468.07-2, 236.98)	2, 070.07(1, 631.01-2, 530.88)	0.33(-0.11, 0.76)	-0.01(-0.33, 0.32)	-0.02(-0.87, 0.85)	0.95(0.56, 1.34)
Pneumoconiosis	47.47(34.19-62.00)	41.82(32.70-52.57)	-0.58(-1.11, -0.04)	-3.13(-3.90, -2.35)	0.32(-0.49, 1.13)	0.81(-0.31, 1.93)
Poisonings	60.59(41.73-130.37)	73.38(43.13-93.62)	0.82(0.26, 1.37)	-0.21(-1.24, 0.83)	3.54(2.54, 4.56)	-1.79(-2.22, -1.36)
Police conflict and executions	4.69(1.08-7.74)	1.76(1.04-2.63)	-3.12(-3.89, -2.35)	-4.48(-6.28, -2.64)	-5.12(-5.57, -4.67)	0.46(-0.76, 1.70)
Prostate cancer	264.53(166.20-350.13)	371.87(217.00-513.62)	1.32(-0.40, 3.07)	-0.69(-2.42, 1.07)	0.09(-3.08, 3.36)	3.90(2.37, 5.47)
Protein-energy malnutrition	5, 160.27(4, 117.14-6, 089.15)	1, 538.79(1, 121.21-1, 887.04)	-3.82(-4.35, -3.28)	-6.01(-6.84, -5.17)	-4.09(-4.40, -3.78)	-0.96(-2.40, 0.51)
Pruritus	23.85(10.32-46.87)	27.08(11.95-53.09)	0.40(0.39, 0.42)	0.41(0.36, 0.46)	0.54(0.52, 0.56)	0.24(0.23, 0.26)
Psoriasis	30.42(22.43-39.30)	39.61(29.87-50.97)	0.86(0.81, 0.91)	1.43(1.33, 1.54)	0.72(0.64, 0.80)	0.33(0.30, 0.37)
Pulmonary Arterial Hypertension	171.31(105.31-306.59)	189.06(122.87-265.02)	0.37(-0.08, 0.82)	-0.26(-0.60, 0.08)	4.09(3.00, 5.18)	-2.37(-3.08, -1.64)
Rabies	1.61(0.41-3.59)	1.27(0.39-2.70)	-2.15(-4.86, 0.65)	0.40(-3.34, 4.28)	9.80(2.20, 17.96)	-18.00(-19.33, -16.65)
Rheumatic heart disease	5, 608.59(4, 219.82-7, 557.61)	1, 755.34(1, 160.44-2, 311.73)	-3.74(-4.24, -3.23)	-3.80(-4.37, -3.23)	-3.90(-4.88, -2.92)	-3.69(-4.43, -2.96)
Rheumatoid arthritis	263.47(210.52-316.60)	285.67(218.00-349.58)	0.26(-0.19, 0.71)	0.06(-0.59, 0.71)	1.14(0.42, 1.87)	-1.00(-1.30, -0.70)
Road injuries	488.45(413.36-583.98)	553.34(448.69-649.91)	0.52(0.18, 0.85)	1.15(0.71, 1.58)	0.69(0.14, 1.25)	-0.84(-1.33, -0.34)
Scabies	140.31(74.19-238.38)	137.34(72.30-231.79)	-0.05(-0.09, -0.02)	0.03(0.00, 0.07)	-0.09(-0.20, 0.02)	-0.11(-0.15, -0.07)
Schistosomiasis	30.71(11.47-62.39)	8.42(4.60-13.01)	-4.16(-4.40, -3.92)	-4.96(-5.15, -4.77)	-4.96(-5.15, -4.77)	-2.18(-2.88, -1.48)
Schizophrenia	24.46(16.21-33.48)	32.92(22.65-44.69)	0.97(0.80, 1.13)	1.07(0.86, 1.28)	0.97(0.52, 1.42)	0.89(0.80, 0.98)
Self-harm	997.61(591.38-1, 172.92)	476.14(367.14-585.41)	-2.44(-2.95, -1.93)	-2.33(-2.71, -1.96)	-3.57(-5.01, -2.11)	-0.93(-1.32, -0.54)
Sexually transmitted infections excluding HIV	20.09(12.73-28.13)	11.40(7.62-16.20)	-1.80(-2.31, -1.30)	-3.91(-5.25, -2.56)	-0.39(-0.80, 0.02)	-1.36(-1.61, -1.11)
Soft tissue and other extraosseous sarcomas	34.69(21.13-48.29)	25.70(16.98-34.99)	-1.02(-1.60, -0.43)	-1.23(-1.70, -0.75)	-2.41(-3.56, -1.24)	0.75(0.27, 1.23)

Stomach cancer	3, 744.07(2, 935.53-4, 523.73)	2, 544.40(1, 817.92-3, 119.12)	-1.20(-1.59, -0.82)	-1.45(-1.84, -1.05)	-1.50(-2.27, -0.71)	-0.85(-1.37, -0.33)
Stroke	72, 523.79(57, 604.07-83, 041.57)	50, 840.45(39, 528.31-60, 880.89)	-1.08(-1.57, -0.57)	-0.24(-0.87, 0.39)	-1.76(-2.51, -1.01)	-1.37(-2.24, -0.49)
Testicular cancer	5.30(4.18-6.59)	6.10(4.69-7.40)	0.54(-0.63, 1.72)	-0.64(-2.09, 0.82)	-2.80(-3.91, -1.69)	4.55(2.90, 6.22)
Tetanus	30.77(7.56-54.48)	1.52(0.33-3.68)	-9.45(-10.62, -8.27)	-9.79(-10.72, -8.84)	-10.15(-12.35, -7.90)	-8.90(-10.32, -7.46)
Thyroid cancer	64.72(48.68-82.80)	55.19(40.84-69.28)	-0.45(-0.74, -0.16)	0.19(-0.43, 0.81)	-1.81(-2.16, -1.45)	0.62(0.20, 1.04)
Tracheal, bronchus, and lung cancer	2, 988.62(2, 395.71-3, 554.08)	3, 956.34(2, 972.29-4, 767.60)	0.97(0.68, 1.26)	1.17(0.53, 1.81)	0.04(-0.16, 0.23)	2.01(1.38, 2.64)
Trachoma	12.56(5.55-24.61)	1.63(0.55-3.58)	-6.33(-6.83, -5.83)	-6.68(-7.89, -5.45)	-8.40(-8.69, -8.10)	-3.11(-4.05, -2.15)
Tuberculosis	2, 699.36(2, 174.55-3, 139.70)	178.55(136.48-219.03)	-8.79(-9.73, -7.84)	-6.68(-7.51, -5.84)	-10.97(-13.47, -8.40)	-9.27(-9.86, -8.68)
Typhoid and paratyphoid	0.22(0.06-0.52)	0.15(0.05-0.31)	-1.28(-2.20, -0.35)	-2.59(-3.51, -1.66)	1.29(-1.31, 3.94)	-2.53(-2.98, -2.08)
Upper digestive system diseases	3, 536.19(2, 721.76-4, 279.00)	1, 587.48(1, 171.12-2, 003.62)	-2.50(-2.87, -2.12)	-3.52(-3.93, -3.11)	-2.08(-2.83, -1.32)	-2.51(-2.99, -2.02)
Upper respiratory infections	2, 337.69(471.13-3, 819.55)	172.07(92.36-459.23)	-8.19(-9.41, -6.95)	-3.16(-4.12, -2.18)	-13.91(-15.59, -12.19)	-5.26(-7.35, -3.12)
Urinary diseases and male infertility	683.09(459.39-842.13)	459.63(349.33-623.42)	-1.25(-1.61, -0.88)	-0.27(-0.69, 0.15)	-3.21(-3.77, -2.64)	-0.12(-0.51, 0.27)
Urticaria	20.81(12.40-33.23)	20.34(12.17-32.11)	-0.07(-0.09, -0.04)	-0.02(-0.05, 0.01)	0.07(0.03, 0.10)	-0.26(-0.31, -0.20)
Uterine cancer	87.99(63.74-130.95)	60.60(38.74-84.71)	-1.11(-1.46, -0.76)	1.95(1.74, 2.16)	-2.42(-3.08, -1.76)	-2.27(-2.96, -1.58)
Varicella and herpes zoster	289.86(198.21-422.56)	45.33(28.41-65.46)	-5.96(-6.46, -5.47)	-5.53(-5.88, -5.17)	-6.56(-7.93, -5.16)	-5.63(-6.13, -5.12)
Vascular intestinal disorders	58.31(24.19-77.52)	29.89(16.15-42.96)	-2.12(-2.41, -1.83)	0.54(0.24, 0.84)	-5.50(-5.97, -5.03)	-0.66(-1.21, -0.10)
Viral skin diseases	18.02(11.43-26.17)	17.73(11.36-25.69)	-0.06(-0.08, -0.03)	-0.14(-0.17, -0.10)	-0.08(-0.11, -0.05)	0.03(-0.03, 0.08)
Vitamin A deficiency	1.03(0.44-1.89)	0.90(0.38-1.70)	-0.40(-0.60, -0.20)	-1.76(-2.00, -1.51)	-0.24(-0.55, 0.07)	1.00(0.57, 1.44)

UI, Uncertainty Interval; CI, Confidence Interval.

Supplementary Table 3. The number of disability-adjusted life years for level 3 causes in 1990 and 2021 and average annual percent change between 1990 and 2021, for both sexes, by age group.

	1990	2021	1990-2021	1990-2000	2001-2011	2012-2021
Cause	N(95%UI)	N(95%UI)	AAPC (95%CI)	APC (95%CI)	APC (95%CI)	APC (95%CI)
			Number			
**65-69 years**						
Acne vulgaris	1, 625(1, 032-2, 537)	5, 504(3, 479-8, 599)	4.02(3.86, 4.19)	3.18(2.96, 3.40)	2.45(2.19, 2.71)	6.52(6.14, 6.90)
Acute glomerulonephritis	20, 153(14, 555-27, 035)	16, 675(11, 044-22, 836)	-0.47(-1.25, 0.30)	-1.34(-1.66, -1.02)	-0.87(-3.00, 1.30)	0.68(-0.23, 1.58)
Acute hepatitis	58, 782(47, 280-72, 229)	11, 522(8, 999-14, 949)	-5.01(-5.42, -4.59)	-5.17(-5.60, -4.74)	-9.73(-10.55, -8.90)	1.44(0.86, 2.02)
Adverse effects of medical treatment	13, 870(9, 262-17, 622)	9, 179(7, 206-12, 535)	-1.35(-2.03, -0.68)	-2.04(-2.26, -1.81)	-4.80(-6.73, -2.82)	3.00(2.51, 3.49)
Age-related and other hearing loss	569, 762(359, 717-839, 942)	1, 812, 805(1, 168, 115-2, 669, 640)	3.84(3.66, 4.02)	2.66(2.50, 2.82)	2.22(1.91, 2.53)	6.76(6.30, 7.21)
Alcohol use disorders	37, 053(27, 377-48, 183)	109, 714(79, 406-147, 347)	3.53(3.01, 4.06)	3.08(1.78, 4.40)	3.97(3.61, 4.33)	3.45(2.76, 4.15)
Alopecia areata	2, 083(1, 334-2, 964)	5, 856(3, 766-8, 445)	3.40(3.23, 3.56)	2.44(2.23, 2.65)	1.84(1.58, 2.10)	6.01(5.62, 6.41)
Alzheimer's disease and other dementias	352, 725(151, 848-769, 780)	1, 033, 932(484, 837-2, 353, 029)	3.45(3.11, 3.80)	2.47(2.29, 2.64)	1.89(0.87, 2.92)	6.16(5.98, 6.33)
Animal contact	14, 120(10, 595-16, 747)	9, 015(7, 282-11, 242)	-1.45(-1.73, -1.16)	-0.88(-1.42, -0.34)	-5.63(-6.11, -5.14)	2.43(2.05, 2.80)
Anxiety disorders	138, 710(89, 439-204, 659)	394, 541(257, 326-577, 559)	3.41(3.20, 3.61)	2.95(2.73, 3.17)	0.47(0.00, 0.95)	7.46(7.18, 7.74)
Aortic aneurysm	6, 271(4, 841-8, 079)	23, 768(18, 523-30, 742)	4.43(3.96, 4.90)	2.93(2.50, 3.37)	4.63(3.31, 5.97)	6.02(5.78, 6.26)
Appendicitis	7, 282(5, 246-8, 970)	4, 612(3, 398-5, 978)	-1.41(-1.77, -1.05)	-1.12(-1.29, -0.95)	-4.07(-4.91, -3.22)	1.36(0.92, 1.81)
Asthma	137, 923(106, 821-185, 005)	96, 794(74, 684-123, 104)	-1.20(-1.43, -0.97)	-1.28(-1.41, -1.15)	-3.83(-4.40, -3.25)	1.85(1.56, 2.14)
Atrial fibrillation and flutter	63, 502(45, 753-84, 058)	188, 205(131, 832-256, 666)	3.56(3.21, 3.92)	1.84(1.55, 2.13)	2.32(1.58, 3.06)	6.49(6.30, 6.68)
Attention-deficit/hyperactivity disorder	470(219-863)	1, 158(506-2, 147)	3.00(2.80, 3.19)	1.58(1.14, 2.01)	1.67(1.35, 1.98)	5.89(5.75, 6.03)
Autism spectrum disorders	23, 797(16, 369-33, 538)	75, 960(52, 199-107, 501)	3.87(3.71, 4.04)	3.25(3.03, 3.47)	2.22(1.95, 2.48)	6.22(5.83, 6.61)

Bacterial skin diseases	9, 307(7, 347-11, 151)	5, 640(4, 268-7, 543)	-1.57(-1.86, -1.29)	-0.57(-0.92, -0.22)	-8.69(-9.26, -8.11)	4.92(4.39, 5.46)
Bipolar disorder	12, 977(8, 132-19, 634)	36, 710(23, 190-55, 918)	3.39(3.23, 3.56)	2.45(2.24, 2.66)	1.81(1.55, 2.07)	6.02(5.63, 6.41)
Bladder cancer	80, 335(60, 443-95, 685)	141, 417(107, 281-184, 738)	1.80(1.57, 2.04)	1.27(1.07, 1.48)	-0.50(-1.03, 0.04)	4.87(4.49, 5.25)
Blindness and vision loss	308, 011(203, 590-472, 478)	877, 659(540, 307-1, 440, 882)	3.57(3.10, 4.04)	3.97(2.66, 5.30)	1.19(0.71, 1.66)	5.90(5.54, 6.26)
Brain and central nervous system cancer	82, 212(62, 620-107, 653)	233, 590(174, 251-303, 140)	3.52(3.24, 3.81)	3.47(3.28, 3.65)	1.38(0.57, 2.20)	5.77(5.57, 5.98)
Breast cancer	101, 342(84, 308-120, 630)	322, 350(254, 454-406, 127)	3.90(3.56, 4.25)	3.02(2.68, 3.35)	1.96(1.15, 2.77)	7.15(6.68, 7.62)
Cardiomyopathy and myocarditis	24, 665(19, 944-35, 974)	62, 401(49, 465-80, 274)	3.06(2.67, 3.46)	4.73(3.66, 5.82)	1.33(1.04, 1.61)	3.05(2.62, 3.49)
Cervical cancer	82, 621(67, 482-99, 617)	164, 455(121, 493-213, 776)	2.41(1.73, 3.09)	-0.09(-0.77, 0.60)	1.26(-0.40, 2.95)	5.93(5.55, 6.31)
Chronic kidney disease	372, 865(318, 305-436, 148)	796, 257(655, 931-954, 597)	2.52(2.32, 2.72)	1.62(1.35, 1.90)	1.24(0.75, 1.73)	4.59(4.47, 4.71)
Chronic obstructive pulmonary disease	3, 832, 244(3, 305, 452-4, 302, 442)	2, 817, 958(2, 334, 610-3, 379, 482)	-1.13(-1.53, -0.74)	-1.06(-1.29, -0.83)	-3.85(-4.79, -2.89)	1.85(1.30, 2.41)
Cirrhosis and other chronic liver diseases	481, 730(406, 937-561, 047)	495, 067(392, 421-615, 174)	0.05(-0.27, 0.38)	0.05(-0.09, 0.19)	-1.74(-2.63, -0.85)	2.06(1.73, 2.39)
Colon and rectum cancer	393, 287(331, 542-452, 843)	992, 168(798, 055-1, 203, 275)	3.10(2.74, 3.47)	2.34(2.17, 2.52)	1.52(0.52, 2.54)	5.65(5.32, 5.98)
Conflict and terrorism	55(38-80)	153(114-205)	3.98(-14.26, 26.11)	23.30(9.88, 38.36)	-3.55(-45.38, 70.31)	-9.55(-19.84, 2.06)
Congenital birth defects	14, 000(10, 556-18, 333)	34, 557(25, 814-46, 279)	2.97(2.74, 3.19)	3.49(2.96, 4.01)	0.92(0.56, 1.29)	4.54(4.37, 4.72)
COVID-19	NA	2, 015(295-7, 076)	NA	NA	NA	NA
Cystic echinococcosis	602(413-801)	706(426-1, 137)	0.55(0.24, 0.85)	-2.57(-2.91, -2.23)	0.16(-0.50, 0.82)	3.82(3.54, 4.09)
Cysticercosis	15, 785(7, 349-29, 242)	21, 926(8, 762-47, 769)	1.05(0.64, 1.47)	0.99(0.61, 1.37)	-2.17(-2.69, -1.64)	4.69(3.50, 5.89)
Decubitus ulcer	889(523-1, 664)	4, 586(2, 553-5, 970)	5.41(4.17, 6.65)	7.87(4.29, 11.58)	3.85(3.41, 4.29)	4.14(3.01, 5.28)
Dengue	10(5-18)	18(8-36)	1.40(0.42, 2.38)	-2.08(-3.43, -0.70)	0.07(-1.79, 1.96)	5.78(5.04, 6.53)
Depressive disorders	222, 011(153, 824-302, 767)	752, 421(529, 075-1, 030, 624)	4.13(3.72, 4.55)	4.39(3.30, 5.49)	2.21(1.68, 2.75)	5.93(5.66, 6.19)
Dermatitis	26, 383(15, 045-44, 929)	74, 247(42, 085-126, 897)	3.39(3.23, 3.56)	2.51(2.30, 2.71)	1.81(1.56, 2.06)	5.96(5.58, 6.34)
Diabetes mellitus	533, 891(442, 411-643, 476)	1, 643, 113(1, 293, 687-2, 065, 652)	3.69(3.40, 3.99)	3.89(3.66, 4.13)	1.43(0.76, 2.11)	5.96(5.63, 6.29)

Diarrheal diseases	39, 626(20, 134-57, 730)	12, 016(8, 210-18, 699)	-3.84(-4.36, -3.32)	-5.72(-5.96, -5.48)	-6.42(-7.45, -5.38)	1.58(1.08, 2.08)
Dietary iron deficiency	85, 499(57, 577-123, 901)	90, 054(56, 904-136, 432)	0.19(0.02, 0.35)	0.14(-0.23, 0.51)	-2.79(-3.03, -2.54)	3.41(3.23, 3.60)
Drowning	56, 301(48, 365-64, 576)	89, 463(74, 895-106, 381)	1.65(0.83, 2.48)	-0.07(-0.72, 0.58)	1.26(-0.97, 3.55)	3.58(3.25, 3.91)
Drug use disorders	51, 852(43, 504-61, 153)	43, 458(32, 591-55, 338)	-0.75(-1.26, -0.24)	-1.21(-1.76, -0.66)	-4.63(-5.49, -3.77)	5.00(4.20, 5.80)
Encephalitis	4, 859(3, 891-5, 812)	9, 114(7, 389-11, 194)	2.06(1.75, 2.37)	1.67(1.11, 2.22)	0.48(-0.03, 0.99)	3.96(3.62, 4.30)
Endocarditis	4, 282(2, 344-5, 578)	3, 844(2, 934-5, 667)	-0.31(-0.77, 0.14)	1.23(0.85, 1.60)	-5.77(-6.94, -4.58)	4.72(4.25, 5.18)
Endocrine, metabolic, blood, and immune disorders	85, 870(54, 517-134, 328)	228, 805(143, 637-351, 574)	3.30(2.73, 3.87)	3.89(3.32, 4.47)	1.39(0.23, 2.57)	4.45(3.22, 5.70)
Environmental heat and cold exposure	12, 864(6, 467-16, 781)	12, 481(6, 981-17, 873)	-0.09(-0.29, 0.11)	-0.76(-1.15, -0.38)	-2.52(-2.91, -2.13)	3.26(3.10, 3.43)
Esophageal cancer	865, 592(693, 545-1, 012, 460)	1, 225, 345(951, 842-1, 512, 056)	1.20(0.68, 1.72)	1.29(1.05, 1.52)	-0.78(-2.17, 0.63)	3.25(2.59, 3.91)
Exposure to forces of nature	2, 244(2, 030-2, 470)	2, 955(2, 463-3, 664)	-2.39(-19.65, 18.58)	-7.22(-13.87, -0.05)	2.25(-42.94, 83.22)	-1.48(-12.89, 11.42)
Exposure to mechanical forces	29, 308(22, 791-41, 939)	79, 103(57, 199-104, 581)	3.35(2.88, 3.81)	2.33(1.44, 3.22)	4.14(3.74, 4.54)	3.35(2.40, 4.32)
Eye cancer	1, 119(685-1, 525)	2, 011(1, 174-2, 755)	1.95(1.69, 2.21)	1.60(1.42, 1.79)	0.29(-0.37, 0.94)	4.25(3.86, 4.63)
Falls	252, 499(199, 935-312, 591)	710, 709(534, 526-916, 130)	3.59(2.95, 4.23)	2.58(2.16, 3.00)	0.29(-1.54, 2.15)	8.17(7.83, 8.51)
Fire, heat, and hot substances	28, 497(22, 622-34, 258)	37, 204(28, 119-48, 424)	0.80(0.50, 1.10)	-0.40(-0.60, -0.20)	-0.16(-1.01, 0.69)	3.13(2.91, 3.35)
Food-borne trematodiases	43, 396(17, 667-88, 027)	59, 120(29, 032-107, 203)	1.05(0.49, 1.62)	-0.95(-1.23, -0.66)	0.26(-0.12, 0.64)	3.30(1.95, 4.67)
Foreign body	13, 603(10, 680-17, 551)	31, 040(21, 208-40, 961)	2.70(2.18, 3.23)	1.75(1.42, 2.08)	-0.32(-1.84, 1.21)	6.90(6.62, 7.19)
Fungal skin diseases	9, 432(3, 695-20, 481)	25, 956(10, 290-56, 611)	3.32(3.17, 3.48)	2.48(2.29, 2.68)	1.70(1.45, 1.94)	5.88(5.51, 6.25)
Gallbladder and biliary diseases	121, 463(83, 474-168, 702)	238, 983(157, 342-346, 916)	2.23(2.05, 2.40)	1.37(1.19, 1.56)	1.01(0.60, 1.42)	4.53(4.28, 4.77)
Gallbladder and biliary tract cancer	64, 479(48, 872-86, 051)	140, 924(96, 966-186, 163)	2.65(2.09, 3.21)	1.82(1.55, 2.09)	1.35(-0.19, 2.92)	5.06(4.48, 5.65)
Gout	19, 154(11, 366-29, 612)	70, 155(41, 343-107, 477)	4.34(4.16, 4.52)	2.05(1.74, 2.36)	4.10(3.91, 4.29)	6.91(6.51, 7.32)
Gynecological diseases	48, 461(31, 013-73, 620)	88, 442(56, 025-129, 674)	1.96(1.77, 2.16)	2.06(1.94, 2.17)	-2.20(-2.60, -1.81)	6.14(5.71, 6.57)
Headache disorders	126, 607(39, 328-253, 726)	378, 648(110, 554-780, 866)	3.66(3.51, 3.81)	2.53(2.34, 2.72)	2.35(1.99, 2.72)	6.26(6.07, 6.44)

Hemoglobinopathies and hemolytic anemias	51, 091(38, 861-66, 754)	63, 884(49, 379-84, 708)	0.69(0.54, 0.84)	0.20(0.09, 0.32)	-1.14(-1.45, -0.82)	3.31(3.12, 3.51)
HIV/AIDS	2, 260(260-3, 582)	73, 898(51, 971-99, 376)	11.06(9.97, 12.15)	16.74(14.87, 18.64)	1.96(0.71, 3.22)	16.19(13.61, 18.82)
Hodgkin lymphoma	11, 114(4, 272-15, 669)	8, 568(4, 979-11, 254)	-0.88(-1.11, -0.65)	-1.54(-1.71, -1.38)	-3.81(-4.31, -3.30)	3.15(2.76, 3.55)
Hypertensive heart disease	727, 679(442, 877-874, 787)	673, 451(487, 709-876, 936)	-0.34(-0.59, -0.08)	-2.41(-2.63, -2.19)	-2.32(-2.83, -1.81)	4.02(3.43, 4.61)
Idiopathic developmental intellectual disability	2, 245(582-4, 659)	4, 503(1, 157-9, 628)	2.29(2.08, 2.50)	2.80(2.23, 3.37)	-0.10(-0.34, 0.13)	4.51(4.32, 4.71)
Idiopathic epilepsy	30, 494(20, 853-43, 921)	64, 885(39, 362-106, 195)	2.48(2.20, 2.75)	2.35(2.11, 2.60)	-0.23(-0.67, 0.21)	5.58(4.87, 6.31)
Inflammatory bowel disease	8, 997(5, 576-11, 660)	13, 260(10, 000-17, 592)	1.32(0.93, 1.72)	1.47(1.29, 1.64)	-0.70(-1.79, 0.40)	3.46(3.14, 3.77)
Inguinal, femoral, and abdominal hernia	6, 062(4, 034-8, 758)	11, 043(6, 529-17, 489)	1.90(1.53, 2.27)	1.18(0.97, 1.39)	0.94(0.12, 1.77)	3.79(2.99, 4.59)
Interpersonal violence	51, 117(42, 601-61, 270)	66, 244(52, 968-82, 011)	0.77(0.53, 1.01)	0.51(0.32, 0.71)	-1.20(-1.71, -0.68)	3.30(2.98, 3.61)
Interstitial lung disease and pulmonary sarcoidosis	13, 200(9, 824-19, 084)	35, 782(25, 958-48, 100)	3.31(3.05, 3.58)	2.20(2.05, 2.34)	3.54(2.74, 4.33)	4.33(4.18, 4.48)
Intestinal nematode infections	18, 547(9, 543-29, 737)	991(485-1, 646)	-9.10(-9.60, -8.60)	-10.24(-10.62, -9.86)	-14.80(-15.84, -13.74)	-0.59(-1.65, 0.49)
Invasive Non-typhoidal Salmonella (iNTS)	954(251-2, 087)	1, 422(371-3, 216)	1.37(1.10, 1.64)	1.07(0.85, 1.30)	-0.99(-1.75, -0.23)	4.12(3.87, 4.36)
Iodine deficiency	5, 264(2, 820-9, 450)	15, 434(7, 250-29, 660)	3.56(3.37, 3.76)	2.32(2.09, 2.55)	3.40(2.97, 3.83)	4.32(4.16, 4.47)
Ischemic heart disease	1, 714, 322(1, 499, 401-1, 964, 586)	4, 369, 215(3, 616, 722-5, 187, 420)	3.11(2.65, 3.58)	2.60(2.13, 3.07)	2.19(0.94, 3.46)	4.32(4.14, 4.50)
Kidney cancer	23, 835(20, 427-27, 113)	86, 068(69, 656-103, 884)	4.24(3.26, 5.23)	3.31(1.27, 5.38)	2.64(2.16, 3.13)	6.68(6.00, 7.36)
Larynx cancer	53, 029(42, 226-63, 636)	86, 713(65, 409-112, 955)	1.61(1.42, 1.79)	0.46(0.32, 0.61)	0.19(-0.34, 0.72)	4.33(4.21, 4.45)
Leishmaniasis	298(16-921)	191(29-499)	-1.50(-2.05, -0.93)	-3.80(-4.32, -3.27)	0.64(-0.23, 1.52)	-1.25(-2.06, -0.43)
Leprosy	62(37-94)	48(27-75)	-0.83(-1.00, -0.66)	-1.04(-1.19, -0.89)	-4.00(-4.23, -3.77)	2.75(2.28, 3.23)
Leukemia	84, 773(68, 612-105, 809)	171, 783(123, 868-218, 305)	2.35(2.14, 2.56)	1.78(1.42, 2.15)	0.68(0.23, 1.14)	4.60(4.46, 4.74)
Lip and oral cavity cancer	34, 083(28, 486-39, 784)	91, 189(69, 752-117, 528)	3.13(2.60, 3.67)	1.21(1.03, 1.39)	3.34(2.63, 4.06)	4.69(3.20, 6.21)
Liver cancer	287, 148(242, 132-338, 256)	634, 895(506, 449-789, 968)	2.69(2.25, 3.13)	3.08(2.55, 3.62)	-0.05(-0.82, 0.73)	4.60(3.76, 5.45)
Low back pain	538, 788(323, 712-812, 833)	1, 208, 478(730, 931-1, 802, 687)	2.72(2.49, 2.95)	1.07(0.52, 1.63)	1.45(1.14, 1.77)	5.76(5.56, 5.95)

Lower extremity peripheral arterial disease	12, 514(6, 515-24, 599)	31, 411(16, 565-61, 072)	3.00(2.72, 3.29)	2.53(2.33, 2.74)	1.18(0.36, 2.01)	5.39(5.21, 5.58)
Lower respiratory infections	298, 228(239, 724-347, 670)	250, 169(201, 595-313, 662)	-0.67(-0.95, -0.38)	-0.75(-1.17, -0.33)	-3.35(-3.98, -2.71)	2.37(2.13, 2.60)
Malaria	642(57-6, 482)	68, 566(40, 084-94, 676)	-19.83(-24.41, -14.97)	-18.12(-21.23, -14.89)	-15.71(-19.31, -11.95)	-26.14(-35.93, -14.85)
Malignant neoplasm of bone and articular cartilage	14, 271(8, 661-25, 724)	14, 454(7, 300-19, 203)	5.51(5.02, 6.00)	6.48(5.60, 7.37)	4.97(4.07, 5.89)	4.42(4.07, 4.78)
Malignant skin melanoma	5, 804(4, 009-7, 632)	12, 170(9, 872-14, 600)	2.98(2.66, 3.30)	2.41(2.04, 2.79)	1.12(0.43, 1.81)	5.65(5.11, 6.19)
Meningitis	17, 785(15, 199-20, 610)	8, 816(6, 993-10, 858)	-1.28(-1.65, -0.91)	-3.00(-3.46, -2.53)	-2.87(-3.29, -2.44)	2.59(1.77, 3.41)
Mesothelioma	3, 140(2, 583-3, 889)	13, 947(9, 211-18, 070)	3.34(3.01, 3.67)	2.38(1.95, 2.81)	3.13(2.38, 3.88)	4.49(4.34, 4.64)
Motor neuron disease	3, 221(1, 817-4, 225)	56, 790(34, 864-75, 969)	4.71(4.08, 5.35)	5.86(5.36, 6.35)	0.38(-1.37, 2.16)	9.19(8.92, 9.46)
Multiple myeloma	6, 353(4, 187-13, 061)	1, 004(711-1, 381)	7.78(6.69, 8.87)	14.39(11.07, 17.82)	2.39(1.50, 3.29)	7.80(7.45, 8.15)
Multiple sclerosis	208(140-295)	112, 141(87, 784-140, 644)	5.20(4.98, 5.42)	4.22(3.86, 4.59)	3.47(3.22, 3.73)	8.31(7.80, 8.83)
Nasopharynx cancer	100, 440(83, 267-117, 554)	428, 426(227, 601-681, 013)	0.29(0.01, 0.57)	0.11(-0.21, 0.43)	-2.59(-3.08, -2.11)	4.27(3.76, 4.78)
Neck pain	134, 923(70, 987-217, 247)	61, 777(45, 365-78, 305)	3.87(3.57, 4.17)	2.69(2.45, 2.92)	3.01(2.16, 3.86)	6.07(5.85, 6.29)
Neonatal disorders	10, 714(7, 451-15, 701)	2, 904(1, 938-3, 760)	5.92(5.73, 6.11)	4.59(4.45, 4.73)	5.04(4.91, 5.17)	8.21(7.68, 8.74)
Neuroblastoma and other peripheral nervous cell tumors	274(190-404)	165, 808(126, 399-203, 441)	8.06(7.77, 8.36)	5.52(5.26, 5.79)	9.28(8.65, 9.92)	9.09(8.78, 9.41)
Non-Hodgkin lymphoma	65, 057(55, 520-81, 023)	58, 582(45, 112-74, 145)	3.06(2.80, 3.33)	1.94(1.67, 2.21)	2.15(1.49, 2.81)	4.87(4.60, 5.14)
Non-melanoma skin cancer	19, 920(15, 888-25, 769)	11, 578(8, 380-16, 254)	3.58(3.04, 4.11)	2.64(2.38, 2.90)	3.66(2.13, 5.21)	4.25(3.90, 4.60)
Non-rheumatic valvular heart disease	6, 230(4, 574-8, 207)	541, 964(330, 153-823, 502)	2.07(1.80, 2.34)	2.09(1.91, 2.26)	-0.54(-1.19, 0.11)	5.04(4.77, 5.31)
Oral disorders	212, 730(125, 539-330, 584)	825, 403(394, 610-1, 674, 987)	3.07(2.66, 3.49)	0.00(-0.39, 0.39)	2.48(2.28, 2.68)	7.43(6.05, 8.82)
Osteoarthritis	249, 042(119, 181-499, 690)	72, 629(44, 773-114, 344)	4.05(3.75, 4.34)	2.40(2.02, 2.78)	2.78(2.16, 3.40)	7.01(6.79, 7.23)
Other cardiovascular and circulatory diseases	29, 479(20, 291-42, 055)	11, 022(8, 274-13, 957)	3.02(2.79, 3.25)	2.27(2.06, 2.47)	1.33(0.70, 1.97)	5.58(5.39, 5.77)
Other chronic respiratory diseases	3, 092(2, 322-5, 041)	32, 641(26, 092-40, 673)	4.32(4.19, 4.46)	4.72(4.62, 4.82)	4.72(4.62, 4.82)	3.34(2.96, 3.72)
Other digestive diseases	40, 938(28, 441-50, 359)	154(8-408)	-0.79(-0.98, -0.59)	-1.90(-2.36, -1.43)	-3.24(-3.51, -2.96)	3.43(3.26, 3.60)

Other intestinal infectious diseases	183(8-478)	143, 525(108, 520-184, 440)	-0.60(-0.92, -0.28)	-1.32(-1.65, -0.98)	-2.44(-3.23, -1.64)	1.93(1.71, 2.15)
Other malignant neoplasms	85, 993(58, 334-108, 523)	149, 429(96, 665-224, 088)	1.62(1.47, 1.78)	-0.06(-0.22, 0.11)	0.17(-0.18, 0.52)	4.91(4.67, 5.16)
Other mental disorders	53, 194(34, 087-79, 330)	708, 120(433, 588-1, 123, 547)	3.39(3.23, 3.55)	2.59(2.38, 2.79)	1.79(1.54, 2.04)	5.88(5.50, 6.26)
Other musculoskeletal disorders	211, 919(128, 428-338, 005)	5, 523(4, 189-7, 382)	3.99(3.85, 4.13)	3.21(3.00, 3.43)	2.66(2.34, 2.97)	6.20(6.03, 6.38)
Other neglected tropical diseases	4, 294(3, 194-5, 683)	26, 812(17, 899-42, 196)	0.76(0.45, 1.07)	0.06(-0.31, 0.42)	-1.13(-1.70, -0.56)	3.08(2.81, 3.35)
Other neoplasms	6, 280(4, 117-10, 463)	47, 625(37, 250-59, 459)	4.81(4.45, 5.16)	3.72(3.49, 3.95)	4.73(3.76, 5.70)	6.05(5.59, 6.51)
Other neurological disorders	5, 898(4, 239-7, 878)	3, 347(2, 526-4, 356)	7.02(6.76, 7.29)	7.48(7.10, 7.85)	4.68(4.22, 5.14)	8.78(8.24, 9.32)
Other nutritional deficiencies	2, 507(1, 956-3, 288)	22, 663(17, 362-29, 448)	1.18(0.17, 2.20)	-2.67(-3.70, -1.62)	2.49(0.03, 5.02)	2.83(2.24, 3.42)
Other pharynx cancer	15, 484(12, 411-19, 518)	124, 856(60, 289-234, 720)	1.17(0.82, 1.52)	0.54(-0.31, 1.41)	-1.48(-1.98, -0.98)	5.27(5.03, 5.51)
Other sense organ diseases	42, 773(20, 345-80, 335)	76, 547(38, 731-138, 301)	3.51(3.34, 3.67)	2.63(2.44, 2.82)	1.99(1.76, 2.22)	5.99(5.56, 6.41)
Other skin and subcutaneous diseases	20, 563(10, 282-36, 872)	25, 317(20, 692-30, 606)	4.35(4.20, 4.50)	3.33(3.17, 3.49)	3.09(2.83, 3.35)	6.74(6.39, 7.10)
Other transport injuries	17, 868(14, 800-21, 149)	51, 594(37, 321-70, 199)	1.28(0.80, 1.76)	1.95(1.61, 2.28)	-0.56(-1.72, 0.62)	2.59(1.85, 3.33)
Other unintentional injuries	38, 981(29, 084-50, 548)	8, 569(5, 842-12, 486)	0.83(0.51, 1.14)	0.24(-0.25, 0.73)	-0.81(-1.45, -0.16)	2.97(2.65, 3.29)
Other unspecified infectious diseases	8, 502(4, 809-11, 369)	11, 346(5, 759-19, 787)	0.06(-0.28, 0.40)	-0.70(-1.32, -0.07)	-3.15(-3.83, -2.47)	4.33(4.01, 4.66)
Otitis media	6, 322(3, 360-11, 337)	98, 762(70, 475-128, 966)	1.91(1.80, 2.03)	1.15(0.97, 1.34)	0.23(-0.02, 0.47)	4.49(4.33, 4.65)
Ovarian cancer	35, 867(28, 319-45, 382)	486, 491(377, 765-607, 451)	3.38(3.05, 3.71)	3.34(2.88, 3.80)	0.26(-0.32, 0.84)	7.61(7.09, 8.14)
Pancreatic cancer	142, 793(120, 156-166, 703)	37, 927(29, 477-51, 185)	4.13(3.76, 4.50)	3.39(3.10, 3.67)	2.69(1.75, 3.63)	6.50(6.03, 6.97)
Pancreatitis	21, 811(16, 294-28, 425)	31, 298(24, 312-45, 496)	1.82(1.52, 2.13)	1.17(1.03, 1.31)	0.62(-0.25, 1.49)	3.93(3.69, 4.16)
Paralytic ileus and intestinal obstruction	29, 036(23, 231-36, 821)	276, 363(224, 584-330, 790)	0.24(0.02, 0.47)	0.23(0.09, 0.36)	-1.71(-2.31, -1.12)	2.55(2.30, 2.80)
Parkinson's disease	93, 408(78, 853-107, 378)	28, 064(20, 984-37, 072)	3.60(3.35, 3.86)	3.58(3.41, 3.75)	1.46(0.72, 2.20)	5.83(5.64, 6.01)
Pneumoconiosis	25, 481(19, 979-31, 775)	51, 127(26, 921-66, 822)	0.34(-0.13, 0.81)	-0.05(-0.20, 0.11)	-1.68(-3.06, -0.28)	2.84(2.61, 3.07)
Poisonings	20, 979(15, 448-37, 912)	4, 400(1, 310-7, 668)	2.99(2.37, 3.62)	2.14(1.10, 3.20)	4.55(3.87, 5.23)	1.56(0.74, 2.39)

Police conflict and executions	561(124-957)	101, 959(71, 029-141, 016)	6.79(6.64, 6.95)	4.95(4.75, 5.14)	5.46(5.29, 5.63)	10.23(9.94, 10.52)
Prostate cancer	36, 927(26, 985-48, 244)	8, 542(6, 795-10, 749)	3.36(3.04, 3.69)	3.89(3.50, 4.29)	0.66(-0.02, 1.34)	5.77(5.23, 6.32)
Protein-energy malnutrition	17, 576(14, 831-20, 375)	18, 851(8, 384-39, 183)	-2.42(-2.92, -1.92)	-5.63(-6.27, -4.99)	-4.08(-4.55, -3.61)	3.02(1.86, 4.20)
Pruritus	5, 747(2, 573-11, 946)	62, 799(44, 965-81, 957)	3.95(3.79, 4.11)	2.86(2.64, 3.07)	2.66(2.29, 3.03)	6.49(6.33, 6.65)
Psoriasis	16, 793(11, 832-21, 923)	16, 458(10, 976-21, 324)	4.39(4.22, 4.56)	4.00(3.77, 4.24)	2.60(2.32, 2.87)	6.59(6.20, 6.99)
Pulmonary Arterial Hypertension	8, 928(6, 668-12, 134)	1, 357(573-2, 248)	2.02(1.83, 2.21)	0.31(0.17, 0.46)	4.49(3.97, 5.01)	1.48(1.26, 1.70)
Rabies	923(431-1, 459)	233, 871(183, 147-295, 922)	0.85(-0.23, 1.94)	-2.09(-4.14, 0.00)	7.74(6.59, 8.91)	-4.56(-6.42, -2.67)
Rheumatic heart disease	390, 903(315, 669-463, 233)	102, 806(77, 867-132, 587)	-1.67(-2.23, -1.10)	-2.20(-2.41, -1.98)	-3.59(-5.12, -2.03)	1.21(0.65, 1.77)
Rheumatoid arthritis	38, 885(30, 548-50, 132)	709, 034(601, 100-829, 098)	3.24(3.01, 3.47)	2.30(1.95, 2.65)	2.04(1.60, 2.47)	5.27(5.11, 5.43)
Road injuries	284, 046(244, 583-326, 362)	61, 501(31, 370-108, 445)	3.15(2.44, 3.88)	3.39(2.57, 4.21)	2.75(0.84, 4.69)	3.08(2.80, 3.36)
Scabies	21, 925(11, 041-38, 804)	4, 498(2, 658-7, 801)	3.39(3.25, 3.54)	2.56(2.37, 2.75)	1.76(1.53, 1.99)	5.95(5.60, 6.30)
Schistosomiasis	8, 681(6, 453-11, 658)	157, 654(116, 266-201, 714)	-2.12(-2.77, -1.48)	-4.47(-4.68, -4.25)	-3.95(-5.74, -2.12)	2.73(2.16, 3.30)
Schizophrenia	54, 437(39, 857-68, 036)	327, 275(267, 232-405, 199)	3.51(3.27, 3.76)	2.50(2.31, 2.70)	1.71(1.01, 2.41)	6.50(6.31, 6.69)
Self-harm	324, 395(228, 197-376, 097)	6, 607(3, 512-11, 900)	-0.08(-0.50, 0.35)	0.61(0.43, 0.79)	-3.18(-4.32, -2.03)	2.71(2.31, 3.11)
Sexually transmitted infections excluding HIV	3, 746(2, 336-5, 813)	15, 394(10, 335-22, 213)	1.80(1.59, 2.01)	-0.38(-0.62, -0.14)	0.74(0.54, 0.94)	5.35(4.91, 5.79)
Soft tissue and other extraosseous sarcomas	10, 149(6, 666-13, 104)	1, 731, 067(1, 319, 131-2, 181, 334)	1.38(1.00, 1.75)	1.27(1.11, 1.44)	-0.81(-1.84, 0.23)	3.93(3.61, 4.24)
Stomach cancer	1, 435, 081(1, 187, 914-1, 708, 500)	7, 525, 691(6, 310, 752-8, 878, 795)	0.53(0.15, 0.91)	0.48(0.15, 0.82)	-1.12(-2.15, -0.07)	2.08(1.91, 2.25)
Stroke	5, 299, 303(4, 633, 281-6, 030, 801)	3, 122(2, 305-4, 274)	1.20(0.75, 1.65)	1.45(1.25, 1.65)	-0.86(-2.09, 0.38)	3.15(2.77, 3.53)
Testicular cancer	1, 441(1, 115-1, 836)	671(200-1, 621)	2.60(2.17, 3.03)	2.20(1.52, 2.89)	-0.43(-0.90, 0.04)	6.86(6.02, 7.70)
Tetanus	7, 221(3, 213-10, 627)	23, 124(18, 325-28, 914)	-7.35(-8.37, -6.33)	-8.10(-10.64, -5.50)	-8.48(-9.17, -7.78)	-4.98(-6.88, -3.04)
Thyroid cancer	11, 070(8, 840-13, 424)	3, 388, 113(2, 695, 936-4, 147, 382)	2.40(2.11, 2.69)	1.51(1.31, 1.71)	0.56(-0.16, 1.29)	5.46(5.09, 5.83)
Tracheal, bronchus, and lung cancer	1, 155, 073(974, 154-1, 348, 655)	282(101-591)	3.56(3.23, 3.88)	3.41(3.05, 3.76)	2.06(1.20, 2.94)	5.17(5.05, 5.29)

Trachoma	960(387-1, 924)	178, 240(138, 826-225, 989)	-3.84(-4.35, -3.33)	-1.16(-2.13, -0.18)	-8.21(-9.24, -7.16)	-2.73(-3.13, -2.32)
Tuberculosis	507, 033(418, 432-616, 201)	316(134-597)	-3.40(-3.73, -3.08)	-3.70(-4.06, -3.33)	-6.47(-7.17, -5.78)	-0.13(-0.55, 0.30)
Typhoid and paratyphoid	363(143-733)	269, 054(210, 487-350, 828)	-0.38(-0.59, -0.17)	-1.72(-2.17, -1.27)	-1.27(-1.66, -0.88)	1.98(1.83, 2.12)
Upper digestive system diseases	283, 658(241, 327-331, 736)	25, 073(14, 903-40, 386)	-0.18(-0.39, 0.03)	-1.20(-1.46, -0.94)	-1.78(-2.25, -1.30)	2.33(2.15, 2.51)
Upper respiratory infections	26, 632(10, 959-38, 792)	140, 781(93, 019-209, 080)	-0.21(-0.44, 0.02)	-1.19(-1.37, -1.00)	-3.57(-4.19, -2.96)	4.87(4.65, 5.09)
Urinary diseases and male infertility	78, 422(55, 664-110, 228)	27, 879(16, 620-42, 686)	1.94(1.71, 2.17)	0.79(0.32, 1.25)	0.15(-0.26, 0.56)	4.90(4.68, 5.13)
Urticaria	9, 914(5, 963-15, 310)	58, 331(42, 235-79, 317)	3.44(3.11, 3.78)	2.48(2.27, 2.68)	1.95(0.96, 2.95)	6.05(5.87, 6.23)
Uterine cancer	36, 189(26, 362-46, 233)	7, 075(4, 046-11, 041)	1.65(1.19, 2.11)	2.06(1.84, 2.27)	-0.94(-2.18, 0.32)	4.33(3.83, 4.83)
Varicella and herpes zoster	5, 079(3, 767-6, 650)	2, 104(1, 696-2, 612)	1.12(0.87, 1.38)	-0.25(-0.59, 0.10)	-1.90(-2.43, -1.36)	5.71(5.45, 5.96)
Vascular intestinal disorders	1, 828(1, 436-2, 180)	36, 529(22, 999-54, 211)	0.48(0.07, 0.88)	1.63(1.42, 1.84)	-3.40(-4.34, -2.45)	3.73(3.25, 4.21)
Viral skin diseases	12, 935(8, 238-19, 223)	1, 280(630-2, 151)	3.41(3.25, 3.56)	2.65(2.45, 2.85)	1.80(1.56, 2.05)	5.85(5.48, 6.23)
Vitamin A deficiency	458(237-780)	1, 280(630, 2, 151)	3.57(3.03, 4.11)	3.34(2.04, 4.65)	1.85(1.10, 2.61)	5.41(5.03, 5.79)
**70-74 years**						
Acne vulgaris	962(613-1, 478)	3, 257(2, 039-5, 035)	4.00(3.91, 4.10)	3.78(3.69, 3.88)	3.11(3.00, 3.23)	5.45(5.24, 5.65)
Acute glomerulonephritis	16, 354(11, 861-22, 050)	14, 396(9, 409-19, 603)	-0.41(-0.64, -0.17)	-1.11(-1.33, -0.89)	-0.29(-0.94, 0.36)	0.06(-0.05, 0.17)
Acute hepatitis	40, 173(31, 799-50, 391)	8, 339(6, 506-10, 818)	-4.99(-5.42, -4.56)	-5.47(-5.69, -5.25)	-9.73(-10.90, -8.54)	0.55(0.10, 1.00)
Adverse effects of medical treatment	11, 734(7, 807-14, 931)	8, 520(6, 684-11, 828)	-1.09(-1.44, -0.74)	-1.26(-1.44, -1.08)	-4.07(-4.76, -3.37)	2.62(2.04, 3.20)
Age-related and other hearing loss	494, 610(324, 725-722, 049)	1, 579, 818(1, 059, 064-2, 205, 744)	3.86(3.69, 4.03)	3.15(2.90, 3.39)	3.01(2.69, 3.34)	5.79(5.57, 6.02)
Alcohol use disorders	20, 986(15, 764-28, 448)	65, 165(45, 826-89, 973)	3.69(3.29, 4.10)	3.58(2.40, 4.77)	4.81(4.53, 5.08)	2.35(2.02, 2.69)
Alopecia areata	1, 290(841-1, 917)	3, 597(2, 375-5, 213)	3.35(3.15, 3.56)	3.00(2.92, 3.09)	2.40(2.23, 2.58)	5.07(4.41, 5.72)
Alzheimer's disease and other dementias	421, 670(196, 161-935, 659)	1, 294, 058(655, 354-2, 769, 215)	3.58(3.33, 3.83)	3.22(3.06, 3.38)	2.31(2.03, 2.60)	5.70(4.99, 6.41)
Animal contact	8, 999(6, 919-10, 434)	6, 270(5, 071-7, 735)	-1.20(-1.42, -0.99)	-0.28(-0.70, 0.15)	-4.44(-4.68, -4.19)	1.90(1.52, 2.28)

Anxiety disorders	96, 845(64, 923-138, 024)	276, 604(186, 638-391, 068)	3.37(3.09, 3.65)	3.66(3.28, 4.04)	1.28(0.94, 1.62)	6.17(5.60, 6.74)
Aortic aneurysm	6, 206(4, 941-7, 877)	23, 227(18, 229-29, 524)	4.30(4.11, 4.48)	3.66(3.51, 3.82)	4.35(3.95, 4.76)	5.34(5.06, 5.62)
Appendicitis	8, 281(5, 963-10, 134)	4, 645(3, 599-6, 048)	-1.95(-2.15, -1.74)	-0.95(-1.29, -0.61)	-4.51(-4.80, -4.23)	0.14(-0.25, 0.53)
Asthma	154, 714(121, 208-206, 043)	107, 206(83, 821-136, 128)	-1.23(-1.44, -1.03)	-0.48(-0.59, -0.36)	-3.69(-4.10, -3.28)	0.86(0.47, 1.25)
Atrial fibrillation and flutter	75, 478(56, 393-99, 196)	214, 338(160, 021-281, 169)	3.42(3.25, 3.59)	2.60(2.37, 2.82)	2.50(2.18, 2.83)	5.52(5.29, 5.75)
Attention-deficit/hyperactivity disorder	140(47-303)	348(107-761)	2.98(2.81, 3.16)	2.08(1.70, 2.46)	2.24(2.06, 2.42)	5.01(4.79, 5.24)
Autism spectrum disorders	13, 611(9, 316-19, 210)	47, 140(31, 904-65, 716)	4.13(4.02, 4.23)	4.08(3.99, 4.18)	3.31(3.19, 3.44)	5.31(5.09, 5.53)
Bacterial skin diseases	10, 343(7, 938-12, 414)	5, 674(4, 264-8, 148)	-1.90(-2.39, -1.41)	-0.06(-0.36, 0.24)	-9.16(-10.43, -7.87)	5.49(4.86, 6.13)
Bipolar disorder	8, 124(5, 043-12, 022)	23, 140(14, 799-34, 796)	3.41(3.22, 3.61)	3.12(3.04, 3.21)	2.35(1.95, 2.75)	5.08(4.69, 5.46)
Bladder cancer	81, 550(60, 370-95, 837)	155, 097(121, 167-201, 098)	2.10(1.83, 2.37)	2.40(2.21, 2.59)	-0.23(-0.40, -0.05)	4.65(3.76, 5.55)
Blindness and vision loss	283, 767(195, 692-397, 467)	734, 163(474, 744-1, 124, 801)	3.20(2.81, 3.60)	4.60(3.49, 5.71)	1.46(1.26, 1.67)	4.00(3.49, 4.52)
Brain and central nervous system cancer	55, 441(43, 336-70, 731)	173, 932(130, 165-224, 046)	3.77(3.59, 3.95)	4.26(3.81, 4.72)	2.26(2.11, 2.41)	5.16(4.95, 5.36)
Breast cancer	67, 775(56, 501-80, 467)	205, 887(166, 515-250, 580)	3.67(3.52, 3.82)	3.48(3.25, 3.71)	2.42(2.27, 2.57)	5.74(5.44, 6.04)
Cardiomyopathy and myocarditis	18, 621(15, 132-29, 609)	57, 158(45, 423-72, 660)	3.67(3.18, 4.16)	6.49(5.10, 7.90)	2.26(2.03, 2.49)	2.28(1.76, 2.80)
Cervical cancer	59, 488(49, 201-73, 556)	114, 392(86, 220-146, 373)	2.17(1.90, 2.44)	0.14(-0.15, 0.43)	1.26(0.68, 1.85)	5.47(5.18, 5.76)
Chronic kidney disease	335, 057(287, 243-399, 442)	786, 672(645, 700-932, 282)	2.83(2.65, 3.01)	2.46(2.17, 2.76)	2.10(1.87, 2.33)	4.01(3.75, 4.28)
Chronic obstructive pulmonary disease	4, 954, 229(4, 266, 084-5, 557, 102)	3, 976, 193(3, 291, 563-4, 791, 778)	-0.79(-1.02, -0.57)	0.07(-0.36, 0.50)	-3.40(-3.64, -3.16)	1.58(1.16, 2.00)
Cirrhosis and other chronic liver diseases	341, 679(292, 162-399, 302)	382, 863(305, 170-470, 237)	0.32(0.12, 0.51)	0.63(0.39, 0.88)	-1.19(-1.48, -0.90)	1.78(1.45, 2.11)
Colon and rectum cancer	326, 171(280, 853-373, 955)	911, 386(726, 940-1, 106, 812)	3.41(3.26, 3.56)	3.43(3.15, 3.72)	1.94(1.78, 2.11)	5.22(5.00, 5.45)
Conflict and terrorism	29(19-43)	73(54-100)	3.34(-9.15, 17.54)	19.48(9.32, 30.59)	-3.10(-33.17, 40.50)	-7.94(-16.71, 1.76)
Congenital birth defects	5, 565(3, 710-8, 022)	15, 443(10, 290-22, 173)	3.15(2.82, 3.49)	4.28(4.07, 4.49)	1.54(1.06, 2.03)	4.08(3.18, 4.98)
COVID-19	NA	2, 272(286-7, 816)	NA	NA	NA	NA

Cystic echinococcosis	513(338-701)	527(313-868)	0.09(-0.12, 0.30)	-2.06(-2.27, -1.85)	-0.07(-0.49, 0.35)	2.42(2.15, 2.70)
Cysticercosis	12, 675(5, 339-22, 161)	17, 662(7, 164-35, 204)	1.04(0.59, 1.48)	1.14(0.85, 1.44)	-1.40(-2.17, -0.62)	3.85(2.74, 4.97)
Decubitus ulcer	1, 003(598-2, 192)	6, 102(3, 307-7, 895)	6.89(5.93, 7.85)	13.10(11.88, 14.33)	4.77(3.06, 6.51)	3.13(1.47, 4.82)
Dengue	27(12-43)	26(12-44)	-0.48(-1.61, 0.66)	-6.35(-7.91, -4.75)	2.02(0.51, 3.56)	2.51(0.69, 4.35)
Depressive disorders	154, 429(106, 922-207, 632)	523, 222(363, 316-711, 483)	4.03(3.83, 4.23)	4.75(4.28, 5.21)	2.86(2.66, 3.06)	4.83(4.58, 5.08)
Dermatitis	18, 843(10, 747-32, 598)	53, 255(30, 436-91, 893)	3.38(3.17, 3.60)	3.11(3.03, 3.19)	2.41(2.17, 2.66)	5.04(4.39, 5.69)
Diabetes mellitus	428, 822(364, 065-516, 641)	1, 373, 154(1, 119, 636-1, 681, 591)	3.77(3.64, 3.89)	4.90(4.74, 5.07)	1.94(1.77, 2.11)	4.79(4.54, 5.04)
Diarrheal diseases	41, 593(20, 475-62, 020)	11, 022(7, 602-17, 819)	-4.29(-4.68, -3.91)	-5.54(-5.77, -5.31)	-6.73(-7.00, -6.45)	0.12(-0.94, 1.18)
Dietary iron deficiency	76, 217(51, 640-108, 550)	83, 422(55, 089-120, 383)	0.27(0.05, 0.49)	0.81(0.60, 1.03)	-1.81(-1.94, -1.69)	2.49(1.78, 3.19)
Drowning	45, 895(39, 935-52, 415)	79, 391(65, 679-93, 595)	1.84(1.63, 2.04)	1.29(1.02, 1.56)	0.82(0.45, 1.19)	3.52(3.27, 3.78)
Drug use disorders	29, 548(23, 894-36, 262)	26, 238(19, 990-34, 290)	-0.53(-0.86, -0.20)	0.23(-0.18, 0.64)	-4.13(-4.57, -3.70)	3.61(2.94, 4.27)
Encephalitis	3, 083(2, 540-3, 716)	5, 914(4, 818-7, 031)	2.09(1.89, 2.28)	2.36(2.02, 2.71)	1.04(0.81, 1.27)	3.07(2.74, 3.40)
Endocarditis	4, 030(2, 146-5, 273)	3, 723(2, 835-5, 575)	-0.27(-0.55, 0.02)	1.53(1.20, 1.86)	-4.47(-4.92, -4.01)	3.55(3.03, 4.06)
Endocrine, metabolic, blood, and immune disorders	62, 924(40, 816-92, 192)	166, 152(105, 856-247, 543)	3.30(2.48, 4.13)	4.37(3.47, 5.29)	1.67(-0.59, 3.97)	3.78(3.38, 4.18)
Environmental heat and cold exposure	10, 662(5, 043-13, 756)	10, 828(5, 732-15, 024)	0.04(-0.24, 0.32)	-0.43(-0.76, -0.09)	-1.97(-2.29, -1.65)	3.20(2.70, 3.70)
Esophageal cancer	670, 812(544, 255-777, 593)	1, 068, 588(853, 276-1, 312, 455)	1.52(1.34, 1.71)	2.32(1.99, 2.65)	-0.81(-1.04, -0.59)	3.56(3.26, 3.86)
Exposure to forces of nature	1, 754(1, 580-1, 933)	2, 202(1, 806-2, 717)	-2.99(-18.76, 15.84)	-6.78(-13.28, 0.22)	4.07(-38.69, 76.62)	-5.52(-16.20, 6.52)
Exposure to mechanical forces	18, 092(14, 152-24, 339)	53, 385(38, 931-71, 271)	3.75(3.37, 4.14)	3.20(2.52, 3.89)	4.62(3.99, 5.26)	3.12(2.90, 3.35)
Eye cancer	935(581-1, 221)	1, 910(1, 073-2, 580)	2.34(2.22, 2.47)	2.35(2.12, 2.59)	1.31(1.17, 1.44)	3.67(3.47, 3.87)
Falls	211, 486(169, 474-260, 407)	621, 443(462, 176-788, 462)	3.56(3.35, 3.78)	3.21(2.69, 3.73)	0.76(0.53, 1.00)	7.44(7.20, 7.68)
Fire, heat, and hot substances	24, 743(19, 501-29, 281)	32, 649(24, 809-41, 747)	0.77(0.34, 1.20)	-0.17(-0.42, 0.09)	0.54(-0.66, 1.75)	2.40(1.99, 2.81)
Food-borne trematodiases	28, 064(11, 468-56, 576)	39, 708(19, 789-71, 793)	0.99(-0.12, 2.11)	-0.65(-2.19, 0.92)	0.66(0.27, 1.06)	2.48(0.14, 4.88)

Foreign body	10, 760(8, 403-13, 370)	26, 768(17, 799-33, 624)	3.04(2.85, 3.22)	2.78(2.52, 3.04)	1.06(0.74, 1.39)	5.98(5.68, 6.28)
Fungal skin diseases	8, 213(3, 446-17, 516)	22, 910(9, 565-48, 521)	3.34(3.13, 3.55)	3.08(3.00, 3.17)	2.38(2.14, 2.62)	4.95(4.31, 5.60)
Gallbladder and biliary diseases	109, 338(74, 155-143, 779)	210, 152(140, 681-294, 692)	2.12(1.83, 2.41)	1.58(1.50, 1.66)	1.40(1.30, 1.50)	3.87(2.93, 4.81)
Gallbladder and biliary tract cancer	56, 707(44, 287-73, 760)	130, 459(92, 069-174, 039)	2.75(2.60, 2.89)	2.54(2.33, 2.76)	1.65(1.43, 1.88)	4.33(4.11, 4.56)
Gout	14, 578(8, 224-23, 090)	55, 456(32, 100-87, 311)	4.48(4.31, 4.66)	2.54(2.26, 2.82)	5.07(4.75, 5.40)	5.96(5.70, 6.22)
Gynecological diseases	31, 248(20, 994-46, 428)	59, 070(39, 355-85, 831)	2.07(1.90, 2.23)	2.67(2.60, 2.75)	-1.33(-1.68, -0.97)	5.11(4.83, 5.39)
Headache disorders	72, 707(21, 666-151, 734)	216, 936(60, 931-453, 001)	3.58(3.43, 3.74)	3.14(2.95, 3.32)	2.98(2.61, 3.35)	5.00(4.79, 5.22)
Hemoglobinopathies and hemolytic anemias	43, 358(32, 517-57, 332)	57, 687(44, 696-74, 037)	0.91(0.73, 1.09)	0.85(0.76, 0.94)	-0.36(-0.50, -0.22)	2.64(2.07, 3.22)
HIV/AIDS	1, 088(173-1, 739)	41, 713(28, 661-58, 599)	11.67(10.59, 12.76)	19.81(18.41, 21.22)	3.41(2.24, 4.60)	13.34(10.52, 16.24)
Hodgkin lymphoma	8, 163(3, 368-11, 241)	7, 091(4, 270-9, 422)	-0.45(-0.64, -0.27)	-0.15(-0.31, 0.01)	-3.51(-3.76, -3.26)	2.75(2.33, 3.16)
Hypertensive heart disease	860, 496(555, 916-1, 025, 259)	887, 676(638, 745-1, 138, 466)	0.02(-0.15, 0.20)	-1.62(-1.83, -1.42)	-1.44(-1.73, -1.14)	3.90(3.61, 4.19)
Idiopathic developmental intellectual disability	1, 382(422-2, 756)	2, 632(686-5, 734)	2.05(1.82, 2.29)	3.40(2.82, 3.99)	0.01(-0.17, 0.19)	3.12(2.77, 3.46)
Idiopathic epilepsy	23, 689(16, 108-34, 065)	50, 944(32, 593-78, 611)	2.51(2.18, 2.84)	3.04(2.76, 3.32)	0.03(-0.36, 0.42)	5.05(4.09, 6.03)
Inflammatory bowel disease	10, 481(6, 262-13, 632)	14, 570(10, 858-19, 452)	1.07(0.89, 1.25)	1.72(1.41, 2.03)	-0.69(-0.89, -0.49)	2.49(2.17, 2.80)
Inguinal, femoral, and abdominal hernia	5, 387(3, 884-7, 291)	9, 396(5, 989-14, 362)	1.76(1.59, 1.94)	1.59(1.37, 1.82)	1.04(0.79, 1.29)	2.75(2.44, 3.06)
Interpersonal violence	33, 256(27, 583-39, 754)	45, 390(36, 522-56, 089)	0.97(0.76, 1.19)	1.22(1.11, 1.33)	-0.58(-0.74, -0.42)	2.75(2.07, 3.42)
Interstitial lung disease and pulmonary sarcoidosis	12, 126(9, 356-19, 562)	34, 633(24, 006-46, 253)	3.48(3.30, 3.65)	2.91(2.74, 3.09)	4.31(3.95, 4.67)	3.51(3.19, 3.82)
Intestinal nematode infections	12, 322(6, 471-19, 553)	704(357-1, 192)	-9.01(-9.51, -8.50)	-9.66(-10.11, -9.20)	-13.55(-14.35, -12.74)	-1.29(-2.63, 0.08)
Invasive Non-typhoidal Salmonella (iNTS)	605(162-1, 353)	963(261-2, 212)	1.53(1.38, 1.69)	1.32(1.23, 1.40)	0.37(0.24, 0.49)	3.31(2.82, 3.81)
Iodine deficiency	3, 370(1, 807-5, 937)	9, 725(4, 541-18, 828)	3.48(3.31, 3.66)	2.55(2.30, 2.80)	4.38(4.04, 4.71)	3.13(2.87, 3.39)
Ischemic heart disease	1, 622, 342(1, 412, 499-1, 829, 893)	4, 868, 617(4, 043, 859-5, 728, 020)	3.62(3.45, 3.79)	3.90(3.60, 4.19)	3.20(3.00, 3.40)	3.59(3.33, 3.84)
Kidney cancer	19, 727(16, 952-22, 191)	71, 951(58, 086-86, 713)	4.23(3.99, 4.47)	4.15(3.64, 4.67)	3.23(2.99, 3.48)	5.51(5.17, 5.85)

Larynx cancer	40, 904(33, 062-48, 078)	71, 635(56, 018-90, 944)	1.85(1.54, 2.16)	1.42(1.28, 1.57)	0.65(-0.20, 1.51)	3.84(3.53, 4.16)
Leishmaniasis	176(11-521)	124(21-313)	-1.36(-2.07, -0.63)	-3.68(-4.44, -2.91)	2.92(1.03, 4.83)	-3.24(-3.76, -2.71)
Leprosy	46(28-71)	36(20-57)	-0.69(-1.00, -0.38)	-0.53(-0.75, -0.32)	-2.90(-3.11, -2.69)	1.89(0.87, 2.91)
Leukemia	62, 752(51, 358-78, 473)	136, 645(99, 838-173, 550)	2.56(2.41, 2.71)	2.65(2.30, 2.99)	1.16(1.01, 1.31)	4.23(4.03, 4.44)
Lip and oral cavity cancer	26, 799(22, 562-31, 224)	76, 989(59, 941-96, 324)	3.49(3.34, 3.64)	2.58(2.50, 2.67)	3.47(3.26, 3.67)	4.54(4.13, 4.95)
Liver cancer	191, 789(162, 873-223, 647)	430, 680(346, 764-530, 674)	2.71(2.27, 3.15)	4.42(4.09, 4.76)	-0.31(-1.52, 0.92)	4.32(3.95, 4.68)
Low back pain	400, 802(253, 563-594, 266)	864, 659(547, 654-1, 277, 692)	2.50(2.34, 2.65)	1.27(0.91, 1.64)	2.15(2.02, 2.28)	4.37(4.15, 4.59)
Lower extremity peripheral arterial disease	13, 602(6, 852-25, 981)	34, 915(18, 730-65, 040)	3.06(2.93, 3.19)	3.20(2.94, 3.46)	1.89(1.75, 2.03)	4.48(4.27, 4.69)
Lower respiratory infections	383, 126(306, 450-436, 857)	349, 191(283, 034-436, 442)	-0.47(-0.71, -0.23)	0.24(-0.16, 0.63)	-2.76(-3.04, -2.48)	1.86(1.28, 2.44)
Malaria	561(43-5, 830)	58, 011(35, 003-78, 399)	-21.37(-26.66, -15.70)	-17.91(-21.24, -14.43)	-16.60(-20.77, -12.21)	-29.94(-40.87, -16.98)
Malignant neoplasm of bone and articular cartilage	11, 980(7, 586-21, 487)	12, 897(6, 834-17, 088)	5.48(5.15, 5.81)	7.50(6.84, 8.15)	5.15(4.71, 5.58)	3.44(3.05, 3.83)
Malignant skin melanoma	4, 762(3, 236-6, 783)	0(0-0)	3.29(3.17, 3.42)	3.28(3.02, 3.54)	2.20(2.09, 2.31)	4.79(4.52, 5.07)
Meningitis	14, 488(12, 511-16, 691)	10, 456(8, 547-12, 558)	-1.20(-1.71, -0.69)	-2.75(-3.43, -2.07)	-1.89(-2.92, -0.86)	1.99(1.68, 2.30)
Mesothelioma	2, 428(1, 991-2, 927)	7, 058(5, 583-8, 636)	3.49(3.36, 3.62)	3.03(2.83, 3.24)	4.21(4.06, 4.35)	3.49(3.22, 3.76)
Motor neuron disease	1, 765(1, 059-2, 292)	7, 956(5, 055-10, 614)	4.87(4.52, 5.22)	6.61(6.19, 7.04)	1.19(0.51, 1.87)	8.40(8.02, 8.79)
Multiple myeloma	5, 090(3, 411-10, 659)	48, 170(31, 605-63, 991)	7.66(6.80, 8.52)	13.49(10.79, 16.26)	3.33(3.11, 3.55)	7.23(6.75, 7.71)
Multiple sclerosis	131(89-183)	647(463-886)	5.34(5.07, 5.61)	5.41(5.27, 5.55)	4.13(3.91, 4.36)	7.08(6.29, 7.87)
Nasopharynx cancer	66, 467(56, 164-76, 770)	75, 302(60, 933-92, 431)	0.36(0.19, 0.52)	0.28(0.08, 0.48)	-2.00(-2.24, -1.75)	3.59(3.28, 3.91)
Neck pain	104, 295(52, 524-174, 299)	319, 311(163, 442-533, 015)	3.62(3.38, 3.87)	2.57(2.42, 2.73)	3.76(3.15, 4.37)	4.93(4.60, 5.27)
Neonatal disorders	4, 259(3, 019-6, 004)	24, 823(18, 070-31, 520)	5.92(5.69, 6.15)	4.65(4.22, 5.08)	5.63(5.30, 5.96)	7.77(7.49, 8.06)
Neuroblastoma and other peripheral nervous cell tumors	224(158-329)	2, 460(1, 680-3, 085)	8.12(7.89, 8.34)	6.25(5.98, 6.52)	9.65(9.36, 9.94)	8.46(7.88, 9.05)
Non-Hodgkin lymphoma	50, 556(43, 223-64, 548)	138, 342(108, 396-167, 893)	3.34(3.15, 3.53)	2.93(2.58, 3.27)	2.48(2.26, 2.69)	4.98(4.65, 5.30)

Non-melanoma skin cancer	17, 564(13, 997-22, 158)	55, 226(43, 132-68, 309)	3.75(3.52, 3.98)	3.27(2.83, 3.72)	4.45(4.27, 4.62)	3.68(3.16, 4.20)
Non-rheumatic valvular heart disease	7, 419(5, 560-10, 118)	15, 978(11, 435-22, 958)	2.49(2.37, 2.61)	2.91(2.79, 3.03)	0.96(0.79, 1.12)	4.02(3.78, 4.26)
Oral disorders	183, 058(113, 389-280, 256)	475, 027(297, 508-730, 695)	3.29(2.84, 3.74)	0.80(0.16, 1.45)	2.85(2.11, 3.61)	6.95(6.18, 7.73)
Osteoarthritis	185, 175(87, 239-377, 462)	615, 106(289, 194-1, 251, 167)	4.03(3.78, 4.27)	3.03(2.70, 3.37)	3.10(2.61, 3.59)	6.30(6.01, 6.60)
Other cardiovascular and circulatory diseases	31, 756(22, 668-44, 494)	75, 827(47, 674-118, 447)	2.86(2.71, 3.01)	3.03(2.71, 3.35)	1.61(1.44, 1.77)	4.36(4.03, 4.69)
Other chronic respiratory diseases	2, 924(2, 184-4, 810)	11, 106(8, 259-13, 832)	4.58(4.32, 4.84)	6.16(5.99, 6.34)	5.58(5.41, 5.75)	2.04(1.34, 2.74)
Other digestive diseases	42, 788(29, 763-52, 668)	35, 347(28, 648-43, 166)	-0.67(-0.83, -0.50)	-1.76(-2.01, -1.50)	-2.57(-2.75, -2.40)	3.11(2.78, 3.44)
Other intestinal infectious diseases	89(2-249)	80(2-220)	-0.34(-0.55, -0.14)	-0.48(-0.77, -0.19)	-1.98(-2.26, -1.70)	1.85(1.50, 2.20)
Other malignant neoplasms	68, 567(46, 325-85, 273)	123, 945(95, 818-156, 988)	1.90(1.74, 2.06)	0.82(0.63, 1.01)	1.02(0.71, 1.33)	4.38(4.13, 4.64)
Other mental disorders	36, 355(23, 082-54, 917)	103, 423(66, 740-154, 601)	3.42(3.32, 3.51)	3.20(3.11, 3.30)	2.54(2.43, 2.66)	4.83(4.62, 5.04)
Other musculoskeletal disorders	144, 458(84, 625-236, 088)	472, 676(280, 740-764, 824)	3.86(3.72, 3.99)	3.94(3.82, 4.06)	3.08(2.92, 3.23)	4.87(4.58, 5.15)
Other neglected tropical diseases	3, 504(2, 578-4, 687)	4, 496(3, 386-6, 036)	0.73(0.50, 0.96)	0.74(0.44, 1.04)	-0.47(-0.82, -0.12)	2.09(1.71, 2.47)
Other neoplasms	5, 321(3, 399-8, 699)	22, 878(15, 639-36, 478)	4.86(4.68, 5.05)	4.66(4.50, 4.82)	5.17(4.76, 5.59)	5.10(4.82, 5.38)
Other neurological disorders	4, 278(3, 075-5, 834)	39, 197(30, 582-49, 024)	7.47(7.22, 7.72)	8.86(8.25, 9.47)	5.03(4.76, 5.30)	9.16(8.93, 9.38)
Other nutritional deficiencies	2, 318(1, 875-2, 841)	4, 130(3, 110-5, 268)	1.97(1.66, 2.28)	-0.65(-1.08, -0.23)	3.50(2.94, 4.07)	2.19(1.83, 2.55)
Other pharynx cancer	11, 109(9, 198-13, 367)	18, 194(14, 200-22, 882)	1.55(1.34, 1.76)	0.88(0.57, 1.18)	-0.40(-0.69, -0.11)	5.09(4.74, 5.45)
Other sense organ diseases	36, 935(17, 736-65, 791)	108, 105(52, 719-191, 236)	3.51(3.28, 3.74)	3.25(3.16, 3.34)	2.59(2.34, 2.85)	5.07(4.39, 5.76)
Other skin and subcutaneous diseases	16, 591(8, 615-30, 292)	60, 584(30, 817-111, 702)	4.29(4.19, 4.39)	3.95(3.86, 4.04)	3.51(3.40, 3.63)	5.74(5.53, 5.94)
Other transport injuries	12, 475(10, 354-14, 718)	17, 931(14, 687-21, 837)	1.22(0.88, 1.56)	2.41(1.67, 3.16)	-0.77(-1.04, -0.51)	2.34(1.71, 2.96)
Other unintentional injuries	23, 455(18, 113-30, 219)	35, 171(25, 503-48, 045)	1.28(1.03, 1.53)	1.33(0.90, 1.76)	0.18(-0.11, 0.48)	2.60(2.16, 3.05)
Other unspecified infectious diseases	7, 421(4, 221-9, 859)	7, 779(5, 184-10, 721)	0.10(-0.11, 0.32)	0.12(-0.34, 0.59)	-3.00(-3.27, -2.72)	4.12(3.78, 4.46)
Otitis media	3, 883(1, 871-6, 977)	7, 275(3, 372-13, 079)	2.00(1.91, 2.10)	1.77(1.68, 1.85)	1.08(0.96, 1.19)	3.52(3.30, 3.74)

Ovarian cancer	23, 906(18, 872-30, 718)	65, 121(48, 477-83, 543)	3.30(3.11, 3.50)	3.55(3.23, 3.87)	1.07(0.85, 1.29)	6.14(5.88, 6.40)
Pancreatic cancer	111, 233(95, 301-130, 587)	412, 760(325, 561-505, 036)	4.37(4.20, 4.54)	4.52(4.13, 4.92)	2.99(2.83, 3.16)	5.98(5.74, 6.22)
Pancreatitis	18, 982(14, 417-24, 763)	34, 899(26, 411-49, 145)	1.96(1.80, 2.12)	1.72(1.50, 1.94)	1.30(1.05, 1.55)	3.05(2.79, 3.30)
Paralytic ileus and intestinal obstruction	35, 010(28, 028-44, 453)	38, 533(29, 528-53, 938)	0.29(0.14, 0.44)	0.61(0.39, 0.83)	-0.98(-1.17, -0.79)	1.47(1.21, 1.74)
Parkinson's disease	139, 160(120, 703-160, 966)	390, 652(324, 698-464, 390)	3.47(3.35, 3.59)	3.95(3.80, 4.09)	1.94(1.80, 2.08)	4.88(4.62, 5.14)
Pneumoconiosis	24, 711(19, 831-30, 276)	29, 045(21, 921-37, 205)	0.45(0.23, 0.66)	0.78(0.46, 1.09)	-1.24(-1.53, -0.95)	2.16(1.82, 2.51)
Poisonings	16, 799(12, 551-29, 618)	43, 039(22, 023-56, 435)	3.12(2.87, 3.37)	2.66(2.27, 3.06)	4.91(4.39, 5.44)	1.10(0.93, 1.26)
Police conflict and executions	224(47-386)	2, 158(653-3, 747)	7.39(7.05, 7.74)	5.51(4.62, 6.41)	7.04(6.78, 7.31)	10.00(9.57, 10.44)
Prostate cancer	41, 694(31, 265-55, 566)	131, 437(95, 268-185, 003)	3.90(3.75, 4.05)	5.24(4.99, 5.49)	1.64(1.47, 1.80)	5.12(4.80, 5.43)
Protein-energy malnutrition	22, 731(19, 706-25, 891)	11, 949(9, 615-14, 648)	-2.09(-2.39, -1.79)	-4.94(-5.58, -4.29)	-3.72(-3.98, -3.45)	3.02(2.59, 3.45)
Pruritus	4, 470(1, 958-8, 753)	14, 730(6, 388-28, 542)	3.91(3.71, 4.11)	3.58(3.50, 3.66)	2.98(2.74, 3.21)	5.57(4.96, 6.19)
Psoriasis	12, 081(8, 849-15, 830)	45, 369(33, 524-59, 616)	4.42(4.25, 4.58)	4.73(4.47, 4.99)	3.29(2.98, 3.60)	5.59(5.35, 5.82)
Pulmonary Arterial Hypertension	9, 659(7, 060-13, 280)	19, 235(12, 257-24, 197)	2.30(2.05, 2.54)	0.84(0.65, 1.03)	4.93(4.47, 5.40)	1.50(0.96, 2.05)
Rabies	535(237-856)	781(335-1, 352)	1.16(-0.33, 2.68)	-1.57(-4.22, 1.15)	8.97(7.35, 10.63)	-5.56(-8.45, -2.57)
Rheumatic heart disease	361, 004(297, 594-427, 286)	229, 653(180, 374-295, 978)	-1.55(-1.79, -1.32)	-1.88(-2.15, -1.61)	-2.95(-3.31, -2.58)	0.51(0.11, 0.92)
Rheumatoid arthritis	30, 812(24, 618-38, 733)	83, 287(64, 736-105, 657)	3.27(3.12, 3.42)	2.95(2.67, 3.23)	2.56(2.37, 2.75)	4.48(4.27, 4.69)
Road injuries	184, 942(160, 258-213, 088)	478, 719(407, 854-560, 143)	3.18(3.02, 3.34)	4.40(4.08, 4.72)	2.60(2.41, 2.79)	2.46(2.21, 2.71)
Scabies	16, 926(8, 537-31, 071)	47, 984(24, 299-87, 415)	3.40(3.30, 3.50)	3.16(3.06, 3.25)	2.52(2.40, 2.64)	4.87(4.66, 5.09)
Schistosomiasis	8, 232(6, 048-10, 943)	3, 635(2, 384-5, 891)	-2.64(-3.01, -2.28)	-3.92(-4.05, -3.79)	-4.88(-5.85, -3.90)	1.61(1.19, 2.03)
Schizophrenia	29, 502(21, 547-37, 330)	86, 968(64, 313-109, 809)	3.53(3.44, 3.63)	3.17(3.08, 3.27)	2.48(2.36, 2.59)	5.36(5.15, 5.57)
Self-harm	269, 193(177, 114-307, 938)	288, 930(232, 949-356, 952)	0.18(-0.14, 0.50)	1.68(1.55, 1.80)	-3.03(-3.23, -2.83)	2.59(1.53, 3.67)
Sexually transmitted infections excluding HIV	2, 594(1, 673-3, 981)	4, 355(2, 375-7, 634)	1.49(1.00, 1.98)	-0.40(-0.91, 0.12)	0.82(-0.20, 1.86)	4.39(3.85, 4.94)

Soft tissue and other extraosseous sarcomas	8, 028(5, 563-10, 782)	13, 433(9, 286-18, 601)	1.72(1.47, 1.97)	2.32(2.19, 2.46)	-0.35(-0.93, 0.23)	3.57(3.19, 3.95)
Stomach cancer	1, 125, 074(955, 720-1, 362, 290)	1, 557, 053(1, 193, 823-1, 968, 689)	1.06(0.89, 1.23)	1.85(1.59, 2.12)	-0.87(-1.10, -0.64)	2.51(2.24, 2.78)
Stroke	5, 489, 751(4, 830, 383-6, 196, 830)	8, 531, 116(7, 210, 033-9, 944, 567)	1.45(1.28, 1.63)	2.11(1.84, 2.39)	-0.25(-0.47, -0.04)	2.82(2.53, 3.11)
Testicular cancer	1, 112(881-1, 381)	2, 681(1, 955-3, 596)	2.93(2.70, 3.17)	3.76(3.44, 4.09)	-0.10(-0.40, 0.20)	5.85(5.42, 6.29)
Tetanus	5, 476(2, 114-8, 161)	543(134-1, 403)	-7.71(-8.78, -6.63)	-7.82(-10.04, -5.55)	-7.35(-8.59, -6.09)	-8.43(-9.03, -7.83)
Thyroid cancer	9, 420(7, 608-11, 386)	20, 075(16, 361-25, 085)	2.49(2.35, 2.63)	2.33(2.08, 2.57)	1.10(0.93, 1.27)	4.57(4.33, 4.82)
Tracheal, bronchus, and lung cancer	873, 670(746, 304-995, 713)	2, 999, 641(2, 386, 014-3, 664, 160)	4.10(3.94, 4.27)	5.02(4.67, 5.38)	2.65(2.49, 2.81)	4.81(4.59, 5.04)
Trachoma	1, 073(518-1, 825)	317(136-604)	-4.07(-4.66, -3.47)	-0.60(-1.66, 0.48)	-7.47(-8.77, -6.14)	-4.86(-5.24, -4.48)
Tuberculosis	419, 416(349, 199-496, 994)	170, 758(132, 534-212, 560)	-2.94(-3.23, -2.65)	-2.46(-2.85, -2.08)	-5.05(-5.43, -4.66)	-1.06(-1.57, -0.55)
Typhoid and paratyphoid	190(61-404)	181(73-333)	-0.12(-0.31, 0.07)	-1.27(-1.55, -0.99)	-0.19(-0.32, -0.05)	1.43(1.03, 1.83)
Upper digestive system diseases	248, 230(212, 832-287, 728)	241, 881(195, 361-306, 319)	-0.12(-0.35, 0.11)	-0.62(-0.91, -0.34)	-1.22(-1.61, -0.84)	1.60(1.27, 1.93)
Upper respiratory infections	29, 164(8, 758-42, 007)	17, 447(10, 553-27, 608)	-1.71(-1.98, -1.44)	-1.66(-1.91, -1.42)	-5.41(-5.73, -5.09)	3.04(2.46, 3.63)
Urinary diseases and male infertility	75, 841(52, 249-108, 263)	150, 527(98, 147-221, 606)	2.26(2.10, 2.42)	1.83(1.49, 2.17)	1.26(1.11, 1.42)	3.96(3.70, 4.21)
Urticaria	6, 866(4, 149-10, 490)	19, 197(11, 612-29, 851)	3.36(3.16, 3.56)	3.03(2.95, 3.11)	2.40(2.23, 2.57)	5.07(4.44, 5.70)
Uterine cancer	24, 486(18, 320-30, 865)	38, 790(28, 313-54, 649)	1.50(1.34, 1.67)	2.35(2.24, 2.46)	-0.31(-0.58, -0.04)	3.17(2.84, 3.49)
Varicella and herpes zoster	4, 130(3, 132-5, 329)	5, 515(3, 279-8, 602)	1.04(0.63, 1.44)	0.50(0.32, 0.67)	-1.38(-2.26, -0.49)	4.49(3.80, 5.19)
Vascular intestinal disorders	2, 033(1, 587-2, 413)	2, 457(1, 998-3, 057)	0.66(0.05, 1.28)	2.86(2.64, 3.08)	-3.24(-3.72, -2.75)	3.37(1.35, 5.42)
Viral skin diseases	8, 370(5, 300-12, 739)	24, 049(15, 099-36, 854)	3.45(3.36, 3.55)	3.28(3.18, 3.37)	2.59(2.48, 2.71)	4.81(4.60, 5.01)
Vitamin A deficiency	324(176-519)	909(471-1, 503)	3.56(3.34, 3.79)	4.10(3.88, 4.32)		4.06(3.58, 4.55)
**75-79 years**						
Acne vulgaris	488(311-747)	1, 678(1, 062-2, 602)	4.07(3.97, 4.17)	4.20(4.13, 4.27)	4.75(4.57, 4.92)	3.31(3.06, 3.56)
Acute glomerulonephritis	11, 585(8, 409-15, 979)	11, 525(7, 410-16, 156)	-0.07(-0.34, 0.21)	-0.95(-1.37, -0.53)	1.81(1.44, 2.19)	-1.61(-2.06, -1.16)

Acute hepatitis	22, 944(18, 745-28, 286)	5, 206(4, 118-6, 630)	-4.82(-5.27, -4.37)	-5.11(-5.39, -4.82)	-7.60(-8.52, -6.68)	-1.80(-2.77, -0.83)
Adverse effects of medical treatment	7, 522(4, 898-9, 555)	5, 923(4, 628-8, 061)	-0.94(-1.43, -0.45)	-1.01(-1.23, -0.79)	-2.03(-3.46, -0.58)	0.22(-0.03, 0.47)
Age-related and other hearing loss	366, 962(254, 482-511, 290)	1, 167, 541(811, 818-1, 596, 993)	3.78(3.70, 3.85)	3.14(3.03, 3.26)	4.85(4.77, 4.93)	3.31(3.14, 3.48)
Alcohol use disorders	11, 091(8, 441-14, 653)	37, 268(27, 007-50, 060)	3.77(3.39, 4.15)	3.62(3.14, 4.11)	6.78(6.27, 7.30)	0.75(-0.07, 1.57)
Alopecia areata	632(405-918)	1, 798(1, 134-2, 634)	3.40(3.32, 3.49)	3.44(3.37, 3.52)	3.92(3.81, 4.04)	2.71(2.47, 2.94)
Alzheimer's disease and other dementias	486, 994(225, 567-1, 104, 396)	1, 530, 697(757, 165-3, 333, 341)	3.69(3.57, 3.82)	3.74(3.69, 3.79)	3.74(3.69, 3.79)	3.58(3.17, 4.00)
Animal contact	4, 916(3, 745-5, 720)	3, 923(3, 219-4, 906)	-0.83(-1.08, -0.57)	0.00(-0.48, 0.47)	-2.45(-2.95, -1.94)	0.03(-0.25, 0.31)
Anxiety disorders	59, 048(41, 165-82, 706)	172, 385(116, 915-240, 307)	3.40(2.85, 3.95)	3.47(3.30, 3.64)	3.47(3.30, 3.64)	3.73(2.03, 5.45)
Aortic aneurysm	4, 502(3, 667-5, 591)	17, 904(13, 865-22, 799)	4.52(4.40, 4.64)	4.31(4.22, 4.39)	6.57(6.34, 6.81)	2.80(2.53, 3.07)
Appendicitis	6, 733(4, 714-8, 525)	3, 941(2, 906-5, 243)	-1.87(-2.15, -1.59)	-0.73(-1.16, -0.31)	-3.55(-4.09, -3.02)	-1.56(-1.97, -1.15)
Asthma	127, 963(100, 032-175, 680)	101, 412(81, 237-124, 191)	-0.76(-1.15, -0.37)	0.27(0.12, 0.43)	-2.02(-2.94, -1.09)	-0.53(-1.31, 0.26)
Atrial fibrillation and flutter	78, 185(59, 137-101, 104)	217, 682(167, 370-282, 024)	3.30(3.06, 3.53)	3.01(2.61, 3.42)	3.76(3.56, 3.96)	3.19(2.65, 3.73)
Attention-deficit/hyperactivity disorder	28(6-70)	73(13-182)	3.11(2.90, 3.32)	2.42(2.06, 2.78)	3.78(3.68, 3.88)	2.98(2.42, 3.55)
Autism spectrum disorders	6, 450(4, 381-9, 119)	24, 972(16, 878-35, 150)	4.45(4.36, 4.55)	4.53(4.36, 4.69)	5.41(5.34, 5.49)	3.38(3.14, 3.62)
Bacterial skin diseases	10, 027(7, 749-12, 056)	5, 044(3, 595-7, 789)	-2.35(-2.80, -1.90)	0.19(-0.69, 1.08)	-8.90(-9.35, -8.46)	1.61(1.09, 2.12)
Bipolar disorder	4, 527(2, 850-6, 587)	13, 036(8, 255-19, 312)	3.43(3.33, 3.52)	3.34(3.18, 3.49)	3.99(3.91, 4.07)	2.79(2.53, 3.04)
Bladder cancer	61, 805(47, 404-71, 372)	128, 475(101, 685-161, 428)	2.35(1.98, 2.72)	2.81(2.28, 3.35)	1.87(1.69, 2.06)	2.48(1.39, 3.58)
Blindness and vision loss	222, 148(158, 465-307, 995)	538, 797(375, 277-783, 150)	2.85(2.46, 3.24)	4.47(3.36, 5.60)	2.64(2.31, 2.98)	1.34(0.94, 1.74)
Brain and central nervous system cancer	29, 934(23, 323-38, 871)	105, 427(76, 691-131, 896)	4.07(3.81, 4.34)	4.94(4.34, 5.54)	4.48(4.11, 4.85)	2.62(2.42, 2.82)
Breast cancer	40, 526(33, 370-48, 530)	112, 175(89, 119-137, 452)	3.30(2.64, 3.97)	3.81(3.05, 4.57)	3.03(2.74, 3.33)	3.10(1.07, 5.18)
Cardiomyopathy and myocarditis	14, 585(11, 797-24, 050)	51, 865(39, 606-65, 298)	4.39(4.00, 4.78)	7.84(7.47, 8.21)	4.42(3.80, 5.04)	0.80(-0.14, 1.74)
Cervical cancer	33, 795(27, 557-40, 890)	64, 653(47, 693-81, 988)	2.07(1.69, 2.45)	0.84(0.52, 1.17)	2.60(1.56, 3.64)	2.44(2.29, 2.59)

Chronic kidney disease	256, 257(220, 951-301, 133)	636, 929(523, 941-748, 944)	2.86(2.46, 3.26)	2.66(1.90, 3.44)	3.93(3.38, 4.47)	1.54(1.09, 1.99)
Chronic obstructive pulmonary disease	4, 239, 247(3, 669, 477-4, 698, 859)	4, 030, 286(3, 390, 893-4, 794, 419)	-0.29(-0.60, 0.02)	0.79(0.32, 1.25)	-1.45(-1.68, -1.22)	0.08(-0.77, 0.94)
Cirrhosis and other chronic liver diseases	210, 029(179, 065-244, 961)	257, 702(207, 625-313, 846)	0.60(0.23, 0.98)	0.92(0.53, 1.31)	0.42(0.00, 0.84)	0.37(-0.56, 1.30)
Colon and rectum cancer	220, 831(189, 868-252, 229)	652, 888(531, 627-780, 605)	3.54(3.21, 3.86)	3.61(3.02, 4.20)	4.37(4.16, 4.58)	2.36(1.53, 3.20)
Conflict and terrorism	11(7-16)	37(27-52)	4.13(-4.27, 13.28)	17.68(10.12, 25.75)	-0.98(-22.14, 25.93)	-5.63(-11.78, 0.94)
Congenital birth defects	2, 863(1, 863-4, 158)	8, 335(5, 686-12, 102)	3.39(3.16, 3.63)	4.95(4.79, 5.11)	3.37(3.14, 3.61)	1.66(0.98, 2.35)
COVID-19	NA	1, 710(215-5, 771)	NA	NA	NA	NA
Cystic echinococcosis	313(213-428)	306(195-488)	-0.12(-0.47, 0.22)	-1.23(-1.61, -0.85)	0.52(0.07, 0.97)	0.08(-0.66, 0.83)
Cysticercosis	8, 830(3, 884-15, 833)	12, 741(5, 096-24, 196)	1.25(0.88, 1.63)	1.50(1.18, 1.81)	-0.17(-0.36, 0.03)	2.77(1.62, 3.93)
Decubitus ulcer	931(526-2, 150)	6, 736(3, 763-8, 631)	7.56(6.58, 8.54)	15.55(14.17, 16.94)	6.67(5.16, 8.21)	0.69(-1.23, 2.64)
Dengue	14(6-23)	17(7-28)	0.47(-0.79, 1.74)	-5.93(-8.94, -2.81)	4.95(3.79, 6.12)	1.46(1.00, 1.93)
Depressive disorders	93, 299(63, 769-127, 841)	320, 951(218, 826-448, 427)	4.06(3.97, 4.15)	5.00(4.78, 5.23)	4.54(4.44, 4.64)	2.41(2.33, 2.49)
Dermatitis	11, 889(6, 494-20, 201)	34, 392(19, 056-58, 436)	3.46(3.38, 3.55)	3.53(3.44, 3.62)	4.02(3.91, 4.13)	2.69(2.45, 2.93)
Diabetes mellitus	277, 734(235, 966-326, 313)	928, 776(766, 689-1, 117, 511)	3.88(3.42, 4.33)	5.44(5.23, 5.64)	3.89(2.52, 5.27)	2.18(1.98, 2.38)
Diarrheal diseases	37, 054(17, 620-55, 244)	9, 458(6, 210-16, 355)	-4.35(-4.64, -4.06)	-5.55(-6.00, -5.09)	-5.18(-5.32, -5.04)	-2.01(-2.81, -1.21)
Dietary iron deficiency	52, 611(35, 879-72, 927)	65, 357(43, 130-93, 275)	0.65(0.46, 0.85)	1.48(1.14, 1.82)	-0.08(-0.16, 0.00)	0.72(0.20, 1.24)
Drowning	30, 862(26, 967-35, 064)	58, 793(48, 986-69, 073)	2.02(1.70, 2.35)	2.48(1.86, 3.10)	2.39(1.99, 2.80)	0.80(0.62, 0.98)
Drug use disorders	17, 676(14, 360-21, 326)	15, 804(12, 130-19, 871)	-0.55(-0.91, -0.19)	0.44(-0.01, 0.89)	-3.13(-3.54, -2.73)	1.88(1.04, 2.74)
Encephalitis	1, 884(1, 545-2, 260)	3, 791(3, 116-4, 731)	2.21(1.82, 2.60)	2.73(2.01, 3.45)	3.10(2.78, 3.42)	0.84(0.03, 1.65)
Endocarditis	3, 517(1, 832-4, 634)	3, 417(2, 610-5, 011)	-0.19(-0.60, 0.23)	2.10(0.92, 3.30)	-3.12(-3.53, -2.71)	1.13(0.91, 1.35)
Endocrine, metabolic, blood, and immune disorders	38, 634(26, 543-55, 765)	104, 684(71, 159-153, 753)	3.45(2.80, 4.10)	4.48(3.86, 5.12)	3.37(1.54, 5.23)	2.40(1.89, 2.90)
Environmental heat and cold exposure	8, 525(3, 788-11, 050)	9, 304(4, 457-13, 328)	0.23(-0.12, 0.57)	0.31(-0.43, 1.06)	-0.80(-1.48, -0.11)	1.25(1.05, 1.44)

Esophageal cancer	359, 656(291, 259-413, 078)	692, 785(551, 424-843, 051)	2.11(1.61, 2.62)	3.29(2.51, 4.08)	1.17(0.70, 1.64)	1.74(0.76, 2.73)
Exposure to forces of nature	1, 374(1, 240-1, 523)	1, 665(1, 341-2, 059)	-2.38(-17.39, 15.36)	-6.52(-12.89, 0.32)	6.09(-35.50, 74.50)	-5.79(-15.74, 5.33)
Exposure to mechanical forces	10, 548(8, 269-14, 272)	33, 795(24, 545-44, 679)	3.88(3.61, 4.14)	3.72(3.29, 4.14)	6.89(6.65, 7.12)	0.74(0.07, 1.42)
Eye cancer	655(395-868)	1, 510(760-2, 094)	2.69(2.47, 2.90)	2.54(2.31, 2.77)	3.60(3.34, 3.85)	1.76(1.16, 2.36)
Falls	172, 278(139, 380-212, 136)	536, 788(396, 631-676, 012)	3.70(3.51, 3.89)	3.74(3.31, 4.17)	2.77(2.50, 3.04)	4.42(4.32, 4.53)
Fire, heat, and hot substances	19, 226(14, 778-22, 543)	25, 858(20, 299-31, 816)	0.68(-0.05, 1.41)	-0.31(-0.79, 0.18)	2.65(0.48, 4.87)	-0.19(-0.68, 0.31)
Food-borne trematodiases	15, 814(6, 682-31, 495)	23, 228(11, 931-40, 927)	1.09(0.25, 1.93)	-0.23(-1.23, 0.79)	2.02(1.63, 2.42)	0.55(-1.23, 2.37)
Foreign body	7, 590(5, 736-9, 055)	20, 915(13, 609-25, 759)	3.31(2.81, 3.81)	2.89(2.71, 3.07)	3.96(2.63, 5.32)	3.26(2.49, 4.05)
Fungal skin diseases	5, 933(2, 370-12, 318)	17, 052(6, 841-35, 287)	3.44(3.36, 3.53)	3.51(3.43, 3.60)	4.00(3.89, 4.11)	2.67(2.43, 2.90)
Gallbladder and biliary diseases	85, 140(58, 617-109, 064)	162, 094(113, 471-231, 126)	2.07(1.82, 2.32)	1.96(1.86, 2.05)	2.64(1.97, 3.32)	1.78(1.39, 2.17)
Gallbladder and biliary tract cancer	38, 797(29, 567-49, 573)	95, 020(67, 760-125, 702)	2.87(2.60, 3.13)	3.19(2.74, 3.65)	3.98(3.75, 4.20)	1.50(0.87, 2.14)
Gout	9, 262(5, 582-14, 141)	36, 927(21, 826-57, 478)	4.59(4.48, 4.70)	2.88(2.69, 3.07)	6.76(6.60, 6.91)	3.99(3.78, 4.20)
Gynecological diseases	17, 518(10, 754-26, 534)	35, 630(22, 774-53, 347)	2.32(2.01, 2.64)	3.11(2.99, 3.23)	1.00(0.22, 1.79)	2.85(2.30, 3.40)
Headache disorders	37, 312(10, 161-79, 211)	113, 265(29, 812-249, 197)	3.62(3.54, 3.71)	3.46(3.33, 3.59)	4.54(4.45, 4.63)	2.85(2.61, 3.08)
Hemoglobinopathies and hemolytic anemias	29, 373(22, 371-37, 978)	43, 107(33, 410-54, 511)	1.22(1.11, 1.34)	1.40(1.35, 1.45)	1.58(1.34, 1.82)	0.76(0.48, 1.04)
HIV/AIDS	1, 440(979-1, 883)	23, 125(16, 025-32, 389)	10.19(9.07, 11.31)	15.33(14.19, 16.48)	9.49(8.21, 10.78)	5.63(2.91, 8.42)
Hodgkin lymphoma	4, 756(2, 019-6, 536)	4, 543(2, 583-5, 956)	-0.34(-0.61, -0.07)	0.09(-0.23, 0.40)	-1.47(-2.18, -0.75)	0.34(0.13, 0.55)
Hypertensive heart disease	720, 318(502, 464-844, 195)	845, 603(614, 412-1, 088, 106)	0.36(0.10, 0.63)	-1.08(-1.31, -0.84)	0.56(-0.14, 1.26)	1.39(1.08, 1.70)
Idiopathic developmental intellectual disability	768(266-1, 535)	1, 437(397-3, 040)	1.94(1.70, 2.18)	4.17(3.45, 4.90)	0.89(0.81, 0.98)	0.89(0.81, 0.98)
Idiopathic epilepsy	15, 336(10, 195-21, 951)	35, 105(21, 054-53, 585)	2.81(2.65, 2.98)	3.46(3.31, 3.61)	1.74(1.59, 1.88)	3.36(2.86, 3.86)
Inflammatory bowel disease	9, 077(5, 173-11, 861)	12, 549(9, 577-17, 321)	0.99(0.75, 1.23)	1.88(1.47, 2.30)	0.27(0.02, 0.51)	0.78(0.32, 1.23)
Inguinal, femoral, and abdominal hernia	3, 532(2, 706-4, 700)	5, 901(4, 362-8, 009)	1.69(1.28, 2.10)	2.26(2.08, 2.44)	2.00(1.83, 2.16)	0.87(-0.41, 2.17)

Interpersonal violence	18, 384(15, 288-22, 262)	26, 935(21, 826-33, 554)	1.19(1.05, 1.32)	1.69(1.59, 1.78)	1.13(0.96, 1.30)	0.68(0.29, 1.07)
Interstitial lung disease and pulmonary sarcoidosis	9, 062(6, 975-14, 482)	28, 285(19, 195-38, 223)	3.83(3.55, 4.11)	3.50(3.26, 3.73)	6.93(6.48, 7.38)	1.27(0.55, 2.00)
Intestinal nematode infections	7, 108(3, 724-11, 350)	442(238-727)	-8.80(-9.30, -8.29)	-9.38(-9.87, -8.89)	-12.25(-13.21, -11.27)	-3.55(-4.65, -2.44)
Invasive Non-typhoidal Salmonella (iNTS)	299(79-699)	517(137-1, 282)	1.70(1.56, 1.84)	2.09(1.70, 2.47)	1.51(1.43, 1.60)	1.51(1.43, 1.60)
Iodine deficiency	1, 826(984-3, 121)	5, 520(2, 610-10, 468)	3.52(3.28, 3.77)	2.58(2.29, 2.87)	5.69(5.12, 6.26)	1.53(1.38, 1.69)
Ischemic heart disease	1, 327, 494(1, 168, 502-1, 504, 977)	4, 449, 426(3, 747, 385-5, 223, 117)	3.88(3.30, 4.45)	4.29(3.28, 5.32)	5.76(4.97, 6.57)	0.70(0.15, 1.26)
Kidney cancer	13, 923(12, 036-15, 588)	53, 030(43, 163-63, 406)	4.29(3.81, 4.78)	4.27(3.22, 5.33)	6.59(6.27, 6.92)	1.99(1.04, 2.95)
Larynx cancer	22, 486(18, 517-25, 797)	43, 434(34, 818-53, 708)	2.03(1.76, 2.29)	1.54(1.13, 1.95)	3.21(2.71, 3.72)	0.95(0.64, 1.26)
Leishmaniasis	78(7-227)	77(16-193)	-0.05(-0.67, 0.56)	-2.08(-2.40, -1.75)	2.87(1.33, 4.44)	-0.98(-1.93, -0.01)
Leprosy	29(17-44)	24(14-38)	-0.54(-0.94, -0.13)	-0.49(-0.76, -0.22)	-1.43(-2.13, -0.72)	0.42(-0.64, 1.49)
Leukemia	36, 940(30, 020-44, 744)	83, 594(61, 226-107, 340)	2.64(2.30, 2.97)	2.83(2.19, 3.47)	3.36(3.15, 3.58)	1.71(0.93, 2.50)
Lip and oral cavity cancer	16, 035(13, 871-18, 310)	48, 988(39, 256-59, 975)	3.58(3.36, 3.80)	2.95(2.79, 3.11)	5.18(4.95, 5.42)	2.26(1.63, 2.89)
Liver cancer	99, 679(83, 824-115, 590)	254, 442(208, 839-310, 115)	3.09(2.75, 3.44)	5.17(4.18, 6.18)	2.44(2.15, 2.73)	1.70(1.34, 2.05)
Low back pain	259, 314(160, 168-382, 709)	574, 602(357, 034-837, 523)	2.61(2.45, 2.77)	1.67(1.40, 1.95)	3.57(3.48, 3.65)	2.38(1.94, 2.82)
Lower extremity peripheral arterial disease	11, 060(5, 759-19, 703)	29, 865(15, 993-51, 643)	3.15(2.99, 3.31)	3.41(3.34, 3.48)	3.56(3.29, 3.82)	2.29(1.94, 2.64)
Lower respiratory infections	380, 028(308, 288-435, 583)	387, 892(313, 533-482, 546)	-0.11(-0.44, 0.23)	0.39(-0.60, 1.40)	-0.35(-0.46, -0.23)	-0.35(-0.46, -0.23)
Malaria	169(25-1, 529)	36, 469(22, 423-48, 668)	-18.54(-22.26, -14.64)	-15.10(-17.72, -12.40)	-14.18(-18.14, -10.02)	-26.98(-34.02, -19.20)
Malignant neoplasm of bone and articular cartilage	7, 036(4, 440-12, 267)	9, 785(5, 073-12, 962)	5.62(5.33, 5.91)	7.80(7.26, 8.33)	7.02(6.45, 7.59)	1.28(1.11, 1.44)
Malignant skin melanoma	3, 435(2, 394-4, 524)	0(0-0)	3.27(2.96, 3.57)	3.02(2.82, 3.22)	4.60(4.14, 5.06)	1.80(1.02, 2.58)
Meningitis	10, 440(9, 044-12, 056)	7, 754(6, 510-9, 302)	-0.99(-1.58, -0.40)	-2.88(-3.74, -2.01)	0.18(-0.93, 1.30)	-0.43(-1.26, 0.41)
Mesothelioma	1, 769(1, 427-2, 037)	5, 526(4, 404-6, 640)	3.73(3.48, 3.98)	2.93(2.54, 3.32)	6.80(6.54, 7.07)	0.63(-0.02, 1.28)
Motor neuron disease	795(490-1, 036)	3, 721(2, 336-4, 895)	4.89(4.13, 5.65)	7.46(6.42, 8.50)	2.71(1.19, 4.27)	5.39(5.08, 5.71)

Multiple myeloma	2, 814(1, 863-5, 680)	27, 681(18, 570-36, 395)	7.87(7.22, 8.53)	15.45(14.24, 16.68)	5.10(3.72, 6.51)	3.98(3.48, 4.48)
Multiple sclerosis	89(58-124)	399(293-525)	4.99(4.78, 5.20)	6.05(5.97, 6.12)	4.99(4.90, 5.07)	4.18(3.51, 4.86)
Nasopharynx cancer	32, 980(28, 223-37, 503)	39, 260(32, 042-47, 545)	0.39(0.01, 0.77)	0.42(-0.03, 0.87)	0.19(-0.80, 1.18)	0.84(0.46, 1.21)
Neck pain	63, 134(33, 155-106, 189)	190, 398(100, 071-316, 560)	3.56(3.33, 3.79)	2.76(2.61, 2.92)	5.21(4.75, 5.67)	2.87(2.33, 3.41)
Neonatal disorders	1, 725(1, 183-2, 505)	8, 056(5, 856-10, 423)	5.05(4.92, 5.18)	3.43(3.24, 3.62)	6.63(6.34, 6.92)	4.79(4.66, 4.91)
Neuroblastoma and other peripheral nervous cell tumors	122(80-182)	1, 358(942-1, 703)	8.00(7.56, 8.44)	6.45(5.99, 6.92)	11.74(10.49, 12.99)	5.16(4.88, 5.43)
Non-Hodgkin lymphoma	27, 491(23, 512-34, 053)	78, 493(62, 525-94, 422)	3.29(2.94, 3.65)	3.59(3.16, 4.03)	4.47(3.52, 5.43)	1.96(1.67, 2.25)
Non-melanoma skin cancer	10, 119(8, 167-12, 663)	35, 702(28, 308-43, 423)	4.11(3.78, 4.43)	4.08(3.49, 4.67)	6.20(5.97, 6.44)	1.40(0.61, 2.20)
Non-rheumatic valvular heart disease	6, 422(4, 749-8, 949)	15, 273(10, 816-21, 996)	2.81(2.39, 3.23)	3.52(3.35, 3.70)	3.23(1.96, 4.51)	1.62(1.43, 1.81)
Oral disorders	128, 426(80, 684-189, 048)	354, 886(224, 348-512, 623)	3.40(3.08, 3.73)	1.71(1.25, 2.18)	4.43(4.06, 4.80)	4.46(3.83, 5.09)
Osteoarthritis	121, 044(57, 505-246, 859)	410, 636(195, 604-838, 245)	4.01(3.88, 4.14)	3.46(3.23, 3.69)	4.26(4.10, 4.43)	4.25(4.05, 4.45)
Other cardiovascular and circulatory diseases	24, 458(17, 702-33, 958)	58, 522(40, 320-87, 863)	2.82(2.52, 3.13)	2.78(2.62, 2.94)	3.57(3.11, 4.02)	1.85(1.05, 2.65)
Other chronic respiratory diseases	2, 394(1, 810-4, 133)	9, 272(6, 855-11, 768)	4.50(4.24, 4.75)	5.72(5.37, 6.06)	8.28(7.68, 8.87)	-0.44(-0.76, -0.12)
Other digestive diseases	34, 861(23, 873-43, 356)	30, 518(25, 167-37, 340)	-0.46(-0.75, -0.17)	-1.49(-1.91, -1.07)	-1.26(-1.42, -1.10)	1.80(1.00, 2.61)
Other intestinal infectious diseases	71(2-209)	70(2-201)	-0.17(-0.68, 0.35)	-0.07(-0.53, 0.39)	-0.37(-1.77, 1.05)	-0.27(-0.50, -0.03)
Other malignant neoplasms	43, 446(29, 782-54, 472)	85, 890(66, 448-107, 264)	2.15(1.92, 2.38)	1.05(0.95, 1.16)	3.73(3.23, 4.23)	1.83(1.31, 2.35)
Other mental disorders	21, 945(14, 291-32, 731)	64, 430(42, 120-95, 372)	3.51(3.42, 3.59)	3.57(3.47, 3.66)	4.13(4.03, 4.24)	2.66(2.44, 2.89)
Other musculoskeletal disorders	75, 512(45, 434-118, 464)	238, 259(141, 811-382, 646)	3.68(3.48, 3.88)	3.92(3.63, 4.22)	4.77(4.57, 4.97)	2.29(1.77, 2.82)
Other neglected tropical diseases	2, 310(1, 669-3, 100)	3, 267(2, 405-4, 283)	1.06(0.76, 1.36)	1.54(1.22, 1.87)	0.90(0.56, 1.24)	0.56(-0.16, 1.29)
Other neoplasms	3, 764(2, 472-6, 648)	17, 341(11, 834-27, 779)	4.97(4.70, 5.24)	4.83(4.34, 5.31)	7.29(6.73, 7.85)	2.76(2.57, 2.94)
Other neurological disorders	3, 186(2, 316-4, 214)	27, 167(21, 208-34, 607)	7.17(6.80, 7.54)	8.23(7.88, 8.57)	6.70(6.45, 6.96)	6.49(5.39, 7.61)
Other nutritional deficiencies	2, 459(1, 973-3, 016)	5, 409(4, 163-6, 846)	2.65(2.17, 3.13)	0.38(-0.31, 1.08)	5.98(5.25, 6.71)	0.20(-0.46, 0.86)

Other pharynx cancer	5, 858(4, 920-6, 855)	10, 644(8, 454-12, 957)	1.78(1.42, 2.15)	1.42(0.90, 1.95)	2.14(1.30, 2.98)	2.15(1.79, 2.51)
Other sense organ diseases	26, 608(12, 931-48, 850)	79, 824(38, 547-146, 324)	3.59(3.50, 3.69)	3.68(3.61, 3.75)	4.30(4.12, 4.48)	2.85(2.63, 3.07)
Other skin and subcutaneous diseases	11, 158(5, 808-19, 378)	40, 920(21, 324-71, 876)	4.28(4.17, 4.38)	4.06(3.89, 4.22)	5.09(4.98, 5.20)	3.44(3.17, 3.71)
Other transport injuries	7, 233(5, 972-8, 501)	11, 040(8, 898-13, 511)	1.46(0.97, 1.96)	3.25(2.84, 3.66)	1.10(0.41, 1.80)	0.21(-1.01, 1.45)
Other unintentional injuries	13, 185(10, 003-17, 346)	22, 243(15, 977-30, 246)	1.73(1.45, 2.00)	1.90(1.54, 2.26)	1.89(1.63, 2.15)	1.21(0.52, 1.91)
Other unspecified infectious diseases	5, 429(3, 040-7, 198)	6, 399(4, 115-8, 963)	0.52(0.27, 0.77)	0.75(0.17, 1.34)	-0.78(-1.22, -0.33)	1.65(1.50, 1.81)
Otitis media	2, 096(1, 107-3, 643)	4, 284(2, 130-7, 505)	2.28(2.19, 2.37)	2.31(2.18, 2.44)	2.74(2.66, 2.83)	1.64(1.37, 1.90)
Ovarian cancer	13, 083(10, 411-16, 391)	33, 784(25, 099-45, 454)	3.08(2.65, 3.52)	4.09(3.23, 4.97)	2.05(1.84, 2.26)	3.34(2.28, 4.41)
Pancreatic cancer	64, 336(55, 294-75, 059)	252, 158(201, 662-306, 106)	4.54(4.30, 4.78)	5.29(5.15, 5.43)	5.29(5.15, 5.43)	3.16(2.47, 3.85)
Pancreatitis	13, 439(10, 286-18, 143)	26, 539(20, 525-35, 138)	2.17(1.96, 2.39)	2.16(1.83, 2.49)	3.21(3.00, 3.41)	1.15(0.59, 1.72)
Paralytic ileus and intestinal obstruction	32, 452(26, 022-40, 424)	39, 715(30, 877-54, 290)	0.70(0.38, 1.02)	1.53(1.33, 1.72)	0.69(0.25, 1.13)	-0.11(-1.01, 0.81)
Parkinson's disease	145, 612(125, 538-166, 032)	415, 657(353, 025-488, 467)	3.44(3.17, 3.72)	3.87(3.72, 4.02)	3.87(3.72, 4.02)	2.63(1.80, 3.46)
Pneumoconiosis	16, 162(12, 967-19, 818)	23, 453(18, 416-29, 761)	1.10(0.82, 1.39)	1.54(1.01, 2.07)	1.23(0.87, 1.60)	0.32(0.15, 0.49)
Poisonings	10, 449(7, 737-20, 137)	29, 335(16, 020-37, 840)	3.40(3.05, 3.75)	3.39(2.76, 4.02)	6.93(6.38, 7.48)	-1.37(-1.73, -1.01)
Police conflict and executions	87(19-151)	926(266-1, 664)	7.85(7.30, 8.40)	5.43(3.86, 7.02)	8.63(8.27, 9.00)	9.50(9.31, 9.69)
Prostate cancer	37, 181(28, 231-53, 216)	128, 842(95, 770-177, 680)	3.97(3.58, 4.36)	5.39(4.96, 5.82)	4.13(3.05, 5.22)	2.27(1.96, 2.58)
Protein-energy malnutrition	25, 493(22, 387-29, 216)	15, 707(12, 843-18, 790)	-1.65(-1.96, -1.34)	-3.99(-4.54, -3.43)	-1.95(-2.21, -1.69)	1.22(0.58, 1.86)
Pruritus	2, 848(1, 211-5, 823)	9, 484(4, 072-19, 259)	3.94(3.84, 4.04)	3.77(3.63, 3.92)	4.61(4.51, 4.71)	3.23(2.95, 3.51)
Psoriasis	7, 091(5, 146-9, 510)	27, 162(19, 921-36, 629)	4.44(4.31, 4.56)	4.87(4.81, 4.94)	4.87(4.81, 4.94)	3.37(2.98, 3.76)
Pulmonary Arterial Hypertension	8, 621(6, 103-12, 104)	17, 970(10, 826-22, 948)	2.37(2.03, 2.70)	1.01(0.76, 1.27)	7.39(6.86, 7.91)	-0.88(-1.76, 0.00)
Rabies	218(110-329)	403(202-645)	1.06(-1.55, 3.74)	-0.79(-4.92, 3.51)	10.92(4.43, 17.82)	-8.90(-10.13, -7.66)
Rheumatic heart disease	278, 165(229, 565-336, 518)	192, 694(151, 151-245, 722)	-1.37(-1.84, -0.89)	-1.84(-2.19, -1.49)	-1.29(-2.59, 0.03)	-1.21(-1.44, -0.99)

Rheumatoid arthritis	21, 103(17, 173-25, 657)	59, 486(46, 874-74, 191)	3.28(2.99, 3.57)	3.26(2.73, 3.78)	4.02(3.47, 4.58)	2.28(2.05, 2.52)
Road injuries	101, 123(87, 397-116, 582)	273, 306(233, 648-319, 663)	3.11(2.68, 3.55)	5.12(4.20, 6.05)	4.11(3.40, 4.82)	-0.25(-0.67, 0.17)
Scabies	11, 187(5, 823-18, 765)	32, 557(16, 773-53, 869)	3.50(3.40, 3.60)	3.59(3.53, 3.66)	4.18(4.02, 4.35)	2.77(2.52, 3.02)
Schistosomiasis	5, 041(3, 626-6, 887)	2, 335(1, 575-3, 737)	-2.77(-3.54, -1.99)	-3.62(-4.19, -3.05)	-3.77(-5.89, -1.60)	-0.95(-1.34, -0.55)
Schizophrenia	13, 594(10, 095-17, 178)	41, 150(30, 214-51, 653)	3.60(3.49, 3.71)	3.44(3.27, 3.60)	4.15(4.04, 4.25)	3.05(2.76, 3.35)
Self-harm	168, 297(111, 941-192, 536)	198, 156(163, 478-238, 995)	0.47(0.20, 0.75)	2.46(2.20, 2.72)	-1.17(-1.39, -0.95)	0.26(-0.51, 1.03)
Sexually transmitted infections excluding HIV	1, 595(1, 028-2, 401)	2, 591(1, 483-4, 325)	1.47(0.96, 1.99)	0.28(-1.24, 1.81)	2.29(2.09, 2.49)	1.73(1.35, 2.11)
Soft tissue and other extraosseous sarcomas	4, 937(3, 227-6, 447)	9, 070(6, 011-12, 375)	1.93(1.48, 2.38)	2.52(1.83, 3.21)	1.49(1.06, 1.92)	1.65(0.78, 2.53)
Stomach cancer	635, 038(549, 653-777, 027)	1, 031, 082(803, 136-1, 273, 832)	1.40(0.98, 1.82)	2.52(1.61, 3.44)	1.36(0.93, 1.80)	0.01(-0.25, 0.27)
Stroke	4, 329, 976(3, 835, 101-4, 847, 383)	7, 470, 018(6, 278, 752-8, 653, 422)	1.66(1.20, 2.13)	2.58(1.71, 3.45)	1.46(0.95, 1.96)	0.65(0.42, 0.88)
Testicular cancer	782(613-976)	2, 176(1, 581-2, 900)	3.34(2.82, 3.87)	4.82(4.25, 5.40)	1.56(1.02, 2.10)	3.54(2.88, 4.21)
Tetanus	3, 652(1, 710-5, 722)	401(128-999)	-7.00(-7.49, -6.51)	-7.35(-8.38, -6.32)	-6.53(-7.10, -5.96)	-7.58(-7.85, -7.32)
Thyroid cancer	8, 521(7, 102-9, 916)	23, 227(17, 906-28, 856)	3.20(2.98, 3.42)	2.72(2.58, 2.87)	5.04(4.70, 5.38)	1.31(0.76, 1.87)
Tracheal, bronchus, and lung cancer	494, 239(428, 634-563, 915)	1, 960, 769(1, 584, 738-2, 371, 653)	4.43(3.93, 4.93)	5.70(4.59, 6.82)	5.29(4.57, 6.01)	2.05(1.62, 2.48)
Trachoma	874(472-1, 494)	259(125-498)	-3.81(-4.76, -2.86)	-0.91(-2.24, 0.44)	-6.38(-8.79, -3.91)	-4.77(-5.19, -4.34)
Tuberculosis	272, 096(227, 550-320, 746)	110, 032(88, 115-138, 474)	-2.95(-3.34, -2.56)	-2.60(-2.91, -2.28)	-3.52(-4.59, -2.43)	-3.11(-3.32, -2.90)
Typhoid and paratyphoid	107(40-220)	116(50-231)	0.17(-0.05, 0.38)	-1.09(-1.55, -0.63)	1.74(1.38, 2.09)	-0.08(-0.33, 0.18)
Upper digestive system diseases	184, 402(158, 335-212, 525)	194, 501(160, 727-239, 107)	0.08(-0.24, 0.40)	-0.47(-0.73, -0.21)	0.63(-0.24, 1.51)	-0.42(-0.56, -0.28)
Upper respiratory infections	28, 514(6, 855-41, 917)	11, 621(7, 073-20, 444)	-3.01(-3.30, -2.71)	-1.67(-1.95, -1.40)	-6.55(-6.93, -6.16)	0.13(-0.53, 0.81)
Urinary diseases and male infertility	55, 330(39, 479-74, 434)	119, 754(82, 686-169, 398)	2.52(2.27, 2.77)	2.31(1.88, 2.74)	2.77(2.60, 2.94)	2.30(1.70, 2.90)
Urticaria	3, 870(2, 307-6, 042)	10, 993(6, 578-17, 273)	3.40(3.31, 3.50)	3.45(3.37, 3.53)	3.93(3.81, 4.06)	2.68(2.43, 2.93)
Uterine cancer	14, 513(10, 737-18, 046)	22, 430(16, 742-32, 102)	1.38(1.07, 1.69)	2.82(2.57, 3.08)	1.27(0.84, 1.71)	0.40(-0.38, 1.18)

Varicella and herpes zoster	4, 208(3, 225-5, 330)	4, 252(2, 721-6, 512)	-0.11(-0.36, 0.14)	-0.59(-0.92, -0.25)	-1.18(-1.70, -0.65)	1.38(1.13, 1.63)
Vascular intestinal disorders	1, 896(1, 369-2, 263)	2, 415(1, 969-2, 932)	0.72(0.49, 0.94)	2.83(2.45, 3.22)	-0.70(-0.92, -0.48)	-0.05(-0.49, 0.38)
Viral skin diseases	4, 477(2, 885-6, 694)	13, 331(8, 543-19, 988)	3.56(3.47, 3.65)	3.53(3.41, 3.66)	4.19(4.10, 4.28)	2.75(2.50, 3.01)
Vitamin A deficiency	187(106-296)	528(299-866)	3.50(3.31, 3.69)	4.31(4.20, 4.41)	4.31(4.20, 4.41)	1.90(1.33, 2.46)
**80-84 years**						
Acne vulgaris	182(116-281)	797(500-1, 242)	4.89(4.82, 4.96)	4.99(4.85, 5.14)	5.19(5.12, 5.26)	4.39(4.28, 4.50)
Acute glomerulonephritis	5, 418(3, 866-7, 499)	8, 061(5, 319-10, 648)	1.43(0.84, 2.02)	1.32(1.10, 1.53)	2.92(1.32, 4.53)	-0.20(-0.95, 0.56)
Acute hepatitis	8, 349(6, 672-10, 361)	2, 869(2, 200-3, 634)	-3.45(-3.76, -3.15)	-3.14(-3.40, -2.88)	-5.64(-6.12, -5.15)	-0.83(-1.56, -0.09)
Adverse effects of medical treatment	3, 658(2, 281-4, 726)	4, 109(3, 257-5, 564)	0.41(0.11, 0.71)	0.46(0.08, 0.84)	-1.21(-1.95, -0.46)	2.00(1.73, 2.27)
Age-related and other hearing loss	204, 211(144, 618-273, 102)	804, 036(580, 516-1, 071, 028)	4.53(4.47, 4.59)	3.92(3.81, 4.03)	5.37(5.32, 5.41)	4.26(4.11, 4.40)
Alcohol use disorders	4, 304(3, 026-5, 889)	20, 110(14, 088-27, 308)	5.07(4.70, 5.45)	5.41(5.22, 5.60)	6.82(5.77, 7.89)	2.68(2.30, 3.06)
Alopecia areata	243(157-353)	878(570-1, 278)	4.25(4.15, 4.35)	4.35(4.15, 4.55)	4.50(4.39, 4.60)	3.84(3.68, 4.01)
Alzheimer's disease and other dementias	541, 691(230, 454-1, 258, 624)	2, 125, 107(982, 062-4, 558, 620)	4.38(4.19, 4.56)	4.22(4.10, 4.34)	4.35(4.23, 4.48)	4.46(4.01, 4.92)
Animal contact	2, 039(1, 577-2, 430)	2, 426(1, 966-2, 938)	0.60(0.39, 0.80)	1.42(0.99, 1.85)	-1.19(-1.43, -0.95)	1.77(1.40, 2.14)
Anxiety disorders	27, 417(18, 579-38, 572)	102, 402(70, 598-143, 531)	4.39(4.16, 4.62)	5.28(4.89, 5.67)	3.61(3.50, 3.71)	4.45(3.84, 5.07)
Aortic aneurysm	2, 055(1, 663-2, 448)	10, 599(8, 511-12, 956)	5.38(4.97, 5.78)	5.13(4.86, 5.40)	7.03(6.04, 8.03)	3.91(3.23, 4.59)
Appendicitis	3, 934(2, 705-4, 963)	3, 540(2, 654-4, 735)	-0.27(-0.68, 0.14)	0.71(0.43, 0.99)	-1.54(-2.69, -0.37)	0.32(-0.09, 0.72)
Asthma	73, 390(56, 078-103, 141)	88, 826(71, 576-106, 272)	0.59(0.17, 1.02)	1.69(1.48, 1.91)	-0.58(-1.81, 0.66)	0.57(0.37, 0.78)
Atrial fibrillation and flutter	72, 254(57, 817-91, 258)	251, 218(201, 037-311, 428)	4.11(3.88, 4.34)	4.11(4.01, 4.21)	4.11(4.01, 4.21)	4.10(3.36, 4.84)
Attention-deficit/hyperactivity disorder	1(0-4)	4(0-16)	4.87(1.09, 8.80)	7.51(-3.53, 19.81)	2.21(-0.18, 4.66)	5.58(4.50, 6.67)
Autism spectrum disorders	2, 202(1, 481-3, 173)	12, 080(8, 256-16, 961)	5.67(5.60, 5.74)	5.69(5.64, 5.75)	6.29(6.11, 6.48)	4.88(4.78, 4.99)
Bacterial skin diseases	8, 594(6, 452-10, 496)	4, 668(3, 132-8, 088)	-2.02(-2.39, -1.65)	0.97(0.71, 1.23)	-8.92(-9.26, -8.58)	3.26(2.05, 4.49)

Bipolar disorder	1, 907(1, 210-2, 729)	7, 027(4, 477-10, 197)	4.33(4.25, 4.40)	4.36(4.21, 4.51)	4.65(4.57, 4.72)	3.94(3.82, 4.06)
Bladder cancer	32, 888(25, 552-37, 881)	96, 249(77, 626-118, 707)	3.61(3.30, 3.92)	3.78(3.03, 4.53)	3.10(2.80, 3.40)	4.11(3.89, 4.33)
Blindness and vision loss	125, 411(89, 012-172, 749)	372, 809(261, 826-534, 161)	3.68(3.23, 4.12)	4.94(3.67, 6.22)	3.37(3.20, 3.54)	2.69(2.13, 3.26)
Brain and central nervous system cancer	11, 311(8, 612-14, 607)	52, 968(36, 842-66, 688)	5.19(4.98, 5.39)	5.56(5.47, 5.66)	6.05(5.49, 6.62)	3.78(3.53, 4.03)
Breast cancer	18, 805(15, 710-21, 826)	64, 391(50, 987-78, 644)	4.15(3.59, 4.71)	5.19(4.37, 6.01)	3.84(3.59, 4.09)	3.80(2.66, 4.95)
Cardiomyopathy and myocarditis	10, 003(8, 051-16, 149)	50, 040(37, 761-60, 810)	5.42(4.71, 6.13)	9.60(7.43, 11.81)	4.72(4.28, 5.17)	2.11(1.80, 2.43)
Cervical cancer	13, 283(10, 863-15, 775)	35, 203(26, 509-44, 071)	3.35(2.83, 3.87)	2.80(2.12, 3.47)	4.09(3.01, 5.18)	2.78(2.56, 3.00)
Chronic kidney disease	137, 837(118, 252-163, 426)	478, 496(392, 779-558, 749)	4.16(3.92, 4.41)	3.88(3.28, 4.48)	5.05(4.87, 5.23)	3.30(2.89, 3.72)
Chronic obstructive pulmonary disease	2, 758, 574(2, 336, 889-3, 040, 138)	3, 873, 601(3, 199, 744-4, 478, 223)	1.07(0.80, 1.35)	2.11(1.55, 2.67)	-0.13(-0.42, 0.16)	1.54(0.97, 2.10)
Cirrhosis and other chronic liver diseases	87, 987(74, 310-103, 539)	155, 024(123, 245-189, 220)	1.87(1.67, 2.07)	2.30(2.09, 2.50)	1.53(1.20, 1.87)	1.81(1.43, 2.18)
Colon and rectum cancer	102, 244(88, 263-115, 750)	412, 968(334, 908-480, 639)	4.72(4.27, 5.17)	4.81(4.60, 5.02)	5.39(4.07, 6.73)	3.96(3.69, 4.22)
Conflict and terrorism	2(1-4)	18(12-26)	6.21(4.78, 7.66)	14.29(10.54, 18.17)	4.31(3.08, 5.55)	0.29(-1.86, 2.48)
Congenital birth defects	1, 161(787-1, 673)	4, 495(3, 071-6, 488)	4.32(4.04, 4.60)	5.64(5.44, 5.85)	4.08(3.62, 4.55)	2.90(2.30, 3.51)
COVID-19	NA	178(119-255)	NA	NA	NA	NA
Cystic echinococcosis	148(95-213)	8, 139(3, 556-15, 025)	0.64(0.22, 1.06)	0.10(-0.20, 0.40)	0.89(-0.27, 2.07)	0.66(0.49, 0.83)
Cysticercosis	4, 388(2, 173-7, 680)	8, 103(4, 427-10, 561)	2.15(1.69, 2.61)	2.39(1.73, 3.06)	0.75(0.55, 0.94)	3.77(2.43, 5.12)
Decubitus ulcer	805(452-1, 978)	9(4-16)	8.83(7.88, 9.79)	17.53(16.07, 19.01)	7.99(6.51, 9.48)	1.17(-0.48, 2.84)
Dengue	5(2-8)	187, 798(129, 913-261, 602)	1.85(0.89, 2.82)	-0.71(-2.15, 0.75)	4.03(2.47, 5.62)	1.48(1.01, 1.96)
Depressive disorders	43, 813(30, 299-60, 614)	19, 480(10, 562-34, 814)	4.80(4.69, 4.92)	5.57(5.31, 5.83)	5.13(5.05, 5.21)	3.55(3.36, 3.75)
Dermatitis	5, 276(2, 873-9, 381)	558, 817(467, 960-663, 442)	4.33(4.25, 4.40)	4.45(4.29, 4.60)	4.58(4.50, 4.66)	3.88(3.75, 4.01)
Diabetes mellitus	119, 571(101, 163-141, 352)	8, 114(5, 107-14, 585)	5.00(4.78, 5.21)	6.19(5.88, 6.50)	5.12(4.97, 5.28)	3.44(2.91, 3.97)
Diarrheal diseases	25, 028(11, 227-37, 836)	59, 428(40, 858-83, 223)	-3.60(-3.87, -3.33)	-4.60(-4.84, -4.37)	-5.08(-5.51, -4.64)	-0.61(-1.06, -0.16)

Dietary iron deficiency	34, 827(23, 601-47, 640)	44, 422(37, 267-52, 062)	1.75(1.64, 1.86)	2.83(2.65, 3.01)	0.74(0.64, 0.85)	1.75(1.52, 1.98)
Drowning	16, 883(14, 564-19, 288)	8, 928(6, 938-11, 093)	3.26(2.84, 3.67)	3.62(3.11, 4.13)	4.24(3.44, 5.04)	1.25(0.67, 1.83)
Drug use disorders	7, 250(5, 788-8, 934)	1, 658(1, 338-2, 080)	0.54(0.23, 0.85)	2.10(1.68, 2.52)	-2.69(-3.21, -2.17)	3.24(2.76, 3.72)
Encephalitis	695(573-854)	2, 896(2, 163-4, 222)	2.91(2.51, 3.32)	4.13(3.31, 4.95)	3.25(2.78, 3.73)	1.27(0.69, 1.85)
Endocarditis	2, 142(1, 111-2, 892)	65, 933(46, 247-90, 419)	1.14(0.50, 1.77)	3.78(1.96, 5.62)	-2.41(-3.03, -1.78)	2.87(2.55, 3.18)
Endocrine, metabolic, blood, and immune disorders	18, 592(13, 356-25, 949)	9, 142(4, 106-12, 833)	4.22(3.87, 4.57)	5.17(4.70, 5.65)	3.80(3.15, 4.45)	3.89(3.31, 4.47)
Environmental heat and cold exposure	6, 387(2, 562-8, 329)	389, 120(315, 900-467, 114)	1.13(0.82, 1.44)	0.80(0.36, 1.23)	0.41(0.07, 0.76)	2.35(1.62, 3.10)
Esophageal cancer	134, 669(112, 143-153, 655)	1, 452(1, 142-1, 878)	3.58(3.18, 3.98)	4.82(4.02, 5.62)	3.15(2.71, 3.59)	2.59(2.02, 3.17)
Exposure to forces of nature	760(687-847)	21, 583(15, 849-28, 505)	-1.37(-16.55, 16.56)	-6.31(-12.94, 0.83)	6.61(-35.27, 75.56)	-3.46(-12.88, 6.99)
Exposure to mechanical forces	5, 045(3, 972-6, 854)	1, 227(609-1, 651)	4.96(4.66, 5.26)	4.69(4.15, 5.24)	7.53(7.17, 7.89)	2.19(1.61, 2.76)
Eye cancer	429(241-575)	508, 397(382, 226-635, 591)	3.21(3.05, 3.38)	3.21(3.05, 3.38)	3.21(3.05, 3.38)	3.21(3.05, 3.38)
Falls	119, 569(98, 863-146, 196)	23, 493(18, 151-28, 509)	4.96(4.71, 5.21)	5.23(4.95, 5.51)	4.17(3.70, 4.64)	5.30(4.80, 5.81)
Fire, heat, and hot substances	13, 731(9, 809-15, 982)	12, 959(6, 550-22, 750)	1.83(1.11, 2.57)	0.89(-0.15, 1.93)	3.26(1.33, 5.23)	1.56(1.33, 1.80)
Food-borne trematodiases	6, 640(2, 915-13, 102)	16, 774(10, 836-20, 171)	2.12(1.68, 2.57)	0.81(0.00, 1.62)	2.37(2.13, 2.61)	2.40(1.46, 3.35)
Foreign body	4, 317(3, 318-5, 130)	13, 025(5, 165-26, 879)	4.56(4.23, 4.90)	4.47(4.32, 4.62)	4.96(3.95, 5.98)	4.27(4.13, 4.41)
Fungal skin diseases	3, 528(1, 407-7, 268)	125, 932(91, 294-176, 506)	4.33(4.25, 4.41)	4.43(4.27, 4.59)	4.57(4.49, 4.66)	3.91(3.78, 4.04)
Gallbladder and biliary diseases	49, 260(33, 888-62, 911)	57, 943(39, 287-72, 323)	3.09(2.86, 3.33)	3.07(2.84, 3.31)	3.30(2.64, 3.96)	2.97(2.85, 3.09)
Gallbladder and biliary tract cancer	17, 955(13, 366-22, 862)	22, 456(12, 698-36, 106)	3.90(3.73, 4.07)	4.45(4.26, 4.63)	4.45(4.26, 4.63)	2.77(2.37, 3.17)
Gout	4, 360(2, 476-7, 022)	21, 718(13, 944-32, 843)	5.48(5.36, 5.60)	4.01(3.84, 4.17)	7.15(6.89, 7.41)	5.37(5.16, 5.57)
Gynecological diseases	7, 785(4, 434-12, 432)	59, 558(15, 943-127, 897)	3.35(3.21, 3.49)	3.83(3.75, 3.91)	2.36(2.03, 2.69)	3.66(3.43, 3.90)
Headache disorders	15, 349(4, 217-32, 563)	33, 015(25, 585-42, 333)	4.50(4.33, 4.67)	4.35(3.85, 4.85)	5.09(5.04, 5.15)	3.87(3.73, 4.01)
Hemoglobinopathies and hemolytic anemias	16, 118(11, 993-20, 901)	9, 370(4, 804-16, 503)	2.33(2.19, 2.47)	2.68(2.45, 2.90)	2.17(2.00, 2.33)	2.21(1.96, 2.47)

HIV/AIDS	255(54-1, 258)	2, 474(1, 408-3, 244)	11.13(8.78, 13.53)	16.95(10.27, 24.03)	2.56(1.37, 3.78)	14.70(12.28, 17.17)
Hodgkin lymphoma	1, 955(804-2, 659)	846, 786(583, 783-1, 093, 068)	0.76(0.43, 1.09)	1.46(0.80, 2.11)	-0.96(-1.41, -0.50)	2.05(1.60, 2.50)
Hypertensive heart disease	460, 375(338, 270-540, 316)	801(271-1, 610)	1.98(1.68, 2.28)	0.48(0.02, 0.94)	2.25(1.85, 2.66)	3.31(2.79, 3.84)
Idiopathic developmental intellectual disability	321(124-625)	22, 140(13, 026-34, 310)	3.06(2.78, 3.33)	5.30(4.50, 6.11)	1.91(1.70, 2.11)	2.13(1.99, 2.26)
Idiopathic epilepsy	7, 260(4, 713-10, 746)	11, 986(9, 127-16, 785)	3.76(3.53, 3.99)	4.46(4.08, 4.84)	2.59(2.47, 2.71)	4.56(3.91, 5.23)
Inflammatory bowel disease	6, 819(3, 822-9, 143)	4, 536(3, 572-5, 792)	1.94(1.76, 2.11)	3.47(3.27, 3.67)	0.44(0.21, 0.68)	2.06(1.69, 2.43)
Inguinal, femoral, and abdominal hernia	2, 153(1, 665-2, 781)	13, 753(10, 763-17, 411)	2.36(1.75, 2.98)	2.97(2.79, 3.14)	2.64(2.10, 3.17)	1.63(0.33, 2.94)
Interpersonal violence	6, 702(5, 304-8, 299)	17, 125(11, 027-22, 739)	2.36(2.29, 2.44)	2.82(2.67, 2.98)	1.90(1.79, 2.00)	2.45(2.38, 2.52)
Interstitial lung disease and pulmonary sarcoidosis	3, 815(2, 915-6, 054)	254(140-412)	5.02(4.80, 5.24)	4.04(3.82, 4.26)	8.20(7.75, 8.66)	2.82(2.44, 3.21)
Intestinal nematode infections	2, 915(1, 563-4, 603)	258(61-665)	-7.59(-8.01, -7.15)	-8.25(-8.70, -7.80)	-11.36(-12.19, -10.52)	-1.81(-2.71, -0.91)
Invasive Non-typhoidal Salmonella (iNTS)	112(29-279)	2, 962(1, 369-5, 464)	2.83(2.61, 3.05)	3.48(3.12, 3.84)	2.49(2.23, 2.75)	2.56(2.14, 2.97)
Iodine deficiency	750(407-1, 261)	4, 520, 444(3, 806, 634-5, 212, 453)	4.55(4.19, 4.91)	3.98(3.69, 4.28)	6.58(5.56, 7.61)	3.09(2.70, 3.48)
Ischemic heart disease	916, 577(805, 690-1, 023, 069)	31, 226(25, 449-36, 976)	5.39(4.89, 5.89)	5.59(4.28, 6.93)	7.52(7.06, 7.99)	2.90(2.38, 3.42)
Kidney cancer	6, 421(5, 606-7, 205)	25, 164(20, 532-30, 169)	5.45(5.21, 5.68)	6.27(6.00, 6.54)	6.27(6.00, 6.54)	3.75(3.23, 4.27)
Larynx cancer	9, 109(7, 422-10, 627)	46(10-117)	3.29(2.72, 3.87)	2.74(2.49, 2.99)	4.09(2.37, 5.83)	3.11(2.76, 3.46)
Leishmaniasis	32(3-85)	15(9-24)	1.24(0.67, 1.82)	-1.14(-1.36, -0.92)	1.67(0.30, 3.05)	3.86(3.44, 4.28)
Leprosy	14(8-21)	41, 874(31, 944-54, 451)	0.41(0.13, 0.69)	1.00(0.52, 1.47)	-1.09(-1.63, -0.54)	1.60(1.28, 1.93)
Leukemia	13, 940(11, 128-16, 873)	28, 085(23, 018-33, 397)	3.68(3.46, 3.91)	4.57(4.21, 4.92)	3.69(3.51, 3.88)	2.62(2.09, 3.14)
Lip and oral cavity cancer	6, 931(6, 010-7, 843)	166, 427(137, 563-203, 235)	4.73(4.11, 5.37)	4.64(3.36, 5.94)	5.85(4.76, 6.94)	3.94(3.39, 4.49)
Liver cancer	36, 021(30, 541-41, 979)	359, 020(234, 189-524, 566)	5.56(4.71, 6.42)	6.19(5.19, 7.20)	7.90(6.68, 9.13)	1.85(0.41, 3.31)
Low back pain	120, 352(78, 934-176, 224)	22, 476(12, 827-38, 041)	3.58(3.43, 3.73)	2.89(2.48, 3.31)	4.24(4.17, 4.31)	3.50(3.33, 3.68)
Lower extremity peripheral arterial disease	6, 296(3, 329-10, 831)	481, 951(388, 776-589, 077)	4.20(4.09, 4.32)	4.74(4.50, 4.98)	4.23(4.12, 4.34)	3.58(3.41, 3.75)

Lower respiratory infections	325, 675(251, 712-372, 600)	20, 112(12, 269-26, 978)	1.23(0.91, 1.55)	1.85(1.17, 2.53)	0.32(-0.06, 0.71)	1.72(1.40, 2.03)
Malaria	49(11-332)	6, 496(3, 311-8, 477)	-15.70(-18.54, -12.75)	-10.97(-13.59, -8.26)	-12.30(-15.39, -9.09)	-24.32(-29.54, -18.70)
Malignant neoplasm of bone and articular cartilage	2, 937(1, 830-5, 332)	6, 311(5, 273-7, 372)	6.68(6.23, 7.12)	8.80(7.86, 9.75)	8.12(7.59, 8.66)	2.30(1.76, 2.85)
Malignant skin melanoma	1, 721(1, 242-2, 518)	3, 049(2, 467-3, 629)	4.17(3.99, 4.34)	4.17(3.99, 4.34)	4.17(3.99, 4.34)	4.17(3.99, 4.34)
Meningitis	5, 837(5, 026-6, 730)	1, 278(852-1, 651)	0.21(-0.32, 0.74)	-1.41(-2.20, -0.60)	0.58(-0.10, 1.26)	1.82(0.91, 2.75)
Mesothelioma	695(556-797)	13, 037(8, 164-16, 966)	4.88(4.73, 5.04)	3.85(3.69, 4.02)	8.44(7.98, 8.89)	2.56(2.45, 2.66)
Motor neuron disease	206(138-268)	208(158-280)	6.03(5.68, 6.39)	7.52(6.95, 8.10)	4.50(4.00, 5.01)	6.61(6.03, 7.18)
Multiple myeloma	1, 008(631-2, 234)	20, 009(16, 510-23, 714)	9.16(8.33, 10.00)	16.94(14.91, 19.00)	5.31(4.43, 6.19)	6.22(5.88, 6.56)
Multiple sclerosis	36(24-51)	103, 412(56, 188-176, 207)	5.77(5.57, 5.97)	6.64(6.57, 6.72)	5.65(5.31, 5.99)	5.28(4.93, 5.64)
Nasopharynx cancer	12, 634(10, 705-14, 489)	2, 907(2, 081-3, 950)	1.51(1.16, 1.86)	2.28(1.62, 2.94)	0.10(-0.38, 0.59)	2.70(2.44, 2.97)
Neck pain	27, 083(14, 131-46, 723)	720(512-894)	4.33(4.06, 4.59)	3.55(3.37, 3.73)	5.46(4.79, 6.13)	3.93(3.54, 4.32)
Neonatal disorders	672(441-983)	41, 896(33, 590-50, 771)	4.78(4.53, 5.02)	3.38(3.18, 3.59)	5.64(5.00, 6.29)	5.27(4.95, 5.58)
Neuroblastoma and other peripheral nervous cell tumors	50(30-81)	28, 579(21, 308-34, 754)	8.81(8.28, 9.35)	7.23(6.59, 7.87)	11.86(10.88, 12.84)	7.49(6.38, 8.61)
Non-Hodgkin lymphoma	11, 468(9, 793-14, 355)	12, 401(8, 999-17, 944)	4.38(3.93, 4.84)	4.99(4.40, 5.59)	4.95(3.72, 6.19)	3.36(3.11, 3.60)
Non-melanoma skin cancer	5, 722(4, 699-7, 227)	242, 784(155, 354-345, 981)	5.34(5.07, 5.61)	5.86(5.15, 6.58)	7.22(6.97, 7.47)	2.80(2.52, 3.08)
Non-rheumatic valvular heart disease	3, 818(2, 821-5, 357)	263, 167(127, 426-528, 567)	3.89(3.72, 4.06)	4.56(4.25, 4.86)	3.91(3.81, 4.00)	3.13(2.69, 3.56)
Oral disorders	66, 015(41, 784-95, 726)	44, 740(32, 770-63, 136)	4.45(3.94, 4.96)	3.21(2.51, 3.91)	5.13(4.49, 5.78)	5.27(4.41, 6.13)
Osteoarthritis	60, 950(29, 464-122, 536)	8, 903(6, 207-11, 430)	4.84(4.61, 5.08)	4.50(3.91, 5.08)	4.90(4.80, 5.00)	5.16(4.70, 5.62)
Other cardiovascular and circulatory diseases	14, 643(10, 810-20, 802)	27, 815(22, 437-34, 067)	3.70(3.52, 3.87)	3.87(3.59, 4.15)	3.67(3.40, 3.94)	3.54(3.26, 3.82)
Other chronic respiratory diseases	1, 691(1, 263-2, 984)	80(3-209)	5.77(5.30, 6.23)	7.16(6.10, 8.24)	9.60(8.93, 10.27)	0.84(0.50, 1.19)
Other digestive diseases	22, 334(14, 884-28, 203)	51, 494(40, 837-63, 038)	0.72(0.56, 0.88)	-0.34(-0.71, 0.03)	-0.40(-0.54, -0.25)	3.29(3.13, 3.46)
Other intestinal infectious diseases	56(1-156)	38, 970(25, 387-56, 898)	1.26(1.16, 1.35)	1.26(1.16, 1.35)	1.26(1.16, 1.35)	1.26(1.16, 1.35)

Other malignant neoplasms	18, 997(12, 949-23, 437)	95, 495(44, 593-179, 283)	3.16(2.66, 3.66)	2.12(1.92, 2.32)	4.35(2.97, 5.74)	3.02(2.39, 3.65)
Other mental disorders	10, 327(6, 742-15, 053)	2, 632(1, 982-3, 510)	4.39(4.32, 4.47)	4.50(4.35, 4.64)	4.65(4.58, 4.73)	3.96(3.84, 4.08)
Other musculoskeletal disorders	27, 992(14, 049-49, 249)	10, 562(7, 029-18, 542)	4.08(3.71, 4.46)	4.46(3.40, 5.53)	5.63(5.24, 6.02)	1.95(1.73, 2.17)
Other neglected tropical diseases	1, 409(995-1, 859)	16, 581(12, 653-20, 706)	1.97(1.71, 2.23)	2.75(2.59, 2.90)	1.46(1.28, 1.64)	1.69(0.87, 2.52)
Other neoplasms	1, 784(1, 127-3, 067)	6, 905(5, 387-8, 726)	5.95(5.83, 6.07)	5.94(5.82, 6.07)	7.98(7.73, 8.22)	3.86(3.63, 4.10)
Other neurological disorders	1, 490(1, 018-2, 002)	5, 811(4, 685-6, 978)	8.14(7.69, 8.60)	8.55(7.50, 9.62)	7.68(7.46, 7.90)	8.30(7.35, 9.27)
Other nutritional deficiencies	1, 831(1, 473-2, 248)	40, 874(20, 251-78, 831)	4.51(3.64, 5.39)	2.41(1.08, 3.77)	7.91(6.60, 9.23)	1.83(0.68, 3.00)
Other pharynx cancer	2, 250(1, 920-2, 711)	26, 926(14, 052-48, 110)	3.15(2.72, 3.58)	2.85(1.95, 3.77)	2.54(2.10, 2.99)	4.60(4.34, 4.86)
Other sense organ diseases	10, 662(5, 294-20, 525)	7, 012(5, 596-8, 626)	4.47(4.40, 4.54)	4.57(4.44, 4.70)	4.71(4.62, 4.80)	4.06(3.95, 4.18)
Other skin and subcutaneous diseases	5, 982(3, 137-10, 342)	14, 107(10, 258-19, 179)	5.01(4.95, 5.07)	4.82(4.74, 4.90)	5.52(5.46, 5.57)	4.58(4.45, 4.72)
Other transport injuries	3, 287(2, 679-3, 922)	5, 622(3, 585-7, 754)	2.65(2.08, 3.22)	4.20(3.74, 4.66)	1.97(1.45, 2.49)	1.86(0.62, 3.12)
Other unintentional injuries	6, 240(4, 734-8, 204)	2, 550(1, 176-4, 767)	2.63(2.26, 3.01)	2.88(2.22, 3.54)	3.07(2.13, 4.02)	1.87(1.77, 1.97)
Other unspecified infectious diseases	3, 329(1, 895-4, 422)	15, 665(11, 700-20, 129)	1.65(1.34, 1.96)	1.85(1.41, 2.29)	-0.33(-0.62, -0.04)	3.45(2.65, 4.26)
Otitis media	944(432-1, 710)	139, 137(112, 178-164, 217)	3.27(3.19, 3.35)	3.35(3.19, 3.52)	3.39(3.31, 3.46)	3.01(2.87, 3.16)
Ovarian cancer	5, 158(4, 033-6, 672)	18, 601(14, 208-24, 307)	3.74(2.84, 4.64)	5.83(4.70, 6.97)	2.61(2.18, 3.03)	3.62(1.71, 5.56)
Pancreatic cancer	27, 481(23, 396-32, 317)	36, 474(28, 636-48, 169)	5.45(5.32, 5.59)	5.99(5.84, 6.14)	5.99(5.84, 6.14)	4.33(4.04, 4.62)
Pancreatitis	6, 623(4, 881-8, 889)	362, 702(304, 412-423, 861)	3.40(3.20, 3.60)	3.33(3.02, 3.64)	4.17(3.91, 4.43)	2.56(2.09, 3.03)
Paralytic ileus and intestinal obstruction	20, 804(16, 565-26, 875)	15, 931(12, 529-19, 754)	1.89(1.72, 2.07)	2.57(2.46, 2.68)	2.30(2.18, 2.42)	0.69(0.22, 1.17)
Parkinson's disease	95, 312(81, 654-107, 497)	18, 824(10, 716-23, 934)	4.43(4.23, 4.64)	4.59(4.49, 4.68)	4.59(4.49, 4.68)	4.05(3.41, 4.70)
Pneumoconiosis	6, 676(5, 450-8, 112)	396(132-714)	2.93(2.75, 3.11)	3.54(3.23, 3.85)	2.83(2.65, 3.00)	2.35(2.13, 2.57)
Poisonings	4, 725(3, 501-9, 248)	97, 548(65, 167-132, 145)	4.81(4.14, 5.48)	4.63(3.57, 5.70)	8.03(6.92, 9.16)	0.53(-0.09, 1.14)
Police conflict and executions	76(15-123)	23, 459(19, 273-28, 712)	5.18(4.34, 6.03)	1.70(1.16, 2.24)	4.50(2.02, 7.03)	9.86(9.58, 10.15)

Prostate cancer	21, 321(16, 532-31, 876)	5, 810(2, 536-12, 105)	5.14(4.36, 5.93)	7.07(5.92, 8.23)	3.85(3.53, 4.17)	4.75(2.46, 7.09)
Protein-energy malnutrition	23, 203(19, 870-26, 514)	14, 571(10, 664-18, 927)	-0.03(-0.41, 0.34)	-2.61(-3.20, -2.02)	-0.09(-0.39, 0.21)	2.99(2.09, 3.90)
Pruritus	1, 374(602-2, 844)	16, 175(10, 777-20, 120)	4.79(4.71, 4.87)	4.85(4.71, 5.00)	5.11(5.01, 5.21)	4.33(4.21, 4.46)
Psoriasis	2, 995(2, 196-3, 902)	136(68-218)	5.31(5.19, 5.43)	5.91(5.72, 6.11)	5.38(5.20, 5.56)	4.57(4.39, 4.74)
Pulmonary Arterial Hypertension	5, 741(3, 937-8, 688)	154, 935(120, 741-197, 039)	3.36(2.88, 3.85)	2.18(1.98, 2.39)	7.63(6.11, 9.16)	0.53(0.34, 0.72)
Rabies	51(24-81)	40, 829(31, 656-49, 883)	2.21(-0.50, 4.99)	1.33(-3.50, 6.40)	11.89(5.51, 18.67)	-8.26(-9.42, -7.09)
Rheumatic heart disease	148, 957(123, 900-180, 826)	146, 111(125, 126-171, 695)	0.09(0.01, 0.17)	0.09(0.01, 0.17)	0.09(0.01, 0.17)	0.09(0.01, 0.17)
Rheumatoid arthritis	10, 183(8, 371-12, 469)	21, 986(11, 570-36, 101)	4.62(4.35, 4.90)	4.74(4.21, 5.27)	5.19(4.94, 5.44)	3.67(3.20, 4.13)
Road injuries	40, 155(34, 767-47, 179)	1, 410(965-2, 146)	4.36(4.05, 4.68)	6.25(5.57, 6.94)	5.18(4.68, 5.68)	1.35(1.07, 1.63)
Scabies	5, 886(3, 100-9, 665)	18, 105(13, 272-22, 537)	4.36(4.29, 4.43)	4.50(4.36, 4.64)	4.61(4.54, 4.69)	3.90(3.78, 4.02)
Schistosomiasis	2, 003(1, 470-2, 702)	130, 191(107, 356-155, 095)	-1.16(-1.60, -0.72)	-1.72(-1.89, -1.54)	-2.46(-3.65, -1.26)	0.95(0.49, 1.41)
Schizophrenia	4, 569(3, 333-5, 849)	1, 440(822-2, 434)	4.57(4.49, 4.65)	4.47(4.32, 4.63)	4.86(4.78, 4.94)	4.35(4.23, 4.47)
Self-harm	81, 652(53, 691-92, 425)	7, 391(5, 311-10, 333)	1.49(1.29, 1.70)	3.69(3.46, 3.92)	-0.20(-0.43, 0.04)	1.11(0.62, 1.59)
Sexually transmitted infections excluding HIV	673(436-1, 015)	601, 036(458, 253-723, 571)	2.47(1.95, 3.01)	1.23(-0.16, 2.63)	3.43(3.22, 3.63)	2.61(1.76, 3.46)
Soft tissue and other extraosseous sarcomas	2, 882(1, 973-3, 766)	6, 247, 952(5, 260, 685-7, 143, 848)	3.12(2.89, 3.34)	3.73(3.45, 4.01)	2.60(2.24, 2.96)	3.06(2.65, 3.47)
Stomach cancer	252, 767(217, 035-311, 737)	1, 455(1, 110-1, 834)	2.94(2.57, 3.32)	3.70(2.97, 4.45)	3.01(2.55, 3.48)	1.84(1.63, 2.04)
Stroke	2, 531, 359(2, 234, 766-2, 832, 829)	224(64-578)	3.06(2.56, 3.57)	4.18(3.96, 4.39)	2.93(1.42, 4.47)	1.98(1.75, 2.21)
Testicular cancer	432(346-516)	13, 081(9, 930-15, 744)	4.29(3.53, 5.05)	6.22(4.34, 8.15)	1.46(0.76, 2.17)	5.64(4.84, 6.43)
Tetanus	1, 382(524-2, 125)	1, 090, 998(894, 973-1, 306, 700)	-5.70(-6.44, -4.96)	-5.75(-7.32, -4.15)	-5.50(-6.34, -4.65)	-6.11(-6.49, -5.74)
Thyroid cancer	3, 732(3, 177-4, 676)	192(89-343)	4.25(3.74, 4.76)	4.24(3.27, 5.22)	5.18(4.40, 5.97)	3.24(2.69, 3.79)
Tracheal, bronchus, and lung cancer	185, 494(160, 537-211, 426)	70, 860(57, 774-89, 498)	6.06(5.90, 6.21)	7.18(7.00, 7.36)	7.18(7.00, 7.36)	3.75(3.42, 4.07)
Trachoma	463(252-760)	54(22-105)	-2.85(-3.37, -2.33)	0.13(-0.81, 1.07)	-6.12(-7.29, -4.94)	-3.02(-3.33, -2.70)

Tuberculosis	125, 575(105, 259-149, 255)	152, 827(126, 713-184, 149)	-1.85(-2.28, -1.41)	-1.63(-1.98, -1.28)	-2.90(-4.08, -1.71)	-1.25(-1.47, -1.03)
Typhoid and paratyphoid	37(13-78)	8, 768(5, 297-15, 690)	1.26(0.79, 1.74)	0.30(0.08, 0.52)	2.55(1.51, 3.59)	0.92(-0.14, 2.00)
Upper digestive system diseases	99, 247(85, 178-115, 653)	81, 268(57, 078-109, 353)	1.50(0.95, 2.06)	1.06(0.63, 1.49)	1.86(0.34, 3.41)	1.09(0.88, 1.31)
Upper respiratory infections	26, 300(5, 076-40, 526)	5, 686(3, 432-8, 582)	-3.53(-3.92, -3.13)	-0.43(-0.73, -0.13)	-8.88(-9.50, -8.24)	0.27(-0.40, 0.95)
Urinary diseases and male infertility	27, 730(19, 816-35, 918)	12, 268(8, 740-17, 137)	3.61(3.21, 4.01)	3.51(2.96, 4.06)	3.48(2.42, 4.54)	3.72(3.57, 3.87)
Urticaria	1, 574(938-2, 375)	3, 428(2, 287-5, 094)	4.25(4.16, 4.35)	4.37(4.19, 4.55)	4.47(4.37, 4.57)	3.84(3.68, 4.00)
Uterine cancer	5, 985(4, 421-7, 651)	2, 069(1, 655-2, 470)	2.35(1.99, 2.71)	4.45(4.19, 4.70)	2.13(1.81, 2.46)	1.13(0.33, 1.93)
Varicella and herpes zoster	3, 567(2, 747-4, 479)	6, 605(4, 259-9, 768)	-0.10(-0.29, 0.09)	-0.13(-0.35, 0.09)	-2.12(-2.56, -1.68)	1.99(1.75, 2.23)
Vascular intestinal disorders	1, 112(726-1, 352)	282(157-449)	2.09(1.85, 2.33)	3.86(3.51, 4.20)	0.85(0.46, 1.24)	1.45(1.14, 1.76)
Viral skin diseases	1, 721(1, 113-2, 547)	6, 605(4, 259, 9, 768)	4.45(4.38, 4.52)	4.53(4.39, 4.67)	4.71(4.64, 4.79)	4.04(3.93, 4.15)
Vitamin A deficiency	80(45-129)	282(157, 449)	4.28(4.12, 4.43)	5.09(4.74, 5.43)	4.34(4.21, 4.46)	3.30(2.99, 3.61)
**85-89 years**						
Acne vulgaris	44(28-68)	797(500-1, 242)	6.22(5.97, 6.47)	5.89(5.70, 6.08)	5.95(5.21, 6.69)	6.86(6.79, 6.94)
Acute glomerulonephritis	2, 290(1, 670-3, 168)	8, 061(5, 319-10, 648)	2.87(1.66, 4.09)	2.80(0.32, 5.35)	3.25(2.84, 3.67)	1.91(0.75, 3.09)
Acute hepatitis	2, 919(2, 371-3, 557)	2, 869(2, 200-3, 634)	-2.07(-2.57, -1.57)	-1.38(-1.79, -0.97)	-6.09(-7.06, -5.11)	1.21(0.16, 2.26)
Adverse effects of medical treatment	1, 635(1, 014-2, 146)	4, 109(3, 257-5, 564)	1.76(0.96, 2.57)	2.00(1.64, 2.37)	-0.55(-2.74, 1.70)	4.01(3.35, 4.67)
Age-related and other hearing loss	74, 354(53, 762-98, 786)	804, 036(580, 516-1, 071, 028)	5.85(5.52, 6.18)	5.08(4.08, 6.09)	5.95(5.85, 6.05)	6.57(6.50, 6.64)
Alcohol use disorders	1, 313(932-1, 801)	20, 110(14, 088-27, 308)	6.61(6.46, 6.76)	7.16(7.05, 7.27)	7.16(7.05, 7.27)	5.27(4.86, 5.69)
Alopecia areata	60(40-87)	878(570-1, 278)	5.60(5.46, 5.73)	5.32(5.12, 5.51)	5.27(4.91, 5.64)	6.23(6.15, 6.31)
Alzheimer's disease and other dementias	300, 744(127, 852-677, 727)	2, 125, 107(982, 062-4, 558, 620)	5.78(5.59, 5.97)	5.42(5.18, 5.67)	5.07(4.70, 5.44)	6.93(6.54, 7.32)
Animal contact	687(548-817)	2, 426(1, 966-2, 938)	1.90(1.52, 2.29)	2.92(2.74, 3.10)	-0.70(-1.77, 0.37)	3.84(3.53, 4.15)
Anxiety disorders	8, 253(5, 420-11, 713)	102, 402(70, 598-143, 531)	5.74(5.42, 6.06)	6.37(5.98, 6.77)	4.45(4.24, 4.67)	6.56(5.61, 7.51)

Aortic aneurysm	820(658-990)	10, 599(8, 511-12, 956)	7.13(6.60, 7.66)	6.82(6.54, 7.09)	7.79(6.23, 9.38)	6.82(6.54, 7.11)
Appendicitis	1, 592(1, 036-2, 027)	3, 540(2, 654-4, 735)	1.14(0.82, 1.47)	2.57(1.95, 3.20)	-1.31(-1.79, -0.83)	2.51(2.03, 2.99)
Asthma	31, 183(23, 292-46, 760)	88, 826(71, 576-106, 272)	1.94(1.36, 2.52)	3.16(2.33, 4.00)	0.05(-1.30, 1.42)	2.28(1.92, 2.64)
Atrial fibrillation and flutter	42, 683(34, 278-52, 933)	251, 218(201, 037-311, 428)	5.37(4.86, 5.89)	5.81(5.52, 6.11)	4.21(2.78, 5.65)	6.21(5.78, 6.65)
Autism spectrum disorders	474(314-689)	12, 080(8, 256-16, 961)	7.32(7.06, 7.58)	6.71(6.57, 6.84)	7.67(6.96, 8.39)	7.61(7.26, 7.96)
Bacterial skin diseases	6, 061(4, 377-7, 460)	4, 668(3, 132-8, 088)	-0.77(-1.19, -0.34)	1.62(1.14, 2.11)	-8.30(-9.01, -7.59)	6.29(5.24, 7.35)
Bipolar disorder	541(329-802)	7, 027(4, 477-10, 197)	5.70(5.36, 6.04)	5.42(4.71, 6.15)	5.36(4.61, 6.11)	6.41(6.33, 6.49)
Bladder cancer	13, 250(9, 909-15, 136)	96, 249(77, 626-118, 707)	5.11(4.72, 5.50)	5.95(5.25, 6.65)	2.86(2.03, 3.70)	6.47(6.08, 6.86)
Blindness and vision loss	46, 917(34, 103-65, 921)	372, 809(261, 826-534, 161)	4.90(4.41, 5.39)	6.02(4.65, 7.42)	3.77(3.25, 4.30)	5.12(4.92, 5.32)
Brain and central nervous system cancer	3, 367(2, 482-4, 122)	52, 968(36, 842-66, 688)	6.73(6.21, 7.26)	7.10(6.04, 8.18)	6.15(5.51, 6.80)	6.63(6.50, 6.76)
Breast cancer	6, 748(5, 436-8, 051)	64, 391(50, 987-78, 644)	5.32(4.93, 5.72)	6.36(5.42, 7.30)	3.70(3.24, 4.15)	6.37(5.69, 7.05)
Cardiomyopathy and myocarditis	5, 598(4, 438-8, 956)	50, 040(37, 761-60, 810)	6.93(6.28, 7.59)	10.41(8.97, 11.86)	5.07(3.69, 6.47)	4.92(4.75, 5.09)
Cervical cancer	4, 067(3, 276-4, 935)	35, 203(26, 509-44, 071)	4.54(4.02, 5.06)	4.59(3.67, 5.52)	3.62(2.80, 4.44)	5.41(4.84, 5.98)
Chronic kidney disease	63, 395(54, 361-74, 539)	478, 496(392, 779-558, 749)	5.44(4.98, 5.91)	5.43(4.83, 6.03)	4.91(3.73, 6.12)	5.75(5.60, 5.89)
Chronic obstructive pulmonary disease	1, 312, 275(1, 120, 026-1, 451, 099)	3, 873, 601(3, 199, 744-4, 478, 223)	2.52(2.14, 2.91)	4.02(3.72, 4.31)	0.19(-0.76, 1.14)	3.48(2.96, 4.00)
Cirrhosis and other chronic liver diseases	28, 764(23, 707-33, 895)	155, 024(123, 245-189, 220)	3.34(2.89, 3.79)	3.72(2.95, 4.50)	2.17(1.60, 2.75)	4.06(3.56, 4.56)
Colon and rectum cancer	38, 753(33, 245-44, 421)	412, 968(334, 908-480, 639)	5.86(5.21, 6.50)	6.18(5.79, 6.58)	4.83(2.96, 6.73)	6.53(6.29, 6.78)
Conflict and terrorism	0(0-0)	18(12-26)	9.05(5.08, 13.17)	19.52(14.98, 24.25)	4.15(-6.12, 15.53)	2.66(-0.01, 5.39)
Congenital birth defects	326(210-480)	4, 495(3, 071-6, 488)	5.98(5.70, 6.26)	7.02(6.23, 7.82)	5.36(5.04, 5.67)	5.52(5.39, 5.65)
COVID-19	NA	2, 053(226-7, 204)	NA	NA	NA	NA
Cystic echinococcosis	58(35-86)	178(119-255)	1.60(1.10, 2.09)	2.03(1.23, 2.84)	0.54(-0.06, 1.15)	2.22(1.54, 2.90)
Cysticercosis	1, 432(731-2, 486)	8, 139(3, 556-15, 025)	3.33(3.03, 3.63)	3.16(2.86, 3.46)	0.98(0.53, 1.42)	6.26(5.45, 7.08)

Decubitus ulcer	623(345-1, 560)	8, 103(4, 427-10, 561)	10.56(9.59, 11.55)	20.47(18.88, 22.09)	7.69(6.22, 9.18)	3.82(2.19, 5.48)
Dengue	4(2-6)	9(4-16)	2.71(1.01, 4.44)	-2.43(-6.89, 2.24)	5.88(4.96, 6.81)	3.71(3.28, 4.15)
Depressive disorders	14, 569(10, 390-19, 796)	187, 798(129, 913-261, 602)	5.90(5.71, 6.09)	6.03(5.74, 6.32)	5.78(5.47, 6.10)	5.93(5.55, 6.30)
Dermatitis	1, 490(880-2, 414)	19, 480(10, 562-34, 814)	5.67(5.45, 5.88)	5.35(5.19, 5.51)	5.36(4.72, 6.00)	6.33(6.26, 6.39)
Diabetes mellitus	42, 364(36, 591-49, 116)	558, 817(467, 960-663, 442)	6.32(5.92, 6.73)	7.55(7.31, 7.79)	5.30(4.22, 6.38)	5.96(5.41, 6.51)
Diarrheal diseases	12, 574(5, 685-19, 157)	8, 114(5, 107-14, 585)	-2.37(-2.90, -1.83)	-3.27(-3.55, -3.00)	-4.55(-5.86, -3.21)	1.23(0.54, 1.93)
Dietary iron deficiency	11, 629(8, 267-15, 590)	59, 428(40, 858-83, 223)	3.47(3.19, 3.75)	5.01(4.22, 5.80)	1.92(1.64, 2.21)	3.79(3.67, 3.91)
Drowning	6, 811(5, 776-7, 821)	44, 422(37, 267-52, 062)	4.77(4.37, 5.17)	5.26(4.52, 6.01)	4.64(4.07, 5.21)	4.03(3.60, 4.46)
Drug use disorders	2, 227(1, 816-2, 645)	8, 928(6, 938-11, 093)	1.90(1.41, 2.39)	5.07(4.44, 5.70)	-3.16(-4.06, -2.26)	5.41(4.66, 6.16)
Encephalitis	265(216-329)	1, 658(1, 338-2, 080)	4.28(3.82, 4.74)	5.03(4.10, 5.97)	3.77(3.31, 4.23)	3.73(3.06, 4.41)
Endocarditis	1, 099(573-1, 458)	2, 896(2, 163-4, 222)	2.24(1.90, 2.58)	4.71(4.38, 5.04)	-3.71(-4.52, -2.90)	6.42(6.01, 6.84)
Endocrine, metabolic, blood, and immune disorders	7, 174(5, 301-9, 292)	65, 933(46, 247-90, 419)	5.58(5.32, 5.84)	5.48(5.15, 5.80)	4.35(3.74, 4.97)	6.75(6.40, 7.11)
Environmental heat and cold exposure	3, 161(1, 190-4, 199)	9, 142(4, 106-12, 833)	2.61(2.01, 3.22)	2.36(2.05, 2.67)	1.06(-0.70, 2.84)	4.47(4.21, 4.74)
Esophageal cancer	42, 023(33, 801-47, 403)	389, 120(315, 900-467, 114)	5.05(4.50, 5.61)	6.37(5.35, 7.41)	3.63(2.99, 4.27)	4.95(4.36, 5.54)
Exposure to forces of nature	298(265-337)	1, 452(1, 142-1, 878)	-0.53(-14.19, 15.30)	-5.80(-12.11, 0.96)	7.47(-30.79, 66.89)	-2.25(-10.58, 6.85)
Exposure to mechanical forces	1, 900(1, 506-2, 653)	21, 583(15, 849-28, 505)	6.21(5.75, 6.67)	6.09(5.21, 6.98)	7.56(7.01, 8.11)	4.38(3.85, 4.91)
Eye cancer	162(87-214)	1, 227(609-1, 651)	5.30(4.67, 5.94)	5.65(4.78, 6.54)	3.87(2.42, 5.33)	6.37(5.67, 7.08)
Falls	62, 997(52, 960-78, 569)	508, 397(382, 226-635, 591)	6.38(5.99, 6.77)	6.30(5.51, 7.10)	4.81(4.20, 5.42)	7.72(7.46, 7.99)
Fire, heat, and hot substances	5, 966(4, 321-6, 944)	23, 493(18, 151-28, 509)	3.19(2.46, 3.92)	2.38(1.83, 2.94)	4.18(2.10, 6.30)	3.21(2.59, 3.84)
Food-borne trematodiases	1, 887(822-3, 587)	12, 959(6, 550-22, 750)	3.56(3.07, 4.04)	1.82(0.98, 2.68)	3.25(2.95, 3.55)	5.13(4.10, 6.17)
Foreign body	2, 030(1, 523-2, 486)	16, 774(10, 836-20, 171)	6.25(5.51, 7.01)	6.39(5.41, 7.37)	5.61(3.67, 7.57)	6.53(6.28, 6.78)
Fungal skin diseases	1, 500(580-3, 054)	13, 025(5, 165-26, 879)	5.65(5.44, 5.87)	5.33(5.17, 5.49)	5.33(4.69, 5.96)	6.34(6.27, 6.40)

Gallbladder and biliary diseases	23, 180(15, 195-29, 427)	125, 932(91, 294-176, 506)	4.25(3.73, 4.78)	4.30(3.98, 4.62)	3.38(1.91, 4.86)	5.22(4.77, 5.67)
Gallbladder and biliary tract cancer	6, 030(4, 272-7, 503)	57, 943(39, 287-72, 323)	5.52(5.16, 5.87)	6.09(5.59, 6.58)	4.60(4.03, 5.17)	6.03(5.45, 6.61)
Gout	1, 368(859-2, 143)	22, 456(12, 698-36, 106)	6.73(6.54, 6.92)	5.02(4.72, 5.33)	7.40(7.28, 7.51)	7.75(7.23, 8.28)
Gynecological diseases	2, 610(1, 627-3, 892)	21, 718(13, 944-32, 843)	4.95(4.78, 5.13)	4.85(4.52, 5.19)	3.86(3.57, 4.15)	6.09(5.96, 6.22)
Headache disorders	4, 335(1, 273-9, 056)	59, 558(15, 943-127, 897)	5.90(5.61, 6.20)	5.52(4.63, 6.42)	5.90(5.81, 5.98)	6.31(6.25, 6.38)
Hemoglobinopathies and hemolytic anemias	5, 619(4, 231-7, 291)	33, 015(25, 585-42, 333)	3.97(3.75, 4.18)	4.22(4.03, 4.42)	3.05(2.52, 3.58)	4.67(4.33, 5.01)
HIV/AIDS	61(13-290)	9, 370(4, 804-16, 503)	11.48(9.14, 13.87)	15.55(9.20, 22.27)	3.21(1.75, 4.69)	16.44(13.90, 19.03)
Hodgkin lymphoma	568(236-780)	2, 474(1, 408-3, 244)	2.38(1.91, 2.84)	3.80(3.03, 4.58)	-1.39(-2.34, -0.43)	4.68(4.01, 5.35)
Hypertensive heart disease	229, 240(176, 333-262, 888)	846, 786(583, 783-1, 093, 068)	3.52(2.81, 4.24)	2.28(1.89, 2.67)	2.79(0.77, 4.84)	5.54(4.90, 6.19)
Idiopathic developmental intellectual disability	89(38-173)	801(271-1, 610)	4.60(4.34, 4.86)	7.00(6.22, 7.78)	2.65(2.46, 2.84)	4.39(4.23, 4.55)
Idiopathic epilepsy	2, 650(1, 743-3, 816)	22, 140(13, 026-34, 310)	5.10(4.84, 5.37)	5.36(5.15, 5.57)	3.30(2.69, 3.91)	6.93(6.43, 7.42)
Inflammatory bowel disease	3, 588(2, 014-4, 730)	11, 986(9, 127-16, 785)	3.27(2.78, 3.77)	5.49(4.50, 6.50)	0.15(-0.42, 0.72)	4.34(3.72, 4.96)
Inguinal, femoral, and abdominal hernia	1, 016(778-1, 433)	4, 536(3, 572-5, 792)	4.18(3.62, 4.75)	5.38(4.34, 6.43)	2.86(2.35, 3.37)	4.07(3.52, 4.63)
Interpersonal violence	1, 931(1, 497-2, 444)	13, 753(10, 763-17, 411)	3.64(3.47, 3.81)	3.82(3.61, 4.03)	2.15(1.80, 2.50)	5.10(4.82, 5.37)
Interstitial lung disease and pulmonary sarcoidosis	1, 294(962-2, 110)	17, 125(11, 027-22, 739)	6.71(6.41, 7.01)	5.39(4.83, 5.96)	9.42(8.81, 10.03)	5.30(5.09, 5.51)
Intestinal nematode infections	805(421-1, 271)	254(140-412)	-6.36(-6.84, -5.89)	-7.38(-7.91, -6.85)	-10.59(-11.52, -9.66)	0.26(-0.68, 1.21)
Invasive Non-typhoidal Salmonella (iNTS)	35(9-85)	258(61-665)	4.06(3.67, 4.46)	4.27(3.51, 5.03)	3.01(2.36, 3.67)	4.64(4.32, 4.95)
Iodine deficiency	212(115-352)	2, 962(1, 369-5, 464)	5.81(5.68, 5.93)	4.45(4.28, 4.62)	7.02(6.76, 7.28)	5.38(5.27, 5.49)
Ischemic heart disease	430, 593(379, 358-481, 660)	4, 520, 444(3, 806, 634-5, 212, 453)	7.02(6.31, 7.75)	7.69(6.30, 9.09)	7.79(6.86, 8.74)	4.83(4.27, 5.39)
Kidney cancer	3, 455(2, 969-3, 945)	31, 226(25, 449-36, 976)	5.81(5.15, 6.47)	6.72(5.58, 7.88)	3.35(1.87, 4.85)	7.31(6.76, 7.86)
Larynx cancer	2, 712(2, 262-3, 144)	25, 164(20, 532-30, 169)	4.99(4.40, 5.59)	5.71(5.16, 6.28)	3.69(2.06, 5.35)	5.51(5.17, 5.84)
Leishmaniasis	9(1-23)	46(10-117)	1.67(1.34, 2.01)	0.04(-0.26, 0.34)	1.37(1.12, 1.62)	3.98(3.02, 4.94)

Leprosy	4(2-6)	15(9-24)	1.24(0.73, 1.74)	0.22(-0.21, 0.64)	0.22(-0.21, 0.64)	3.78(2.28, 5.30)
Leukemia	4, 311(3, 338-5, 218)	41, 874(31, 944-54, 451)	5.14(4.64, 5.64)	6.44(6.10, 6.78)	3.83(2.39, 5.29)	5.03(4.85, 5.21)
Lip and oral cavity cancer	2, 420(2, 085-2, 723)	28, 085(23, 018-33, 397)	5.93(5.11, 6.75)	5.80(5.52, 6.07)	5.80(5.52, 6.07)	6.25(3.54, 9.02)
Liver cancer	15, 055(12, 470-17, 914)	166, 427(137, 563-203, 235)	5.38(4.68, 6.08)	4.23(3.44, 5.02)	6.88(5.88, 7.90)	4.67(3.49, 5.87)
Low back pain	37, 023(24, 356-56, 557)	359, 020(234, 189-524, 566)	5.16(4.98, 5.34)	4.58(4.23, 4.93)	4.94(4.57, 5.31)	5.93(5.88, 5.99)
Lower extremity peripheral arterial disease	2, 299(1, 273-3, 858)	22, 476(12, 827-38, 041)	5.61(5.31, 5.90)	5.85(5.65, 6.05)	5.00(4.14, 5.87)	5.94(5.84, 6.05)
Lower respiratory infections	173, 902(140, 578-198, 012)	481, 951(388, 776-589, 077)	2.94(2.20, 3.68)	4.13(3.73, 4.54)	1.28(-0.86, 3.46)	3.31(2.99, 3.64)
Malaria	8(3-26)	20, 112(12, 269-26, 978)	-8.87(-14.64, -2.72)	-4.11(-4.84, -3.37)	-4.11(-4.84, -3.37)	-13.91(-27.21, 1.81)
Malignant neoplasm of bone and articular cartilage	904(560-1, 614)	6, 496(3, 311-8, 477)	8.08(7.79, 8.38)	10.16(9.57, 10.76)	8.97(8.47, 9.47)	4.41(4.24, 4.58)
Malignant skin melanoma	627(452-896)	0(0-0)	5.93(5.27, 6.60)	6.95(5.69, 8.22)	4.12(2.73, 5.54)	6.64(6.01, 7.28)
Meningitis	2, 192(1, 897-2, 491)	6, 311(5, 273-7, 372)	1.87(1.36, 2.38)	0.65(-0.15, 1.45)	1.69(0.88, 2.52)	3.48(2.81, 4.15)
Mesothelioma	239(188-271)	3, 049(2, 467-3, 629)	6.33(5.97, 6.68)	6.29(6.01, 6.57)	8.36(7.59, 9.13)	4.51(3.83, 5.20)
Motor neuron disease	58(41-77)	1, 278(852-1, 651)	7.51(7.19, 7.83)	9.06(8.51, 9.62)	4.96(4.37, 5.56)	9.25(8.88, 9.63)
Multiple myeloma	296(181-651)	13, 037(8, 164-16, 966)	10.69(9.88, 11.51)	19.43(17.32, 21.58)	5.43(4.39, 6.47)	8.45(8.00, 8.91)
Multiple sclerosis	11(6-15)	208(158-280)	7.16(6.97, 7.35)	8.35(8.11, 8.59)	6.25(6.04, 6.45)	7.06(6.67, 7.46)
Nasopharynx cancer	4, 012(3, 436-4, 650)	20, 009(16, 510-23, 714)	2.51(2.01, 3.00)	4.00(3.09, 4.91)	-0.73(-1.67, 0.22)	4.92(4.39, 5.45)
Neck pain	7, 210(3, 841-12, 250)	103, 412(56, 188-176, 207)	5.78(5.50, 6.06)	5.14(4.53, 5.75)	5.94(5.35, 6.54)	6.38(6.29, 6.48)
Neonatal disorders	197(134-287)	2, 907(2, 081-3, 950)	5.65(5.45, 5.85)	4.39(3.85, 4.94)	5.63(5.55, 5.71)	7.10(6.88, 7.31)
Neuroblastoma and other peripheral nervous cell tumors	18(10-29)	720(512-894)	10.64(10.21, 11.07)	9.67(8.97, 10.38)	11.74(11.27, 12.20)	10.35(9.67, 11.03)
Non-Hodgkin lymphoma	3, 492(2, 973-4, 146)	41, 896(33, 590-50, 771)	5.80(5.19, 6.41)	6.52(5.99, 7.05)	4.62(2.87, 6.40)	6.20(5.90, 6.50)
Non-melanoma skin cancer	2, 536(2, 047-3, 322)	28, 579(21, 308-34, 754)	6.77(6.18, 7.37)	7.21(5.99, 8.44)	6.98(6.37, 7.59)	5.54(5.33, 5.74)
Non-rheumatic valvular heart disease	1, 659(1, 259-2, 217)	12, 401(8, 999-17, 944)	5.32(4.83, 5.81)	5.96(5.69, 6.24)	4.19(2.83, 5.57)	5.86(5.52, 6.20)

Oral disorders	22, 242(14, 183-31, 986)	242, 784(155, 354-345, 981)	5.79(5.31, 6.27)	4.65(4.06, 5.25)	6.17(5.84, 6.49)	7.01(6.00, 8.02)
Osteoarthritis	20, 646(10, 236-41, 460)	263, 167(127, 426-528, 567)	6.17(6.04, 6.31)	5.32(5.05, 5.60)	5.98(5.89, 6.06)	7.39(7.07, 7.71)
Other cardiovascular and circulatory diseases	5, 824(4, 416-7, 944)	44, 740(32, 770-63, 136)	4.96(4.62, 5.30)	5.34(5.03, 5.66)	3.88(2.95, 4.81)	5.63(5.44, 5.81)
Other chronic respiratory diseases	827(583-1, 547)	8, 903(6, 207-11, 430)	7.09(6.87, 7.31)	9.05(8.80, 9.30)	8.96(8.39, 9.53)	3.19(2.99, 3.38)
Other digestive diseases	11, 365(7, 631-14, 616)	27, 815(22, 437-34, 067)	2.20(1.65, 2.76)	1.91(0.47, 3.38)	-0.17(-0.93, 0.59)	5.11(4.79, 5.44)
Other intestinal infectious diseases	49(1-133)	80(3-209)	2.55(1.79, 3.31)	2.99(2.14, 3.84)	1.43(-0.58, 3.49)	2.96(2.65, 3.27)
Other malignant neoplasms	6, 376(4, 419-7, 989)	51, 494(40, 837-63, 038)	4.83(4.52, 5.15)	4.70(4.36, 5.03)	4.63(3.75, 5.52)	5.18(4.94, 5.41)
Other mental disorders	3, 420(2, 252-5, 029)	38, 970(25, 387-56, 898)	5.70(5.48, 5.92)	5.37(5.21, 5.53)	5.37(4.72, 6.02)	6.40(6.33, 6.47)
Other musculoskeletal disorders	7, 062(3, 917-12, 366)	95, 495(44, 593-179, 283)	4.19(3.83, 4.56)	5.00(4.19, 5.82)	6.51(5.87, 7.16)	0.78(0.43, 1.13)
Other neglected tropical diseases	473(349-613)	2, 632(1, 982-3, 510)	3.63(3.46, 3.79)	4.51(4.28, 4.75)	2.55(2.24, 2.85)	3.94(3.65, 4.23)
Other neoplasms	611(364-1, 075)	10, 562(7, 029-18, 542)	7.44(7.16, 7.73)	7.16(6.42, 7.91)	8.34(8.05, 8.63)	6.72(6.48, 6.96)
Other neurological disorders	560(393-758)	16, 581(12, 653-20, 706)	9.25(8.88, 9.62)	9.51(8.74, 10.28)	8.41(8.15, 8.67)	10.09(9.23, 10.96)
Other nutritional deficiencies	1, 075(857-1, 354)	6, 905(5, 387-8, 726)	6.39(5.33, 7.46)	5.69(4.25, 7.15)	7.89(5.22, 10.62)	4.31(3.96, 4.66)
Other pharynx cancer	731(630-843)	5, 811(4, 685-6, 978)	4.57(3.92, 5.22)	5.06(3.93, 6.20)	2.63(1.32, 3.97)	6.63(6.20, 7.07)
Other sense organ diseases	3, 317(1, 653-5, 998)	40, 874(20, 251-78, 831)	5.79(5.55, 6.04)	5.53(5.34, 5.71)	5.45(4.73, 6.17)	6.43(6.36, 6.51)
Other skin and subcutaneous diseases	2, 315(1, 285-3, 970)	26, 926(14, 052-48, 110)	6.11(5.90, 6.32)	5.54(5.09, 5.99)	5.86(5.52, 6.20)	6.89(6.81, 6.96)
Other transport injuries	1, 107(899-1, 319)	7, 012(5, 596-8, 626)	4.17(3.75, 4.58)	5.41(4.39, 6.44)	2.55(2.31, 2.78)	4.34(3.83, 4.86)
Other unintentional injuries	2, 297(1, 770-3, 062)	14, 107(10, 258-19, 179)	3.84(3.57, 4.12)	4.15(3.61, 4.70)	3.39(3.07, 3.72)	3.87(3.53, 4.22)
Other unspecified infectious diseases	1, 326(719-1, 809)	5, 622(3, 585-7, 754)	3.23(2.58, 3.87)	3.37(2.93, 3.82)	0.73(-0.95, 2.43)	5.93(5.33, 6.54)
Otitis media	306(159-557)	2, 550(1, 176-4, 767)	4.76(4.53, 5.00)	4.46(4.30, 4.62)	4.29(3.61, 4.97)	5.62(5.47, 5.77)
Ovarian cancer	1, 516(1, 185-1, 969)	15, 665(11, 700-20, 129)	4.92(4.35, 5.50)	7.10(5.78, 8.44)	1.90(1.32, 2.48)	6.63(5.62, 7.65)
Pancreatic cancer	8, 977(7, 631-10, 372)	139, 137(112, 178-164, 217)	6.95(6.64, 7.25)	7.52(6.85, 8.19)	5.92(5.48, 6.36)	7.51(7.07, 7.96)

Pancreatitis	2, 644(1, 903-3, 609)	18, 601(14, 208-24, 307)	4.88(4.52, 5.24)	4.58(3.94, 5.23)	4.84(4.41, 5.27)	4.97(4.59, 5.34)
Paralytic ileus and intestinal obstruction	10, 998(8, 649-14, 005)	36, 474(28, 636-48, 169)	3.26(2.92, 3.60)	3.21(2.65, 3.77)	2.55(1.98, 3.12)	3.55(3.28, 3.81)
Parkinson's disease	38, 793(32, 303-43, 640)	362, 702(304, 412-423, 861)	5.77(5.29, 6.25)	6.32(5.53, 7.12)	4.62(3.58, 5.67)	6.13(5.93, 6.33)
Pneumoconiosis	2, 192(1, 757-2, 701)	15, 931(12, 529-19, 754)	4.66(4.08, 5.25)	5.91(5.23, 6.59)	3.53(2.01, 5.08)	4.17(3.95, 4.39)
Poisonings	1, 553(1, 126-3, 166)	18, 824(10, 716-23, 934)	6.29(5.73, 6.85)	6.67(5.55, 7.79)	8.42(7.66, 9.18)	2.64(2.15, 3.14)
Police conflict and executions	17(4-32)	396(132-714)	6.11(5.41, 6.80)	4.41(3.01, 5.83)	3.10(1.60, 4.63)	11.34(11.02, 11.67)
Prostate cancer	9, 692(7, 284-13, 677)	97, 548(65, 167-132, 145)	6.37(5.61, 7.15)	9.03(7.81, 10.27)	2.74(1.55, 3.93)	6.89(5.45, 8.35)
Protein-energy malnutrition	18, 445(15, 796-21, 322)	23, 459(19, 273-28, 712)	1.74(1.28, 2.19)	-0.55(-1.54, 0.46)	1.14(0.83, 1.46)	4.96(4.13, 5.79)
Pruritus	444(214-873)	5, 810(2, 536-12, 105)	6.13(5.91, 6.35)	5.79(5.64, 5.95)	5.91(5.31, 6.51)	6.70(6.43, 6.97)
Psoriasis	804(591-1, 049)	14, 571(10, 664-18, 927)	6.61(6.38, 6.85)	6.78(6.30, 7.27)	5.96(5.49, 6.44)	7.00(6.93, 7.07)
Pulmonary Arterial Hypertension	2, 654(1, 836-3, 805)	16, 175(10, 777-20, 120)	4.93(4.36, 5.50)	4.39(4.02, 4.76)	7.72(6.32, 9.13)	2.89(1.91, 3.89)
Rabies	13(6-22)	136(68-218)	4.44(2.20, 6.74)	3.64(-1.24, 8.76)	12.28(10.29, 14.31)	-4.34(-8.02, -0.52)
Rheumatic heart disease	66, 390(54, 000-81, 914)	154, 935(120, 741-197, 039)	1.41(0.55, 2.28)	1.51(0.69, 2.33)	0.29(-2.03, 2.66)	2.19(1.82, 2.56)
Rheumatoid arthritis	3, 770(3, 121-4, 631)	40, 829(31, 656-49, 883)	6.05(5.69, 6.40)	6.11(5.37, 6.86)	5.61(5.16, 6.05)	6.15(5.82, 6.47)
Road injuries	12, 201(10, 572-14, 174)	146, 111(125, 126-171, 695)	5.95(5.62, 6.29)	7.86(7.12, 8.61)	5.58(5.26, 5.91)	3.99(3.56, 4.42)
Scabies	2, 139(1, 132-3, 692)	21, 986(11, 570-36, 101)	5.68(5.45, 5.90)	5.39(5.22, 5.55)	5.34(4.68, 6.00)	6.34(6.27, 6.41)
Schistosomiasis	660(473-891)	1, 410(965-2, 146)	0.11(-0.84, 1.07)	-0.19(-1.08, 0.72)	-1.57(-4.15, 1.08)	1.90(1.47, 2.34)
Schizophrenia	1, 008(739-1, 303)	18, 105(13, 272-22, 537)	6.02(5.74, 6.30)	5.43(5.22, 5.64)	5.78(4.96, 6.60)	6.92(6.83, 7.00)
Self-harm	29, 265(18, 610-33, 370)	130, 191(107, 356-155, 095)	2.99(2.50, 3.48)	5.15(4.19, 6.12)	0.41(-0.06, 0.87)	3.50(2.78, 4.22)
Sexually transmitted infections excluding HIV	240(157-356)	1, 440(822-2, 434)	3.41(2.60, 4.22)	1.66(0.79, 2.55)	3.58(1.78, 5.41)	4.67(4.33, 5.00)
Soft tissue and other extraosseous sarcomas	1, 057(697-1, 388)	7, 391(5, 311-10, 333)	4.72(4.05, 5.39)	6.01(5.63, 6.39)	2.96(1.12, 4.83)	5.29(4.73, 5.85)
Stomach cancer	74, 474(62, 211-93, 726)	601, 036(458, 253-723, 571)	4.40(3.66, 5.14)	5.14(4.29, 5.99)	3.63(1.69, 5.60)	3.95(3.68, 4.23)

Stroke	1, 045, 980(910, 145-1, 165, 877)	6, 247, 952(5, 260, 685-7, 143, 848)	4.40(3.58, 5.24)	5.89(4.85, 6.94)	3.14(0.99, 5.33)	3.86(3.55, 4.17)
Testicular cancer	159(134-186)	1, 455(1, 110-1, 834)	5.46(4.38, 6.55)	8.85(6.97, 10.76)	0.78(-0.61, 2.20)	7.07(4.95, 9.23)
Tetanus	560(185-905)	224(64-578)	-4.46(-6.33, -2.55)	-4.73(-5.82, -3.62)	-5.17(-10.26, 0.21)	-4.06(-5.07, -3.03)
Thyroid cancer	1, 216(1, 024-1, 471)	13, 081(9, 930-15, 744)	5.66(5.12, 6.20)	6.10(5.33, 6.87)	5.03(3.85, 6.22)	5.61(4.85, 6.37)
Tracheal, bronchus, and lung cancer	61, 180(52, 651-69, 973)	1, 090, 998(894, 973-1, 306, 700)	7.20(6.57, 7.83)	8.11(7.30, 8.92)	6.38(4.82, 7.96)	6.78(6.60, 6.95)
Trachoma	150(82-248)	192(89-343)	-0.88(-1.79, 0.03)	1.95(1.23, 2.68)	-5.06(-6.68, -3.41)	0.58(-1.62, 2.83)
Tuberculosis	39, 936(33, 565-47, 089)	70, 860(57, 774-89, 498)	-1.01(-1.60, -0.41)	-0.27(-0.50, -0.04)	-4.02(-5.63, -2.38)	1.72(1.16, 2.28)
Typhoid and paratyphoid	9(3-18)	54(22-105)	2.88(2.12, 3.65)	2.12(0.90, 3.35)	2.74(0.99, 4.52)	3.58(3.20, 3.95)
Upper digestive system diseases	40, 986(34, 496-47, 565)	152, 827(126, 713-184, 149)	2.79(2.33, 3.26)	3.18(2.54, 3.82)	1.99(0.90, 3.08)	2.66(2.39, 2.93)
Upper respiratory infections	17, 831(3, 078-27, 825)	8, 768(5, 297-15, 690)	-3.37(-3.84, -2.90)	1.46(0.32, 2.61)	-11.90(-12.40, -11.39)	2.03(1.11, 2.95)
Urinary diseases and male infertility	10, 696(8, 185-13, 515)	81, 268(57, 078-109, 353)	4.68(4.24, 5.11)	5.69(4.77, 6.62)	2.42(1.96, 2.89)	5.89(5.46, 6.32)
Urticaria	430(264-661)	5, 686(3, 432-8, 582)	5.60(5.44, 5.77)	5.24(5.07, 5.42)	5.31(4.85, 5.77)	6.29(6.21, 6.37)
Uterine cancer	1, 664(1, 215-2, 155)	12, 268(8, 740-17, 137)	3.85(3.23, 4.47)	6.02(5.55, 6.49)	2.58(2.03, 3.14)	3.63(2.28, 5.01)
Varicella and herpes zoster	2, 162(1, 586-2, 875)	3, 428(2, 287-5, 094)	0.22(-0.13, 0.57)	0.38(0.18, 0.58)	-2.82(-3.71, -1.93)	3.69(3.31, 4.08)
Vascular intestinal disorders	615(403-759)	2, 069(1, 655-2, 470)	3.60(3.28, 3.93)	5.57(4.92, 6.23)	1.12(0.70, 1.54)	4.33(3.92, 4.75)
Viral skin diseases	453(294-696)	6, 605(4, 259-9, 768)	5.76(5.54, 5.99)	5.44(5.27, 5.61)	5.39(4.73, 6.05)	6.49(6.43, 6.56)
Vitamin A deficiency	22(12-36)	282(157-449)	5.49(5.34, 5.65)	5.25(5.16, 5.35)	5.25(5.16, 5.35)	6.09(5.61, 6.57)
**90-94 years**						
Acne vulgaris	5(3-8)	61(38-96)	8.17(7.67, 8.68)	8.36(7.16, 9.56)	7.87(7.41, 8.34)	8.45(7.90, 9.00)
Acute glomerulonephritis	463(332-634)	1, 980(1, 339-2, 621)	4.83(4.32, 5.35)	5.14(4.91, 5.38)	6.47(5.05, 7.90)	2.62(2.03, 3.22)
Acute hepatitis	530(429-642)	468(365-582)	-0.45(-0.73, -0.17)	0.17(-0.16, 0.49)	-2.62(-3.06, -2.17)	1.82(1.31, 2.34)
Adverse effects of medical treatment	375(232-499)	1, 115(817-1, 547)	3.55(3.33, 3.76)	4.57(4.15, 4.99)	1.65(1.30, 2.01)	4.85(4.67, 5.04)

Age-related and other hearing loss	14, 556(10, 730-19, 316)	138, 811(102, 174-183, 316)	7.67(7.50, 7.84)	7.57(7.20, 7.93)	7.69(7.49, 7.90)	7.83(7.67, 8.00)
Alcohol use disorders	242(161-364)	2, 793(1, 878-4, 151)	8.27(7.94, 8.60)	8.77(8.10, 9.45)	9.13(8.69, 9.58)	6.67(6.38, 6.97)
Alopecia areata	7(4-10)	67(44-97)	7.60(7.34, 7.85)	8.19(7.62, 8.75)	7.07(6.78, 7.36)	7.65(7.36, 7.94)
Alzheimer's disease and other dementias	87, 039(34, 649-201, 471)	884, 551(382, 093-1, 927, 903)	7.81(7.53, 8.08)	8.23(7.58, 8.89)	7.04(6.76, 7.32)	8.43(8.30, 8.57)
Animal contact	113(89-139)	371(290-459)	3.97(3.73, 4.21)	4.99(4.41, 5.57)	2.30(2.06, 2.54)	5.10(4.71, 5.49)
Anxiety disorders	1, 319(859-1, 860)	12, 795(8, 425-17, 924)	7.66(7.37, 7.94)	9.07(8.62, 9.53)	6.48(6.10, 6.86)	7.74(7.27, 8.21)
Aortic aneurysm	203(165-245)	2, 116(1, 628-2, 684)	7.95(7.60, 8.29)	8.60(8.12, 9.08)	7.58(6.68, 8.49)	7.90(7.76, 8.03)
Appendicitis	455(299-589)	1, 101(798-1, 442)	2.90(2.71, 3.09)	4.73(4.50, 4.96)	0.85(0.48, 1.21)	3.42(3.07, 3.77)
Asthma	6, 958(5, 192-10, 588)	22, 398(17, 129-27, 384)	3.70(3.26, 4.13)	4.74(4.49, 5.00)	2.61(1.33, 3.90)	3.64(3.46, 3.83)
Atrial fibrillation and flutter	17, 273(13, 471-21, 180)	146, 198(114, 618-175, 797)	7.20(6.91, 7.48)	8.23(7.50, 8.97)	6.01(5.77, 6.25)	7.65(7.26, 8.04)
Autism spectrum disorders	55(34-85)	915(587-1, 315)	9.59(9.39, 9.79)	9.58(9.13, 10.03)	9.96(9.72, 10.19)	9.27(9.10, 9.43)
Bacterial skin diseases	2, 176(1, 583-2, 749)	2, 350(1, 296-4, 821)	0.40(-0.14, 0.94)	3.51(2.19, 4.86)	-8.75(-9.47, -8.03)	6.96(6.20, 7.73)
Bipolar disorder	86(51-130)	814(480-1, 207)	7.60(7.44, 7.77)	8.03(7.67, 8.39)	7.16(6.97, 7.34)	7.83(7.65, 8.01)
Bladder cancer	2, 996(2, 217-3, 424)	20, 538(16, 614-25, 608)	6.37(6.02, 6.72)	7.64(6.96, 8.32)	4.76(4.31, 5.21)	7.13(6.64, 7.61)
Blindness and vision loss	10, 290(7, 599-14, 123)	78, 381(56, 775-108, 496)	6.91(6.61, 7.21)	8.09(7.51, 8.67)	5.82(5.35, 6.30)	7.14(6.99, 7.30)
Brain and central nervous system cancer	554(407-683)	6, 455(4, 257-8, 189)	8.38(8.09, 8.66)	9.12(8.60, 9.64)	8.92(8.34, 9.52)	7.27(7.11, 7.44)
Breast cancer	1, 480(1, 208-1, 759)	13, 256(10, 127-16, 040)	7.48(7.01, 7.95)	9.77(8.52, 11.04)	4.51(4.13, 4.89)	8.98(8.39, 9.58)
Cardiomyopathy and myocarditis	1, 992(1, 552-3, 219)	26, 115(17, 786-33, 165)	8.82(7.75, 9.90)	13.39(11.89, 14.90)	8.52(5.65, 11.47)	4.92(4.43, 5.40)
Cervical cancer	649(522-794)	4, 965(3, 566-6, 201)	7.01(6.19, 7.84)	8.64(8.14, 9.13)	5.10(2.73, 7.52)	7.17(6.81, 7.52)
Chronic kidney disease	17, 858(15, 183-21, 251)	143, 210(119, 752-166, 828)	7.01(6.78, 7.24)	7.61(7.17, 8.06)	6.74(6.38, 7.09)	6.70(6.47, 6.92)
Chronic obstructive pulmonary disease	325, 503(270, 872-365, 713)	1, 184, 262(954, 081-1, 387, 658)	4.26(4.04, 4.49)	5.99(5.60, 6.37)	2.63(2.38, 2.88)	4.56(4.11, 5.01)
Cirrhosis and other chronic liver diseases	4, 806(3, 881-5, 739)	22, 911(17, 484-28, 229)	5.08(4.84, 5.32)	5.89(5.57, 6.21)	4.26(3.80, 4.71)	5.17(4.79, 5.55)

Colon and rectum cancer	8, 559(7, 169-9, 741)	69, 147(54, 356-81, 910)	7.05(6.78, 7.31)	7.83(7.24, 8.42)	5.55(5.21, 5.90)	8.19(8.03, 8.35)
Conflict and terrorism	0(0-0)	2(1-3)	4.99(1.15, 8.97)	4.34(3.04, 5.67)	4.34(3.04, 5.67)	6.58(-5.93, 20.77)
Congenital birth defects	53(33-80)	564(361-835)	7.93(7.75, 8.12)	9.26(8.81, 9.71)	7.50(7.32, 7.67)	7.02(6.81, 7.22)
COVID-19	NA	1, 518(137-5, 786)	NA	NA	NA	NA
Cystic echinococcosis	13(6-20)	35(20-52)	3.37(3.07, 3.68)	4.53(3.83, 5.23)	2.48(2.29, 2.68)	3.29(2.67, 3.91)
Cysticercosis	285(140-518)	1, 370(571-2, 795)	5.34(5.03, 5.65)	5.86(5.31, 6.42)	3.15(2.89, 3.40)	7.58(6.85, 8.32)
Decubitus ulcer	303(169-729)	6, 428(4, 110-8, 280)	11.41(10.41, 12.42)	21.38(19.09, 23.71)	7.93(6.51, 9.37)	4.98(3.53, 6.46)
Dengue	0(0-1)	3(1-6)	3.88(1.19, 6.65)	0.00(-1.01, 1.03)	7.16(-0.93, 15.91)	5.01(3.36, 6.68)
Depressive disorders	2, 810(1, 929-3, 975)	27, 383(19, 172-38, 566)	7.70(7.53, 7.87)	8.26(7.91, 8.61)	7.58(7.43, 7.72)	7.39(7.10, 7.67)
Dermatitis	250(138-414)	2, 371(1, 312-3, 883)	7.60(7.50, 7.71)	7.87(7.64, 8.10)	7.32(7.20, 7.44)	7.76(7.65, 7.87)
Diabetes mellitus	8, 210(7, 039-9, 427)	89, 402(75, 007-105, 112)	8.01(7.74, 8.28)	9.92(9.34, 10.51)	6.70(6.38, 7.02)	7.58(7.20, 7.97)
Diarrheal diseases	3, 129(1, 326-5, 058)	2, 723(1, 602-5, 060)	-0.50(-0.78, -0.21)	-1.04(-1.44, -0.64)	-1.77(-2.17, -1.38)	1.86(1.36, 2.36)
Dietary iron deficiency	1, 639(1, 034-2, 422)	8, 285(4, 632-13, 668)	5.41(5.24, 5.58)	6.53(6.19, 6.87)	4.13(3.93, 4.33)	6.05(5.83, 6.27)
Drowning	1, 227(1, 051-1, 433)	8, 734(7, 123-10, 144)	6.63(6.10, 7.16)	7.27(6.78, 7.77)	7.22(5.67, 8.79)	5.34(5.20, 5.47)
Drug use disorders	405(340-472)	1, 277(1, 042-1, 567)	3.73(3.49, 3.97)	6.35(5.96, 6.73)	-0.41(-0.68, -0.13)	6.52(6.00, 7.04)
Encephalitis	71(57-88)	461(355-583)	6.23(5.95, 6.51)	6.51(5.98, 7.04)	7.29(6.91, 7.67)	5.05(4.54, 5.57)
Endocarditis	346(157-486)	1, 107(772-1, 442)	3.91(3.55, 4.28)	7.45(6.93, 7.97)	-0.72(-1.43, 0.00)	6.41(5.97, 6.85)
Endocrine, metabolic, blood, and immune disorders	1, 894(1, 447-2, 452)	16, 548(11, 164-20, 922)	7.32(7.04, 7.61)	7.45(6.89, 8.00)	7.02(6.65, 7.39)	7.80(7.47, 8.14)
Environmental heat and cold exposure	919(334-1, 252)	3, 262(1, 374-4, 428)	4.21(3.87, 4.56)	4.31(3.59, 5.05)	2.99(2.51, 3.46)	5.66(5.38, 5.95)
Esophageal cancer	7, 693(6, 054-8, 743)	53, 466(41, 483-63, 253)	6.51(6.13, 6.90)	8.62(7.96, 9.28)	5.44(5.07, 5.82)	5.79(5.20, 6.38)
Exposure to forces of nature	45(39-52)	238(177-319)	1.70(-10.05, 14.99)	-4.67(-10.59, 1.64)	14.79(-20.42, 65.57)	-3.26(-8.82, 2.64)
Exposure to mechanical forces	394(308-561)	4, 155(2, 960-5, 294)	8.00(7.63, 8.36)	8.17(7.61, 8.74)	10.08(9.53, 10.64)	5.56(4.86, 6.26)

Eye cancer	34(18-44)	298(127-428)	7.08(6.40, 7.76)	6.75(6.51, 6.99)	6.75(6.51, 6.99)	7.88(5.61, 10.20)
Falls	18, 142(15, 431-21, 878)	205, 688(144, 943-253, 930)	8.36(8.06, 8.67)	8.95(8.60, 9.30)	7.01(6.39, 7.64)	9.01(8.48, 9.54)
Fire, heat, and hot substances	1, 450(1, 041-1, 683)	6, 788(5, 063-8, 119)	5.10(4.13, 6.07)	4.58(3.02, 6.16)	6.60(4.58, 8.66)	4.71(4.53, 4.89)
Food-borne trematodiases	296(134-563)	1, 598(848-2, 809)	5.59(5.30, 5.89)	4.55(4.35, 4.74)	5.25(5.02, 5.48)	6.60(5.93, 7.27)
Foreign body	567(423-678)	6, 404(4, 048-7, 823)	8.27(7.95, 8.58)	9.24(8.30, 10.19)	7.81(7.68, 7.94)	7.81(7.68, 7.94)
Fungal skin diseases	335(132-722)	3, 149(1, 236-6, 770)	7.58(7.47, 7.69)	7.85(7.62, 8.09)	7.29(7.17, 7.41)	7.75(7.63, 7.86)
Gallbladder and biliary diseases	6, 041(3, 697-7, 745)	34, 523(27, 010-46, 024)	5.79(5.66, 5.93)	6.19(5.94, 6.44)	4.85(4.63, 5.06)	6.63(6.48, 6.77)
Gallbladder and biliary tract cancer	1, 091(750-1, 338)	8, 979(5, 732-11, 598)	7.07(6.70, 7.45)	7.79(6.94, 8.65)	5.98(5.69, 6.27)	7.46(6.86, 8.06)
Gout	248(147-402)	3, 094(1, 856-5, 011)	8.56(8.23, 8.88)	7.57(6.88, 8.26)	9.14(8.88, 9.41)	9.15(8.77, 9.52)
Gynecological diseases	506(318-800)	4, 319(2, 764-6, 726)	7.25(7.10, 7.39)	7.72(7.39, 8.06)	6.48(6.37, 6.59)	7.56(7.33, 7.79)
Headache disorders	624(156-1, 407)	6, 251(1, 387-14, 077)	7.79(7.65, 7.93)	7.94(7.64, 8.25)	7.81(7.66, 7.96)	7.71(7.57, 7.85)
Hemoglobinopathies and hemolytic anemias	1, 276(924-1, 709)	7, 642(5, 369-10, 534)	5.95(5.76, 6.14)	7.07(6.66, 7.48)	4.85(4.61, 5.10)	6.27(6.17, 6.38)
HIV/AIDS	8(1-40)	793(400-1, 404)	13.59(11.07, 16.17)	18.25(11.16, 25.79)	6.17(4.64, 7.72)	17.40(15.28, 19.56)
Hodgkin lymphoma	71(28-98)	258(141-339)	4.30(3.82, 4.79)	5.44(4.40, 6.49)	2.16(1.51, 2.82)	5.80(5.39, 6.21)
Hypertensive heart disease	60, 922(47, 404-69, 763)	312, 988(208, 528-405, 864)	5.52(5.21, 5.84)	4.93(4.32, 5.55)	5.40(4.98, 5.82)	6.34(5.98, 6.71)
Idiopathic developmental intellectual disability	13(6-25)	102(39-192)	6.66(6.37, 6.95)	9.14(8.30, 9.99)	5.17(4.98, 5.36)	5.99(5.91, 6.07)
Idiopathic epilepsy	520(334-760)	4, 110(2, 549-6, 205)	7.03(6.71, 7.35)	7.62(7.05, 8.20)	5.40(5.05, 5.75)	8.63(8.02, 9.24)
Inflammatory bowel disease	1, 094(606-1, 446)	4, 942(3, 676-7, 426)	5.01(4.70, 5.32)	7.51(7.24, 7.79)	2.44(1.89, 3.00)	5.16(4.59, 5.73)
Inguinal, femoral, and abdominal hernia	275(212-388)	1, 421(1, 096-1, 765)	5.44(5.22, 5.67)	6.99(6.34, 7.65)	4.71(4.59, 4.84)	4.71(4.59, 4.84)
Interpersonal violence	324(248-414)	1, 583(1, 214-2, 057)	5.31(5.17, 5.45)	5.87(5.62, 6.12)	3.56(3.40, 3.72)	6.96(6.69, 7.23)
Interstitial lung disease and pulmonary sarcoidosis	288(220-460)	3, 235(1, 947-4, 232)	8.02(7.46, 8.58)	6.86(6.56, 7.15)	10.48(8.94, 12.04)	6.42(6.05, 6.78)
Intestinal nematode infections	149(81-232)	40(22-63)	-4.16(-4.53, -3.78)	-4.73(-5.22, -4.25)	-7.85(-8.59, -7.11)	1.44(0.78, 2.10)

Invasive Non-typhoidal Salmonella (iNTS)	6(1-17)	38(6-112)	5.66(4.71, 6.63)	5.88(3.03, 8.80)	5.18(4.75, 5.61)	6.00(5.58, 6.42)
Iodine deficiency	34(18-56)	332(154-606)	7.62(7.13, 8.12)	6.94(6.50, 7.40)	9.29(7.84, 10.75)	6.62(6.36, 6.88)
Ischemic heart disease	129, 242(113, 451-145, 515)	1, 712, 072(1, 392, 996-1, 982, 910)	8.88(8.29, 9.48)	10.66(10.26, 11.05)	10.02(8.26, 11.81)	5.85(5.59, 6.12)
Kidney cancer	582(488-660)	5, 226(4, 243-6, 237)	7.20(6.54, 7.86)	8.35(7.22, 9.50)	5.46(4.24, 6.70)	8.26(7.46, 9.08)
Larynx cancer	426(360-498)	2, 992(2, 342-3, 571)	6.50(6.08, 6.93)	7.42(6.51, 8.33)	5.97(5.47, 6.47)	6.21(5.70, 6.73)
Leishmaniasis	2(0-7)	3(0-9)	0.17(-1.67, 2.05)	-3.10(-8.22, 2.30)	1.77(1.00, 2.55)	1.77(1.00, 2.55)
Leprosy	0(0-1)	2(1-3)	2.20(2.20, 2.20)	-0.03(-0.03, -0.03)	-0.03(-0.03, -0.03)	4.96(4.96, 4.96)
Leukemia	745(576-914)	5, 358(4, 149-7, 162)	6.63(6.35, 6.91)	8.89(8.48, 9.30)	5.47(4.78, 6.17)	5.89(5.72, 6.07)
Lip and oral cavity cancer	457(390-523)	4, 649(3, 757-5, 431)	7.89(7.25, 8.53)	8.83(7.33, 10.36)	7.68(7.10, 8.26)	7.28(6.57, 7.99)
Liver cancer	2, 546(2, 055-3, 041)	24, 126(18, 921-29, 736)	7.62(5.84, 9.44)	8.48(6.66, 10.34)	7.44(2.48, 12.65)	7.74(7.09, 8.39)
Low back pain	5, 858(3, 752-8, 246)	50, 232(32, 631-70, 415)	7.28(7.14, 7.43)	7.61(7.30, 7.91)	7.13(6.95, 7.30)	7.23(7.08, 7.37)
Lower extremity peripheral arterial disease	517(295-840)	5, 061(3, 155-7, 845)	7.68(7.55, 7.82)	8.74(8.50, 8.99)	7.17(6.86, 7.47)	7.35(7.27, 7.42)
Lower respiratory infections	65, 040(53, 650-74, 185)	263, 628(205, 975-330, 307)	4.53(4.18, 4.87)	6.50(5.74, 7.26)	2.97(2.51, 3.43)	4.44(4.19, 4.69)
Malaria	147(93-261)	2, 429(1, 478-3, 206)	-4.44(-4.44, -4.44)	-0.06(-0.06, -0.06)	-0.06(-0.06, -0.06)	-10.04(-10.04, -10.04)
Malignant neoplasm of bone and articular cartilage	130(94-194)	1, 373(676-1, 821)	9.45(8.73, 10.17)	12.06(9.92, 14.25)	10.58(10.02, 11.13)	5.34(5.13, 5.54)
Malignant skin melanoma	524(451-604)	0(0-0)	8.01(7.33, 8.69)	9.30(7.68, 10.95)	7.01(6.39, 7.64)	7.91(7.12, 8.71)
Meningitis	39(30-45)	1, 776(1, 404-2, 151)	4.09(3.44, 4.75)	4.15(3.12, 5.18)	4.34(2.84, 5.87)	4.51(3.93, 5.09)
Mesothelioma	8(6-11)	436(330-522)	8.09(7.50, 8.69)	8.07(6.84, 9.32)	11.35(10.37, 12.33)	5.34(4.96, 5.72)
Motor neuron disease	45(27-99)	137(101-175)	9.21(8.92, 9.50)	10.95(10.43, 11.48)	6.68(6.10, 7.26)	10.95(10.82, 11.07)
Multiple myeloma	4(2-6)	1, 440(882-1, 916)	12.28(11.38, 13.19)	21.37(19.05, 23.73)	7.38(6.44, 8.33)	9.29(8.41, 10.16)
Multiple sclerosis	750(627-893)	50(40-62)	8.32(7.91, 8.73)	10.84(9.92, 11.76)	6.93(6.46, 7.41)	7.50(6.97, 8.03)
Nasopharynx cancer	1, 057(566-1, 801)	2, 570(2, 074-3, 036)	4.04(3.56, 4.53)	6.28(5.27, 7.30)	1.15(0.51, 1.80)	5.50(5.12, 5.87)

Neck pain	34(22-50)	10, 158(5, 470-16, 938)	7.66(7.52, 7.79)	7.87(7.58, 8.15)	7.46(7.31, 7.61)	7.78(7.64, 7.92)
Neonatal disorders	2(0-3)	300(204-428)	7.34(7.20, 7.48)	6.56(6.24, 6.88)	7.26(7.13, 7.39)	8.45(8.29, 8.62)
Neuroblastoma and other peripheral nervous cell tumors	3(1-4)	120(81-150)	12.37(11.67, 13.07)	10.40(9.21, 11.61)	14.99(14.16, 15.82)	11.31(10.47, 12.15)
Non-Hodgkin lymphoma	613(516-735)	5, 652(4, 505-6, 860)	7.55(7.21, 7.88)	8.78(8.08, 9.48)	6.48(6.02, 6.94)	7.62(7.31, 7.93)
Non-melanoma skin cancer	640(513-880)	8, 748(6, 372-10, 643)	8.82(8.66, 8.97)	10.03(9.83, 10.23)	10.03(9.83, 10.23)	6.32(6.03, 6.61)
Non-rheumatic valvular heart disease	459(346-595)	3, 365(2, 575-4, 430)	6.69(6.43, 6.96)	8.16(7.63, 8.69)	5.11(4.86, 5.37)	6.95(6.44, 7.47)
Oral disorders	4, 139(2, 608-5, 848)	40, 675(26, 430-56, 916)	7.62(7.04, 8.21)	6.98(6.59, 7.37)	8.27(7.47, 9.07)	8.03(6.85, 9.21)
Osteoarthritis	3, 936(1, 985-7, 878)	43, 121(21, 915-86, 374)	8.14(7.98, 8.30)	7.93(7.57, 8.28)	8.20(8.04, 8.36)	8.45(8.29, 8.62)
Other cardiovascular and circulatory diseases	1, 287(959-1, 787)	8, 891(6, 799-11, 764)	6.53(6.34, 6.72)	7.48(7.02, 7.94)	5.50(5.30, 5.70)	6.86(6.75, 6.98)
Other chronic respiratory diseases	245(174-467)	3, 515(2, 309-4, 560)	9.22(8.51, 9.94)	12.07(10.41, 13.76)	12.62(11.84, 13.40)	3.45(3.07, 3.82)
Other digestive diseases	3, 121(2, 029-4, 054)	10, 884(8, 615-14, 350)	4.08(3.89, 4.27)	4.01(3.68, 4.34)	2.31(2.02, 2.59)	6.59(6.36, 6.82)
Other intestinal infectious diseases	8(0-26)	34(1-94)	4.53(3.71, 5.36)	5.51(4.96, 6.07)	3.39(1.01, 5.82)	4.60(4.30, 4.91)
Other malignant neoplasms	1, 263(833-1, 620)	8, 564(6, 620-10, 514)	6.44(6.20, 6.68)	6.76(6.30, 7.23)	6.49(6.09, 6.90)	6.01(5.79, 6.24)
Other mental disorders	669(445-971)	6, 313(4, 229-9, 166)	7.60(7.48, 7.71)	7.83(7.58, 8.09)	7.34(7.21, 7.47)	7.77(7.65, 7.90)
Other musculoskeletal disorders	1, 005(521-1, 754)	6, 521(3, 896-10, 997)	6.08(5.16, 7.00)	7.90(5.19, 10.68)	9.72(9.00, 10.45)	0.39(-0.05, 0.83)
Other neglected tropical diseases	73(50-103)	411(283-614)	5.76(5.60, 5.91)	6.72(6.30, 7.15)	5.11(5.00, 5.23)	5.55(5.46, 5.63)
Other neoplasms	107(62-160)	1, 573(1, 028-2, 416)	9.14(8.78, 9.50)	9.37(8.58, 10.18)	10.02(9.49, 10.55)	7.80(7.52, 8.07)
Other neurological disorders	106(75-141)	3, 348(2, 663-4, 106)	12.06(11.64, 12.48)	14.04(12.95, 15.14)	10.44(10.10, 10.77)	11.70(11.12, 12.27)
Other nutritional deficiencies	436(338-559)	5, 080(3, 766-6, 569)	8.51(7.60, 9.44)	7.75(6.16, 9.37)	11.89(10.22, 13.58)	4.90(4.35, 5.45)
Other pharynx cancer	118(101-139)	814(658-968)	6.39(5.85, 6.92)	6.87(5.77, 7.97)	5.39(4.80, 5.98)	7.43(6.73, 8.13)
Other sense organ diseases	473(245-838)	4, 599(2, 378-8, 248)	7.70(7.59, 7.82)	8.00(7.74, 8.25)	7.42(7.29, 7.55)	7.83(7.71, 7.96)
Other skin and subcutaneous diseases	468(267-760)	4, 557(2, 450-7, 878)	7.74(7.56, 7.91)	7.48(7.11, 7.86)	7.72(7.48, 7.95)	8.13(7.97, 8.28)

Other transport injuries	226(184-273)	1, 457(1, 150-1, 821)	6.30(5.75, 6.86)	8.10(7.49, 8.72)	5.67(4.17, 7.19)	5.33(5.09, 5.57)
Other unintentional injuries	473(360-638)	2, 615(2, 025-3, 420)	5.79(5.51, 6.07)	6.94(6.65, 7.23)	5.46(5.28, 5.63)	4.91(4.13, 5.70)
Other unspecified infectious diseases	321(156-442)	1, 408(855-2, 003)	4.99(4.61, 5.37)	5.74(4.84, 6.65)	2.46(2.04, 2.88)	7.20(6.82, 7.59)
Otitis media	59(31-107)	443(225-841)	6.76(6.64, 6.89)	7.01(6.74, 7.28)	6.44(6.30, 6.59)	7.00(6.86, 7.15)
Ovarian cancer	269(206-340)	2, 270(1, 561-2, 907)	7.24(6.55, 7.94)	10.29(8.45, 12.16)	3.46(2.91, 4.03)	9.06(8.14, 9.98)
Pancreatic cancer	1, 639(1, 390-1, 944)	19, 501(15, 211-23, 642)	8.35(7.98, 8.72)	9.20(8.41, 9.99)	7.03(6.45, 7.61)	9.18(8.98, 9.38)
Pancreatitis	590(420-817)	4, 186(3, 033-5, 561)	6.56(6.42, 6.70)	6.94(6.66, 7.22)	6.45(6.34, 6.57)	6.25(6.12, 6.37)
Paralytic ileus and intestinal obstruction	2, 995(2, 287-3, 834)	12, 753(9, 502-16, 626)	4.84(4.50, 5.19)	5.29(4.98, 5.61)	4.13(3.55, 4.71)	4.97(4.21, 5.73)
Parkinson's disease	7, 490(6, 144-8, 503)	70, 519(58, 100-83, 047)	7.60(7.36, 7.84)	8.76(8.37, 9.16)	6.89(6.32, 7.47)	7.40(7.26, 7.54)
Pneumoconiosis	284(231-347)	1, 988(1, 574-2, 453)	6.51(6.12, 6.89)	7.36(6.54, 8.19)	6.78(6.30, 7.26)	5.21(4.80, 5.63)
Poisonings	247(177-492)	2, 686(1, 569-3, 417)	8.23(7.64, 8.83)	9.24(8.32, 10.16)	10.58(9.33, 11.84)	3.77(3.37, 4.16)
Police conflict and executions	13(3-21)	45(24-67)	3.84(3.19, 4.49)	4.30(3.12, 5.49)	1.05(-0.41, 2.54)	6.47(6.11, 6.83)
Prostate cancer	2, 276(1, 467-2, 914)	20, 308(13, 986-27, 642)	7.26(6.33, 8.21)	9.65(7.75, 11.58)	5.44(4.27, 6.63)	7.08(5.87, 8.30)
Protein-energy malnutrition	9, 217(7, 808-10, 653)	25, 474(20, 416-30, 649)	3.34(3.12, 3.56)	1.30(0.88, 1.73)	3.53(3.30, 3.77)	5.66(5.31, 6.01)
Pruritus	78(35-154)	855(381-1, 690)	8.10(7.96, 8.24)	8.29(7.99, 8.60)	7.91(7.76, 8.06)	8.23(8.09, 8.37)
Psoriasis	117(88-146)	1, 479(1, 104-1, 852)	8.64(8.48, 8.81)	9.56(9.21, 9.90)	8.10(7.89, 8.32)	8.43(8.27, 8.60)
Pulmonary Arterial Hypertension	619(420-918)	4, 875(3, 172-6, 140)	6.77(6.32, 7.22)	5.31(4.87, 5.75)	11.47(10.56, 12.40)	3.98(3.09, 4.89)
Rabies	3(1-6)	28(12-49)	6.59(4.46, 8.76)	6.11(1.04, 11.43)	16.17(14.35, 18.03)	-3.64(-6.66, -0.51)
Rheumatic heart disease	15, 473(12, 674-19, 392)	44, 628(32, 497-57, 432)	3.49(3.07, 3.91)	4.37(3.86, 4.89)	2.62(1.51, 3.74)	3.50(3.34, 3.65)
Rheumatoid arthritis	742(620-908)	7, 655(5, 772-9, 262)	7.93(7.41, 8.44)	8.34(7.55, 9.14)	8.15(7.48, 8.83)	6.94(6.39, 7.50)
Road injuries	1, 974(1, 686-2, 341)	20, 030(17, 009-23, 358)	7.94(7.74, 8.13)	10.36(10.05, 10.67)	7.91(7.55, 8.26)	5.19(4.92, 5.46)
Scabies	413(218-700)	3, 891(2, 069-6, 626)	7.61(7.47, 7.74)	7.93(7.64, 8.22)	7.29(7.14, 7.44)	7.76(7.62, 7.90)

Schistosomiasis	112(70-163)	236(160-346)	2.36(1.92, 2.80)	1.95(1.74, 2.16)	1.08(-0.14, 2.32)	4.20(3.82, 4.59)
Schizophrenia	121(87-156)	1, 389(996-1, 785)	8.28(8.12, 8.44)	8.44(8.09, 8.79)	7.96(7.77, 8.15)	8.64(8.49, 8.79)
Self-harm	4, 597(3, 031-5, 211)	19, 499(15, 905-23, 200)	4.71(4.48, 4.95)	7.24(6.71, 7.77)	2.61(2.40, 2.81)	4.77(4.29, 5.26)
Sexually transmitted infections excluding HIV	51(33-78)	247(160-381)	5.28(4.87, 5.69)	4.00(2.85, 5.16)	5.67(5.38, 5.97)	6.19(6.05, 6.33)
Soft tissue and other extraosseous sarcomas	192(126-248)	1, 284(899-1, 802)	6.36(6.07, 6.64)	7.70(6.98, 8.43)	5.28(4.98, 5.58)	6.24(5.99, 6.48)
Stomach cancer	14, 198(11, 642-17, 559)	86, 570(66, 717-103, 671)	6.02(5.80, 6.24)	7.82(7.16, 8.49)	5.17(5.04, 5.30)	5.17(5.04, 5.30)
Stroke	223, 265(188, 527-251, 451)	1, 488, 340(1, 207, 896-1, 722, 217)	6.32(6.06, 6.58)	8.74(7.95, 9.54)	5.18(5.03, 5.33)	5.18(5.03, 5.33)
Testicular cancer	27(22-32)	216(168-271)	6.88(5.69, 8.09)	9.45(6.77, 12.19)	3.68(2.78, 4.59)	7.38(5.42, 9.38)
Tetanus	97(30-146)	42(11-93)	-2.67(-3.71, -1.61)	-1.85(-3.47, -0.20)	-2.85(-4.68, -0.97)	-3.58(-3.94, -3.21)
Thyroid cancer	221(179-268)	2, 025(1, 491-2, 440)	7.45(6.99, 7.91)	8.81(7.79, 9.84)	6.83(6.34, 7.32)	6.78(6.21, 7.35)
Tracheal, bronchus, and lung cancer	12, 127(10, 471-13, 862)	143, 362(114, 406-169, 996)	8.34(8.02, 8.67)	10.02(9.38, 10.66)	7.53(6.99, 8.08)	7.59(7.31, 7.86)
Trachoma	28(14-50)	35(15-68)	1.03(0.20, 1.87)	3.20(2.51, 3.89)	-3.13(-4.94, -1.28)	3.32(1.65, 5.01)
Tuberculosis	6, 438(5, 373-7, 527)	6, 380(5, 209-7, 772)	-0.01(-0.45, 0.44)	2.01(1.20, 2.84)	-3.97(-4.51, -3.43)	2.87(2.12, 3.62)
Typhoid and paratyphoid	1(0-2)	4(2-9)	5.14(1.08, 9.35)	1.07(-3.90, 6.30)	7.21(3.06, 11.52)	5.58(4.84, 6.33)
Upper digestive system diseases	9, 612(8, 062-11, 267)	39, 588(31, 326-47, 576)	4.69(4.14, 5.25)	5.37(5.00, 5.74)	4.80(3.14, 6.48)	3.85(3.69, 4.02)
Upper respiratory infections	5, 464(993-8, 440)	3, 344(2, 022-8, 140)	-1.71(-2.23, -1.19)	2.92(1.86, 4.00)	-8.59(-9.09, -8.09)	2.08(1.16, 3.00)
Urinary diseases and male infertility	2, 434(1, 790-3, 141)	14, 980(12, 037-19, 471)	6.09(5.89, 6.28)	7.66(7.45, 7.86)	4.35(3.86, 4.83)	6.19(5.91, 6.47)
Urticaria	68(40-106)	644(372-991)	7.57(7.46, 7.68)	7.94(7.69, 8.18)	7.22(7.10, 7.34)	7.71(7.58, 7.84)
Uterine cancer	276(204-372)	1, 728(1, 191-2, 358)	6.08(5.43, 6.73)	9.26(8.59, 9.94)	3.55(2.95, 4.15)	5.83(4.45, 7.24)
Varicella and herpes zoster	553(381-750)	868(595-1, 224)	1.43(1.19, 1.67)	1.53(1.05, 2.00)	-0.44(-0.80, -0.08)	3.80(3.46, 4.14)
Vascular intestinal disorders	166(94-217)	793(522-1, 019)	5.25(5.08, 5.42)	7.88(7.65, 8.10)	2.05(1.74, 2.36)	6.33(6.05, 6.62)
Viral skin diseases	68(43-102)	656(412-982)	7.62(7.50, 7.75)	7.80(7.53, 8.06)	7.40(7.26, 7.54)	7.84(7.71, 7.97)

Vitamin A deficiency	3(1-5)	29(15-51)	7.39(6.65, 8.15)	7.85(5.93, 9.82)	6.48(5.84, 7.14)	8.10(7.70, 8.50)
**95+years**						
Acne vulgaris	0(0-0)	6(4-10)	9.73(6.86, 12.67)	9.31(2.30, 16.81)	8.61(6.37, 10.89)	12.25(11.03, 13.50)
Acute glomerulonephritis	59(40-80)	452(303-591)	6.84(6.22, 7.46)	3.80(2.32, 5.30)	9.58(9.04, 10.12)	6.65(5.99, 7.32)
Acute hepatitis	51(40-63)	87(64-109)	1.86(1.21, 2.52)	0.48(-0.14, 1.10)	-0.46(-2.04, 1.14)	5.42(4.46, 6.39)
Adverse effects of medical treatment	61(36-82)	333(232-464)	5.75(5.00, 6.52)	3.69(3.10, 4.29)	4.41(2.43, 6.42)	9.31(8.42, 10.20)
Age-related and other hearing loss	1, 891(1, 380-2, 504)	29, 185(21, 184-38, 250)	9.61(9.01, 10.22)	7.94(7.29, 8.59)	9.92(8.31, 11.56)	10.86(10.40, 11.31)
Alcohol use disorders	33(21-50)	636(415-961)	10.28(9.62, 10.94)	8.61(7.89, 9.34)	11.07(9.30, 12.86)	10.78(10.32, 11.24)
Alopecia areata	0(0-0)	8(5-12)	7.50(6.23, 8.79)	0.27(-2.92, 3.56)	11.70(10.85, 12.55)	11.70(10.85, 12.55)
Alzheimer's disease and other dementias	18, 609(6, 794-44, 030)	303, 381(122, 968-692, 562)	9.67(9.11, 10.22)	8.23(7.63, 8.83)	9.37(7.91, 10.86)	11.34(10.92, 11.76)
Animal contact	18(14-22)	102(78-128)	5.93(5.57, 6.29)	4.95(4.59, 5.31)	4.94(4.09, 5.79)	7.89(7.36, 8.43)
Anxiety disorders	149(97-217)	2, 324(1, 540-3, 342)	9.58(8.42, 10.75)	9.01(8.10, 9.93)	9.43(6.09, 12.87)	10.12(9.59, 10.67)
Aortic aneurysm	22(15-30)	423(298-537)	10.01(9.42, 10.60)	10.97(10.09, 11.86)	7.44(6.06, 8.84)	11.39(10.78, 12.01)
Appendicitis	51(29-68)	211(135-288)	4.81(4.15, 5.47)	3.43(2.06, 4.83)	3.46(2.31, 4.62)	7.49(7.06, 7.92)
Asthma	870(579-1, 294)	5, 318(3, 823-6, 839)	6.14(5.20, 7.08)	4.68(3.23, 6.16)	5.28(2.92, 7.70)	8.44(8.17, 8.70)
Atrial fibrillation and flutter	3, 237(2, 342-4, 138)	48, 531(36, 015-60, 630)	9.55(8.22, 10.89)	8.97(8.22, 9.74)	8.95(5.04, 13.02)	10.79(10.42, 11.16)
Autism spectrum disorders	4(2-7)	131(79-199)	11.73(11.17, 12.30)	9.49(9.02, 9.95)	12.48(11.74, 13.23)	12.82(11.57, 14.08)
Bacterial skin diseases	424(286-559)	830(413-1, 860)	2.12(1.53, 2.72)	3.44(2.90, 3.98)	-6.68(-7.49, -5.86)	9.97(8.32, 11.64)
Bipolar disorder	9(5-15)	152(87-239)	9.59(8.96, 10.22)	8.45(7.78, 9.14)	9.21(7.54, 10.90)	10.94(10.46, 11.42)
Bladder cancer	262(202-306)	3, 301(2, 530-3, 970)	8.58(8.15, 9.01)	6.05(5.43, 6.67)	6.67(5.69, 7.66)	13.21(12.68, 13.74)
Blindness and vision loss	1, 703(1, 235-2, 284)	22, 257(16, 020-29, 938)	8.79(7.95, 9.64)	7.30(5.46, 9.17)	7.75(6.10, 9.42)	11.26(11.04, 11.48)
Brain and central nervous system cancer	60(45-76)	1, 273(772-1, 636)	10.74(10.09, 11.38)	8.23(7.70, 8.77)	12.27(10.61, 13.95)	11.78(11.02, 12.55)

Breast cancer	264(207-310)	3, 795(2, 698-4, 853)	9.24(8.50, 9.99)	10.07(9.15, 11.01)	5.81(3.92, 7.73)	12.18(11.56, 12.80)
Cardiomyopathy and myocarditis	383(280-561)	9, 469(6, 291-13, 065)	11.58(10.15, 13.03)	14.20(12.16, 16.28)	11.82(8.11, 15.66)	7.80(7.01, 8.59)
Cervical cancer	99(76-124)	1, 317(884-1, 722)	8.93(7.75, 10.11)	7.95(5.71, 10.24)	8.11(5.71, 10.56)	10.43(10.02, 10.83)
Chronic kidney disease	2, 933(2, 474-3, 529)	38, 582(30, 216-47, 908)	9.03(8.16, 9.90)	7.63(7.04, 8.23)	8.81(6.33, 11.34)	10.57(10.02, 11.12)
Chronic obstructive pulmonary disease	44, 358(33, 306-50, 442)	285, 487(213, 032-352, 858)	6.38(5.90, 6.87)	5.70(5.03, 6.38)	5.04(3.79, 6.31)	8.50(8.25, 8.76)
Cirrhosis and other chronic liver diseases	589(433-775)	4, 537(3, 277-5, 856)	6.91(6.03, 7.80)	4.99(3.58, 6.41)	6.12(4.20, 8.07)	9.45(9.11, 9.79)
Colon and rectum cancer	996(797-1, 177)	13, 259(9, 706-16, 331)	8.82(8.24, 9.41)	7.52(6.67, 8.37)	6.57(5.32, 7.83)	12.51(11.54, 13.49)
Congenital birth defects	6(3-9)	117(76-173)	10.12(9.70, 10.55)	9.55(8.98, 10.12)	9.89(8.87, 10.92)	10.66(10.27, 11.05)
COVID-19	NA	805(70-3, 331)	NA	NA	NA	NA
Cystic echinococcosis	2(0-4)	11(5-17)	5.75(5.09, 6.41)	4.85(3.82, 5.89)	5.65(4.96, 6.33)	6.85(5.98, 7.73)
Cysticercosis	47(19-90)	395(130-888)	7.37(6.62, 8.12)	6.26(5.32, 7.20)	5.79(4.11, 7.50)	10.37(9.11, 11.66)
Decubitus ulcer	63(33-161)	2, 247(1, 279-3, 008)	13.48(12.44, 14.53)	20.33(18.20, 22.50)	10.93(9.42, 12.46)	9.06(7.64, 10.50)
Dengue	0(0-0)	1(0-2)	2.20(2.20, 2.20)	-0.03(-0.03, -0.03)	-0.03(-0.03, -0.03)	7.86(7.86, 7.86)
Depressive disorders	388(242-579)	6, 002(3, 637-8, 908)	9.58(8.65, 10.52)	8.45(7.72, 9.18)	9.59(6.94, 12.31)	10.62(9.95, 11.29)
Dermatitis	32(17-57)	509(273-885)	9.57(9.12, 10.02)	8.28(7.63, 8.93)	9.39(8.28, 10.51)	10.90(10.50, 11.29)
Diabetes mellitus	989(829-1, 142)	17, 608(14, 010-20, 957)	9.85(9.20, 10.51)	9.11(8.64, 9.58)	8.61(6.75, 10.50)	11.65(11.22, 12.08)
Diarrheal diseases	476(186-820)	715(409-1, 395)	1.40(0.95, 1.84)	-2.18(-3.21, -1.14)	1.17(0.50, 1.86)	5.83(5.59, 6.06)
Dietary iron deficiency	376(229-547)	4, 220(2, 397-6, 332)	8.24(7.98, 8.51)	8.72(8.35, 9.09)	6.39(5.76, 7.03)	9.30(9.04, 9.56)
Drowning	144(116-168)	1, 890(1, 467-2, 275)	8.93(7.46, 10.42)	7.27(5.23, 9.35)	9.10(5.38, 12.95)	10.11(9.70, 10.53)
Drug use disorders	32(25-40)	199(155-252)	6.08(5.87, 6.29)	5.09(4.75, 5.42)	4.03(3.69, 4.36)	10.04(9.76, 10.33)
Encephalitis	7(5-8)	98(73-125)	9.21(7.99, 10.45)	8.91(8.14, 9.67)	10.27(6.70, 13.96)	8.51(7.99, 9.03)
Endocarditis	54(23-76)	323(196-463)	6.15(5.58, 6.72)	7.67(6.90, 8.45)	1.56(0.38, 2.77)	9.11(8.13, 10.10)

Endocrine, metabolic, blood, and immune disorders	340(247-459)	4, 840(3, 211-6, 334)	9.21(8.77, 9.65)	6.86(6.26, 7.46)	9.04(7.94, 10.16)	11.59(11.18, 11.99)
Environmental heat and cold exposure	140(48-203)	896(374-1, 225)	6.38(5.84, 6.92)	3.53(3.19, 3.88)	5.97(5.03, 6.91)	10.06(8.86, 11.27)
Esophageal cancer	715(456-875)	8, 236(5, 514-10, 411)	8.41(7.55, 9.27)	6.76(5.21, 8.33)	7.67(5.85, 9.53)	10.78(10.47, 11.08)
Exposure to forces of nature	7(6-8)	58(43-78)	4.83(-8.35, 19.91)	-3.79(-10.69, 3.65)	18.32(-20.74, 76.64)	2.07(-3.46, 7.92)
Exposure to mechanical forces	52(41-74)	1, 011(692-1, 268)	10.20(9.63, 10.76)	7.67(6.22, 9.14)	12.44(12.01, 12.88)	10.07(9.62, 10.52)
Eye cancer	4(2-6)	73(24-116)	10.01(9.32, 10.70)	6.90(5.85, 7.96)	11.68(10.38, 13.00)	11.62(10.85, 12.39)
Falls	2, 943(2, 389-3, 586)	61, 824(41, 386-79, 500)	10.82(10.30, 11.35)	9.36(8.91, 9.82)	9.36(8.91, 9.82)	13.71(12.36, 15.08)
Fire, heat, and hot substances	202(145-243)	1, 686(1, 203-2, 089)	7.23(6.68, 7.79)	4.08(3.11, 5.06)	8.55(7.52, 9.59)	9.00(8.23, 9.76)
Food-borne trematodiases	10(6-15)	162(101-234)	9.64(9.16, 10.12)	9.56(9.01, 10.11)	8.95(7.69, 10.21)	10.26(9.84, 10.68)
Foreign body	82(64-98)	1, 596(992-2, 042)	10.06(9.12, 11.01)	7.57(4.98, 10.22)	10.76(10.31, 11.21)	11.87(11.08, 12.67)
Fungal skin diseases	51(19-114)	796(299-1, 765)	9.57(9.11, 10.03)	8.35(7.69, 9.01)	9.35(8.23, 10.49)	10.85(10.45, 11.25)
Gallbladder and biliary diseases	981(579-1, 293)	9, 092(6, 891-12, 707)	7.56(6.65, 8.49)	5.35(4.01, 6.70)	6.88(4.54, 9.28)	10.56(10.32, 10.81)
Gallbladder and biliary tract cancer	112(69-143)	1, 587(961-2, 073)	9.06(8.64, 9.48)	6.93(6.59, 7.27)	8.50(8.08, 8.93)	12.15(11.04, 13.28)
Gout	34(19-57)	671(376-1, 101)	10.13(9.58, 10.69)	6.90(5.37, 8.45)	11.32(11.04, 11.61)	12.23(11.83, 12.64)
Gynecological diseases	72(37-127)	1, 106(576-1, 890)	9.55(8.75, 10.37)	8.27(7.63, 8.90)	9.83(7.51, 12.20)	10.45(10.01, 10.88)
Headache disorders	60(7-147)	994(106-2, 351)	9.83(9.31, 10.35)	8.38(7.81, 8.96)	10.16(8.78, 11.56)	10.81(10.42, 11.20)
Hemoglobinopathies and hemolytic anemias	205(146-273)	2, 346(1, 644-3, 183)	8.46(8.01, 8.91)	8.14(7.52, 8.75)	7.43(6.34, 8.53)	9.62(9.23, 10.02)
HIV/AIDS	0(0-3)	113(57-207)	14.54(12.32, 16.81)	17.58(11.84, 23.61)	9.26(7.21, 11.35)	17.72(15.92, 19.54)
Hodgkin lymphoma	6(2-9)	43(22-60)	6.58(5.98, 7.19)	4.44(3.53, 5.35)	4.82(3.33, 6.33)	10.82(10.42, 11.22)
Hypertensive heart disease	9, 172(6, 837-11, 147)	83, 142(52, 795-112, 642)	7.55(6.48, 8.63)	4.57(3.85, 5.29)	7.96(4.87, 11.15)	10.16(9.44, 10.87)
Idiopathic developmental intellectual disability	1(0-2)	20(8-37)	8.29(6.68, 9.93)	7.71(5.89, 9.55)	8.69(4.30, 13.26)	7.96(6.93, 9.00)
Idiopathic epilepsy	73(43-113)	1, 049(595-1, 704)	9.16(8.11, 10.22)	8.44(7.25, 9.64)	7.79(4.95, 10.71)	11.36(10.95, 11.78)

Inflammatory bowel disease	208(96-294)	1, 515(1, 026-2, 436)	6.75(6.21, 7.29)	6.35(5.13, 7.58)	4.47(3.84, 5.11)	9.85(9.35, 10.34)
Inguinal, femoral, and abdominal hernia	27(21-38)	283(209-367)	8.07(6.96, 9.19)	6.61(5.96, 7.26)	7.01(3.88, 10.23)	10.60(9.74, 11.46)
Interpersonal violence	45(36-57)	346(268-442)	6.85(6.33, 7.37)	5.52(4.13, 6.92)	4.92(4.29, 5.55)	10.80(10.58, 11.02)
Interstitial lung disease and pulmonary sarcoidosis	41(29-62)	797(470-1, 102)	10.26(9.37, 11.17)	6.96(6.45, 7.47)	12.21(9.62, 14.86)	11.76(11.07, 12.47)
Intestinal nematode infections	15(9-24)	8(5-12)	-1.99(-2.80, -1.16)	-5.18(-6.02, -4.34)	-4.06(-6.10, -1.97)	4.57(3.71, 5.44)
Invasive Non-typhoidal Salmonella (iNTS)	0(0-2)	9(0-36)	7.57(3.25, 12.07)	2.43(-1.96, 7.02)	7.77(-0.93, 17.23)	10.69(9.90, 11.48)
Iodine deficiency	4(2-6)	62(28-115)	9.31(8.58, 10.04)	6.42(4.50, 8.38)	11.16(10.68, 11.65)	10.05(9.44, 10.66)
Ischemic heart disease	23, 872(19, 386-27, 591)	481, 506(362, 861-577, 625)	10.32(9.42, 11.22)	8.19(5.69, 10.75)	12.16(11.78, 12.53)	10.27(9.36, 11.19)
Kidney cancer	43(34-52)	745(571-901)	9.76(8.99, 10.54)	7.22(6.43, 8.02)	8.38(6.35, 10.44)	13.95(13.20, 14.71)
Larynx cancer	30(23-38)	411(290-531)	8.89(8.59, 9.19)	5.64(5.27, 6.01)	9.22(8.91, 9.52)	11.96(11.36, 12.56)
Leishmaniasis	0(0-1)	0(0-2)	2.21(2.21, 2.21)	-0.03(-0.03, -0.03)	4.97(4.97, 4.97)	-0.54(-0.54, -0.54)
Leukemia	68(51-85)	871(628-1, 117)	8.88(8.04, 9.73)	8.75(8.16, 9.35)	7.58(5.21, 10.01)	10.17(9.56, 10.79)
Lip and oral cavity cancer	52(41-62)	862(647-1, 046)	9.58(8.88, 10.29)	7.44(6.46, 8.42)	8.68(6.96, 10.42)	12.76(12.05, 13.47)
Liver cancer	143(109-179)	3, 403(2, 544-4, 221)	10.97(10.08, 11.87)	7.15(5.87, 8.45)	13.43(12.30, 14.57)	12.05(10.83, 13.29)
Low back pain	680(421-1, 008)	9, 808(6, 124-14, 328)	9.31(8.59, 10.03)	8.08(7.53, 8.64)	9.36(7.30, 11.47)	10.30(9.91, 10.69)
Lower extremity peripheral arterial disease	76(44-124)	1, 367(855-2, 097)	10.09(9.19, 10.99)	10.08(9.34, 10.82)	9.19(6.67, 11.78)	10.83(10.37, 11.28)
Lower respiratory infections	12, 101(8, 581-14, 365)	81, 100(58, 900-106, 481)	6.43(5.56, 7.31)	5.78(5.23, 6.33)	5.24(2.81, 7.73)	8.28(7.45, 9.12)
Malignant neoplasm of bone and articular cartilage	14(8-26)	414(243-582)	11.79(10.85, 12.73)	11.96(9.56, 14.41)	13.13(12.52, 13.75)	9.46(8.86, 10.06)
Malignant skin melanoma	15(10-28)	299(130-418)	10.20(9.07, 11.35)	8.55(7.11, 10.00)	9.43(6.35, 12.60)	12.85(12.47, 13.22)
Meningitis	97(79-118)	603(446-731)	6.20(5.57, 6.83)	3.43(2.48, 4.40)	6.82(5.80, 7.86)	8.80(8.07, 9.53)
Mesothelioma	4(3-5)	86(60-110)	10.15(8.82, 11.49)	8.26(7.30, 9.23)	12.50(8.52, 16.63)	9.91(9.51, 10.32)
Motor neuron disease	0(0-1)	25(18-32)	11.30(10.34, 12.27)	7.49(5.89, 9.11)	10.86(9.85, 11.88)	16.11(15.15, 17.09)

Multiple myeloma	3(1-9)	198(107-276)	13.77(12.82, 14.73)	20.43(18.13, 22.78)	8.21(6.60, 9.84)	14.19(13.80, 14.58)
Multiple sclerosis	0(0-0)	8(6-10)	10.03(6.84, 13.33)	9.22(0.82, 18.31)	10.37(9.11, 11.64)	11.52(10.50, 12.54)
Nasopharynx cancer	74(58-89)	555(406-695)	6.77(6.32, 7.23)	7.48(6.86, 8.11)	2.58(1.62, 3.55)	10.25(9.51, 11.00)
Neck pain	122(66-217)	1, 923(1, 053-3, 411)	9.63(9.05, 10.20)	8.36(7.75, 8.98)	9.42(7.90, 10.96)	10.92(10.49, 11.36)
Neonatal disorders	4(2-6)	59(40-86)	9.17(8.10, 10.25)	7.00(6.20, 7.80)	8.93(5.85, 12.09)	11.60(10.99, 12.22)
Neuroblastoma and other peripheral nervous cell tumors	0(0-0)	27(13-38)	14.45(11.34, 17.64)	9.20(1.58, 17.39)	17.92(15.91, 19.97)	17.40(15.73, 19.09)
Non-Hodgkin lymphoma	71(56-87)	1, 144(803-1, 415)	9.52(9.04, 10.01)	8.33(7.65, 9.02)	7.95(6.78, 9.13)	12.37(11.80, 12.94)
Non-melanoma skin cancer	92(69-125)	2, 465(1, 647-3, 063)	11.25(10.23, 12.28)	9.87(6.91, 12.91)	12.69(12.40, 12.99)	10.89(10.15, 11.62)
Non-rheumatic valvular heart disease	81(59-106)	1, 079(810-1, 422)	9.01(8.53, 9.48)	8.80(8.19, 9.41)	7.40(6.28, 8.54)	10.72(10.09, 11.35)
Oral disorders	550(339-787)	8, 975(5, 784-12, 601)	9.78(9.30, 10.27)	7.98(7.48, 8.49)	10.71(9.45, 11.98)	10.86(10.44, 11.28)
Osteoarthritis	543(271-1, 074)	9, 745(4, 942-19, 413)	10.06(9.75, 10.37)	7.89(7.46, 8.33)	10.95(10.57, 11.34)	11.30(10.83, 11.77)
Other cardiovascular and circulatory diseases	177(128-249)	2, 233(1, 579-2, 993)	8.84(7.89, 9.80)	7.59(6.95, 8.24)	7.98(5.30, 10.73)	11.01(10.25, 11.78)
Other chronic respiratory diseases	41(26-99)	1, 321(813-1, 826)	12.21(11.77, 12.64)	12.87(12.42, 13.32)	15.71(14.63, 16.79)	7.85(7.48, 8.22)
Other digestive diseases	528(296-692)	3, 166(2, 284-4, 701)	6.09(5.57, 6.60)	3.94(3.28, 4.61)	4.54(3.19, 5.91)	10.11(9.83, 10.39)
Other intestinal infectious diseases	2(0-7)	13(0-41)	6.87(6.22, 7.53)	5.37(4.34, 6.42)	6.70(6.02, 7.38)	8.72(7.89, 9.56)
Other malignant neoplasms	127(78-173)	1, 455(1, 072-1, 866)	8.33(7.53, 9.14)	5.91(5.35, 6.48)	7.77(5.48, 10.12)	11.37(10.85, 11.89)
Other mental disorders	98(63-144)	1, 515(981-2, 234)	9.56(9.04, 10.07)	8.15(7.59, 8.70)	9.45(8.07, 10.84)	10.91(10.52, 11.31)
Other musculoskeletal disorders	100(37-217)	1, 331(858-2, 051)	8.80(8.05, 9.56)	7.81(6.56, 9.08)	14.04(13.38, 14.70)	4.75(3.39, 6.13)
Other neglected tropical diseases	15(10-22)	183(120-261)	8.40(8.08, 8.71)	8.32(7.98, 8.66)	7.49(6.68, 8.31)	9.21(8.92, 9.50)
Other neoplasms	11(7-16)	319(226-456)	11.57(11.23, 11.92)	9.55(9.03, 10.07)	12.27(11.93, 12.62)	12.92(12.34, 13.50)
Other neurological disorders	13(10-18)	779(613-952)	14.25(13.28, 15.23)	13.85(13.01, 14.71)	13.48(10.75, 16.28)	15.28(14.72, 15.84)
Other nutritional deficiencies	94(67-125)	1, 869(1, 280-2, 465)	10.36(8.90, 11.84)	7.45(4.59, 10.40)	13.69(12.18, 15.23)	8.65(7.59, 9.73)

Other pharynx cancer	9(7-12)	134(89-175)	8.83(8.41, 9.25)	4.97(4.18, 5.77)	9.47(9.09, 9.85)	12.57(12.18, 12.96)
Other sense organ diseases	42(22-76)	685(347-1, 210)	9.67(9.15, 10.20)	8.34(7.77, 8.92)	9.62(8.22, 11.05)	10.91(10.51, 11.32)
Other skin and subcutaneous diseases	58(33-96)	921(505-1, 523)	9.41(8.78, 10.05)	6.50(4.79, 8.24)	10.22(9.83, 10.62)	11.50(11.05, 11.95)
Other transport injuries	32(24-41)	373(279-485)	8.79(7.55, 10.04)	7.91(7.07, 8.76)	8.91(5.37, 12.58)	9.31(8.68, 9.94)
Other unintentional injuries	72(55-105)	681(512-882)	7.52(6.89, 8.15)	5.81(4.19, 7.45)	8.51(8.07, 8.94)	8.07(7.39, 8.75)
Other unspecified infectious diseases	48(25-68)	453(282-632)	7.65(7.01, 8.31)	6.39(5.64, 7.15)	5.95(4.42, 7.51)	10.64(9.74, 11.55)
Otitis media	7(3-14)	101(51-188)	8.91(8.25, 9.57)	7.34(6.65, 8.05)	8.98(7.22, 10.76)	10.29(9.76, 10.81)
Ovarian cancer	37(27-48)	505(334-660)	8.92(7.40, 10.45)	10.20(8.81, 11.60)	4.84(0.72, 9.13)	12.18(11.23, 13.15)
Pancreatic cancer	168(132-201)	3, 529(2, 560-4, 405)	10.47(9.63, 11.32)	8.80(7.98, 9.62)	8.70(6.57, 10.88)	14.10(13.07, 15.14)
Pancreatitis	88(55-135)	1, 039(686-1, 463)	8.30(7.56, 9.04)	5.90(4.15, 7.69)	8.14(7.70, 8.59)	11.24(9.83, 12.67)
Paralytic ileus and intestinal obstruction	414(301-535)	2, 923(2, 029-3, 877)	6.53(5.71, 7.36)	3.71(1.91, 5.54)	6.09(5.58, 6.61)	10.36(8.58, 12.17)
Parkinson's disease	758(594-905)	13, 229(10, 423-16, 174)	9.97(9.32, 10.63)	8.60(8.13, 9.07)	9.54(7.66, 11.45)	11.77(11.34, 12.21)
Pneumoconiosis	19(13-25)	267(208-335)	9.00(8.73, 9.28)	5.12(4.78, 5.46)	10.04(9.73, 10.35)	11.76(11.27, 12.26)
Poisonings	24(16-52)	468(275-598)	10.08(9.31, 10.86)	7.57(5.70, 9.47)	13.01(12.46, 13.57)	8.89(8.34, 9.44)
Police conflict and executions	1(0-3)	11(6-16)	5.73(5.14, 6.33)	-0.10(-0.71, 0.51)	6.43(5.77, 7.10)	11.24(10.03, 12.46)
Prostate cancer	107(67-141)	2, 376(1, 386-3, 282)	10.06(8.27, 11.88)	4.57(3.79, 5.35)	10.06(6.70, 13.53)	14.52(12.31, 16.79)
Protein-energy malnutrition	2, 089(1, 667-2, 465)	9, 834(7, 165-12, 060)	5.11(4.75, 5.47)	1.22(0.73, 1.72)	4.82(4.10, 5.56)	10.01(9.51, 10.50)
Pruritus	9(4-18)	173(76-339)	10.04(9.19, 10.90)	8.56(7.89, 9.24)	10.16(7.70, 12.67)	11.29(10.85, 11.73)
Psoriasis	12(9-15)	253(190-325)	10.63(10.00, 11.27)	9.93(9.21, 10.66)	10.34(8.67, 12.03)	11.41(10.95, 11.87)
Pulmonary Arterial Hypertension	69(42-124)	1, 208(785-1, 693)	9.96(9.13, 10.80)	7.45(6.91, 7.99)	14.36(11.86, 16.93)	8.45(8.14, 8.76)
Rabies	0(0-1)	8(2-17)	5.71(0.93, 10.73)	3.53(-7.43, 15.80)	19.93(11.14, 29.43)	-7.59(-9.18, -5.97)
Rheumatic heart disease	2, 271(1, 708-3, 060)	11, 218(7, 416-14, 774)	5.47(4.45, 6.51)	3.91(2.58, 5.26)	5.12(2.45, 7.85)	7.30(6.99, 7.61)

Rheumatoid arthritis	106(85-128)	1, 825(1, 393-2, 234)	9.67(8.85, 10.49)	7.75(5.75, 9.79)	10.83(10.42, 11.24)	10.28(8.79, 11.79)
Road injuries	197(167-236)	3, 536(2, 867-4, 153)	9.79(8.61, 10.98)	8.54(5.12, 12.08)	10.86(10.59, 11.13)	9.76(8.77, 10.74)
Scabies	56(30-96)	877(462-1, 481)	9.59(9.10, 10.07)	8.41(7.72, 9.10)	9.26(8.07, 10.46)	10.93(10.51, 11.35)
Schistosomiasis	12(4-25)	53(29-83)	5.02(4.01, 6.04)	2.14(1.50, 2.79)	4.20(1.67, 6.80)	8.73(8.48, 8.98)
Schizophrenia	9(6-13)	210(144-285)	10.64(9.99, 11.31)	9.43(8.90, 9.97)	10.34(8.45, 12.26)	12.06(11.71, 12.42)
Self-harm	403(239-474)	3, 043(2, 346-3, 741)	7.06(6.32, 7.80)	6.00(5.54, 6.47)	5.47(3.36, 7.61)	10.10(9.67, 10.53)
Sexually transmitted infections excluding HIV	8(5-11)	72(48-103)	7.28(6.67, 7.89)	3.14(1.89, 4.41)	9.26(8.57, 9.94)	9.37(8.61, 10.13)
Soft tissue and other extraosseous sarcomas	14(8-19)	164(108-223)	8.48(7.90, 9.06)	7.28(6.75, 7.82)	7.15(5.66, 8.67)	11.05(10.38, 11.73)
Stomach cancer	1, 516(1, 188-1, 831)	16, 261(11, 618-19, 934)	8.36(7.27, 9.47)	7.58(6.90, 8.26)	7.85(4.77, 11.03)	9.64(8.67, 10.63)
Stroke	29, 366(23, 325-33, 625)	324, 920(252, 624-389, 088)	8.26(7.09, 9.45)	7.42(5.53, 9.34)	7.54(4.66, 10.50)	9.64(9.34, 9.94)
Testicular cancer	2(1-2)	38(30-47)	10.42(8.88, 11.98)	7.94(4.99, 10.98)	7.33(5.48, 9.21)	15.56(14.25, 16.89)
Tetanus	12(3-22)	9(2-23)	-0.65(-1.67, 0.38)	-2.46(-3.13, -1.80)	-1.28(-3.65, 1.15)	1.15(0.70, 1.60)
Thyroid cancer	26(19-33)	352(261-442)	8.93(8.48, 9.38)	8.01(7.53, 8.49)	7.45(6.29, 8.64)	11.37(10.88, 11.87)
Tracheal, bronchus, and lung cancer	1, 210(970-1, 439)	25, 284(18, 995-30, 469)	10.47(9.90, 11.05)	8.91(8.50, 9.33)	9.54(7.91, 11.20)	13.06(12.65, 13.47)
Trachoma	5(2-9)	10(3-22)	1.92(-1.01, 4.93)	-0.51(-4.70, 3.85)	0.66(-6.79, 8.72)	5.55(4.64, 6.47)
Tuberculosis	1, 093(880-1, 271)	1, 141(872-1, 399)	0.13(-0.26, 0.53)	0.74(0.23, 1.24)	-2.57(-3.51, -1.62)	1.89(1.61, 2.17)
Typhoid and paratyphoid	0(0-0)	0(0-1)	2.20(2.20, 2.20)	-0.03(-0.03, -0.03)	-0.03(-0.03, -0.03)	7.86(7.86, 7.86)
Upper digestive system diseases	1, 431(1, 102-1, 732)	10, 145(7, 484-12, 805)	6.77(5.69, 7.85)	4.82(3.23, 6.42)	6.92(4.62, 9.27)	8.00(7.60, 8.40)
Upper respiratory infections	946(190-1, 546)	1, 099(590-2, 934)	0.60(-0.05, 1.26)	4.59(3.43, 5.76)	-6.06(-7.02, -5.10)	5.57(4.01, 7.14)
Urinary diseases and male infertility	276(186-340)	2, 937(2, 232-3, 984)	8.30(7.55, 9.06)	8.42(7.71, 9.14)	6.28(4.35, 8.25)	10.11(9.28, 10.93)
Urticaria	8(5-13)	129(77-205)	9.58(8.76, 10.41)	8.36(7.72, 9.01)	9.50(7.14, 11.92)	10.75(10.31, 11.19)
Uterine cancer	35(25-53)	387(247-541)	8.41(7.87, 8.95)	10.38(9.56, 11.21)	6.89(6.21, 7.57)	8.43(7.31, 9.57)

Varicella and herpes zoster	117(80-171)	289(181-418)	2.99(2.58, 3.39)	1.88(1.14, 2.63)	2.15(1.24, 3.07)	4.94(4.77, 5.12)
Vascular intestinal disorders	23(9-31)	191(103-274)	7.02(6.43, 7.61)	7.81(6.32, 9.31)	3.16(2.59, 3.73)	10.73(10.20, 11.25)
Viral skin diseases	7(4-10)	113(72-164)	9.53(8.84, 10.23)	7.94(7.19, 8.69)	9.46(7.62, 11.33)	11.09(10.52, 11.66)
Vitamin A deficiency	0(0-0)	5(2-10)	9.04(6.48, 11.65)	9.34(3.85, 15.12)	6.00(2.65, 9.45)	12.89(11.37, 14.42)

UI, Uncertainty Interval; CI, Confidence Interval.

Supplementary Table 4. Projection of number and rate of disease-adjusted life years aged 65-69 years attributable to all causes from 2022 to 2045

Year	Number（Female）	Rate (Female)	Number (Male)	Rate (Male)
2022	19,025,924	48,608.12	25,324,130	67,328.22
2023	18,846,673	48,169.25	25,005,584	66,682.57
2024	18,240,310	47,730.38	24,127,800	66,036.91
2025	17,682,127	47,651.84	23,232,132	65,615.88
2026	17,352,727	47,573.31	22,651,054	65,194.85
2027	17,562,014	47,494.77	22,765,655	64,773.81
2028	18,633,732	47,416.24	23,926,068	64,352.78
2029	20,403,355	47,337.70	25,886,653	63,931.75
2030	22,638,789	47,658.73	28,438,927	64,190.28
2031	24,772,374	47,979.76	30,854,751	64,448.81
2032	26,391,784	48,300.78	32,673,710	64,707.34
2033	27,346,808	48,621.81	33,782,220	64,965.87
2034	27,847,584	48,942.84	34,437,028	65,224.40
2035	28,027,747	49,276.22	34,478,609	64,990.19
2036	28,005,987	49,609.59	34,264,188	64,755.97
2037	27,908,361	49,942.97	33,886,762	64,521.76
2038	27,679,462	50,276.35	33,271,834	64,287.54
2039	27,239,634	50,609.73	32,379,286	64,053.32
2040	26,757,521	51,092.86	31,587,246	64,312.01
2041	26,241,193	51,575.99	30,767,914	64,570.69

2042	25,782,871	52,059.13	30,045,929	64,829.38
2043	25,252,016	52,542.26	29,267,457	65,088.07
2044	24,583,978	53,025.39	28,347,220	65,346.75
2045	23,975,460	53,508.53	27,512,106	65,605.44

Supplementary Table 5. Projection of number and rate of disease-adjusted life years aged 70-74 years attributable to all cause from 2022 to 2045

Year	Number（female）	Rate (female)	Number (male)	Rate (male)
2022	19,298,456	67,727.92	24,544,654	94,511.70
2023	20,294,768	67,249.43	25,668,890	93,702.53
2024	21,357,391	66,770.94	26,937,045	92,893.35
2025	22,388,314	66,413.91	28,110,843	92,012.01
2026	23,217,528	66,056.89	29,014,055	91,130.67
2027	23,716,920	65,699.86	29,466,433	90,249.34
2028	23,594,440	65,342.84	29,118,531	89,368.00
2029	22,938,016	64,985.81	28,124,436	88,486.66
2030	22,332,785	65,091.94	27,200,064	88,179.91
2031	22,017,974	65,198.07	26,646,435	87,873.16
2032	22,390,503	65,304.20	26,916,163	87,566.41
2033	23,870,323	65,410.33	28,429,743	87,259.66
2034	26,254,551	65,516.45	30,902,335	86,952.91
2035	29,238,418	66,147.06	34,070,427	87,514.96
2036	32,102,729	66,777.66	37,085,189	88,077.01
2037	34,310,806	67,408.27	39,392,323	88,639.07
2038	35,667,339	68,038.87	40,858,971	89,201.12
2039	36,443,801	68,669.48	41,794,196	89,763.17
2040	36,694,650	69,106.21	41,905,470	89,460.65

2041	36,682,304	69,542.94	41,705,108	89,158.12
2042	36,567,692	69,979.67	41,300,098	88,855.59
2043	36,278,779	70,416.40	40,601,763	88,553.07
2044	35,715,271	70,853.13	39,567,309	88,250.54
2045	34,994,692	71,289.86	38,359,564	87,948.02

Supplementary Table 6. Projection of number and rate of disease-adjusted life years aged 75-79 years attributable to all cause from 2022 to 2045

Year	Number（female）	Rate (female)	Number (male)	Rate (male)
2022	15,892,851	88,309.77	19,646,395	123,583.48
2023	16,754,869	87,599.02	20,507,270	122,602.88
2024	17,796,544	86,888.27	21,554,068	121,622.28
2025	18,991,255	86,484.36	22,672,951	120,554.12
2026	20,197,598	86,080.46	23,798,205	119,485.95
2027	21,341,732	85,676.56	24,867,341	118,417.78
2028	22,534,784	85,272.66	26,057,944	117,349.62
2029	23,819,225	84,868.76	27,410,556	116,281.45
2030	25,096,366	84,688.13	28,758,166	115,503.01
2031	26,147,760	84,507.49	29,819,760	114,724.58
2032	26,819,215	84,326.86	30,398,882	113,946.15
2033	26,781,452	84,146.23	30,143,982	113,167.71
2034	26,144,883	83,965.60	29,236,146	112,389.28
2035	25,570,819	84,352.63	28,413,782	112,294.04
2036	25,346,620	84,739.65	28,005,623	112,198.81
2037	25,929,958	85,126.68	28,482,918	112,103.58
2038	27,808,687	85,513.70	30,286,071	112,008.35
2039	30,747,664	85,900.73	33,108,683	111,913.12
2040	34,273,853	86,683.15	36,558,639	112,600.47

2041	37,635,688	87,465.57	39,815,547	113,287.83
2042	40,208,396	88,248.00	42,290,869	113,975.18
2043	41,791,728	89,030.42	43,880,693	114,662.54
2044	42,721,359	89,812.84	44,937,052	115,349.89
2045	43,172,879	90,595.27	45,564,648	116,037.25

Supplementary Table 7. Projection of number and rate of disease-adjusted life years aged 80-84 years attributable to all cause from 2022 to 2045

Year	Number（female）	Rate (female)	Number (male)	Rate (male)
2022	13,575,737	122,249.85	15,149,736	164,635.81
2023	13,904,937	120,786.01	15,326,813	161,867.08
2024	14,292,177	119,322.16	15,511,411	159,098.34
2025	14,892,263	118,917.86	15,971,848	158,467.87
2026	15,601,290	118,513.56	16,542,435	157,837.39
2027	16,432,585	118,109.26	17,246,340	157,206.92
2028	17,467,645	117,704.96	18,149,498	156,576.44
2029	18,707,533	117,300.66	19,237,996	155,945.97
2030	20,087,879	117,114.34	20,374,729	154,993.22
2031	21,485,273	116,928.03	21,519,071	154,040.46
2032	22,818,934	116,741.71	22,612,478	153,087.71
2033	24,231,632	116,555.40	23,845,822	152,134.96
2034	25,777,230	116,369.08	25,263,759	151,182.21
2035	27,321,117	116,465.05	26,678,816	150,567.05
2036	28,610,264	116,561.02	27,812,925	149,951.90
2037	29,459,170	116,656.99	28,463,705	149,336.75
2038	29,515,750	116,752.97	28,321,576	148,721.59
2039	28,932,970	116,848.94	27,597,721	148,106.44
2040	28,363,334	117,352.79	26,924,138	147,987.67

2041	28,226,733	117,856.64	26,688,523	147,868.90
2042	29,023,802	118,360.50	27,327,633	147,750.13
2043	31,284,652	118,864.35	29,249,905	147,631.36
2044	34,718,490	119,368.21	32,139,939	147,512.59
2045	38,594,047	119,872.06	35,354,099	147,393.83

Supplementary Table 8. Projection of number and rate of disease-adjusted life years aged 85-89 years attributable to all cause from 2022 to 2045

Year	Number（female）	Rate (female)	Number (male)	Rate (male)
2022	9,744,457	159,310.58	12,192,874	282,196.65
2023	9,903,454	158,151.71	12,450,272	280,238.75
2024	10,022,613	156,992.83	12,705,353	278,280.84
2025	10,156,940	155,666.21	12,935,508	275,111.42
2026	10,354,936	154,339.60	13,200,878	271,942.00
2027	10,639,215	153,012.99	13,494,931	268,772.57
2028	10,992,086	151,686.37	13,797,250	265,603.15
2029	11,400,981	150,359.76	14,132,086	262,433.72
2030	11,981,951	150,274.35	14,696,002	262,035.58
2031	12,659,204	150,188.94	15,371,953	261,637.43
2032	13,443,479	150,103.53	16,175,022	261,239.29
2033	14,411,696	150,018.12	17,180,784	260,841.15
2034	15,568,288	149,932.71	18,383,770	260,443.00
2035	16,851,080	150,109.01	19,648,650	259,541.15
2036	18,152,579	150,285.31	20,928,374	258,639.31
2037	19,401,019	150,461.61	22,164,022	257,737.46
2038	20,746,135	150,637.91	23,578,991	256,835.61
2039	22,241,872	150,814.21	25,218,496	255,933.76
2040	23,680,291	150,932.29	26,798,011	254,995.68

2041	24,878,642	151,050.38	28,075,560	254,057.60
2042	25,658,365	151,168.46	28,833,804	253,119.51
2043	25,728,412	151,286.54	28,777,445	252,181.43
2044	25,267,913	151,404.63	28,160,806	251,243.35
2045	24,783,764	151,522.71	27,550,139	250,305.27

Supplementary Table 9. Projection of number and rate of disease-adjusted life years aged 90-94 years attributable to all cause from 2022 to 2045

Year	Number（female）	Rate (female)	Number (male)	Rate (male)
2022	4,769,411	215,882.92	5,182,517	407,309.76
2023	4,935,431	213,395.66	5,485,112	407,134.02
2024	5,160,003	210,908.40	5,900,022	406,958.27
2025	5,421,906	209,577.20	6,273,852	403,542.45
2026	5,654,053	208,246.00	6,583,241	400,126.63
2027	5,829,002	206,914.80	6,793,692	396,710.80
2028	5,953,909	205,583.60	6,965,785	393,294.98
2029	6,077,438	204,252.41	7,174,180	389,879.16
2030	6,234,025	203,115.93	7,411,846	386,446.16
2031	6,432,541	201,979.45	7,665,347	383,013.17
2032	6,680,516	200,842.98	7,921,171	379,580.18
2033	6,972,967	199,706.50	8,184,380	376,147.18
2034	7,311,773	198,570.02	8,486,766	372,714.19
2035	7,768,015	198,969.82	8,934,387	372,912.66
2036	8,295,635	199,369.61	9,459,112	373,111.14
2037	8,899,993	199,769.40	10,066,911	373,309.62
2038	9,641,773	200,169.19	10,818,596	373,508.09
2039	10,526,364	200,568.98	11,716,648	373,706.57
2040	11,472,346	200,791.46	12,639,678	372,502.09

2041	12,426,546	201,013.94	13,569,434	371,297.61
2042	13,339,060	201,236.41	14,465,520	370,093.13
2043	14,344,820	201,458.89	15,514,231	368,888.66
2044	15,486,056	201,681.37	16,753,223	367,684.18
2045	16,591,539	201,903.85	17,954,310	366,479.70

Supplementary Table 10. Projection of number and rate of disease-adjusted life years aged 95+ years attributable to all cause from 2022 to 2045

Year	Number（female）	Rate (female)	Number (male)	Rate (male)
2022	1,509,642	298,793.81	749,369	361,601.25
2023	1,587,022	295,328.19	798,447	350,351.06
2024	1,728,712	291,862.58	894,343	339,100.86
2025	1,874,779	289,693.23	1,004,320	336,367.57
2026	1,990,062	287,523.89	1,087,708	333,634.28
2027	2,066,632	285,354.54	1,140,178	330,900.99
2028	2,174,011	283,185.20	1,219,495	328,167.70
2029	2,332,769	281,015.85	1,338,606	325,434.41
2030	2,503,912	279,993.79	1,458,351	323,212.00
2031	2,651,563	278,971.73	1,556,930	320,989.59
2032	2,762,532	277,949.66	1,626,783	318,767.18
2033	2,867,539	276,927.60	1,700,788	316,544.78
2034	2,997,055	275,905.53	1,798,694	314,322.37
2035	3,147,946	275,105.31	1,904,626	312,048.55
2036	3,313,619	274,305.09	2,009,600	309,774.73
2037	3,492,018	273,504.86	2,108,537	307,500.91
2038	3,692,015	272,704.64	2,213,690	305,227.09
2039	3,925,307	271,904.42	2,338,775	302,953.27
2040	4,215,156	272,418.38	2,503,366	303,121.43

2041	4,546,130	272,932.34	2,689,725	303,289.59
2042	4,920,230	273,446.30	2,897,813	303,457.75
2043	5,373,964	273,960.26	3,148,762	303,625.91
2044	5,911,326	274,474.22	3,446,706	303,794.08
2045	6,496,004	274,988.17	3,772,029	303,962.24

Supplementary Table 11. Percentage of disability-adjusted life years attributable to level 1 risk factors, by level 1 causes and age group, 2021, both sexes, China.

Risk factor	Percentage of disability-adjusted life years 1990	Percentage of disability-adjusted life years 2021
**Communicable, maternal, neonatal, and nutritional diseases**		
	**65+years**	
Behavioral risks	34.78(25.83,45.33)	36.08(27.28,45.83)
Environmental/occupational risks	24.45(13.22,33.49)	21.76(10.95,32.67)
Metabolic risks	4.40(2.98,6.21)	2.15(1.41,3.08)
	**65-69 years**	
Behavioral risks	38.57(29.77,49.33)	41.28(34.22,47.95)
Environmental/occupational risks	16.51(9.53,21.99)	12.03(6.35,17.25)
Metabolic risks	5.33(3.65,7.36)	2.99(2.05,4.24)
	**70-74years**	
Behavioral risks	36.56(28.04,46.38)	39.37(31.77,47.16)
Environmental/occupational risks	21.86(12.38,29.27)	17.08(8.88,24.52)
Metabolic risks	5.14(3.53,7.06)	3.27(2.25,4.52)
	**75-79years**	
Behavioral risks	32.78(24.88,41.84)	36.73(28.90,44.05)
Environmental/occupational risks	26.83(15.06,35.71)	21.94(11.16,30.97)
Metabolic risks	4.39(3.00,6.07)	2.58(1.73,3.57)
	**80-84years**	

Behavioral risks	30.15(22.71,37.37)	32.65(25.26,39.18)
Environmental/occupational risks	32.96(18.04,44.22)	26.57(13.54,37.62)
Metabolic risks	3.03(2.04,4.33)	1.67(1.13,2.38)
	**85-89 years**	
Behavioral risks	29.70(22.43,36.89)	31.92(23.91,39.37)
Environmental/occupational risks	36.11(19.73,48.56)	29.86(14.98,42.11)
Metabolic risks	1.92(1.29,2.68)	0.77(0.52,1.11)
	**90-94 years**	
Behavioral risks	29.97(22.86,36.80)	29.67(21.72,36.62)
Environmental/occupational risks	39.61(20.66,54.00)	31.48(15.81,44.70)
Metabolic risks	0.86(0.57,1.21)	0.27(0.19,0.37)
	**95+years**	
Behavioral risks	30.29(22.83,37.41)	29.23(21.25,36.32)
Environmental/occupational risks	39.59(20.53,53.99)	29.89(14.57,42.32)
Metabolic risks	0.69(0.47,1.02)	0.14(0.09,0.19)
**Injuries**		
	**65+years**	
Behavioral risks	5.77(4.24,7.49)	5.09(3.62,7.01)
Environmental/occupational risks	6.26(3.90,8.94)	0.43(-0.69,1.61)
Metabolic risks	18.18(14.84,21.93)	26.84(20.43,33.67)
	**65-69 years**	
Behavioral risks	6.09(4.72,7.68)	5.09(3.62,7.01)

Environmental/occupational risks	13.53(11.66,15.34)	0.43(-0.69,1.61)
Metabolic risks	13.03(9.01,17.79)	26.84(20.43,33.67)
	**70-74years**	
Behavioral risks	5.77(4.24,7.49)	5.86(4.51,7.35)
Environmental/occupational risks	6.26(3.90,8.94)	5.35(3.10,8.17)
Metabolic risks	18.18(14.84,21.93)	16.05(13.85,18.67)
	**75-79years**	
Behavioral risks	5.66(4.34,7.04)	5.09(3.62,7.01)
Environmental/occupational risks	0.89(-0.25,2.20)	0.43(-0.69,1.61)
Metabolic risks	20.88(18.15,23.94)	26.84(20.43,33.67)
	**80-84years**	
Behavioral risks	5.09(3.89,6.30)	4.52(3.34,5.75)
Environmental/occupational risks	-1.47(-2.17, -0.65)	-1.43(-2.04, -0.92)
Metabolic risks	26.81(23.32,30.82)	35.04(30.86,38.09)
	**85-89 years**	
Behavioral risks	5.77(4.24,7.49)	4.34(3.16,5.62)
Environmental/occupational risks	6.26(3.90,8.94)	42.68(37.45,46.19)
Metabolic risks	18.18(14.84,21.93)	-1.50(-2.06, -1.06)
	**90-94 years**	
Behavioral risks	4.58(3.56,5.71)	3.77(2.71,4.98)
Environmental/occupational risks	41.30(35.19,45.63)	49.44(42.25,54.81)
Metabolic risks	-1.68(-2.08, -1.27)	-1.03(-1.46, -0.71)

	**95+years**	
Behavioral risks	3.36(2.40,4.31)	2.78(1.95,3.75)
Environmental/occupational risks	47.20(39.24,52.44)	53.99(44.36,60.64)
Metabolic risks	-1.28(-1.62, -0.96)	-0.74(-1.07, -0.50)
**Non-communicable diseases**		
	**65+years**	
Behavioral risks	38.83(31.14,46.77)	33.82(25.05,42.78)
Environmental/occupational risks	40.03(34.31,45.49)	26.53(20.93,32.83)
Metabolic risks	28.77(23.02,34.52)	35.34(28.18,42.95)
	**65-69 years**	
Behavioral risks	41.30(34.24,47.78)	35.81(29.23,42.51)
Environmental/occupational risks	35.40(31.74,38.99)	22.33(18.84,25.89)
Metabolic risks	27.13(21.98,32.03)	31.30(26.03,36.02)
	**70-74years**	
Behavioral risks	40.79(33.51,47.31)	35.85(28.95,42.82)
Environmental/occupational risks	40.16(36.01,43.82)	25.58(21.63,29.51)
Metabolic risks	28.80(23.96,33.66)	34.26(28.46,38.90)
	**75-79years**	
Behavioral risks	37.20(30.11,44.39)	34.02(26.37,41.36)
Environmental/occupational risks	42.10(37.82,45.99)	27.25(22.69,31.74)
Metabolic risks	29.94(24.66,34.89)	36.16(30.57,41.20)
	**80-84years**	

Behavioral risks	34.07(26.92,41.29)	30.61(22.19,38.09)
Environmental/occupational risks	44.14(38.68,48.51)	29.27(23.86,34.13)
Metabolic risks	29.94(24.11,35.22)	37.81(30.82,43.93)
**85-89 years**		
Behavioral risks	34.20(26.69,41.55)	31.27(22.28,38.99)
Environmental/occupational risks	45.42(39.47,50.18)	30.73(24.46,35.88)
Metabolic risks	29.65(23.63,35.27)	39.27(31.90,46.16)
**90-94 years**		
Behavioral risks	34.07(26.41,41.53)	29.96(19.88,38.51)
Environmental/occupational risks	45.42(38.73,50.43)	30.87(24.01,36.53)
Metabolic risks	30.51(24.08,36.29)	40.61(31.57,47.87)
**95+years**		
Behavioral risks	31.05(21.95,39.49)	26.80(16.25,35.92)
Environmental/occupational risks	43.42(33.69,49.39)	29.35(21.39,35.76)
Metabolic risks	32.08(24.54,38.59)	40.32(30.91,48.62)

Supplementary Table 12. Percentage of disability-adjusted life years attributable to level 2 risk factors, by level 2 causes, 1990 and 2021, 65+ years, both sexes, China

Risk factor	Percentage of disability-adjusted life years, 1990	Risk factor	Percentage of disability-adjusted life years, 2021
**Cardiovascular diseases**			
High systolic blood pressure	52.05(35.37,69.14)	High systolic blood pressure	57.23(39.92,75.48)
Air pollution	39.35(32.34,46.85)	Air pollution	28.63(21.68,36.41)
Dietary risks	32.39(17.43,46.47)	Dietary risks	27.61(10.65,43.37)
Tobacco	19.04(14.71,23.95)	Tobacco	18.36(13.48,24.01)
High LDL cholesterol	10.03(4.62,16.20)	High LDL cholesterol	15.35(7.38,24.70)
Other environmental risks	9.61(-0.92,20.00)	High fasting plasma glucose	11.13(8.74,14.02)
Impaired kidney function	8.62(6.35,11.41)	Other environmental risks	9.55(-1.15,19.99)
High fasting plasma glucose	7.77(6.04,9.88)	Impaired kidney function	8.70(6.20,11.81)
Suboptimal temperature	7.77(6.43,9.33)	Suboptimal temperature	7.44(6.03,9.27)
High body-mass index	3.40(1.62,5.35)	High body-mass index	6.58(3.11,10.63)
Alcohol use	3.28(1.02,5.99)	Alcohol use	3.06(0.79,5.91)
Low physical activity	1.54(0.36,3.16)	Low physical activity	2.20(0.48,4.66)
**Chronic respiratory diseases**			
Air pollution	70.32(57.98,80.73)	Tobacco	47.67(34.61,61.09)
Tobacco	48.99(37.54,60.69)	Air pollution	42.25(30.86,57.76)
Occupational risks	20.22(12.98,27.70)	Occupational risks	19.57(12.23,27.69)
Suboptimal temperature	11.51(9.12,14.22)	Suboptimal temperature	10.15(7.65,13.14)

High body-mass index	0.26(0.11,0.44)	High body-mass index	0.41(0.18,0.66)
**Diabetes and kidney diseases**			
High fasting plasma glucose	72.90(59.49,88.41)	High fasting plasma glucose	78.95(63.40,96.14)
Impaired kidney function	44.69(38.29,52.77)	Impaired kidney function	39.29(32.29,46.45)
High body-mass index	19.59(8.74,33.90)	High body-mass index	34.87(16.23,55.85)
Dietary risks	19.58(8.15,30.75)	Dietary risks	20.08(6.62,33.25)
Air pollution	10.63(6.33,15.80)	Air pollution	12.00(7.02,18.06)
Tobacco	9.35(5.89,13.20)	High systolic blood pressure	10.36(3.91,19.42)
High systolic blood pressure	9.00(2.98,18.24)	Tobacco	9.43(5.76,13.82)
Low physical activity	6.73(2.63,11.49)	Low physical activity	7.67(3.11,13.16)
Suboptimal temperature	5.01(3.97,6.16)	Suboptimal temperature	4.39(3.44,5.64)
Other environmental risks	1.58(-0.18,4.31)	Other environmental risks	1.69(-0.22,4.47)
Alcohol use	0.55(0.15,1.12)	Alcohol use	1.06(0.37,2.03)
**Digestive diseases**			
Alcohol use	11.62(8.47,15.69)	Alcohol use	13.22(9.17,18.38)
Tobacco	5.50(3.70,7.48)	High body-mass index	7.06(1.55,13.15)
High body-mass index	2.55(0.52,4.89)	Tobacco	3.79(2.40,5.62)
Drug use	1.63(0.80,2.74)	Drug use	2.51(1.50,3.79)
**Enteric infections**			
Unsafe water, sanitation, and handwashing	89.18(41.82,134.25)	Unsafe water, sanitation, and handwashing	66.68(38.21,116.22)
**Maternal and neonatal disorders**			
Child and maternal malnutrition	64.61(45.19,88.94)	Child and maternal malnutrition	45.31(32.81,59.45)

**Mental disorders**			
Intimate partner violence	1.33(0.00,4.08)	Intimate partner violence	1.54(0.00,4.84)
Childhood maltreatment	0.46(0.16,1.00)	Childhood maltreatment	0.51(0.20,1.06)
Other environmental risks	0.13(0.03,0.28)	Other environmental risks	0.19(0.05,0.41)
**Musculoskeletal disorders**			
Occupational risks	9.76(5.55,15.54)	High body-mass index	9.01(0.55,21.14)
Tobacco	8.50(4.56,13.87)	Tobacco	5.73(3.10,9.38)
High body-mass index	4.29(0.25,10.34)	Occupational risks	5.33(3.01,8.84)
Impaired kidney function	0.35(0.20,0.57)	Impaired kidney function	0.35(0.20,0.56)
**Neoplasms**			
Tobacco	30.30(24.26,36.48)	Tobacco	34.09(26.20,43.36)
Dietary risks	10.42(2.05,21.47)	Air pollution	8.14(5.09,11.61)
Air pollution	8.21(5.54,10.98)	Dietary risks	6.32(2.17,13.15)
Alcohol use	3.31(2.54,4.25)	Alcohol use	3.28(2.42,4.38)
High fasting plasma glucose	1.81(0.21,3.55)	High fasting plasma glucose	2.82(0.31,5.43)
Occupational risks	1.50(1.04,2.02)	Occupational risks	2.77(1.87,3.91)
Unsafe sex	1.47(1.20,1.78)	High body-mass index	1.85(0.76,3.14)
Other environmental risks	0.77(-0.37,2.11)	Unsafe sex	1.24(0.92,1.59)
High body-mass index	0.66(0.33,1.05)	Other environmental risks	1.14(-0.58,3.12)
Low physical activity	0.60(0.28,1.03)	Low physical activity	0.79(0.36,1.32)
Drug use	0.39(0.19,0.67)	Drug use	0.73(0.43,1.06)
**Neurological disorders**			

High fasting plasma glucose	7.91(0.43,22.22)	High fasting plasma glucose	9.18(0.55,24.82)
Tobacco	2.33(-0.54,8.81)	High body-mass index	3.30(-0.28,13.48)
High body-mass index	0.21(-0.45,2.81)	Tobacco	2.07(-0.16,7.31)
Alcohol use	0.20(0.12,0.32)	Alcohol use	0.14(0.08,0.24)
**Nutritional deficiencies**			
Child and maternal malnutrition	94.51(72.07,122.02)	Child and maternal malnutrition	87.28(64.26,117.60)
**Other infectious diseases**			
Drug use	1.64(0.51,4.11)	Drug use	0.44(0.24,0.75)
**Respiratory infections and tuberculosis**			
Tobacco	28.85(20.41,37.91)	Tobacco	26.48(17.15,37.19)
Air pollution	21.90(3.45,35.35)	Air pollution	21.35(2.92,37.33)
Suboptimal temperature	7.09(5.13,9.08)	Suboptimal temperature	10.01(7.17,13.65)
Alcohol use	6.65(-5.07,24.77)	Alcohol use	3.42(-2.67,12.45)
High fasting plasma glucose	5.91(4.00,8.34)	High fasting plasma glucose	3.07(2.02,4.41)
Unsafe water, sanitation, and handwashing	4.59(-2.46,13.43)	Unsafe water, sanitation, and handwashing	2.14(-0.96,6.52)
High body-mass index	1.45(0.36,3.92)	High body-mass index	1.50(0.33,3.93)
Dietary risks	0.81(0.17,1.63)	Dietary risks	0.45(0.11,0.95)
Low physical activity	0.51(0.14,1.07)	Low physical activity	0.26(0.07,0.56)
**Self-harm and interpersonal violence**			
Alcohol use	5.17(2.29,8.66)	Alcohol use	6.14(2.80,10.43)
Intimate partner violence	2.04(1.37,2.87)	Intimate partner violence	2.73(1.76,3.94)
Low bone mineral density	1.06(0.84,1.32)	Low bone mineral density	1.12(0.86,1.43)

Drug use	0.88(0.20,2.20)	Drug use	0.34(0.08,0.83)
Tobacco	0.08(0.06,0.11)	Tobacco	0.07(0.05,0.10)
Suboptimal temperature	-4.17(-5.68, -2.49)	Suboptimal temperature	-3.95(-6.23, -2.07)
**Sense organ diseases**			
Occupational risks	8.02(4.88,11.99)	Occupational risks	8.38(5.27,12.39)
Air pollution	5.26(-2.22,9.99)	Air pollution	2.82(-0.65,6.32)
High fasting plasma glucose	2.22(-0.30,5.34)	High fasting plasma glucose	2.07(-0.25,4.85)
Tobacco	0.81(0.53,1.18)	Tobacco	0.59(0.38,0.87)
High body-mass index	0.12(0.00,0.35)	High body-mass index	0.33(-0.01,0.80)
**Substance use disorders**			
Drug use	59.22(48.66,71.12)	Alcohol use	70.97(50.85,96.31)
Alcohol use	40.77(30.26,54.01)	Drug use	29.03(22.10,37.00)
Childhood maltreatment	1.53(0.17,4.60)	Childhood maltreatment	2.60(0.27,8.19)
**Substance use disorders**			
Low bone mineral density	20.91(17.41,24.55)	Low bone mineral density	20.68(16.99,24.46)
Occupational risks	16.78(12.86,21.20)	Occupational risks	3.89(2.76,5.24)
Alcohol use	3.89(1.90,6.42)	Alcohol use	2.48(1.09,4.32)
Tobacco	2.17(1.62,2.81)	Tobacco	1.85(1.33,2.45)
Suboptimal temperature	-1.85(-2.42, -1.21)	Suboptimal temperature	-1.55(-2.41, -0.82)
**Unintentional injuries**			
Low bone mineral density	28.59(22.89,35.33)	Low bone mineral density	36.73(26.91,46.95)
Occupational risks	11.50(8.78,14.71)	Occupational risks	2.36(1.65,3.25)

Tobacco	2.24(1.58,3.04)	Tobacco	2.28(1.49,3.22)
Alcohol use	1.93(0.75,3.71)	Alcohol use	2.04(0.71,4.00)
Suboptimal temperature	-2.64(-3.45, -1.93)	Suboptimal temperature	-1.55(-2.04, -1.08)

Supplementary Table 13. Percentage of disability-adjusted life years attributable to level 2 risk factors, by level 2 causes and gender, 2021, 65+ years, China

Risk factor	Percentage of disability-adjusted life years	Risk factor	Percentage of disability-adjusted life years
**Female**		**Male**	
**Cardiovascular diseases**			
High systolic blood pressure	57.92(31.73,83.07)	High systolic blood pressure	56.68(35.16,78.37)
Air pollution	28.57(21.21,36.52)	Dietary risks	29.52(12.01,46.80)
Dietary risks	25.24(8.53,42.79)	Air pollution	28.68(21.20,37.78)
High LDL cholesterol	15.82(7.57,25.96)	Tobacco	25.51(18.53,34.36)
High fasting plasma glucose	11.02(8.15,14.44)	High LDL cholesterol	14.97(6.96,24.15)
Tobacco	9.48(6.17,13.77)	High fasting plasma glucose	11.21(8.44,14.68)
Kidney dysfunction	9.26(6.37,12.76)	Other environmental risks	10.68(-1.23,21.91)
Other environmental risks	8.16(-1.00,18.26)	Kidney dysfunction	8.25(5.64,11.59)
High body-mass index	7.71(3.49,12.94)	Non-optimal temperature	7.55(5.89,9.64)
Non-optimal temperature	7.30(5.58,9.41)	High body-mass index	5.68(2.71,9.43)
Low physical activity	2.85(0.62,6.36)	High alcohol use	5.13(1.32,9.97)
High alcohol use	0.50(0.11,1.14)	Low physical activity	1.68(0.16,4.20)
**Chronic respiratory diseases**			
Air pollution	43.31(29.45,62.12)	Tobacco	66.30(48.72,83.94)
Tobacco	23.09(12.25,36.49)	Air pollution	41.45(29.34,58.73)
Occupational risks	16.91(7.67,28.24)	Occupational risks	21.59(11.10,33.67)
Non-optimal temperature	9.58(6.56,13.35)	Non-optimal temperature	10.58(7.69,13.90)

High body-mass index	0.46(0.20,0.76)	High body-mass index	0.37(0.15,0.62)
**Diabetes and kidney diseases**			
High fasting plasma glucose	79.51(62.66,97.57)	High fasting plasma glucose	78.38(61.93,97.45)
Kidney dysfunction	38.17(30.48,47.00)	Kidney dysfunction	40.45(30.92,50.35)
High body-mass index	38.09(17.09,61.07)	High body-mass index	31.52(14.66,52.26)
Dietary risks	19.77(6.11,34.24)	Dietary risks	20.40(6.71,34.03)
Air pollution	12.25(7.06,18.28)	Tobacco	12.31(8.39,17.12)
High systolic blood pressure	10.61(2.78,22.94)	Air pollution	11.75(6.87,17.61)
Low physical activity	9.05(3.41,16.03)	High systolic blood pressure	10.10(3.23,20.16)
Tobacco	6.67(2.77,11.19)	Low physical activity	6.24(2.01,12.36)
Non-optimal temperature	4.29(3.15,5.68)	Non-optimal temperature	4.49(3.28,6.00)
Other environmental risks	1.44(-0.18,3.98)	High alcohol use	2.01(0.70,3.79)
High alcohol use	0.14(0.02,0.37)	Other environmental risks	1.95(-0.24,5.05)
**Diabetes and kidney diseases**			
High body-mass index	9.41(2.00,17.42)	High alcohol use	21.67(14.84,30.63)
High alcohol use	2.36(1.28,4.08)	Tobacco	6.14(3.87,9.15)
Drug use	1.38(0.67,2.46)	High body-mass index	5.23(1.16,9.92)
Tobacco	0.77(0.36,1.44)	Drug use	3.38(2.07,5.14)
**Enteric infections**			
Unsafe water, sanitation, and handwashing	66.07(32.71,141.13)	Unsafe water, sanitation, and handwashing	67.27(36.43,118.96)
**HIV/AIDS and sexually transmitted infections**			
Unsafe sex	94.79(66.42,128.99)	Unsafe sex	73.89(49.55,104.31)

Intimate partner violence	7.96(3.84,13.86)	Drug use	25.71(15.89,40.15)
Drug use	4.91(2.77,8.03)		
**Maternal and neonatal disorders**			
Child and maternal malnutrition	46.82(33.81,61.03)	Child and maternal malnutrition	46.82(33.81,61.03)
**Mental disorders**			
Intimate partner violence	2.64(0.00,8.26)	Childhood sexual abuse and bullying	0.56(0.12,1.60)
Childhood sexual abuse and bullying	0.48(0.19,0.96)	Other environmental risks	0.15(0.01,0.42)
Other environmental risks	0.22(0.07,0.42)		
**Musculoskeletal disorders**			
High body-mass index	9.96(0.39,23.58)	Tobacco	11.56(6.63,18.38)
Occupational risks	5.52(3.00,9.25)	High body-mass index	7.45(0.78,16.98)
Tobacco	2.20(0.94,4.28)	Occupational risks	5.01(2.80,8.41)
Kidney dysfunction	0.19(0.11,0.31)	Kidney dysfunction	0.61(0.34,1.00)
**Neoplasms**			
Tobacco	12.66(7.41,19.19)	Tobacco	46.00(34.68,59.74)
Air pollution	7.60(4.67,10.90)	Air pollution	8.44(5.04,12.45)
Dietary risks	7.14(2.37,14.13)	Dietary risks	5.86(1.94,13.20)
Unsafe sex	3.46(2.57,4.44)	High alcohol use	4.56(3.27,6.22)
High fasting plasma glucose	3.22(0.35,6.53)	Occupational risks	2.90(1.84,4.38)
High body-mass index	2.96(1.18,5.29)	High fasting plasma glucose	2.59(0.30,5.25)
Occupational risks	2.53(1.58,3.61)	High body-mass index	1.23(0.52,2.12)
Low physical activity	1.20(0.48,2.16)	Other environmental risks	1.18(-0.62,3.28)

Other environmental risks	1.08(-0.54,3.05)	Drug use	0.67(0.41,1.01)
High alcohol use	0.98(0.67,1.36)	Low physical activity	0.56(0.18,1.15)
Drug use	0.84(0.43,1.39)		
**Neurological disorders**			
High fasting plasma glucose	9.73(0.58,26.27)	High fasting plasma glucose	8.29(0.49,22.64)
High body-mass index	4.00(-0.45,15.72)	Tobacco	3.66(-1.02,15.04)
Tobacco	1.08(0.30,2.83)	High body-mass index	2.18(-0.07,9.38)
High alcohol use	0.02(0.01,0.04)	High alcohol use	0.33(0.18,0.56)
**Nutritional deficiencies**			
Child and maternal malnutrition	88.97(62.90,122.85)	Child and maternal malnutrition	83.88(63.57,111.56)
**Other infectious diseases**			
Drug use	0.21(0.10,0.37)	Drug use	0.65(0.36,1.20)
**Respiratory infections and tuberculosis**			
Air pollution	23.11(2.92,44.54)	Tobacco	34.66(23.71,47.70)
Tobacco	14.12(6.02,25.30)	Air pollution	20.19(2.95,35.71)
Non-optimal temperature	10.66(7.11,16.55)	Non-optimal temperature	9.59(6.60,13.03)
Unsafe water, sanitation, and handwashing	2.28(-1.12,7.23)	High alcohol use	5.32(-4.27,19.16)
High fasting plasma glucose	2.13(1.36,3.16)	High fasting plasma glucose	3.69(2.30,5.54)
High body-mass index	1.34(0.28,3.47)	Unsafe water, sanitation, and handwashing	2.05(-0.87,6.11)
High alcohol use	0.55(-0.24,2.86)	High body-mass index	1.61(0.35,4.48)
Dietary risks	0.32(0.07,0.72)	Dietary risks	0.53(0.12,1.20)
Low physical activity	0.21(0.05,0.46)	Low physical activity	0.30(0.07,0.70)

**Self-harm and interpersonal violence**			
Intimate partner violence	6.17(3.98,8.91)	High alcohol use	10.25(4.74,17.42)
Low bone mineral density	1.68(1.30,2.16)	Low bone mineral density	0.67(0.52,0.87)
High alcohol use	0.96(0.29,1.99)	Drug use	0.26(0.06,0.65)
Drug use	0.42(0.10,1.09)	Tobacco	0.09(0.07,0.13)
Tobacco	0.04(0.02,0.07)	Non-optimal temperature	-4.07(-6.69, -1.96)
Non-optimal temperature	-3.80(-6.26, -1.88)		
**Sense organ diseases**			
Occupational risks	6.98(4.31,10.35)	Occupational risks	10.05(6.35,14.77)
Air pollution	3.29(-0.76,7.37)	Air pollution	2.26(-0.52,5.11)
High fasting plasma glucose	2.36(-0.27,5.55)	High fasting plasma glucose	1.71(-0.22,4.12)
High body-mass index	0.42(-0.02,1.02)	Tobacco	1.08(0.70,1.60)
Tobacco	0.17(0.08,0.31)	High body-mass index	0.21(0.00,0.53)
**Substance use disorders**			
Drug use	50.57(35.58,69.14)	High alcohol use	79.29(57.00,106.49)
High alcohol use	49.42(33.16,70.75)	Drug use	20.71(16.11,25.53)
Childhood sexual abuse and bullying	1.28(0.13,3.71)	Childhood sexual abuse and bullying	3.11(0.26,10.30)
**Transport injuries**			
Low bone mineral density	25.73(20.50,31.48)	Low bone mineral density	17.90(14.30,21.69)
Occupational risks	1.55(1.02,2.23)	Occupational risks	5.17(3.49,7.16)
Tobacco	0.53(0.26,0.94)	High alcohol use	3.62(1.60,6.23)
High alcohol use	0.42(0.14,0.81)	Tobacco	2.58(1.86,3.41)

Non-optimal temperature	-1.42(-2.23, -0.73)	Non-optimal temperature	-1.62(-2.57, -0.85)
**Unintentional injuries**			
Low bone mineral density	45.52(33.27,58.80)	Low bone mineral density	28.17(20.15,35.94)
Occupational risks	0.91(0.57,1.35)	Occupational risks	3.79(2.48,5.35)
Tobacco	0.80(0.38,1.47)	Tobacco	3.73(2.40,5.26)
High alcohol use	0.41(0.11,0.90)	High alcohol use	3.63(1.26,7.11)
Non-optimal temperature	-1.45(-1.97, -0.98)	Non-optimal temperature	-1.66(-2.30, -1.12)

Supplementary Table 14. Percentage of disability-adjusted life years attributable to level 3 risk factors, by level 2 causes, 1990 and 2021, 65+ years, both sexes, China

Risk factor	Percentage of disability-adjusted life years 1990	Risk factor	Percentage of disability-adjusted life years 2021
**Cardiovascular diseases**			
Particulate matter pollution	39.35(32.34,46.85)	Particulate matter pollution	28.63(21.68,36.41)
Diet high in sodium	19.72(6.74,34.60)	Diet high in sodium	16.07(4.27,30.62)
Smoking	14.79(11.45,18.52)	Smoking	14.11(10.35,18.68)
Diet low in fruits	9.76(4.89,14.75)	Lead exposure	9.55(-1.15,19.99)
Lead exposure	9.61(-0.92,20.00)	Low temperature	6.90(5.69,8.16)
Low temperature	7.39(6.38,8.46)	Secondhand smoke	4.90(3.10,7.11)
Diet low in vegetables	5.06(3.21,7.14)	Diet low in fruits	4.81(2.16,7.90)
Secondhand smoke	4.92(3.29,6.81)	Diet low in whole grains	4.60(1.45,8.20)
Diet low in whole grains	2.91(0.66,5.32)	Diet low in polyunsaturated fatty acids	2.79(-7.08,10.98)
Diet low in fiber	2.23(0.23,4.83)	Diet low in nuts and seeds	1.93(0.47,3.74)
Diet low in nuts and seeds	1.89(0.51,3.32)	Diet low in seafood omega-3 fatty acids	1.23(0.21,2.56)
Diet low in polyunsaturated fatty acids	1.78(-4.83,6.90)	Diet low in fiber	1.13(0.20,2.91)
Diet low in seafood omega-3 fatty acids	1.77(0.34,3.20)	Diet low in legumes	0.94(-0.68,2.61)
Diet low in legumes	0.73(-0.54,1.97)	High temperature	0.58(0.08,1.50)
High temperature	0.41(-0.07,1.19)	Diet low in vegetables	0.56(0.10,1.37)
Diet high in trans fatty acids	0.12(0.01,0.33)	Diet high in processed meat	0.09(0.03,0.18)
Diet high in processed meat	0.04(0.01,0.07)	Diet high in trans fatty acids	0.09(0.01,0.29)

Diet high in sugar-sweetened beverages	0.01(0.00,0.02)	Diet high in sugar-sweetened beverages	0.05(0.01,0.10)
Diet high in red meat	-1.25(-5.64,2.31)	Diet high in red meat	-0.39(-4.10,2.36)
**Chronic respiratory diseases**			
Particulate matter pollution	67.68(55.95,78.34)	Smoking	42.15(31.64,53.21)
Smoking	42.83(34.26,52.07)	Particulate matter pollution	37.31(26.75,53.06)
Occupational particulate matter, gases, and fumes	19.75(12.49,27.19)	Occupational particulate matter, gases, and fumes	19.03(11.71,27.12)
Low temperature	11.19(9.21,13.15)	Low temperature	9.75(7.73,12.05)
Secondhand smoke	9.53(3.75,15.75)	Secondhand smoke	8.72(3.34,14.81)
Ambient ozone pollution	8.96(1.91,15.72)	Ambient ozone pollution	8.19(1.69,14.81)
High temperature	0.36(-0.34,1.39)	High temperature	0.45(-0.35,1.56)
Occupational carcinogens	0.29(0.21,0.38)	Occupational carcinogens	0.43(0.33,0.56)
Occupational asthmagens	0.18(0.11,0.30)	Occupational asthmagens	0.11(0.08,0.16)
**Diabetes and kidney diseases**			
Particulate matter pollution	10.63(6.33,15.80)	Particulate matter pollution	12.00(7.02,18.06)
Diet low in fruits	7.20(3.18,11.70)	Diet high in red meat	6.02(-0.39,13.59)
Smoking	5.49(4.22,7.09)	Smoking	5.56(4.12,7.38)
Low temperature	4.59(3.88,5.38)	Diet low in whole grains	5.15(1.41,9.25)
Diet low in whole grains	4.36(1.23,7.62)	Secondhand smoke	4.18(1.40,7.30)
Secondhand smoke	4.17(1.50,7.01)	Low temperature	3.92(3.16,4.76)
Diet high in sodium	3.75(0.75,9.21)	Diet low in fruits	3.76(1.61,6.48)
Diet high in red meat	3.06(-0.07,7.38)	Diet high in sodium	3.19(0.54,8.31)
Diet low in vegetables	2.58(1.12,4.68)	Diet high in processed meat	2.69(0.62,5.17)

Lead exposure	1.58(-0.18,4.31)	Lead exposure	1.69(-0.22,4.47)
Diet high in processed meat	1.28(0.28,2.32)	Diet high in sugar-sweetened beverages	1.10(0.46,2.05)
Diet low in fiber	0.56(0.25,0.99)	High temperature	0.50(-0.02,1.11)
High temperature	0.46(-0.03,1.00)	Diet low in fiber	0.33(0.12,0.67)
Diet high in sugar-sweetened beverages	0.25(0.10,0.49)	Diet low in vegetables	0.28(0.05,0.76)
**Digestive diseases**			
Smoking	5.50(3.70,7.48)	Smoking	3.79(2.40,5.62)
**Enteric infections**			
Unsafe water source	70.25(25.92,115.44)	Unsafe water source	50.17(16.64,95.56)
Unsafe sanitation	62.93(30.61,94.39)	Unsafe sanitation	35.10(21.80,61.81)
No access to handwashing facility	11.39(1.25,26.25)	No access to handwashing facility	3.25(0.37,7.75)
**Maternal and neonatal disorders**			
Low birth weight and short gestation	64.61(45.19,88.94)	Low birth weight and short gestation	45.31(32.81,59.45)
**Mental disorders**			
Childhood sexual abuse	0.46(0.16,1.00)	Childhood sexual abuse	0.51(0.20,1.06)
Lead exposure	0.13(0.03,0.28)	Lead exposure	0.19(0.05,0.41)
**Musculoskeletal disorders**			
Occupational ergonomic factors	9.76(5.55,15.54)	Smoking	5.73(3.10,9.38)
Smoking	8.50(4.56,13.87)	Occupational ergonomic factors	5.33(3.01,8.84)
**Neoplasms**			
Smoking	29.60(23.90,35.55)	Smoking	33.12(25.67,42.05)
Particulate matter pollution	8.21(5.54,10.98)	Particulate matter pollution	8.14(5.09,11.61)

Diet low in vegetables	3.36(-0.70,6.67)	Occupational carcinogens	2.77(1.87,3.91)
Diet high in sodium	2.24(0.00,11.16)	Secondhand smoke	2.17(0.24,4.32)
Secondhand smoke	1.54(0.17,2.99)	Diet high in red meat	1.91(0.00,4.06)
Diet low in milk	1.50(0.50,2.37)	Diet low in whole grains	1.84(0.73,2.91)
Occupational carcinogens	1.50(1.04,2.02)	Diet low in milk	1.77(0.60,2.87)
Diet low in whole grains	1.49(0.59,2.29)	Diet high in sodium	1.36(0.00,6.74)
Diet high in red meat	1.47(0.00,3.04)	Residential radon	1.14(-0.58,3.12)
Diet low in calcium	1.31(0.94,1.70)	Diet low in calcium	0.74(0.51,1.00)
Diet low in fruits	1.25(0.56,2.19)	Diet low in fruits	0.70(0.32,1.19)
Residential radon	0.77(-0.37,2.11)	Diet low in vegetables	0.32(-0.04,1.12)
Diet high in processed meat	0.12(-0.03,0.25)	Diet high in processed meat	0.23(-0.05,0.51)
Diet low in fiber	0.11(0.03,0.22)	Chewing tobacco	0.08(0.04,0.16)
Chewing tobacco	0.10(0.04,0.18)	Diet low in fiber	0.05(0.01,0.12)
**Neurological disorders**			
Smoking	2.33(-0.54,8.81)	Smoking	2.07(-0.16,7.31)
**Nutritional deficiencies**			
Iron deficiency	64.92(44.03,91.79)	Iron deficiency	63.35(41.58,92.17)
Child growth failure	29.33(25.14,33.68)	Child growth failure	23.35(18.89,28.19)
Vitamin A deficiency	0.27(0.14,0.44)	Vitamin A deficiency	0.58(0.30,0.96)
**Respiratory infections and tuberculosis**			
Smoking	24.08(18.27,30.91)	Particulate matter pollution	21.35(2.92,37.33)
Particulate matter pollution	21.90(3.45,35.35)	Smoking	20.01(14.49,26.76)

Low temperature	6.40(5.04,7.62)	Low temperature	8.74(6.83,11.25)
Secondhand smoke	5.76(1.87,9.82)	Secondhand smoke	7.71(2.49,14.02)
No access to handwashing facility	4.59(-2.46,13.43)	No access to handwashing facility	2.14(-0.96,6.52)
High temperature	0.77(-0.15,1.74)	High temperature	1.37(-0.41,3.29)
Diet low in whole grains	0.28(0.07,0.55)	Diet high in red meat	0.16(0.00,0.41)
Diet low in fruits	0.24(0.03,0.49)	Diet low in whole grains	0.14(0.04,0.29)
Diet high in red meat	0.18(0.00,0.45)	Diet low in fruits	0.08(0.01,0.17)
Diet low in vegetables	0.06(-0.01,0.13)	Diet high in processed meat	0.04(0.01,0.09)
Diet high in processed meat	0.05(0.01,0.11)	Diet high in sugar-sweetened beverages	0.02(0.01,0.04)
Diet high in sugar-sweetened beverages	0.01(0.00,0.02)	Diet low in vegetables	0.00(0.00,0.01)
**Self-harm and interpersonal violence**			
High temperature	0.61(-0.81,2.47)	High temperature	0.56(-0.77,2.24)
Smoking	0.08(0.06,0.11)	Smoking	0.07(0.05,0.10)
Low temperature	-4.82(-6.95, -2.36)	Low temperature	-4.55(-7.42, -1.51)
**Sense organ diseases**			
Occupational noise	8.02(4.88,11.99)	Occupational noise	8.38(5.27,12.39)
Particulate matter pollution	5.26(-2.22,9.99)	Particulate matter pollution	2.82(-0.65,6.32)
Smoking	0.81(0.53,1.18)	Smoking	0.59(0.38,0.87)
**Substance use disorders**			
Childhood sexual abuse	1.53(0.17,4.60)	Childhood sexual abuse	2.60(0.27,8.19)
**Transport injuries**			
Occupational injuries	16.78(12.86,21.20)	Occupational injuries	3.89(2.76,5.24)

Smoking	2.17(1.62,2.81)	Smoking	1.85(1.33,2.45)
High temperature	0.14(-0.36,0.72)	High temperature	0.14(-0.49,0.81)
Low temperature	-1.99(-2.67, -1.44)	Low temperature	-1.69(-2.71, -0.94)
**Unintentional injuries**			
Occupational injuries	11.50(8.78,14.71)	Occupational injuries	2.36(1.65,3.25)
Smoking	2.24(1.58,3.04)	Smoking	2.28(1.49,3.22)
High temperature	0.39(0.10,0.70)	High temperature	0.24(0.05,0.47)
Low temperature	-3.11(-3.81, -2.61)	Low temperature	-1.84(-2.29, -1.45)

Supplementary Table 15. Percentage of disability-adjusted life years attributable to level 3 risk factors, by all cause, 1990 and 2021, 65+ years, both sexes, China

**Leading risk factor, 1990**	**Percentage of disability-adjusted life years**	**Leading risk factor, 2021**	**Percentage of disability-adjusted life years**
Particulate matter pollution	30.49(26.27,34.83)	Particulate matter pollution	17.15(13.39,21.86)
Smoking	20.38(16.43,24.61)	Smoking	16.90(13.11,21.41)
Diet high in sodium	7.34(2.39,13.58)	Diet high in sodium	6.33(1.62,12.55)
Low temperature	5.18(4.52,5.84)	Low temperature	3.85(3.18,4.54)
Secondhand smoke	4.29(2.15,6.63)	Lead exposure	3.62(-0.43,7.55)
Occupational particulate matter, gases, and fumes	4.15(2.62,5.71)	Secondhand smoke	3.46(1.71,5.46)
Diet low in fruits	3.83(1.93,5.77)	Diet low in whole grains	2.26(0.74,3.96)
Lead exposure	3.40(-0.33,7.09)	Occupational particulate matter, gases, and fumes	2.08(1.28,2.96)
Diet low in vegetables	2.36(1.31,3.47)	Diet low in fruits	2.08(0.97,3.37)
Ambient ozone pollution	1.88(0.40,3.30)	Diet low in polyunsaturated fatty acids	1.03(-2.62,4.06)
Diet low in whole grains	1.39(0.37,2.43)	Ambient ozone pollution	0.89(0.18,1.62)
Diet low in fiber	0.81(0.10,1.75)	Diet low in nuts and seeds	0.71(0.17,1.39)
Diet low in nuts and seeds	0.66(0.18,1.16)	Occupational carcinogens	0.54(0.38,0.75)
Diet low in polyunsaturated fatty acids	0.62(-1.69,2.41)	Diet high in red meat	0.47(-0.37,1.05)
Diet low in seafood omega-3 fatty acids	0.62(0.12,1.12)	Diet low in seafood omega-3 fatty acids	0.45(0.08,0.95)
Occupational ergonomic factors	0.34(0.19,0.53)	Diet low in fiber	0.44(0.08,1.13)
Occupational injuries	0.32(0.25,0.41)	Occupational noise	0.42(0.27,0.62)
Iron deficiency	0.30(0.21,0.43)	Diet low in legumes	0.35(-0.25,0.96)

Occupational carcinogens	0.29(0.22,0.37)	High temperature	0.32(0.11,0.65)
High temperature	0.28(0.08,0.60)	Diet low in milk	0.32(0.11,0.51)
Occupational noise	0.26(0.16,0.40)	Diet low in vegetables	0.28(0.05,0.70)
Diet low in legumes	0.25(-0.19,0.69)	Occupational ergonomic factors	0.25(0.14,0.42)
Diet low in milk	0.23(0.08,0.36)	Residential radon	0.20(-0.10,0.56)
Diet low in calcium	0.20(0.14,0.26)	Diet high in processed meat	0.20(0.03,0.38)
No access to handwashing facility	0.19(-0.09,0.54)	Iron deficiency	0.19(0.12,0.28)
Child growth failure	0.14(0.12,0.16)	Diet low in calcium	0.13(0.09,0.18)
Unsafe water source	0.13(0.05,0.22)	Occupational injuries	0.09(0.07,0.13)
Residential radon	0.12(-0.06,0.32)	Child growth failure	0.07(0.06,0.08)
Unsafe sanitation	0.12(0.06,0.18)	Diet high in sugar-sweetened beverages	0.07(0.03,0.13)
Diet high in processed meat	0.07(0.01,0.14)	No access to handwashing facility	0.04(-0.02,0.11)
Diet high in trans fatty acids	0.04(0.00,0.11)	Diet high in trans fatty acids	0.03(0.00,0.11)
Occupational asthmagens	0.04(0.02,0.06)	Low birth weight and short gestation	0.02(0.02,0.03)
Chewing tobacco	0.01(0.01,0.03)	Childhood sexual abuse	0.02(0.01,0.03)
Low birth weight and short gestation	0.01(0.01,0.02)	Unsafe water source	0.02(0.01,0.03)
Diet high in sugar-sweetened beverages	0.01(0.00,0.02)	Chewing tobacco	0.02(0.01,0.03)
Childhood sexual abuse	0.01(0.00,0.02)	Occupational asthmagens	0.01(0.01,0.02)
Vitamin A deficiency	0.00(0.00,0.00)	Unsafe sanitation	0.01(0.01,0.02)
Diet high in red meat	-0.11(-1.31,0.84)	Vitamin A deficiency	0.00(0.00,0.00)

Supplementary Table 16. The contribution of ageing, population and epidemiological change to disability-adjusted life years for all cause and level 1 causes in China

Cause	Overall difference	Aging	Population	Epidemiological change	Percent change of Aging	Percent change of Population	Percent change of Epidemiological change
All causes	95617163.49	10446096.51	148764148.50	-63593081.50	10.92	155.58	-66.51
Non-communicable diseases	91363672.84	9551078.89	137341645.00	-55529051.02	10.45	150.32	-60.78
Injuries	4123980.76	261459.19	5563689.58	-1701168.01	6.34	134.91	-41.25
Communicable, maternal, neonatal, and nutritional diseases	-21245.08	629493.53	5809528.01	-6460266.62	-2963.01	-27345.29	30408.30

Supplementary Table 17. The contribution of ageing, population and epidemiological change to disability-adjusted life years for level 2 causes in China

Overall difference	Aging change	Population change	Epidemiological change	Percent change of Aging	Percent change of Population	Percent change of Epidemiological change
139085.86	77495.51	603712.11	-542121.76	55.72	434.06	-389.77
1037488.98	175384.75	4222365.76	-3360261.53	16.90	406.98	-323.88
2124018.23	66629.89	2545668.57	-488280.23	3.14	119.85	-22.99
37229973.95	4883087.53	53051786.97	-20704900.55	13.12	142.50	-55.61
1743061.48	2647386.28	25376311.31	-26280636.10	151.88	1455.85	-1507.73
5744331.20	-4659.02	5916536.89	-167546.66	-0.08	103.00	-2.92
220541.43	14550.86	1408310.55	-1202319.98	6.60	638.57	-545.17
19220700.19	60537.24	24211191.58	-5051028.63	0.31	125.96	-26.28
8781649.78	1312410.96	7089302.62	379936.19	14.94	80.73	4.33
2697922.12	-14198.38	2483199.61	228920.89	-0.53	92.04	8.49
774388.37	22469.15	765468.62	-13549.40	2.90	98.85	-1.75
6325898.93	209185.76	5957681.29	159031.87	3.31	94.18	2.51
21215.97	-542.31	313108.20	-291349.92	-2.56	1475.81	-1373.26
1104249.69	-15473.54	1266164.61	-146441.38	-1.40	114.66	-13.26
2799189.64	262381.87	2889214.42	-352406.64	9.37	103.22	-12.59
154220.33	-2232.28	71074.46	85378.14	-1.45	46.09	55.36
-203864.32	532217.61	4264701.88	-5000783.80	-261.06	-2091.93	2453.00
-107680.35	17605.74	188818.71	-314104.80	-16.35	-175.35	291.70
-105625.23	7591.62	316782.52	-429999.37	-7.19	-299.91	407.10

81402.67	-2642.36	51330.13	32714.90	-3.25	63.06	40.19
161424.82	-4304.63	301319.79	-135590.34	-2.67	186.66	-84.00
5522814.79	197149.37	5420811.95	-95146.53	3.57	98.15	-1.72

Supplementary Table 18. The contribution of ageing, population and epidemiological change to disability-adjusted life years for level 3 cause in China

Cause	Overall difference	Aging change	Population change	Epidemiological change	percent change of Aging	percent change of Population	percent change of Epidemiological change
Acne vulgaris	8285.28	-226.44	7238.12	1273.60	-2.73	87.36	15.37
Acute glomerulonephritis	2414.03	2594.69	76858.41	-77039.07	107.48	3183.82	-3191.31
Acute hepatitis	-103674.35	-1403.17	144867.53	-247138.71	1.35	-139.73	238.38
Adverse effects of medical treatment	-6765.47	1773.34	50340.79	-58879.59	-26.21	-744.08	870.30
Age-related and other hearing loss	4235275.24	126139.97	3744770.41	364364.86	2.98	88.42	8.60
Alcohol use disorders	170122.37	-2660.47	157855.66	14927.19	-1.56	92.79	8.77
Alopecia areata	8221.05	-252.94	8568.36	-94.38	-3.08	104.22	-1.15
Alzheimer's disease and other dementias	6739175.82	1209992.62	5221634.31	307548.90	17.95	77.48	4.56
Animal contact	-7498.82	-243.01	38965.28	-46221.09	3.24	-519.62	616.38
Anxiety disorders	675795.16	-2285.22	673021.06	5059.32	-0.34	99.59	0.75
Aortic aneurysm	64775.41	1731.37	48644.35	14399.68	2.67	75.10	22.23
Appendicitis	-7950.82	2576.13	36034.53	-46561.48	-32.40	-453.22	585.62
Asthma	-51217.51	50154.74	708657.36	-810029.61	-97.92	-1383.62	1581.55
Atrial fibrillation and flutter	927278.35	174411.07	788765.21	-35897.92	18.81	85.06	-3.87
Attention-deficit/hyperactivity disorder	943.63	-79.84	1176.46	-153.00	-8.46	124.67	-16.21
Autism spectrum disorders	118945.60	-3327.88	102529.37	19744.11	-2.80	86.20	16.60
Bacterial skin diseases	-17933.09	11823.24	60801.97	-90558.29	-65.93	-339.05	504.98

Bipolar disorder	55724.62	-936.97	56602.42	59.17	-1.68	101.58	0.11
Bladder cancer	335139.42	26241.84	480314.67	-171417.09	7.83	143.32	-51.15
Blindness and vision loss	1833418.11	82681.98	1964090.54	-213354.42	4.51	107.13	-11.64
Brain and central nervous system cancer	415877.69	-5333.40	385056.71	36154.38	-1.28	92.59	8.69
Breast cancer	520080.85	583.55	493950.65	25546.65	0.11	94.98	4.91
Cardiomyopathy and myocarditis	224915.60	25459.55	175700.09	23755.96	11.32	78.12	10.56
Cervical cancer	207202.57	-3565.95	330278.38	-119509.86	-1.72	159.40	-57.68
Chronic kidney disease	2027398.49	159888.64	2277107.77	-409597.92	7.89	112.32	-20.20
Chronic obstructive pulmonary disease	1631228.06	2589559.18	24443486.17	-25401817.28	158.75	1498.47	-1557.22
Cirrhosis and other chronic liver diseases	245788.21	1275.16	1635367.25	-1390854.19	0.52	665.36	-565.88
Colon and rectum cancer	2187673.47	50690.92	2200422.41	-63439.86	2.32	100.58	-2.90
Conflict and terrorism	193.72	-7.75	198.06	3.41	-4.00	102.24	1.76
Congenital birth defects	41517.68	-1298.79	46128.25	-3311.78	-3.13	111.11	-7.98
Cystic echinococcosis	210.78	36.08	2308.70	-2134.00	17.12	1095.30	-1012.42
Cysticercosis	22717.97	1202.28	66258.29	-44742.61	5.29	291.66	-196.95
Decubitus ulcer	39348.53	5624.30	18637.07	15087.17	14.29	47.36	38.34
Dengue	24.24	6.06	94.55	-76.36	24.99	389.96	-314.95
Depressive disorders	1375554.84	-218.70	1177000.08	198773.46	-0.02	85.57	14.45
Dermatitis	128408.57	-615.09	129408.07	-384.41	-0.48	100.78	-0.30
Diabetes mellitus	3493002.27	34666.04	3066845.78	391490.46	0.99	87.80	11.21
Diarrheal diseases	-109075.05	17602.37	184066.60	-310744.02	-16.14	-168.75	284.89
Dietary iron deficiency	81749.90	17033.06	382520.54	-317803.69	20.84	467.92	-388.75

Drowning	153483.28	10074.45	264387.17	-120978.34	6.56	172.26	-78.82
Drug use disorders	-8697.55	-1644.16	143464.14	-150517.53	18.90	-1649.48	1730.57
Encephalitis	11167.81	-51.27	18369.49	-7150.42	-0.46	164.49	-64.03
Endocarditis	2086.02	2213.64	21910.48	-22038.09	106.12	1050.35	-1056.47
Endocrine, metabolic, blood, and immune disorders	410521.15	9012.67	427622.76	-26114.28	2.20	104.17	-6.36
Environmental heat and cold exposure	10423.87	6443.49	61937.80	-57957.42	61.81	594.19	-556.01
Esophageal cancer	1555361.35	-17743.54	3311640.98	-1738536.09	-1.14	212.92	-111.78
Exposure to forces of nature	2869.04	364.79	9731.14	-7226.88	12.71	339.18	-251.89
Exposure to mechanical forces	139867.44	1219.75	134635.55	4012.15	0.87	96.26	2.87
Eye cancer	4501.78	350.03	6004.86	-1853.11	7.78	133.39	-41.16
Falls	2227777.55	223482.86	1868476.74	135817.95	10.03	83.87	6.10
Fire, heat, and hot substances	50345.16	11473.35	144585.30	-105713.48	22.79	287.19	-209.98
Food-borne trematodiases	46432.51	-1957.98	144650.88	-96260.39	-4.22	311.53	-207.31
Foreign body	78363.64	6030.96	78358.46	-6025.78	7.70	99.99	-7.69
Fungal skin diseases	62253.34	3327.01	59892.55	-966.22	5.34	96.21	-1.55
Gallbladder and biliary diseases	469922.70	46847.85	693797.26	-270722.41	9.97	147.64	-57.61
Gallbladder and biliary tract cancer	281400.02	6108.17	343370.61	-68078.76	2.17	122.02	-24.19
Gout	150206.34	723.55	116223.75	33259.05	0.48	77.38	22.14
Gynecological diseases	113907.34	70.77	183509.63	-69673.06	0.06	161.10	-61.17
Headache disorders	544128.61	-12578.71	528401.42	28305.89	-2.31	97.11	5.20
Hemoglobinopathies and hemolytic anemias	79843.22	8669.53	225064.45	-153890.77	10.86	281.88	-192.74

HIV/AIDS	147080.63	-2230.96	56672.39	92639.19	-1.52	38.53	62.99
Hodgkin lymphoma	-2459.47	-315.02	34942.14	-37086.59	12.81	-1420.72	1507.91
Hypertensive heart disease	1286381.13	531456.48	4609217.91	-3854293.26	41.31	358.31	-299.62
Idiopathic developmental intellectual disability	5035.16	-136.83	8171.26	-2999.26	-2.72	162.28	-59.57
Idiopathic epilepsy	110389.50	2490.15	144708.75	-36809.40	2.26	131.09	-33.35
Inflammatory bowel disease	28741.91	8840.09	64965.62	-45063.80	30.76	226.03	-156.79
Inguinal, femoral, and abdominal hernia	17753.68	1797.82	30972.41	-15016.55	10.13	174.46	-84.58
Interpersonal violence	48452.43	-2819.46	166242.48	-114970.59	-5.82	343.10	-237.29
Interstitial lung disease and pulmonary sarcoidosis	89841.76	2403.80	83468.93	3969.03	2.68	92.91	4.42
Intestinal nematode infections	-39313.08	-487.61	43033.07	-81858.55	1.24	-109.46	208.22
Invasive Non-typhoidal Salmonella (iNTS)	1319.73	-55.78	3145.71	-1770.20	-4.23	238.36	-134.13
Iodine deficiency	23809.38	-411.75	23410.84	810.29	-1.73	98.33	3.40
Ischemic heart disease	17822930.27	1845219.84	14139042.06	1838668.37	10.35	79.33	10.32
Kidney cancer	200226.43	4392.54	158759.72	37074.16	2.19	79.29	18.52
Larynx cancer	113913.29	-1465.05	210933.97	-95555.64	-1.29	185.17	-83.88
Leishmaniasis	-140.65	-12.69	756.91	-884.87	9.02	-538.14	629.12
Leprosy	-23.74	1.21	206.13	-231.07	-5.09	-868.43	973.52
Leukemia	256982.80	-3475.47	359721.78	-99263.51	-1.35	139.98	-38.63
Lip and oral cavity cancer	179829.66	1186.66	176856.42	1786.58	0.66	98.35	0.99
Liver cancer	960030.80	-7051.33	1170109.76	-203027.63	-0.73	121.88	-21.15
Low back pain	1881364.85	10318.02	2463769.53	-592722.69	0.55	130.96	-31.50
Lower extremity peripheral arterial disease	91630.89	5775.06	93049.02	-7193.19	6.30	101.55	-7.85

Lower respiratory infections	635610.41	492560.07	2485143.60	-2342093.26	77.49	390.99	-368.48
Malignant neoplasm of bone and articular cartilage	158219.01	-265.64	103153.46	55331.19	-0.17	65.20	34.97
Malignant skin melanoma	32670.20	1105.62	33099.54	-1534.96	3.38	101.31	-4.70
Maternal disorders	0.00	0.00	0.00	0.00	-14.37	206.68	-92.31
Meningitis	-8183.91	3005.34	66880.64	-78069.89	-36.72	-817.22	953.94
Mesothelioma	18268.42	158.95	17290.99	818.49	0.87	94.65	4.48
Motor neuron disease	21572.88	-642.19	15419.51	6795.56	-2.98	71.48	31.50
Multiple myeloma	138009.67	-1037.33	67358.00	71688.99	-0.75	48.81	51.94
Multiple sclerosis	1931.49	-1.42	1297.99	634.93	-0.07	67.20	32.87
Nasopharynx cancer	41745.31	-5335.76	305853.58	-258772.50	-12.78	732.67	-619.88
Neck pain	756703.78	-8133.05	708803.13	56033.70	-1.07	93.67	7.40
Neonatal disorders	81402.67	-2642.36	51330.13	32714.90	-3.25	63.06	40.19
Neuroblastoma and other peripheral nervous cell tumors	7321.67	-2.77	3384.34	3940.10	-0.04	46.22	53.81
Non-Hodgkin lymphoma	293283.22	-2248.04	311755.73	-16224.47	-0.77	106.30	-5.53
Non-melanoma skin cancer	152811.91	7146.68	126712.95	18952.28	4.68	82.92	12.40
Non-rheumatic valvular heart disease	41797.55	4750.33	49221.23	-12174.01	11.37	117.76	-29.13
Oral disorders	1173723.98	32855.61	1223050.29	-82181.92	2.80	104.20	-7.00
Osteoarthritis	1659894.38	19162.93	1419091.65	221639.81	1.15	85.49	13.35
Other cardiovascular and circulatory diseases	181988.61	12054.63	206451.50	-36517.53	6.62	113.44	-20.07
Other chronic respiratory diseases	40739.61	3590.64	28477.49	8671.49	8.81	69.90	21.29

Other digestive diseases	7155.64	22068.77	216001.53	-230914.66	308.41	3018.62	-3227.03
Other intestinal infectious diseases	88.57	84.23	661.40	-657.06	95.10	746.76	-741.85
Other malignant neoplasms	218484.51	3899.77	375436.21	-160851.47	1.78	171.84	-73.62
Other mental disorders	257374.34	739.67	255856.30	778.38	0.29	99.41	0.30
Other musculoskeletal disorders	1082577.15	-32309.45	995251.30	119635.30	-2.98	91.93	11.05
Other neglected tropical diseases	5914.16	598.21	18285.76	-12969.81	10.11	309.19	-219.30
Other neoplasms	67325.26	938.08	46527.52	19859.66	1.39	69.11	29.50
Other neurological disorders	127881.75	1353.62	63515.78	63012.36	1.06	49.67	49.27
Other nutritional deficiencies	23144.98	5357.80	21969.49	-4182.31	23.15	94.92	-18.07
Other pharynx cancer	25825.81	-519.23	56367.72	-30022.69	-2.01	218.26	-116.25
Other sense organ diseases	257205.58	363.82	248820.34	8021.43	0.14	96.74	3.12
Other skin and subcutaneous diseases	168061.09	3151.03	133307.12	31602.94	1.87	79.32	18.80
Other transport injuries	24861.72	385.60	65109.94	-40633.82	1.55	261.89	-163.44
Other unintentional injuries	49346.35	601.72	130462.35	-81717.72	1.22	264.38	-165.60
Other unspecified infectious diseases	7514.75	2345.08	38270.62	-33100.96	31.21	509.27	-440.48
Otitis media	13695.76	-116.17	22890.76	-9078.83	-0.85	167.14	-66.29
Ovarian cancer	143336.12	-2996.92	155627.80	-9294.76	-2.09	108.58	-6.48
Pancreatic cancer	1027987.72	-469.60	823893.11	204564.21	-0.05	80.15	19.90
Pancreatitis	70840.89	4400.12	110187.19	-43746.42	6.21	155.54	-61.75
Paralytic ileus and intestinal obstruction	60348.85	24316.63	200526.46	-164494.24	40.29	332.28	-272.57
Parkinson's disease	1236569.72	111796.89	1114324.88	10447.95	9.04	90.11	0.84
Pneumoconiosis	32469.56	1677.91	112221.37	-81429.73	5.17	345.62	-250.79

Poisonings	100977.60	1160.18	107333.85	-7516.44	1.15	106.29	-7.44
Police conflict and executions	7061.49	-124.62	3732.30	3453.81	-1.76	52.85	48.91
Prostate cancer	401530.26	27422.63	334468.69	39638.95	6.83	83.30	9.87
Protein-energy malnutrition	8302.37	55544.36	173663.89	-220905.88	669.02	2091.74	-2660.76
Pruritus	37778.53	242.23	32811.98	4724.32	0.64	86.85	12.51
Psoriasis	117668.52	-1259.45	93181.69	25746.29	-1.07	79.19	21.88
Pulmonary Arterial Hypertension	51343.06	6833.39	66250.38	-21740.71	13.31	129.03	-42.34
Rabies	1025.47	-96.20	2683.62	-1561.95	-9.38	261.70	-152.32
Rheumatic heart disease	-286040.19	93426.93	1621865.80	-2001332.92	-32.66	-567.01	699.67
Rheumatoid arthritis	213584.70	5578.98	213397.53	-5391.82	2.61	99.91	-2.52
Road injuries	1079387.97	-15859.14	1201054.67	-105807.56	-1.47	111.27	-9.80
Scabies	122282.49	2654.67	119768.72	-140.90	2.17	97.94	-0.12
Schistosomiasis	-11842.30	66.50	29353.96	-41262.77	-0.56	-247.87	348.44
Schizophrenia	208548.77	-7952.61	208842.66	7658.72	-3.81	100.14	3.67
Self-harm	164833.79	17502.69	1238137.71	-1090806.61	10.62	751.14	-661.76
Sexually transmitted infections excluding HIV	7139.70	-1.32	14402.07	-7261.05	-0.02	201.72	-101.70
Soft tissue and other extraosseous sarcomas	23817.14	1331.53	44686.82	-22201.21	5.59	187.62	-93.22
Stomach cancer	1779354.47	-11369.72	5343634.82	-3552910.63	-0.64	300.31	-199.67
Stroke	16820887.25	2179755.24	31231668.94	-16590536.93	12.96	185.67	-98.63
Testicular cancer	6608.66	229.78	7539.23	-1160.35	3.48	114.08	-17.56
Tetanus	-16356.00	87.08	19334.39	-35777.47	-0.53	-118.21	218.74
Thyroid cancer	54693.37	2011.70	64278.47	-11596.80	3.68	117.53	-21.20

Tracheal, bronchus, and lung cancer	7357405.00	-8583.62	6197128.74	1168859.88	-0.12	84.23	15.89
Trachoma	-2355.19	153.28	4045.46	-6553.93	-6.51	-171.77	278.28
Tuberculosis	-804483.38	9739.39	1581096.73	-2395319.51	-1.21	-196.54	297.75
Typhoid and paratyphoid	-13.60	-25.07	944.99	-933.52	184.30	-6947.05	6862.74
Upper digestive system diseases	140649.96	61887.05	1222617.07	-1143854.16	44.00	869.26	-813.26
Upper respiratory infections	-60990.20	29023.73	171743.52	-261757.46	-47.59	-281.59	429.18
Urinary diseases and male infertility	304504.85	17320.09	440293.19	-153108.43	5.69	144.59	-50.28
Urticaria	44157.24	-728.47	45402.33	-516.62	-1.65	102.82	-1.17
Uterine cancer	56241.80	-1482.80	130630.78	-72906.18	-2.64	232.27	-129.63
Varicella and herpes zoster	3906.46	3608.55	29059.84	-28761.94	92.37	743.89	-736.27
Vascular intestinal disorders	4237.95	1375.13	11896.44	-9033.62	32.45	280.71	-213.16
Viral skin diseases	55856.82	-1270.94	56450.65	677.11	-2.28	101.06	1.21
Vitamin A deficiency	2079.23	-27.94	2147.35	-40.17	-1.34	103.28	-1.93

Supplementary Table 19. Change in disability-adjusted life years associated with population ageing in China, 1990-2021

Year	All cause	Non-communicable diseases	Injuries	Communicable, maternal, neonatal, and nutritional diseases
1990-1991	235244.71	214962.04	4239.80	16042.87
1990-1992	493542.99	451310.74	8772.49	33459.76
1990-1993	726763.44	664527.31	12779.14	49457.00
1990-1994	948798.95	866750.58	16798.61	65249.75
1990-1995	1210417.38	1105680.97	21427.19	83309.22
1990-1996	1516337.63	1385543.83	27050.67	103743.14
1990-1997	1850441.77	1692059.44	33346.72	125035.61
1990-1998	2186906.08	2002063.78	40056.65	144785.66
1990-1999	2431150.98	2227682.13	45122.93	158345.92
1990-2000	2755150.67	2527707.50	52006.69	175436.48
1990-2001	3067584.85	2817962.87	59337.29	190284.69
1990-2002	3376432.92	3106311.09	66771.53	203350.30
1990-2003	3860598.46	3557651.76	77769.97	225176.72
1990-2004	4402105.40	4064982.40	89715.99	247407.01
1990-2005	4929743.79	4558901.24	101101.59	269740.96
1990-2006	5388927.56	4988128.21	109771.94	291027.41
1990-2007	6084789.03	5633805.85	123687.05	327296.13
1990-2008	6830494.45	6318346.78	145528.28	366619.38

1990-2009	7572088.28	7016888.11	149579.00	405621.17
1990-2010	8262808.94	7658868.20	161487.38	442453.36
1990-2011	8575322.72	7945156.53	166797.48	463368.71
1990-2012	8697336.71	8047075.96	171564.88	478695.87
1990-2013	9183021.60	8487928.92	185804.71	509287.97
1990-2014	9001816.87	8300569.42	190581.87	510665.58
1990-2015	9358569.12	8617590.57	203295.78	537682.78
1990-2016	9536425.82	8767400.29	214074.49	554951.03
1990-2017	9716447.84	8919552.55	224781.42	572113.87
1990-2018	9593405.11	8788660.38	229364.30	575380.43
1990-2019	9872178.31	9038635.34	241412.63	592130.35
1990-2020	9839830.44	8980230.91	249400.14	593823.80
1990-2021	10446096.51	9551078.89	261459.19	629493.53

Supplementary Table 20. Top 15 diseases with the disability-adjusted life years change associated with population ageing between 1990 and 2021 in China

Disease	Disability-adjusted life years related to population ageing
Chronic obstructive pulmonary disease	2589559.18
Stroke	2179755.24
Ischemic heart disease	1845219.84
Alzheimer's disease and other dementias	1209992.62
Hypertensive heart disease	531456.48
Lower respiratory infections	492560.07
Falls	223482.86
Atrial fibrillation and flutter	174411.07
Chronic kidney disease	159888.64
Age-related and other hearing loss	126139.97
Parkinson's disease	111796.89
Rheumatic heart disease	93426.93
Blindness and vision loss	82681.98
Upper digestive system diseases	61887.05
Protein-energy malnutrition	55544.36
